# Video Games as a Potential Modality for Behavioral Health Services for Young Adult Veterans: Exploratory Analysis

**DOI:** 10.2196/games.9327

**Published:** 2018-07-26

**Authors:** Sean Grant, Asya Spears, Eric R Pedersen

**Affiliations:** ^1^ RAND Corporation Santa Monica, CA United States

**Keywords:** behavioral health, replication, veterans, video games

## Abstract

**Background:**

Improving the reach of behavioral health services to young adult veterans is a policy priority.

**Objective:**

The objective of our study was to explore differences in video game playing by behavioral health need for young adult veterans to identify potential conditions for which video games could be used as a modality for behavioral health services.

**Methods:**

We replicated analyses from two cross-sectional, community-based surveys of young adult veterans in the United States and examined the differences in time spent playing video games by whether participants screened positive for behavioral health issues and received the required behavioral health services.

**Results:**

Pooling data across studies, participants with a positive mental health screen for depression or posttraumatic stress disorder (PTSD) spent 4.74 more hours per week (95% CI 2.54-6.94) playing video games. Among participants with a positive screen for a substance use disorder, those who had received substance use services since discharge spent 0.75 more days per week (95% CI 0.28-1.21) playing video games than participants who had not received any substance use services since discharge.

**Conclusions:**

We identified the strongest evidence that participants with a positive PTSD or depression screen and participants with a positive screen for a substance use disorder who also received substance use services since their discharge from active duty spent more time playing video games. Future development and evaluation of video games as modalities for enhancing and increasing access to behavioral health services should be explored for this population.

## Introduction

Behavioral health issues such as posttraumatic stress disorder (PTSD), depressive disorders, and substance use disorders (SUDs) are common diagnoses among veterans from recent conflicts in Iraq and Afghanistan [1,2]. However, at best, only half of the veterans with a documented behavioral health need actually receive behavioral health services [3,4]. Moreover, nearly 40% of the veterans from recent conflicts in Iraq and Afghanistan have never sought services through the Veterans Health Administration (VHA) for any reason since separation from active military duty [5,6]. Young adult veterans are particularly at risk for unmet behavioral health needs, as they are less likely to seek behavioral health services than older veterans [7,8], have higher rates of behavioral health issues than older veterans [1], and report poorer behavioral health than young adult civilians [9]. Improving the reach of behavioral health services to young adult veterans is consequently a policy priority.

Young adult veterans report multiple barriers to seeking and receiving behavioral health services in traditional settings and formats. These include inconvenience of appointments, concerns about high costs, perceived stigma from peers, beliefs that they can handle symptoms on their own, and living in rural settings that are far from care settings [3,4,10-14]. Expanding beyond traditional care settings and developing innovative means of engagement can help address unmet behavioral health needs.

Video games have the potential to improve the reach of behavioral health services [15], including those who have currently unmet behavioral health needs or face difficulty accessing treatment [16]. Given their increasing popularity [17], research on video game use is increasingly shifting from a focus on its potential negative impacts (eg, exposure to violence) to its potential cognitive, emotional, social, and health benefits [18,19]. Specifically, video games are increasingly used for health-related interventions, given their engaging and entertaining format [20,21] and their versatility across different platforms or environments such as consoles, computers, and mobile phone apps [15]. Recently, these apps have been extended to serve as an alternative or additional form of treatment for behavioral health [22,23]. For example, a computer video game that incorporates evidence-based cognitive behavioral therapy was found in a randomized trial to be both an appealing and efficacious treatment for adolescent depression [16,24].

In this exploratory study, we replicated analyses from two cross-sectional, community-based surveys to explore the plausibility of video games as a modality for behavioral health services for young adult US veterans. The lack of data on veteran video game playing precluded us from making clear a priori hypotheses regarding the prevalence of video game playing in the sample. As video game-based interventions appeal more to those who play video games generally [16], the lack of familiarity with and available leisure time to play video games can serve as key barriers to their use for behavioral health services [17]. We therefore used the time spent playing video games as a proxy for familiarity with and time available for video games. We specifically examined differences in time spent playing video games by whether participants screened positive for a behavioral health issue (ie, alcohol misuse, depression, and PTSD) and received the required behavioral health services.

## Methods

### Study Procedures

Data presented in this manuscript are from two surveys conducted as part of a larger randomized controlled trial (RCT) of a Web-based normative feedback intervention for heavy drinking young adult veterans [25,26]. We collected data on the video game behaviors of young adult veterans for the comparator intervention (ie, attention-matched normative feedback on video game behaviors); we did not prespecify any of the analyses on the video game behavior data reported in this manuscript. The Human Subjects Protection Committee at the RAND Corporation approved all procedures for both studies.

### Participant Recruitment and Eligibility

We recruited nontreatment seeking young adult (age, 18-34 years) veteran participants in both samples through advertisements on Facebook that did not mention video games or behavioral health. We have previously reported comprehensive details of the recruitment strategy and methods for validating veteran participants for Study 1 [27] and Study 2 [28]. We conducted all procedures online.

#### Study 1

Study 1 involved a survey on the behavioral health symptoms of a large general sample of young adult veterans recruited outside of VHA settings. We targeted a series of Facebook ads to users between the ages of 18 and 40 years who expressed an interest in (ie, “liked”) specific veteran or military Facebook pages as well as media (movies, TV shows, and video games) related to military (eg, Act of Valor, Generation Kill, Call of Duty). Interested Facebook users who clicked on ads were directed to a Web-based informational statement and consent form. Eligible participants who consented to participate were first verified to be actual veterans using data check procedures we have described in detail elsewhere [27], before completing a longer Web-based survey of the measures described below.

#### Study 2

Study 2 involved a screening and baseline survey for an RCT of a brief, Web-based, personalized normative drinking intervention, where participants saw feedback about their drinking (intervention) or video game playing behavior (control) compared with their peers. As with Study 1, participants clicked on targeted Facebook ads, although we did not include ads targeting any media regarding video games (eg, Call of Duty). The eligibility criteria were the same across studies, except that participants in Study 2 needed to score at least a 3 (females) or 4 (males) on the 10-item Alcohol Use Disorders Identification Test (AUDIT) [29]. These AUDIT cutoff scores were selected to include participants in the larger RCT who drank at moderate to high levels and were at risk for hazardous or problem drinking [30,31].

### Participant Characteristics

#### Study 1

We recruited 1023 young adult veterans overall, of whom 552 (53.9%) reported playing video games at least 1 hour per week. To match the eligibility criteria of Study 2, we restricted the subsample who reported playing video games at least 1 hour per week to the 350 veterans who also had scores of at least 3 (females) or 4 (males) on AUDIT.

#### Study 2

We recruited 784 young adult veterans overall. Because we were interested in veterans who reported any video game use for analyses in the current study, we restricted our sample to 582 veterans (74.2%, 582/784) who reported playing video games at least 1 hour per week.

### Materials

#### Data Collection

For both studies, we collected information on demographics, behavioral health symptoms, behavioral health services use, and video game behaviors. In this manuscript, we report analyses on similar constructs assessed in both Study 1 and Study 2, although we operationalized several constructs using different measures (Multimedia Appendices 1 and 2).

#### Demographics

Participants in both studies filled out the same measures regarding age, gender, ethnicity or race, education, marital status, number of children, annual household income, and branch of military service.

#### Behavioral Health Symptoms

Participants completed brief screening measures for behavioral health problems.

#### Posttraumatic Stress Disorder Symptoms

In Study 1, we assessed PTSD symptoms with the 4-item Primary Care PTSD scale (PC-PTSD). A score of 3 or higher (ie, participants endorsed “yes” for 3 of the 4 PTSD symptoms) on PC-PTSD indicated a probable diagnosis of PTSD [32]. In Study 2, we assessed PTSD symptoms in the past month with the 20-item PTSD Checklist for Diagnostic and Statistical Manual of Mental Disorders, Fifth Edition (PCL-5). Items in PCL-5 ranged from 0 “not at all” to 4 “extremely,” with a cutoff score of 33 or higher as screening for a probable diagnosis of PTSD [33].

#### Depression Symptoms

In Study 1, we assessed depression symptoms with the 2-item Patient Health Questionnaire (PHQ-2). Participants rated two symptoms (ie, “little interest or pleasure in doing things” and “feeling down, depressed, or hopeless”) from 0 “not at all” to 3 “nearly every day” for the past 2 weeks. A score of 2 on the PHQ-2 indicated screening for a depression diagnosis [34]. In Study 2, we assessed depressive symptoms for the past 2 weeks with the 8-item Patient Health Questionnaire (PHQ-8). Items on the PHQ-8 ranged from 0 “not at all” to 3 “nearly every day,” with a cutoff score of 10 or higher as screening for a probable diagnosis of a major depressive disorder [35].

#### Alcohol Use

In both studies, we assessed alcohol use disorder (AUD) symptoms in the past year using AUDIT [29]. A score of 8 or higher indicated hazardous drinking. Participants in both studies also reported for the past 30 days, the number of days the participants drank; the amount of alcohol consumed per occasion; heavy drinking days, that is, days when they consumed more than 4 drinks for females or more than 5 for males; and largest number consumed on any one occasion. We assessed consequences with the Brief Young Adult Alcohol Consequences Questionnaire [36], where participants indicated if they experienced each of the 21 consequences related to drinking in the past month.

#### Cannabis Use

We asked participants if they used any cannabis or marijuana in the past 6 months (yes or no), and if so, how many days in the past month did they use. The Study 1 survey referred to the drug as cannabis and the Study 2 survey referred to it as marijuana.

#### Behavioral Health Services Use

In both studies, participants indicated whether they had attended any appointments (in any setting: VHA, Vet Centers, or community providers) for mental health concerns or substance use concerns since discharge from active duty in the past month or year.

#### Video Game Behaviors

In both studies, participants indicated the typical number of hours they played video games per day, hours they played video games per week, and days they played video games per week in the past 30 days using slightly different methods. In Study 1, participants indicated how many hours on each day of the week they typically played video games, while in Study 2, they responded to 3 single items about the hours per day, hours per week, and days per week they typically played video games. In both studies, participants were asked to consider computer-based games, console video games, arcade video games, mobile phone or tablet games, or Web-based JavaScript games.

### Analysis Procedures

We first calculated descriptive statistics for, and differences in, demographics between Study 1 and Study 2 samples. Then, we used Welch’s *t* test to examine whether participants who screened positive for a behavioral health issues played video games to a different degree than those who did not screen positive. Lastly, among participants who screened positive for behavioral health issues, we used Welch’s *t* test to examine whether those who received services played video games to a different degree than those who did not report using any services. We examined the results from the analyses on behavioral health and video game behaviors in two ways [37,38]. First, we assessed whether the “existence” (statistical significance) and direction of any differences were replicated across both studies: (ie, *P*<.05 in same direction in both studies). Second, we calculated the fixed-effect meta-analytic estimate for the mean difference in effects by pooling differences from both studies [39].

## Results

### Demographics

We included 350 participants from Study 1 and 582 participants from Study 2 (see Table 1). The average ages of participants in Study 1 and Study 2 were 28 and 29 years, respectively. Study 1 had significantly more males (323/350, 92.6%) than Study 2 (505/582, 86.8%), χ^2^_1_=6.8, *P*=.009 (N=931). Study 1 had significantly more Hispanic participants (72/349, 20.6%) than Study 2 (60/582, 10.3%), χ^2^_1_=19.1, *P*<.001 (N=931). Study 2 had more White participants (499/582, 85.7%) than Study 1 (273/350, 78.0%), χ^2^_1_=8.7, *P*=.003 (N=932). A substantial majority of participants in both Study 1 (64/350, 18.3%) and Study 2 (116/582, 19.9%) had not earned a college degree, although more participants in Study 1 (163/350, 47%) were currently in college than in Study 2 (200/582, 34.4%), χ^2^_1_=13.2, *P*<.001 (N=932). In both studies, the modal annual household income was US $25,000 to US $49,999; about half of the participants in each study were married, with an average of one child per veteran. Among those with children, the average number of children living at home was 2. The majority of participants in both studies served previously in the army, with about a quarter previously serving in the marines.

**Table 1:** Participant characteristics.

Demographics		Study 1 (N=350)	Study 2 (N=582)	*P* value^a^
Age, mean (SD)		28.4 (3.4)	28.7 (3.4)	.11
Male, n (%)		323 (92.6)	505 (86.8)	.009
Hispanic ethnicity, n (%)		72 (20.6)	60 (10.3)	<.001
**Race, n (%)**				**.003**
	White	273 (78.0)	499 (85.7)	
	Other	77 (22.0)	83 (14.3)	
**Education, n (%)**				**.60**
	Some college or less	286 (81.7)	437 (80.1)	
	College graduate	64 (18.3)	116 (19.9)	
**Currently in college, n (%)**				**<.001**
	No	187 (53.4)	382 (65.6)	
	Yes	163 (46.6)	200 (34.4)	
**Annual household income, n (%)**				**.29**
	<US $10,000	29 (8.3)	32 (5.5)	
	US $10,000 to US $14,999	29 (8.3)	46 (7.9)	
	US $15,000 to US $24,999	70 (20.0)	90 (15.5)	
	US $25,000 to US $49,999	121 (34.6)	238 (40.9)	
	US $50,000 to US $99,999	84 (24.0)	142 (24.4)	
	US $100,000 to US $149,999	14 (4.0)	28 (4.8)	
	US $150,000 to US $199,999	3 (0.9)	3 (0.9)	
	US $200,000 +	0 (0.0)	1 (0.2)	
Married, n (%)		186 (53.1)	278 (47.8)	.13
Number of children, mean (SD)		1.4 (1.5)	1.3 (1.4)	.14
Number of children living at home, mean (SD)		1.7 (1.1)	1.7 (1.3)	.82
**Branch of service, n (%)**				**.25**
	Air Force	22 (6.3)	57 (9.8)	
	Army	215 (61.4)	348 (59.8)	
	Marine Corps	87 (24.9)	129 (22.2)	
	Navy	26 (7.4)	48 (8.2)	
**Mental health, n (%)**				
	Positive screen for posttraumatic stress disorder	152 (43.4)	227 (39.0)	.21
	Positive screen for depression	169 (48.3)	271 (46.6)	.66
**Alcohol use**				
	Positive screen for disorder, n (%)	151 (43.1)	174 (29.9)	<.001
	Total drinking days, mean (SD)	10.4 (9.1)	12.4 (8.8)	.001
	Drinks per drinking day, mean (SD)	4.8 (4.3)	4.7 (3.3)	.77
	Heavy drinking occasions, mean (SD)	4.6 (6.2)	5.8 (7.2)	.01
	Max drinks on a drinking day, mean (SD)	8.5 (6.0)	9.4 (6.0)	.03
	Alcohol consequences, mean (SD)	7.8 (7.1)	7.6 (6.8)	.58
**Cannabis use**										
	Cannabis use in past 6 months, n (%)	106 (41.9)	169 (29.0)	<.001
	Total number of cannabis use days, mean (SD)	9.9 (11.4)	3.3 (8.6)	<.001
**Any behavioral health services receipt, n (%)**				
	Services since discharge	183 (52.3)	338 (58.1)	.10
	Services in past year	119 (34.0)	238 (40.9)	.04
	Services in past month	42 (12.0)	101 (17.4)	.04
**Mental health services receipt, n (%)**				
	Services since discharge	175 (50.0)	320 (55.0)	.16
	Services in past year	116 (33.1)	228 (39.2)	.08
	Services in past month	42 (12.0)	98 (16.8)	.06
**Substance use services receipt, n (%)**				
	Services since discharge	67 (19.1)	121 (20.8)	.60
	Services in past year	31 (8.9)	66 (11.3)	.28
	Services in past month	1 (0.3)	18 (3.1)	.007
**Video game use, mean (SD)**				
	Total hours spent playing per day	2.3 (1.8)	3.5 (3.2)	<.001
	Total hours spent playing per week	12.8 (13.5)	18.4 (21.9)	<.001
	Total days spent playing per week	5.0 (2.3)	4.7 (2.2)	.046

^a^*P* values are reported from the Welch’s two-sample *t* test for continuous measures and chi-square test for categorical measures.

### Behavioral Health

Similar proportions of patients screened positive for PTSD (Study 1, 152/350, 43.4%; Study 2, 227/582, 39.0%) and depressive disorder (Study 1, 169/350, 48.3%; Study 2, 271/582, 46.6%). Participants consumed about 5 drinks per drinking day. Screening positive for an AUD was more likely for participants in Study 1 (151/350, 43.1%) than in Study 2 (174/582, 29.9%), χ^2^_1_=16.3, *P*<.001 (N=932). However, compared with participants in Study 1, participants in Study 2 drank more days in the past month (Study 1 mean 10.4 [SD 9.1]; Study 2 mean 12.4 [SD 8.8]; *t*_715_=−3.2; *P*=.001), drank more drinks on their peak drinking day (Study 1 mean 8.5 [SD 6.0]; Study 2 mean 9.4 [SD 6.0]; *t*_662_=−2.2; *P*=.03) and had more heavy drinking days (Study 1 mean 4.6 [SD 6.2]; Study 2 mean 5.8 [SD 7.2]; *t*_743_=−2.5; *P*=.01). Compared with participants in Study 2, more participants in Study 1 used cannabis in the past 6 months (Study 1, 106/253, 41.9%; Study 2, 169/582, 29.0%), χ^2^_1_=12.6, *P*<.001 (N=835), and on more days in the past month (Study 1 mean 9.9 [SD 11.4]; Study 2 mean 3.3 [SD 8.6]), *t*_100_=5.01, *P*<.001. Approximately half of the participants reported any use of behavioral health services since their discharge, a little over a third reported use within the past year, and less than one-fifth reported use within the past month.

### Video Game Behaviors

Participants in Study 1 reported playing video games fewer hours per day (Study 1 mean 2.3 [SD 1.8]; Study 2 mean 3.5 [SD 3.2]; *t*_925_=−7.4; *P*<.001) and per week (Study 1 mean 12.8 [SD 13.5]; Study 2 mean 18.4 [SD 21.9]; *t*_929_=−4.8; *P*<.001) than participants in Study 2. However, participants in Study 1 spent more days per week playing video games than participants in Study 2 (Study 1 mean 5.0 [SD 2.3]; Study 2 mean 4.7 [SD 2.2]; *t*_717_=2.0; *P*=.046).

### Video Game Behaviors by Positive Screen

Within each study, participants with any positive screen (PTSD, depression, AUD, or cannabis use) did not differ significantly from participants without any positive screen on video game behavior, while participants with either positive mental health screen (PTSD, depression) spent more hours per day and per week playing video games than those without a positive mental health screen (Multimedia Appendix 3). Pooling data across studies, participants with any positive screen for a mental health or substance use issue spent 2.61 more hours per week (95% CI 0.11-5.10) playing video games than participants without any positive screen. Participants with any positive mental health screen (PTSD, depression) spent 0.61 more hours per day (95% CI 0.30-0.92), 4.74 more hours per week (95% CI 2.54-6.94), and 0.41 more days per week (95% CI 0.13-0.70) playing video games.

### Video Game Use by Services Receipt Among Participants With a Positive Screen

No association was found between video game use and either any services receipt, mental health services receipt, or substance use services receipt within both studies. Pooling data across studies, participants with any positive screen for a behavioral health issue who had received any type of behavioral health services (mental health services, substance use services) since discharge from active duty spent 2.67 more hours per week (95% CI 0.14-5.20) and 0.48 more days per week (95% CI 0.14-0.82) playing video games than participants with any positive screen who had not received any type of behavioral health services since discharge. Participants with any positive SUD screen who had received any type of substance use services since discharge spent 0.75 more days per week (95% CI 0.28-1.21) playing video games than participants with any positive SUD screen who had not received any type of substance use services since discharge.

## Discussion

### Principal Findings

We examined the video game playing behavior of two separate samples of young adult veterans recruited online. First, we found evidence across the two samples that most young veterans played video games: 54% of a general sample of young veterans and 74% of a sample of young adult veteran drinkers reported playing video games at least 1 hour per week. Next, among the video game players, we found that young adult veterans spent about 13-18 hours per week playing video games and about 2.5-3.5 hours per day playing video games. In a typical week, young adult veterans played video games on most days of the week. These findings suggest that video games might be a feasible intervention modality for young veterans generally and for behavioral health concerns specifically.

While most analyses did not yield differences that were replicated across both studies, we did find several replicated differences in video game behaviors among young adult veterans depending on their screening positive for a behavioral health issue as well as their receiving services for a behavioral health need. Regarding screening positive for a behavioral health issue, we identified the strongest evidence for more hours per day and per week playing video games among participants with a positive screen for both PTSD and depression compared with those without positive screens for these conditions. Regarding the receipt of services for a behavioral health need, we identified the strongest evidence that participants who had a positive SUD screen and received substance use services since discharge spent more days per week playing video games than those with a positive SUD screen who had not received substance use services.

Although most veterans in our sample played video games and those that did played quite often, our findings aimed to identify the potential groups of young adult veterans for the development and evaluation of video games as a modality for behavioral health services. Specifically, with respect to playing video games more per day and per week, young adult veterans screening positive for PTSD and depression may be more familiar with and dedicate more time to behavioral health services delivered via video games because these veterans already play more video games and more frequently than those without these issues and those not receiving services. For this population, relatively more intensive video game-based interventions might be acceptable. For example, previous research has demonstrated the feasibility of incorporating the core components of traditional cognitive behavioral therapies and exercise-based interventions for mental health into engaging, video games in Web-based, computer, console, and application-based formats [15-17,20,23,40,41]. Similarly, young adult veterans who have previously sought care but are currently not receiving services for SUDs are a more promising population than those with SUDs who have never sought care since their discharge from active duty. Because these veterans were open to receiving services in the past, video game-based interventions for this population could focus on delivering motivational interviewing techniques that encourage them to enter a new treatment episode [17,42] or be used to supplement care received after initiation to encourage retention and compliance with treatment goals (eg, completing cognitive behavioral therapy “homework” via video games).

### Strengths and Limitations

Several strengths and limitations are worth noting. Strengths of this study include replicating analyses from two independent samples to reduce the rate of false positives [37,38]; sharing the data, code, and materials to facilitate computational reproducibility and verification (Multimedia Appendices 4 and 5) [43]; and signaling that these findings are exploratory [44] as they were not included in the study preregistrations [25]. In addition, using social media, we efficiently recruited hundreds of young adult veterans who currently play video games and screened positive for a behavioral health issue. We did this through Facebook advertisements that did not advertise the study to video game players exclusively. We did not mention in the ads that the study was looking for video game players, heavy drinkers, those with substance use or mental health problems, or those looking for treatment. Limitations include the exploratory nature of these analyses [45], recruiting our sample from Facebook, which limited the generalizability of our findings (though 10% learned about the study from family or friends and not directly from Facebook) [27] and the use of only self-report measures (ie, not diagnostic interviews) collected via the internet [25].

### Future Work

Findings suggest several avenues of future research as well as collaborations between researchers and video game developers. First, analyses from this study would benefit from direct, preregistered replications to strengthen the credibility of our findings. Direct assessment of acceptability and willingness to engage with video games for behavioral health services should be incorporated into this research [16]. If our results are replicated, an empirically testable theoretical framework should be prospectively developed to provide more useful understandings of the relationships among video game use, behavioral health, and intervention than those provided by our entirely exploratory empirical analyses. Second, research is needed on the optimal types of video games (eg, role-playing, adventure, fantasy) for different young adult veteran populations [20]. Third, future research should investigate which types of behavioral health services can be best integrated into video games for different young adult veteran populations [17], with particular attention to young adult veterans facing health concerns not investigated in this manuscript yet likely in this population, such as physical pain and traumatic brain injury. Importantly, future research is needed to confirm that the number of hours spent playing traditional video games can be converted to engagement with games that address their behavioral health issues. Lastly, as video games are developed and implemented, rigorous evaluations are needed to assess the effects for specific behavioral health issues [22].

## Appendix

Multimedia Appendix 1 Codebook for Grant, Spears, and Pedersen (Study 1 and Study 2).

Multimedia Appendix 2 Comparison of measures in Study 1 and Study 2.

Multimedia Appendix 3 Exploratory replication analyses of video game use by young adult veterans.

Multimedia Appendix 4 Markdown analytic code and output for exploratory analyses.

Multimedia Appendix 5 De-identified dataset.

## Abbreviations

AUD: alcohol use disorder
AUDIT: Alcohol Use Disorders Identification Test
PTSD: posttraumatic stress disorder
RCT: randomized controlled trial
SUD: substance use disorder
VHA: Veterans Health Administration
