# Supplementary material for: Video Games as a Potential Modality for Behavioral Health Services for Young Adult Veterans: Exploratory Analysis
Source: JMIR Serious Games. 2018 Jul 26;6(3):e15. doi: 10.2196/games.9327 (PMC6085553; doi:10.2196/games.9327)
Supplement: Multimedia Appendix 1 [file games_v6i3e15_app1.pdf]

Multimedia Appendix 1. Codebook for Grant, Spears, and Pedersen (Study 1 and Study 2)

Study
1: Study 1
2: Study 2

Age
Value between 19 and 34

Study 1 and Study 2: What is your age? *(response between 18 and 34*)

Gender
1: male
0: female

Study 1 and Study 2: What is your gender? (*male*, *female*)

Hispanic
1: Hispanic
0: non-Hispanic

Study 1 and Study 2: Are you Hispanic/Latino(a)? (*no, yes*)

Race
1: White
2: Black or African American
3: Asian
4: Native Hawaiian or Other Pacific Islander
5: American Indian or Alaska Native
6: More than one race/ethnicity
7: Other

Study 1 and Study 2: What is your race/ethnicity? (*White, Black or African American, Asian, Native Hawaiian or Other Pacific Islander, American Indian or Alaska Native, More than one race/ethnicity, Other*)

Racew
1: White
0: non-White

Study 1 and Study 2: recoded from **Race**

Education
1: Never attended school or only attended kindergarten
2: Grades 1 through 8 (Elementary)
3: Grades 9 through 11 (Some high school)

4: Grade 12 or GED (High school graduate)
5: College: 1 year to 3 years (Some college or technical school)
6: College: 4 years or more (College graduate)

Study 1 and Study 2: What is your highest level of education? (*Never attended school or only attended kindergarten, Grades 1 through 8 (Elementary), Grades 9 through 11 (Some high school), Grade 12 or GED (High school graduate), College: 1 year to 3 years (Some college or technical school), College: 4 years or more (College graduate))*

Educationc
0: Some college or less
1: College graduate

Study 1 and Study 2: recoded from **Education**

College
0: No
1: Yes, I attend a community college
2: Yes, I attend a technical college
3: Yes, I attend a state university
4: Yes, I attend a private college or university

Study 1 and Study 2: Are you currently in college? (*No; Yes, I attend a community college; Yes, I attend a technical college; Yes, I attend a state university; Yes, I attend a private college or university*)

Collegeyes
0: not currently in college
1: currently in college

Study 1 and Study 2: recoded from **College**

Income
1: Less than $10,000
2: $10,000 to $14,999
3: $15,000 to $24,999
4: $25,000 to $49,999
5: $50,000 to $99,999
6: $100,000 to $149,999
7: $150,000 to $199,999
8: $200,000 or more

Study 1 and Study 2: What is your annual household income from all sources? (Less than $10,000, $10,000 to $14,999, $15,000 to $24,999, $25,000 to $49,999, $50,000 to $99,999, $100,000 to $149,999, $150,000 to $199,999, $200,000 or more)

Married
0: no
1: yes

Study 1 and Study 2: What is your current marital status? (*Married, Engaged, Separated, Divorced, Widowed, Living together with a partner but not married or engaged, Never married, Other*)
Recoded as married vs all other categories (not married)

Children
Value between 0 and 10

Study 1 and 2: How many children (including step-children) do you have? (*response from 0 to 10)*

Childrenathome
Value between 0 and 10

Study 1 an Study 2: How many of these children live in your home with you? (*response from 0-10)*
Coded as 0 if response to **Children** = 0

Branch
1: Air Force
2: Army
3: Marines
4: Navy

Study 1 and Study 2: What is your former branch of service? (*Air Force, Army, Marines, Navy, Coast Guard (excluded if endorsed), I am not a U.S. veteran (excluded if endorsed)*)

Ptsd
0: negative screen for PTSD
1: positive screen for PTSD

Study 1: sum of 4 PTSD items on PC-PTSD scale $\geq$ 3.

In your life, have you ever had any experience that was so frightening, horrible, or upsetting that, in the past month, you:<br><br>Have had nightmares about it or thought about it when you did not want to?<br>0. No<br>1. Yes

Tried hard not to think about it or went out of your way to avoid situations that reminded you of it?
0. No
1. Yes

Were constantly of guard, watchful, or easily startled?
0. No
1. Yes

Felt numb or detached from others, activities, or your surroundings?
0. No
1. Yes

Study 2: sum of 20 PCL-5 items ≥ 33

In the past month, how much were you bothered by:	*Not at all*	*A little bit*	*Moderately*	*Quite a bit*	*Extremely*
1. Repeated, disturbing, and unwanted memories of the stressful experience?	0	1	2	3	4
1. Repeated, disturbing dreams of the stressful experience?	0	1	2	3	4
2. Suddenly feeling or acting as if the stressful experience were actually happening again (as if you were actually back there reliving it)?	0	1	2	3	4
3. Feeling very upset when something reminded you of the stressful experience?	0	1	2	3	4
4. Having strong physical reactions when something reminded you of the stressful experience (for example, heart pounding, trouble breathing, sweating)?	0	1	2	3	4
5. Avoiding memories, thoughts, or feelings related to the stressful experience?	0	1	2	3	4
6. Avoiding external reminders of the stressful experience (for example, people, places, conversations, activities, objects, or situations)?	0	1	2	3	4
7. Trouble remembering important parts of the stressful experience?	0	1	2	3	4

8. Having strong negative beliefs about yourself, other people, or the world (for example, having thoughts such as: I am bad, there is something seriously wrong with me, no one can be trusted, the world is completely dangerous)?	0	1	2	3	4
9. Blaming yourself or someone else for the stressful experience or what happened after it?	0	1	2	3	4
10. Having strong negative feelings such as fear, horror, anger, guilt, or shame?	0	1	2	3	4
11. Loss of interest in activities that you used to enjoy?	0	1	2	3	4
12. Feeling distant or cut off from other people?	0	1	2	3	4
13. Trouble experiencing positive feelings *(for example, being unable to feel happiness or have loving feelings for people close to you)*?	0	1	2	3	4
14. Irritable behavior, angry outbursts, or acting aggressively?	0	1	2	3	4
15. Taking too many risks or doing things that could cause you harm?	0	1	2	3	4
16. Being "superalert" or watchful or on guard?	0	1	2	3	4
17. Feeling jumpy or easily startled?	0	1	2	3	4
18. Having difficulty concentrating?	0	1	2	3	4
19. Trouble falling or staying asleep?	0	1	2	3	4

Depression
0: negative screen for depression
1: positive screen for depression

Study 1: sum of 2 PHQ-2 items $\geq$ 2.

Over the last 2 weeks, how often have you been bothered by the following problems?

	Not at all	Several days	More than half the days	Nearly every day
Little interest or pleasure in doing things	0	1	2	3

Feeling down, depressed, or hopeless	0	1	2	3

Study 2: sum of 8 PHQ-8 items > 10.

Over the last 2 weeks, how often have you been bothered by any of the following:

	Not at all	Several days	More than half the days	Nearly every day
Little interest or pleasure in doing things?	0	1	2	3
Feeling down, depressed, or hopeless?	0	1	2	3
Trouble falling or staying asleep, or sleeping too much?	0	1	2	3
Feeling tired or having little energy?	0	1	2	3
Poor appetite or overeating?	0	1	2	3
Feeling bad about yourself, or that you are a failure, or have let yourself or your family down?	0	1	2	3
Trouble concentrating on things, such as reading the newspaper or watching television?	0	1	2	3
Moving or speaking so slowly that other people could have noticed. Or the opposite – being so fidgety or restless that you have been moving around a lot more than usual?	0	1	2	3

<u>Audit</u>

0: negative screen for an alcohol use disorder
1: positive screen for an alcohol use disorder

Study 1 and Study 2: sum of 10 AUDIT items > 8.

Consider the past year.

How often do you have a drink containing alcohol?				
(0) Never	(1)Monthly or less	(2) 2 to 4 times a month	(3) 2 to 3 times a week	(4) 4 or more times a week
How many drinks containing alcohol do you have on a typical day when you are drinking?				
(0) 1 or 2	(1) 3 or 4	(2) 5 or 6	(3) 7, 8, or 9	(4) 10 or more

How often do you have six or more drinks on one occasion?				
(0) Never	(1) Less than monthly	(2) Monthly	(3) Weekly	(4) Daily or almost daily
How often during the last year have you found that you were not able to stop drinking once you had started?				
(0) Never	(1) Less than monthly	(2) Monthly	(3) Weekly	(4) Daily or almost daily
How often during the last year have you failed to do what was normally expected of you because of drinking?				
(0) Never	(1) Less than monthly	(2) Monthly	(3) Weekly	(4) Daily or almost daily
How often during the last year have you needed a first drink in the morning to get yourself going after a heavy drinking session?				
(0) Never	(1) Less than monthly	(2) Monthly	(3) Weekly	(4) Daily or almost daily
How often during the last year have you had a feeling of guilt or remorse after drinking?				
(0) Never	(1) Less than monthly	(2) Monthly	(3) Weekly	(4) Daily or almost daily
How often during the last year have you been unable to remember what happened the night before because you had been drinking?				
(0) Never	(1) Less than monthly	(2) Monthly	(3) Weekly	(4) Daily or almost daily
Have you or has someone else been injured as a result of your drinking?				
(0) No	(2) Yes, but not in the last year		(4) Yes, during the last year	

Has a relative or friend or a doctor or another health worker been concerned about your drinking or suggested you cut down?		
(0) No	(2) Yes, but not in the last year	(4) Yes, during the last year

Anyscreen
0: all three **Ptsd**, **Depression**, and **Audit** = 0
1: any 1 value for **Ptsd**, **Depression**, or **Audit**

Anymhscreen
0: both **Ptsd** and **Depression** = 0
1: any 1 value for **Ptsd** or **Depression**

Anysudscreen
0: **Audit** = 0
1: **Audit** = 1

Totdrinkdays
Value from 0 to 30

Study 1 and Study 2: During the past 30 days, how many days did you have at least one drink of any alcoholic beverage such as beer, wine, a malt beverage, or liquor? (*response from 0 to 30 days)*

Avgdrinksday
Value from 0 to 30

Study 1 and Study 2: During the past 30 days, on the days when you drank, about how many drinks did you drink on average? *(response from 0 to 30 drinks)*
Participants responding 0 to totdrinkdays item received a 0 for avgdrinksday

Heavydrink
Value from 0 to 30

Study 1 and Study 2: Considering all types of alcoholic beverages, how many times during the past 30 days did you have [5 for men, 4 for women] or more drinks on an occasion? *(response from 0 to 30 times)*

Peakdrink
Value from 0 to 30

Study 1 and Study 2: During the past 30 days, what is the largest number of drinks you had on any one occasion? *(response from 0 to 30 drinks)*

BYAACQ
Value from 0 to 21

Study 1 and Study 2: sum of 21 BYAACQ items

Consider the past 30 days

	0 = No	1= Yes
While drinking, I have said or done embarrassing things.	0	1
I have had a hangover (headache, sick stomach) the morning after I had been drinking.	0	1
I have felt very sick to my stomach or thrown up after drinking.	0	1
I often have ended up drinking on nights when I had planned not to drink.	0	1
I have taken foolish risks when I have been drinking.	0	1
I have passed out from drinking.	0	1
I have found that I needed larger amounts of alcohol to feel any effect, or that I could no longer get high or drunk on the amount that used to get me high or drunk.	0	1
When drinking, I have done impulsive things that I regretted later.	0	1
I've not been able to remember large stretches of time while drinking heavily.	0	1
I have driven a car when I knew I had too much to drink to drive safely.	0	1
I have not gone to work or missed classes at school because of drinking, a hangover, or illness caused by drinking.	0	1
My drinking has gotten me into sexual situations I later regretted.	0	1
I have often found it difficult to limit how much I drink.	0	1
I have become very rude, obnoxious or insulting after drinking.	0	1
I have woken up in an unexpected place after heavy drinking.	0	1
I have felt badly about myself because of my drinking.	0	1
I have had less energy or felt tired because of my drinking.	0	1
The quality of my work or schoolwork has suffered because of my drinking.	0	1
I have spent too much time drinking.	0	1
I have neglected my obligations to family, work, or school because of drinking.	0	1
My drinking has created problems between myself and my boyfriend/girlfriend/spouse, parents, or other near relatives.	0	1
I have been overweight because of drinking.	0	1
My physical appearance has been harmed by my drinking.	0	1
I have felt like I needed a drink after I'd gotten up (that is, before breakfast).	0	1

Anycannabis
0: no cannabis use past 6 months

1: any cannabis use past 6 months

Study 1: Have you used any cannabis (marijuana, pot, hash, hashish) over the past six months? *(No, Yes)*

Study 2: In the past six months, how often have you used marijuana (pot, hash, hashish)? (*Never, Once or twice in the past six months, Two to five times in the past six months, About once per month, About two to four times per month, About once per week, A few times per week to daily*)

<u>Totcannabis</u>
Value from 0 to 30

Study 1: During the past 30 days, how many days did you use cannabis? *(response from 0 to 30 days)*

Study 2: During the past 30 days, how many days did you use marijuana? *(response from 0 to 30 days)*

<u>Mhdis</u>
0: no mental health care appointments since discharge
1: at least one mental health care appointment since discharge

Study 1: any Yes response to the following three items:

Since you have discharged from the military, have you ever attended an appointment at a VA (Veterans Health Administration) for any of the following? Consider appointments at VA hospitals or VA community-based outpatient clinics, but do not include appointments at a Vet Center.		
Mental health concern (for example help with stress, anxiety, depression, nightmares)	YES (1)	NO (0)

Since you have discharged from the military, have you ever attended an appointment at a Vet Center for any of the following? Consider Vet Centers as separate from VA hospitals or VA community-based outpatient clinics.		
Mental health concern (for example help with stress, anxiety, depression, nightmares)	YES (1)	NO (0)

Since you have discharged from the military, have you ever attended an appointment at a non-VA or non-Vet Center clinic, hospital, or doctor's office for any of the following?		
Mental health concern (for example help with stress, anxiety, depression, nightmares)	YES (1)	NO (0)

Study 2: any Yes response to the following two items

Since you have discharged from the military, have you ever attended an appointment at a VA (Veterans Health Administration) for any of the following? Consider appointments at VA hospitals or VA

community-based outpatient clinics, but do not include appointments at a Vet Center.		
Mental health concern (for example, help with stress, anxiety, depression, nightmares)	YES (1)	NO (0)

Since you have discharged from the military, have you ever attended an appointment at a non-VA clinic, hospital, or doctor's office, or at a Vet Center for any of the following for any of the following?		
Mental health concern (for example, help with stress, anxiety, depression, nightmares)	YES (1)	NO (0)

<u>Mhyear</u>
0: no mental health care appointments in past year
1: at least one mental health care appointment in past year

Study 1: Response options of between 6 months and one year ago, between 3 and 6 months ago, and between one and 3 months ago to the following item:

When was the last time you attended an appointments at the VA or the Vet Center or a non-VA clinic, hospital, or doctor's office with a provider to help you with a mental health concern? (*Over a year ago, Between 6 months and one year ago, Between 3 and 6 months ago, Between one and 3 months ago, Within the past month*)

Study 2: Response options of between 6 months and one year ago, between 3 and 6 months ago, and between one and 3 months ago to the following item:

When was the last time you attended an appointments at the VA or a non-VA clinic, hospital, or doctor's office with a provider to help you with a mental health concern? (*Over a year ago, Between 6 months and one year ago, Between 3 and 6 months ago, Between one and 3 months ago, Within the past month*)

<u>Mhmonth</u>
0: no mental health care appointments in past month
1: at least one mental health care appointment in past month

Study 1: Response option of Within the past month to the following item:

When was the last time you attended an appointments at the VA or the Vet Center or a non-VA clinic, hospital, or doctor's office with a provider to help you with a mental health concern? (*Over a year ago, Between 6 months and one year ago, Between 3 and 6 months ago, Between one and 3 months ago, Within the past month*)

Study 2: Response option of Within the past month to the following item:

When was the last time you attended an appointments at the VA or a non-VA clinic, hospital, or doctor's office with a provider to help you with a mental health concern? (*Over a year ago, Between 6 months and one year ago, Between 3 and 6 months ago, Between one and 3 months ago, Within the past month*)

Alcsuddis
0: no alcohol or substance use care appointments since discharge
1: at least one alcohol or substance use care appointment since discharge

Study 1: any Yes response to the following three items

Since you have discharged from the military, have you ever attended an appointment at a VA (Veterans Health Administration) for any of the following? Consider appointments at VA hospitals or VA community-based outpatient clinics, but do not include appointments at a Vet Center.		
Alcohol use concern (for example help with drinking less, attending groups to stay abstinent from alcohol)	YES (1)	NO (0)
Other substance use concern (for example help reducing cigarette use, attending groups to stay abstinent marijuana or other drugs)	YES (1)	NO (0)

Since you have discharged from the military, have you ever attended an appointment at a Vet Center for any of the following? Consider Vet Centers as separate from VA hospitals or VA community-based outpatient clinics.		
Alcohol use concern (for example help with drinking less, attending groups to stay abstinent from alcohol)	YES (1)	NO (0)
Other substance use concern (for example help reducing cigarette use, attending groups to stay abstinent from marijuana or other drugs)	YES (1)	NO (0)

Since you have discharged from the military, have you ever attended an appointment at a non-VA or non-Vet Center clinic, hospital, or doctor's office for any of the following?		
Alcohol use concern (for example help with drinking less, attending groups to stay abstinent from alcohol)	YES (1)	NO (0)
Other substance use concern (for example help reducing cigarette use, attending groups to stay abstinent from marijuana or other drugs)	YES (1)	NO (0)

Study 2: any Yes response to the following two items

Since you have discharged from the military, have you ever attended an appointment at a VA (Veterans Health Administration) for any of the following? Consider appointments at VA hospitals or VA community-based outpatient clinics, but do not include appointments at a Vet Center.		
Alcohol use concern (for example, help with drinking less, attending groups to stay abstinent from alcohol)	YES (1)	NO (0)
Other substance use concern (for example, help reducing cigarette use, attending groups to stay abstinent from marijuana or other drugs)	YES (1)	NO (0)

Since you have discharged from the military, have you ever attended an appointment at a non-VA

clinic, hospital, or doctor's office, or at a Vet Center for any of the following for any of the following?		
Alcohol use concern (for example, help with drinking less, attending groups to stay abstinent from alcohol)	YES (1)	NO (0)
Other substance use concern (for example, help reducing cigarette use, attending groups to stay abstinent from marijuana or other drugs)	YES (1)	NO (0)

Alcsudyear
0: no alcohol or substance use care appointments in past year
1: at least one alcohol or substance use care appointment in past year

Study 1: Response options of between 6 months and one year ago, between 3 and 6 months ago, and between one and 3 months ago to the following item:

When was the last time you attended an appointments at the VA or the Vet Center or a non-VA clinic, hospital, or doctor's office with a provider to help you with an alcohol use concern or a substance use concern? (*Over a year ago, Between 6 months and one year ago, Between 3 and 6 months ago, Between one and 3 months ago, Within the past month*)

Study 2: Response options of between 6 months and one year ago, between 3 and 6 months ago, and between one and 3 months ago to the following item:

When was the last time you attended an appointments at the VA or a non-VA clinic, hospital, or doctor's office with a provider to help you with an alcohol use concern or a substance use concern? (*Over a year ago, Between 6 months and one year ago, Between 3 and 6 months ago, Between one and 3 months ago, Within the past month*)

Alcsudmonth
0: no alcohol or substance use care appointments in past month
1: at least one alcohol or substance use care appointment in past month

Study 1: Response option of Within the past month to the following item:

When was the last time you attended an appointments at the VA or the Vet Center or a non-VA clinic, hospital, or doctor's office with a provider to help you with an alcohol use concern or a substance use concern? (*Over a year ago, Between 6 months and one year ago, Between 3 and 6 months ago, Between one and 3 months ago, Within the past month*)

Study 2: Response option of Within the past month to the following item:

When was the last time you attended an appointments at the VA or a non-VA clinic, hospital, or doctor's office with a provider to help you with an alcohol use concern or a substance use concern? (*Over a year ago, Between 6 months and one year ago, Between 3 and 6 months ago, Between one and 3 months ago, Within the past month*)

Anydis

0: **Mhdis** and **Alcsuddis** both = 0
1: Either **Mhdis** or **Alcsuddis** = 1

<u>Anyyear</u>
0: **Mhyear** and **Alcsudyear** both = 0
1: Either **Mhyear** or **Alcsudyear** = 1

<u>Anymonth</u>
0: **Mhmonth** and **Alcsudmonth** both = 0
1: Either **Mhmonth** or **Alcsudmonth** = 1

<u>Vghrsday</u>
Value from 0 to 24

Instructions for video games items in Study 1 and Study 2: The following items ask you about your behavior and attitudes about video game playing. Consider video games to be PC-based games (for example, World of Warcraft), console video games (for example games you play on PlayStation 3 or Xbox 360; or portable video game consoles like Nintendo DS), video games you play in an arcade (for example, Big Buck Hunter), games you play on your phone or tablet (for example, Angry Birds, Words with Friends), or computer/online java-script games (for example, computer solitaire or hearts, games played via Facebook, games at www.popcap.com).

Study 1: average of responses to Monday through Sunday items on days where hours > 0

Consider a typical week during the past month (30 days). How many hours, on average,, did you spend playing video games on each day of <u>a typical week</u>?	Hours
Monday	
Tuesday	
Wednesday	
Thursday	
Friday	
Saturday	
Sunday	

Study 2: Consider the past month (30 days). How many hours, on average, did you spend on a typical day playing video games? *(response from 0 to 24 hours)*

<u>Vghrsweek</u>
Value from 0 to 168

Study 1: sum of responses to Monday through Sunday items

Consider a typical week during the past month (30 days). How many hours, on average, did you spend playing video games on each day of a typical week?	Hours
Monday	
Tuesday	
Wednesday	
Thursday	
Friday	
Saturday	
Sunday	

Study 2: Responses to two items multiplied (days x hours)
Consider the past month (30 days). How many days did you play video games in a typical week? *(response from 0 to 30 days)*
Consider the past month (30 days). How many hours, on average, did you spend on a typical day playing video games? *(response from 0 to 24 hours)*

Vgdaysweek
Value from 0 to 7

Study 1: count of days Monday through Sunday with response > 0

Consider a typical week during the past month (30 days). How many hours, on average, did you spend playing video games on each day of a typical week?	Hours
Monday	
Tuesday	
Wednesday	
Thursday	
Friday	
Saturday	
Sunday	

Study 2: Consider the past month (30 days). How many days did you play video games in a typical week? *(response from 0 to 30 days)*
