# Supplementary material for: Video Games as a Potential Modality for Behavioral Health Services for Young Adult Veterans: Exploratory Analysis
Source: JMIR Serious Games. 2018 Jul 26;6(3):e15. doi: 10.2196/games.9327 (PMC6085553; doi:10.2196/games.9327)
Supplement: Multimedia Appendix 2 [file games_v6i3e15_app2.pdf]

Multimedia Appendix 2. Comparison of measures in Study 1 and Study 2

Measure	Study 1	Study 2
Demographics		
Age	What is your age?	
Gender	What is your gender?	
Hispanic/Latino(a)	Are you Hispanic/Latino(a)?	
Ethnicity	What is your ethnicity?	What is your race/ethnicity?
Education	What is your highest level of education?	
Currently in college	Are you currently in college?	
Annual household income	What is your annual household income from all sources?	
Marital status	What is your current marital status?	
Number of children	How many children (including step-children) do you have?	
Number of children living at home	How many of these children live in your home with you?	
Branch of service	What is your former branch of service?	
Mental health		
Positive screen for PTSD	Primary Care Posttraumatic Stress Disorder scale	PTSD Checklist
Positive screen for depressive disorder	2-item Patient Health Questionnaire	8-item Patient Health Questionnaire
Substance use		
Positive screen for alcohol use disorder	Alcohol Use Disorders Identification Test	
Total drinking days	Days in past 30 days in which participant had at least one drink of any alcoholic beverage	
Drinks per drinking day	Average number of drinks on drinking days in past 30 days	
Heavy drinking occasions	Number of times participant drank heavily in past 30 days	
Peak drinks on a drinking day	Largest number of drinks consumed on any one occasion in past 30 days	
Alcohol consequences	Brief Young Adult Alcohol Consequences Questionnaire	
Any cannabis use in past 6 months	Have you used any cannabis?	How often have you used marijuana?
Total cannabis use days	How many days did you use cannabis?	How many days did you use marijuana?
Behavioral health services use		
Any mental health care since discharge	Any mental health care at VA or elsewhere	
Any mental health care in past year	Any mental health care at VA or elsewhere	
Any mental health care in past month	Any mental health care at VA or elsewhere	
Any substance use care since discharge	Any substance use care at VA or elsewhere	
Any substance use care in past year	Any substance use care at VA or elsewhere	
Any substance use care in past month	Any substance use care at VA or elsewhere	
Video game use		
Total hours spent playing per day	Average of typical hours on each day of week	Self-reported average of hours on a typical day
Total hours spent playing per week	Sum of typical hours on each day of week	Self-reported hours per day by days per week
Total days spent playing per week	Sum of days of the week of self-reported playing	Self-reported days in a typical week

Notes: PTSD = Posttraumatic Stress Disorder; VA = Veterans Affairs.
