# Supplementary material for: Video Games as a Potential Modality for Behavioral Health Services for Young Adult Veterans: Exploratory Analysis
Source: JMIR Serious Games. 2018 Jul 26;6(3):e15. doi: 10.2196/games.9327 (PMC6085553; doi:10.2196/games.9327)
Supplement: Multimedia Appendix 3 [file games_v6i3e15_app3.pdf]

Multimedia Appendix 3. Exploratory replication analyses of video game use by young adult veterans

Parameter	Hours per day		Hours per week		Days per week	
	Replicated Effect	MD	Replicated Effect	MD	Replicated Effect	MD
Video game use by positive screen						
Any positive screen	No	0.32 (-0.04, 0.67)	No	2.61 (0.11, 5.10)*	No	0.32 (-0.03, 0.66)
Any mental health screen	Yes	0.61 (0.30, 0.92)*	Yes	4.74 (2.54, 6.94)*	No	0.41 (0.13, 0.70)*
PTSD screen	Yes	0.58 (0.25, 0.90)*	Yes	4.64 (2.30, 6.98)*	No	0.40 (0.10, 0.69)*
Depression screen	Yes	0.61 (0.30, 0.92)*	Yes	4.77 (2.56, 6.99)*	No	0.39 (0.11, 0.68)*
Any SUD screen	No	-0.15 (-0.47, 0.16)	No	-0.87 (-3.13, 1.39)	No	0.07 (-0.22, 0.35)
AUD screen	No	-0.11 (-0.42, 0.20)	No	-0.37 (-2.63, 1.88)	No	0.20 (-0.10, 0.50)
Cannabis use	No	-0.26 (-0.62, 0.09)	No	-1.32 (-3.89, 1.25)	No	0.04 (-0.32, 0.40)
Video game use by services receipt						
Any services (any screen)						
Since discharge	No	0.29 (-0.06, 0.64)	No	2.67 (0.14, 5.20)*	No	0.48 (0.14, 0.82)*
Past year	No	0.04 (-0.31, 0.40)	No	0.26 (-2.34, 2.85)	No	0.15 (-0.18, 0.48)
Past month	No	0.22 (-0.27, 0.71)	No	0.91 (-2.58, 4.41)	No	0.26 (-0.15, 0.67)
Mental health services (PTSD or depression)						
Since discharge	No	0.31 (-0.18, 0.79)	No	2.84 (-0.63, 6.32)	No	0.36 (-0.12, 0.83)
Past year	No	-0.09 (-0.55, 0.37)	No	-0.83 (-4.21, 2.56)	No	0.09 (-0.32, 0.50)
Past month	No	0.14 (-0.44, 0.72)	No	-0.15 (-4.31, 4.01)	No	0.05 (-0.41, 0.51)
Mental health services (PTSD)						
Since discharge	No	0.22 (-0.38, 0.82)	No	2.62 (-1.67, 6.91)	No	0.41 (-0.15, 0.97)
Past year	No	-0.29 (-0.82, 0.25)	No	-2.46 (-6.42, 1.49)	No	0.03 (-0.44, 0.50)
Past month	No	0.10 (-0.54, 0.74)	No	-0.97 (-5.57, 3.63)	No	-0.13 (-0.63, 0.37)
Mental health services (depression)						
Since discharge	No	0.27 (-0.23, 0.78)	No	2.15 (-1.49, 5.79)	No	0.22 (-0.27, 0.70)
Past year	No	-0.10 (-0.58, 0.38)	No	-0.93 (-4.43, 2.57)	No	0.09 (-0.33, 0.51)
Past month	No	0.17 (-0.44, 0.77)	No	0.10 (-4.23, 4.43)	No	0.08 (-0.40, 0.56)
Substance use services (AUD or cannabis)						
Since discharge	No	0.10 (-0.37, 0.56)	No	1.57 (-1.65, 4.78)	No	0.75 (0.28, 1.21)*
Past year	No	-0.29 (-0.82, 0.25)	No	-1.40 (-5.28, 2.48)	No	0.44 (-0.17, 1.05)
Past month	N/A	N/A	N/A	N/A	N/A	N/A
Substance use services (AUD)						
Since discharge	No	0.37 (-0.22, 0.95)	No	3.71 (-0.50, 7.92)	No	0.92 (0.35, 1.49)*
Past year	No	0.06 (-0.73, 0.85)	No	-1.25 (-5.36, 2.86)	No	0.76 (-0.06, 1.57)
Past month	N/A	N/A	N/A	N/A	N/A	N/A
Substance use services (cannabis)						
Since discharge	No	-0.30 (-0.90, 0.30)	No	-0.59 (-4.83, 3.65)	No	0.70 (0.03, 1.36)*
Past year	No	-0.51 (-1.17, 0.15)	No	-2.03 (-6.71, 2.64)	No	0.53 (-0.22, 1.29)
Past month	N/A	N/A	N/A	N/A	N/A	N/A

Notes: Replicated = $p < .05$ and difference in same direction in both studies. MD = mean difference (95% CI) from fixed-effect meta-analysis of both studies. AUD = alcohol use disorder. N/A = Not applicable. PTSD = posttraumatic stress disorder. SUD = substance use disorder. * $p < .05$.
