# Supplementary material for: Video Games as a Potential Modality for Behavioral Health Services for Young Adult Veterans: Exploratory Analysis
Source: JMIR Serious Games. 2018 Jul 26;6(3):e15. doi: 10.2196/games.9327 (PMC6085553; doi:10.2196/games.9327)
Supplement: Multimedia Appendix 4 [file games_v6i3e15_app4.pdf]

Table of Contents

Packages and Datasets 9
Demographics 15
- Age 15
- Gender 16
- Hispanic ethnicity 16
- Race 17
- Highest education level 17
- Currently in college 18
- Annual household income 19
- Marital status 19
- Children 20
- Children living at home 21
- Military branch 22

Behavioral Health 22
- PTSD 22
- Depression 23
- AUDIT 23
- Total drinking days 24
- Drinks per drinking day 25
- Heavy drinking occasions 26
- Peak drinks on a drinking day 26
- Alcohol consequences 27
- Any cannabis use in past 6 months 28
- Total cannabis use days in past 30 days 28

Care Receipt 29
- Behavioral Health - Discharge 29
- Behavioral Health - Year 30
- Behavioral Health - Month 30
- Mental Health - Discharge 31
- Mental Health - Year 32
- Mental Health - Month 32
- Substance Use - Discharge 33

Substance Use - Year 33
Substance Use - Month 34
Video Game Behavior 35
Total hours spent playing per day 35
Total hours spent playing per week 35
Total days spent playing per week 36
Any positive screen 37
Replicated Effect 37
Hours per day 37
Hours per week 38
Days per week 38
MD 39
Hours per day 39
Hours per week 41
Days per week 42
Any mental health screen 44
Replicated Effect 44
Hours per day 44
Hours per week 45
Days per week 45
MD 46
Hours per day 46
Hours per week 48
Days per week 49
PTSD screen 51
Replicated Effect 51
Hours per day 51
Hours per week 52
Days per week 52
MD 53
Hours per day 53
Hours per week 54
Days per week 56
Depression screen 57

Replicated Effect 57
Hours per day 57
Hours per week 58
Days per week 59
MD 60
Hours per day 60
Hours per week 61
Days per week 63
Any SUD screen 64
Replicated Effect 64
Hours per day 64
Hours per week 65
Days per week 66
MD 67
Hours per day 67
Hours per week 68
Days per week 70
AUD screen 71
Replicated Effect 71
Hours per day 71
Hours per week 72
Days per week 73
MD 74
Hours per day 74
Hours per week 75
Days per week 76
Cannabis screen 78
Replicated Effect 78
Hours per day 78
Hours per week 79
Days per week 80
MD 80
Hours per day 80
Hours per week 82

Days per week 83
Any care - Discharge 85
Replicated Effect 85
Hours per day 85
Hours per week 86
Days per week 86
MD 87
Hours per day 87
Hours per week 89
Days per week 90
Any care - Year 92
Replicated Effect 92
Hours per day 92
Hours per week 93
Days per week 93
MD 94
Hours per day 94
Hours per week 96
Days per week 97
Any care - Month 99
Replicated Effect 99
Hours per day 99
Hours per week 100
Days per week 100
MD 101
Hours per day 101
Hours per week 103
Days per week 104
Mental health care - discharge 106
Replicated Effect 106
Hours per day 106
Hours per week 107
Days per week 107
MD 108

Hours per day 108
Hours per week 110
Days per week 111
Mental health care - year 113
Replicated Effect 113
Hours per day 113
Hours per week 113
Days per week 114
MD 115
Hours per day 115
Hours per week 116
Days per week 118
Mental health care - month 120
Replicated Effect 120
Hours per day 120
Hours per week 120
Days per week 121
MD 122
Hours per day 122
Hours per week 123
Days per week 125
PTSD care - discharge 127
Replicated Effect 127
Hours per day 127
Hours per week 127
Days per week 128
MD 129
Hours per day 129
Hours per week 130
Days per week 132
PTSD care - year 134
Replicated Effect 134
Hours per day 134
Hours per week 134

Days per week 135
MD 136
Hours per day 136
Hours per week 137
Days per week 139
PTSD care - month 141
Replicated Effect 141
Hours per day 141
Hours per week 141
Days per week 142
MD 143
Hours per day 143
Hours per week 144
Days per week 146
Depression care - discharge 148
Replicated Effect 148
Hours per day 148
Hours per week 148
Days per week 149
MD 150
Hours per day 150
Hours per week 151
Days per week 153
Depression care - year 155
Replicated Effect 155
Hours per day 155
Hours per week 156
Days per week 156
MD 157
Hours per day 157
Hours per week 159
Days per week 160
Depression care - month 162
Replicated Effect 162

Hours per day 162
Hours per week 163
Days per week 163
MD 164
Hours per day 164
Hours per week 166
Days per week 167
Any SUD - discharge 169
Replicated Effect 169
Hours per day 169
Hours per week 170
Days per week 171
MD 171
Hours per day 171
Hours per week 173
Days per week 175
Any SUD - year 176
Replicated Effect 176
Hours per day 176
Hours per week 177
Days per week 178
MD 179
Hours per day 179
Hours per week 180
Days per week 182
Any SUD - month 184
Replicated Effect 184
Hours per day 184
Hours per week 184
Days per week 185
MD 185
Hours per day 185
Hours per week 187
Days per week 188

AUD - discharge........ 190
  Replicated Effect........ 190
    Hours per day........ 190
    Hours per week........ 191
    Days per week........ 192
  MD........ 192
    Hours per day........ 192
    Hours per week........ 194
    Days per week........ 195
AUD - year........ 197
  Replicated Effect........ 197
    Hours per day........ 197
    Hours per week........ 198
    Days per week........ 199
  MD........ 199
    Hours per day........ 199
    Hours per week........ 201
    Days per week........ 202
AUD - month........ 205
  Replicated Effect........ 205
    Hours per day........ 205
    Hours per week........ 205
    Days per week........ 205
  MD........ 205
    Hours per day - CANNOT CALCULATE........ 205
    Hours per week - CANNOT CALCULATE........ 205
    Days per week - CANNOT CALCULATE........ 205
Cannabis - discharge........ 205
  Replicated Effect........ 205
    Hours per day........ 205
    Hours per week........ 206
    Days per week........ 207
  MD........ 208
    Hours per day........ 208

Hours per week 209
Days per week 211
Cannabis - year 212
Replicated Effect 212
Hours per day 212
Hours per week 213
Days per week 214
MD 215
Hours per day 215
Hours per week 216
Days per week 218
Cannabis - month 219
Replicated Effect 219
Hours per day 219
Hours per week 220
Days per week 220
MD 221
Hours per day 221
Hours per week 222
Days per week 224

Packages and Datasets

```
### Load "Read Excel Files" package
library(readxl)

### Load "Package for Analysis of Space-Time Ecological Series" package
library(pastecs)

### Load "Procedures for Psychological, Psychometric, and Personality
Research" package
library(psych)

### Load "Various R Programming Tools for Model Fitting" package
library(gmodels)

### Load "Support Functions and Datasets for Venables and Ripley's MASS"
package
```

```
library(MASS)

### Load "Functions that Apply to Rows and Columns of Matrices (and to
Vectors)" package
library(matrixStats)

### Load "Meta-Analysis Package for R"
library(metafor)

### Load "General Package for Meta-Analysis"
library(meta)

### Upload data from Excel file
dboth <- read_excel("5. Grant - Veteran Video Game Behavioral Health
Revision - Multimedia Appendix 5.xlsx")

#### Create Study 1 and Study 2 Datasets
ds1 <- subset(dboth, study == "1", select = age:vgdaysweek)
ds2 <- subset(dboth, study == "2", select = age:vgdaysweek)

#### Create Positive Screen Datasets
anyscreen_yes_ds1 <- subset(ds1, anyscreen == "1", select = c(vghrsday,
vghrsweek, vgdaysweek))
anyscreen_no_ds1 <- subset(ds1, anyscreen == "0", select = c(vghrsday,
vghrsweek, vgdaysweek))
anyscreen_yes_ds2 <- subset(ds2, anyscreen == "1", select = c(vghrsday,
vghrsweek, vgdaysweek))
anyscreen_no_ds2 <- subset(ds2, anyscreen == "0", select = c(vghrsday,
vghrsweek, vgdaysweek))
anymhscreen_yes_ds1 <- subset(ds1, anymhscreen == "1", select =
c(vghrsday, vghrsweek, vgdaysweek))
anymhscreen_no_ds1 <- subset(ds1, anymhscreen == "0", select =
c(vghrsday, vghrsweek, vgdaysweek))
anymhscreen_yes_ds2 <- subset(ds2, anymhscreen == "1", select =
c(vghrsday, vghrsweek, vgdaysweek))
anymhscreen_no_ds2 <- subset(ds2, anymhscreen == "0", select =
c(vghrsday, vghrsweek, vgdaysweek))
ptsd_yes_ds1 <- subset(ds1, ptsd == "1", select = c(vghrsday, vghrsweek,
vgdaysweek))
ptsd_no_ds1 <- subset(ds1, ptsd == "0", select = c(vghrsday, vghrsweek,
vgdaysweek))
ptsd_yes_ds2 <- subset(ds2, ptsd == "1", select = c(vghrsday, vghrsweek,
vgdaysweek))
ptsd_no_ds2 <- subset(ds2, ptsd == "0", select = c(vghrsday, vghrsweek,
vgdaysweek))
depression_yes_ds1 <- subset(ds1, depression == "1", select = c(vghrsday,
vghrsweek, vgdaysweek))
depression_no_ds1 <- subset(ds1, depression == "0", select = c(vghrsday,
vghrsweek, vgdaysweek))
depression_yes_ds2 <- subset(ds2, depression == "1", select = c(vghrsday,
```

```
vghrsweek, vgdaysweek))
depression_no_ds2 <- subset(ds2, depression == "0", select = c(vghrsday,
vghrsweek, vgdaysweek))
anysudscreen_yes_ds1 <- subset(ds1, anysudscreen == "1", select =
c(vghrsday, vghrsweek, vgdaysweek))
anysudscreen_no_ds1 <- subset(ds1, anysudscreen == "0", select =
c(vghrsday, vghrsweek, vgdaysweek))
anysudscreen_yes_ds2 <- subset(ds2, anysudscreen == "1", select =
c(vghrsday, vghrsweek, vgdaysweek))
anysudscreen_no_ds2 <- subset(ds2, anysudscreen == "0", select =
c(vghrsday, vghrsweek, vgdaysweek))
audit_yes_ds1 <- subset(ds1, audit == "1", select = c(vghrsday, vghrsweek,
vgdaysweek))
audit_no_ds1 <- subset(ds1, audit == "0", select = c(vghrsday, vghrsweek,
vgdaysweek))
audit_yes_ds2 <- subset(ds2, audit == "1", select = c(vghrsday, vghrsweek,
vgdaysweek))
audit_no_ds2 <- subset(ds2, audit == "0", select = c(vghrsday, vghrsweek,
vgdaysweek))
canscreen_yes_ds1 <- subset(ds1, canscreen == "1", select = c(vghrsday,
vghrsweek, vgdaysweek))
canscreen_no_ds1 <- subset(ds1, canscreen == "0", select = c(vghrsday,
vghrsweek, vgdaysweek))
canscreen_yes_ds2 <- subset(ds2, canscreen == "1", select = c(vghrsday,
vghrsweek, vgdaysweek))
canscreen_no_ds2 <- subset(ds2, canscreen == "0", select = c(vghrsday,
vghrsweek, vgdaysweek))

#### Create Study 1 Care Receipt Datasets
ds1anyscreen <- subset(ds1, anyscreen == "1", select = age:vgdaysweek)
anycaredis_yes_ds1 <- subset(ds1anyscreen, anydis == "1", select =
c(vghrsday, vghrsweek, vgdaysweek))
anycaredis_no_ds1 <- subset(ds1anyscreen, anydis == "0", select =
c(vghrsday, vghrsweek, vgdaysweek))
anycareyear_yes_ds1 <- subset(ds1anyscreen, anyyear == "1", select =
c(vghrsday, vghrsweek, vgdaysweek))
anycareyear_no_ds1 <- subset(ds1anyscreen, anyyear == "0", select =
c(vghrsday, vghrsweek, vgdaysweek))
anycaremonth_yes_ds1 <- subset(ds1anyscreen, anymonth == "1", select =
c(vghrsday, vghrsweek, vgdaysweek))
anycaremonth_no_ds1 <- subset(ds1anyscreen, anymonth == "0", select =
c(vghrsday, vghrsweek, vgdaysweek))
ds1anymhscreen <- subset(ds1, anymhscreen == "1", select =
age:vgdaysweek)
mhdis_yes_ds1 <- subset(ds1anymhscreen, mhdis == "1", select =
c(vghrsday, vghrsweek, vgdaysweek))
mhdis_no_ds1 <- subset(ds1anymhscreen, mhdis == "0", select =
c(vghrsday, vghrsweek, vgdaysweek))
mhyear_yes_ds1 <- subset(ds1anymhscreen, mhyear == "1", select =
c(vghrsday, vghrsweek, vgdaysweek))
```

```
mhyear_no_ds1 <- subset(ds1anymhscreen, mhyear == "0", select =
c(vghrsday, vghrsweek, vgdaysweek))
mhmonth_yes_ds1 <- subset(ds1anymhscreen, mhmonth == "1", select =
c(vghrsday, vghrsweek, vgdaysweek))
mhmonth_no_ds1 <- subset(ds1anymhscreen, mhmonth == "0", select =
c(vghrsday, vghrsweek, vgdaysweek))
ds1ptsd <- subset(ds1, ptsd == "1", select = age:vgdaysweek)
ptsddis_yes_ds1 <- subset(ds1ptsd, mhdis == "1", select = c(vghrsday,
vghrsweek, vgdaysweek))
ptsddis_no_ds1 <- subset(ds1ptsd, mhdis == "0", select = c(vghrsday,
vghrsweek, vgdaysweek))
ptsdyear_yes_ds1 <- subset(ds1ptsd, mhyear == "1", select = c(vghrsday,
vghrsweek, vgdaysweek))
ptsdyear_no_ds1 <- subset(ds1ptsd, mhyear == "0", select = c(vghrsday,
vghrsweek, vgdaysweek))
ptsdmonth_yes_ds1 <- subset(ds1ptsd, mhmonth == "1", select =
c(vghrsday, vghrsweek, vgdaysweek))
ptsdmonth_no_ds1 <- subset(ds1ptsd, mhmonth == "0", select =
c(vghrsday, vghrsweek, vgdaysweek))
ds1depression <- subset(ds1, depression == "1", select = age:vgdaysweek)
depressiondis_yes_ds1 <- subset(ds1depression, mhdis == "1", select =
c(vghrsday, vghrsweek, vgdaysweek))
depressiondis_no_ds1 <- subset(ds1depression, mhdis == "0", select =
c(vghrsday, vghrsweek, vgdaysweek))
depressionyear_yes_ds1 <- subset(ds1depression, mhyear == "1", select =
c(vghrsday, vghrsweek, vgdaysweek))
depressionyear_no_ds1 <- subset(ds1depression, mhyear == "0", select =
c(vghrsday, vghrsweek, vgdaysweek))
depressionmonth_yes_ds1 <- subset(ds1depression, mhmonth == "1", select
= c(vghrsday, vghrsweek, vgdaysweek))
depressionmonth_no_ds1 <- subset(ds1depression, mhmonth == "0", select
= c(vghrsday, vghrsweek, vgdaysweek))
ds1anysudscreen <- subset(ds1, anysudscreen == "1", select =
age:vgdaysweek)
anysudscreendis_yes_ds1 <- subset(ds1anysudscreen, alcsuddis == "1",
select = c(vghrsday, vghrsweek, vgdaysweek))
anysudscreendis_no_ds1 <- subset(ds1anysudscreen, alcsuddis == "0",
select = c(vghrsday, vghrsweek, vgdaysweek))
anysudscreenyear_yes_ds1 <- subset(ds1anysudscreen, alcsudyear == "1",
select = c(vghrsday, vghrsweek, vgdaysweek))
anysudscreenyear_no_ds1 <- subset(ds1anysudscreen, alcsudyear == "0",
select = c(vghrsday, vghrsweek, vgdaysweek))
anysudscreenmonth_yes_ds1 <- subset(ds1anysudscreen, alcsudmonth ==
"1", select = c(vghrsday, vghrsweek, vgdaysweek))
anysudscreenmonth_no_ds1 <- subset(ds1anysudscreen, alcsudmonth ==
"0", select = c(vghrsday, vghrsweek, vgdaysweek))
ds1audit <- subset(ds1, audit == "1", select = age:vgdaysweek)
auditdis_yes_ds1 <- subset(ds1audit, alcsuddis == "1", select = c(vghrsday,
vghrsweek, vgdaysweek))
auditdis_no_ds1 <- subset(ds1audit, alcsuddis == "0", select = c(vghrsday,
```

```
vghrsweek, vgdaysweek))
audityear_yes_ds1 <- subset(ds1audit, alcsudyear == "1", select =
c(vghrsday, vghrsweek, vgdaysweek))
audityear_no_ds1 <- subset(ds1audit, alcsudyear == "0", select =
c(vghrsday, vghrsweek, vgdaysweek))
auditmonth_yes_ds1 <- subset(ds1audit, alcsudmonth == "1", select =
c(vghrsday, vghrsweek, vgdaysweek))
auditmonth_no_ds1 <- subset(ds1audit, alcsudmonth == "0", select =
c(vghrsday, vghrsweek, vgdaysweek))
ds1canscreen <- subset(ds1, canscreen == "1", select = age:vgdaysweek)
cannabisdis_yes_ds1 <- subset(ds1canscreen, alcsuddis == "1", select =
c(vghrsday, vghrsweek, vgdaysweek))
cannabisdis_no_ds1 <- subset(ds1canscreen, alcsuddis == "0", select =
c(vghrsday, vghrsweek, vgdaysweek))
cannabisyear_yes_ds1 <- subset(ds1canscreen, alcsudyear == "1", select =
c(vghrsday, vghrsweek, vgdaysweek))
cannabisyear_no_ds1 <- subset(ds1canscreen, alcsudyear == "0", select =
c(vghrsday, vghrsweek, vgdaysweek))
cannabismonth_yes_ds1 <- subset(ds1canscreen, alcsudmonth == "1",
select = c(vghrsday, vghrsweek, vgdaysweek))
cannabismonth_no_ds1 <- subset(ds1canscreen, alcsudmonth == "0", select
= c(vghrsday, vghrsweek, vgdaysweek))

#### Create Study 2 Care Receipt Datasets
ds2anyscreen <- subset(ds2, anyscreen == "1", select = age:vgdaysweek)
anycaredis_yes_ds2 <- subset(ds2anyscreen, anydis == "1", select =
c(vghrsday, vghrsweek, vgdaysweek))
anycaredis_no_ds2 <- subset(ds2anyscreen, anydis == "0", select =
c(vghrsday, vghrsweek, vgdaysweek))
anycareyear_yes_ds2 <- subset(ds2anyscreen, anyyear == "1", select =
c(vghrsday, vghrsweek, vgdaysweek))
anycareyear_no_ds2 <- subset(ds2anyscreen, anyyear == "0", select =
c(vghrsday, vghrsweek, vgdaysweek))
anycaremonth_yes_ds2 <- subset(ds2anyscreen, anymonth == "1", select =
c(vghrsday, vghrsweek, vgdaysweek))
anycaremonth_no_ds2 <- subset(ds2anyscreen, anymonth == "0", select =
c(vghrsday, vghrsweek, vgdaysweek))
ds2anymhscreen <- subset(ds2, anymhscreen == "1", select =
age:vgdaysweek)
mhdis_yes_ds2 <- subset(ds2anymhscreen, mhdis == "1", select =
c(vghrsday, vghrsweek, vgdaysweek))
mhdis_no_ds2 <- subset(ds2anymhscreen, mhdis == "0", select =
c(vghrsday, vghrsweek, vgdaysweek))
mhyear_yes_ds2 <- subset(ds2anymhscreen, mhyear == "1", select =
c(vghrsday, vghrsweek, vgdaysweek))
mhyear_no_ds2 <- subset(ds2anymhscreen, mhyear == "0", select =
c(vghrsday, vghrsweek, vgdaysweek))
mhmonth_yes_ds2 <- subset(ds2anymhscreen, mhmonth == "1", select =
c(vghrsday, vghrsweek, vgdaysweek))
mhmonth_no_ds2 <- subset(ds2anymhscreen, mhmonth == "0", select =
```

```
c(vghrsday, vghrsweek, vgdaysweek))
ds2ptsd <- subset(ds2, ptsd == "1", select = age:vgdaysweek)
ptsddis_yes_ds2 <- subset(ds2ptsd, mhdis == "1", select = c(vghrsday,
vghrsweek, vgdaysweek))
ptsddis_no_ds2 <- subset(ds2ptsd, mhdis == "0", select = c(vghrsday,
vghrsweek, vgdaysweek))
ptsdyear_yes_ds2 <- subset(ds2ptsd, mhyear == "1", select = c(vghrsday,
vghrsweek, vgdaysweek))
ptsdyear_no_ds2 <- subset(ds2ptsd, mhyear == "0", select = c(vghrsday,
vghrsweek, vgdaysweek))
ptsdmonth_yes_ds2 <- subset(ds2ptsd, mhmonth == "1", select =
c(vghrsday, vghrsweek, vgdaysweek))
ptsdmonth_no_ds2 <- subset(ds2ptsd, mhmonth == "0", select =
c(vghrsday, vghrsweek, vgdaysweek))
ds2depression <- subset(ds2, depression == "1", select = age:vgdaysweek)
depressiondis_yes_ds2 <- subset(ds2depression, mhdis == "1", select =
c(vghrsday, vghrsweek, vgdaysweek))
depressiondis_no_ds2 <- subset(ds2depression, mhdis == "0", select =
c(vghrsday, vghrsweek, vgdaysweek))
depressionyear_yes_ds2 <- subset(ds2depression, mhyear == "1", select =
c(vghrsday, vghrsweek, vgdaysweek))
depressionyear_no_ds2 <- subset(ds2depression, mhyear == "0", select =
c(vghrsday, vghrsweek, vgdaysweek))
depressionmonth_yes_ds2 <- subset(ds2depression, mhmonth == "1", select
= c(vghrsday, vghrsweek, vgdaysweek))
depressionmonth_no_ds2 <- subset(ds2depression, mhmonth == "0", select
= c(vghrsday, vghrsweek, vgdaysweek))
ds2anysudscreen <- subset(ds2, anysudscreen == "1", select =
age:vgdaysweek)
anysudscreendis_yes_ds2 <- subset(ds2anysudscreen, alcsuddis == "1",
select = c(vghrsday, vghrsweek, vgdaysweek))
anysudscreendis_no_ds2 <- subset(ds2anysudscreen, alcsuddis == "0",
select = c(vghrsday, vghrsweek, vgdaysweek))
anysudscreenyear_yes_ds2 <- subset(ds2anysudscreen, alcsudyear == "1",
select = c(vghrsday, vghrsweek, vgdaysweek))
anysudscreenyear_no_ds2 <- subset(ds2anysudscreen, alcsudyear == "0",
select = c(vghrsday, vghrsweek, vgdaysweek))
anysudscreenmonth_yes_ds2 <- subset(ds2anysudscreen, alcsudmonth ==
"1", select = c(vghrsday, vghrsweek, vgdaysweek))
anysudscreenmonth_no_ds2 <- subset(ds2anysudscreen, alcsudmonth ==
"0", select = c(vghrsday, vghrsweek, vgdaysweek))
ds2audit <- subset(ds2, audit == "1", select = age:vgdaysweek)
auditdis_yes_ds2 <- subset(ds2audit, alcsuddis == "1", select = c(vghrsday,
vghrsweek, vgdaysweek))
auditdis_no_ds2 <- subset(ds2audit, alcsuddis == "0", select = c(vghrsday,
vghrsweek, vgdaysweek))
audityear_yes_ds2 <- subset(ds2audit, alcsudyear == "1", select =
c(vghrsday, vghrsweek, vgdaysweek))
audityear_no_ds2 <- subset(ds2audit, alcsudyear == "0", select =
c(vghrsday, vghrsweek, vgdaysweek))
```

```
auditmonth_yes_ds2 <- subset(ds2audit, alcsudmonth == "1", select =
c(vghrsday, vghrsweek, vgdaysweek))
auditmonth_no_ds2 <- subset(ds2audit, alcsudmonth == "0", select =
c(vghrsday, vghrsweek, vgdaysweek))
ds2canscreen <- subset(ds2, canscreen == "1", select = age:vgdaysweek)
cannabisdis_yes_ds2 <- subset(ds2canscreen, alcsuddis == "1", select =
c(vghrsday, vghrsweek, vgdaysweek))
cannabisdis_no_ds2 <- subset(ds2canscreen, alcsuddis == "0", select =
c(vghrsday, vghrsweek, vgdaysweek))
cannabisyear_yes_ds2 <- subset(ds2canscreen, alcsudyear == "1", select =
c(vghrsday, vghrsweek, vgdaysweek))
cannabisyear_no_ds2 <- subset(ds2canscreen, alcsudyear == "0", select =
c(vghrsday, vghrsweek, vgdaysweek))
cannabismonth_yes_ds2 <- subset(ds2canscreen, alcsudmonth == "1",
select = c(vghrsday, vghrsweek, vgdaysweek))
cannabismonth_no_ds2 <- subset(ds2canscreen, alcsudmonth == "0", select
= c(vghrsday, vghrsweek, vgdaysweek))
```

Demographics

Age

```
# Study 1
ds1_age <- subset(ds1, select = c(age))
ds1_age <- data.frame(mean = apply(ds1_age, 2, mean), sd =
apply(ds1_age, 2, sd), n = colSums(!is.na(ds1_age)))

# Study 2
ds2_age <- subset(ds2, select = c(age))
ds2_age <- data.frame(mean = apply(ds2_age, 2, mean), sd =
apply(ds2_age, 2, sd), n = colSums(!is.na(ds2_age)))

# Combined
dboth_age <- rbind(ds1_age, ds2_age)
rownames(dboth_age) <- c("Study 1", "Study 2")
dboth_age

##             mean       sd   n
## Study 1 28.35714 3.364383 350
## Study 2 28.72680 3.418982 582

# Welch Two Sample t-test
(diff_age <- t.test(age ~ study, data=dboth))

##
##  Welch Two Sample t-test
##
## data:  age by study
## t = -1.6145, df = 744.59, p-value = 0.1068
## alternative hypothesis: true difference in means is not equal to 0
```

```
## 95 percent confidence interval:
##  -0.81915586  0.07983332
## sample estimates:
## mean in group 1 mean in group 2
##        28.35714        28.72680
```

Gender

```
# Percentages
attach(dboth)
dboth_gender <- table(study,gender)
colnames(dboth_gender) <- c("Female", "Male")
prop.table(dboth_gender, 1)
```

```
##      gender
## study     Female       Male
##     1 0.07449857 0.92550143
##     2 0.13230241 0.86769759
```

```
# Sample Sizes
margin.table(dboth_gender, 1)
```

```
## study
##   1   2
## 349 582
```

```
# Chi-squared test
chisq.test(dboth_gender)
```

```
##
##  Pearson's Chi-squared test with Yates' continuity correction
##
## data:  dboth_gender
## X-squared = 6.8329, df = 1, p-value = 0.008949
```

Hispanic ethnicity

```
# Percentages
attach(dboth)
dboth_hispanic <- table(study,hispanic)
colnames(dboth_hispanic) <- c("No", "Yes")
prop.table(dboth_hispanic, 1)
```

```
##      hispanic
## study        No       Yes
##     1 0.7936963 0.2063037
##     2 0.8969072 0.1030928
```

```
# Sample Sizes
margin.table(dboth_hispanic, 1)
```

```
## study
##   1   2
## 349 582
```

```
# Chi-squared test
chisq.test(dboth_hispanic)
```

```
##
##  Pearson's Chi-squared test with Yates' continuity correction
##
## data:  dboth_hispanic
## X-squared = 18.261, df = 1, p-value = 1.926e-05
```

Race

```
# Percentages
attach(dboth)
dboth_race <- table(study,racew)
colnames(dboth_race) <- c("Non-White", "White")
prop.table(dboth_race, 1)
```

```
##      racew
## study Non-White     White
##     1 0.2200000 0.7800000
##     2 0.1426117 0.8573883
```

```
# Sample Sizes
margin.table(dboth_race, 1)
```

```
## study
##   1   2
## 350 582
```

```
# Chi-squared test
chisq.test(dboth_race)
```

```
##
##  Pearson's Chi-squared test with Yates' continuity correction
##
## data:  dboth_race
## X-squared = 8.6688, df = 1, p-value = 0.003237
```

Highest education level

```
# Percentages
attach(dboth)
dboth_education <- table(study,educationc)
```

```
colnames(dboth_education) <- c("Not College Graduate", "College Graduate")
prop.table(dboth_education, 1)

##      educationc
## study Not College Graduate College Graduate
##     1            0.8171429        0.1828571
##     2            0.8006873        0.1993127

# Sample Sizes
margin.table(dboth_education, 1)

## study
##   1   2
## 350 582

# Chi-squared test
chisq.test(dboth_education)

##
##  Pearson's Chi-squared test with Yates' continuity correction
##
## data:  dboth_education
## X-squared = 0.28153, df = 1, p-value = 0.5957
```

Currently in college

```
# Percentages
attach(dboth)
dboth_college <- table(study,collegeyes)
colnames(dboth_college) <- c("Not in College", "In College")
prop.table(dboth_college, 1)

##      collegeyes
## study Not in College In College
##     1      0.5342857  0.4657143
##     2      0.6563574  0.3436426

# Sample Sizes
margin.table(dboth_college, 1)

## study
##   1   2
## 350 582

# Chi-squared test
chisq.test(dboth_college)

##
##  Pearson's Chi-squared test with Yates' continuity correction
##
```

```
## data:  dboth_college
## X-squared = 13.188, df = 1, p-value = 0.0002817
```

Annual household income

```
# Percentages
attach(dboth)
dboth_income <- table(study,income)
colnames(dboth_income) <- c("Less than $10,000", "$10,000 to $14,999",
"$15,000 to $24,999", "$25,000 to $49,999", "$50,000 to $99,999",
"$100,000 to $149,999", "$150,000 to $199,999", "$200,000 or more")
prop.table(dboth_income, 1)

##      income
## study Less than $10,000 $10,000 to $14,999 $15,000 to $24,999
##     1       0.082857143        0.082857143        0.200000000
##     2       0.054982818        0.079037801        0.154639175
##      income
## study $25,000 to $49,999 $50,000 to $99,999 $100,000 to $149,999
##     1        0.345714286        0.240000000          0.040000000
##     2        0.408934708        0.243986254          0.048109966
##      income
## study $150,000 to $199,999 $200,000 or more
##     1          0.008571429      0.000000000
##     2          0.008591065      0.001718213

# Sample Sizes
margin.table(dboth_income, 1)

## study
##   1   2
## 350 582

# Chi-squared test
chisq.test(dboth_income)

##
##  Pearson's Chi-squared test
##
## data:  dboth_income
## X-squared = 8.4563, df = 7, p-value = 0.2941
```

Marital status

```
# Percentages
attach(dboth)
dboth_married <- table(study,married)
colnames(dboth_married) <- c("Not Married", "Married")
prop.table(dboth_married, 1)
```

```
##      married
## study Not Married   Married
##     1   0.4685714 0.5314286
##     2   0.5223368 0.4776632
```

```
# Sample Sizes
margin.table(dboth_married, 1)
```

```
## study
##   1   2
## 350 582
```

```
# Chi-squared test
chisq.test(dboth_married)
```

```
##
##  Pearson's Chi-squared test with Yates' continuity correction
##
## data:  dboth_married
## X-squared = 2.3168, df = 1, p-value = 0.128
```

Children

```
# Study 1
ds1_children <- subset(ds1, select = c(children))
ds1_children <- data.frame(mean = apply(ds1_children, 2, mean), sd =
apply(ds1_children, 2, sd), n = colSums(!is.na(ds1_children)))

# Study 2
ds2_children <- subset(ds2, select = c(children))
ds2_children <- data.frame(mean = apply(ds2_children, 2, mean), sd =
apply(ds2_children, 2, sd), n = colSums(!is.na(ds2_children)))

# Combined
dboth_children <- rbind(ds1_children, ds2_children)
rownames(dboth_children) <- c("Study 1", "Study 2")
dboth_children
```

```
##             mean       sd   n
## Study 1 1.425714 1.463568 350
## Study 2 1.281787 1.420157 582
```

```
# Welch Two Sample t-test
(diff_children <- t.test(children ~ study, data=dboth))
```

```
##
##  Welch Two Sample t-test
##
## data:  children by study
## t = 1.4701, df = 717.87, p-value = 0.142
```

```
## alternative hypothesis: true difference in means is not equal to 0
## 95 percent confidence interval:
##  -0.04828768  0.33614236
## sample estimates:
## mean in group 1 mean in group 2
##        1.425714        1.281787
```

Children living at home

```
# Study 1
ds1_childrenhome <- subset(ds1, select = c(childrenathome))
ds1_childrenhome <- na.omit(ds1_childrenhome)
ds1_childrenhome <- data.frame(mean = apply(ds1_childrenhome, 2, mean),
sd = apply(ds1_childrenhome, 2, sd), n = colSums(!is.na(ds1_childrenhome)))

# Study 2
ds2_childrenhome <- subset(ds2, select = c(childrenathome))
ds2_childrenhome <- na.omit(ds2_childrenhome)
ds2_childrenhome <- data.frame(mean = apply(ds2_childrenhome, 2, mean),
sd = apply(ds2_childrenhome, 2, sd), n = colSums(!is.na(ds2_childrenhome)))

# Combined
dboth_childrenhome <- rbind(ds1_childrenhome, ds2_childrenhome)
rownames(dboth_childrenhome) <- c("Study 1", "Study 2")
dboth_childrenhome

##             mean       sd   n
## Study 1 1.715596 1.136668 218
## Study 2 1.738506 1.273996 348

# Welch Two Sample t-test
(diff_childrenhome <- t.test(childrenathome ~ study, data=dboth))

##
##  Welch Two Sample t-test
##
## data:  childrenathome by study
## t = -0.22261, df = 499.48, p-value = 0.8239
## alternative hypothesis: true difference in means is not equal to 0
## 95 percent confidence interval:
##  -0.2251010  0.1792822
## sample estimates:
## mean in group 1 mean in group 2
##        1.715596        1.738506
```

Military branch

```
# Percentages
attach(dboth)
dboth_branch <- table(study,branch)
colnames(dboth_branch) <- c("Air Force", "Army", "Marine Corps", "Navy")
prop.table(dboth_branch, 1)

##      branch
## study  Air Force       Army Marine Corps       Navy
##     1 0.06285714 0.61428571   0.24857143 0.07428571
##     2 0.09793814 0.59793814   0.22164948 0.08247423

# Sample Sizes
margin.table(dboth_branch, 1)

## study
##   1   2
## 350 582

# Chi-squared test
chisq.test(dboth_branch)

##
##  Pearson's Chi-squared test
##
## data:  dboth_branch
## X-squared = 4.1381, df = 3, p-value = 0.2469
```

Behavioral Health

PTSD

```
# Percentages
attach(dboth)
dboth_ptsd <- table(study,ptsd)
colnames(dboth_ptsd) <- c("No", "Yes")
prop.table(dboth_ptsd, 1)

##      ptsd
## study        No       Yes
##     1 0.5657143 0.4342857
##     2 0.6099656 0.3900344

# Sample Sizes
margin.table(dboth_ptsd, 1)

## study
##   1   2
## 350 582
```

```
# Chi-squared test
chisq.test(dboth_ptsd)

##
##  Pearson's Chi-squared test with Yates' continuity correction
##
## data:  dboth_ptsd
## X-squared = 1.5951, df = 1, p-value = 0.2066
```

Depression

```
# Percentages
attach(dboth)
dboth_depression <- table(study,depression)
colnames(dboth_depression) <- c("No", "Yes")
prop.table(dboth_depression, 1)

##      depression
## study        No       Yes
##     1 0.5171429 0.4828571
##     2 0.5343643 0.4656357

# Sample Sizes
margin.table(dboth_depression, 1)

## study
##   1   2
## 350 582

# Chi-squared test
chisq.test(dboth_depression)

##
##  Pearson's Chi-squared test with Yates' continuity correction
##
## data:  dboth_depression
## X-squared = 0.19558, df = 1, p-value = 0.6583
```

AUDIT

```
# Percentages
attach(dboth)
dboth_audit <- table(study,audit)
colnames(dboth_audit) <- c("No", "Yes")
prop.table(dboth_audit, 1)

##      audit
## study        No       Yes
```

```
##     1 0.5685714 0.4314286
##     2 0.7010309 0.2989691

# Sample Sizes
margin.table(dboth_audit, 1)

## study
##   1   2
## 350 582

# Chi-squared test
chisq.test(dboth_audit)

##
##  Pearson's Chi-squared test with Yates' continuity correction
##
## data:  dboth_audit
## X-squared = 16.307, df = 1, p-value = 5.387e-05
```

Total drinking days

```
# Study 1
ds1_totdrinkdays <- subset(ds1, select = c(totdrinkdays))
ds1_totdrinkdays <- na.omit(ds1_totdrinkdays)
ds1_totdrinkdays <- data.frame(mean = apply(ds1_totdrinkdays, 2, mean),
sd = apply(ds1_totdrinkdays, 2, sd), n = colSums(!is.na(ds1_totdrinkdays)))

# Study 2
ds2_totdrinkdays <- subset(ds2, select = c(totdrinkdays))
ds2_totdrinkdays <- na.omit(ds2_totdrinkdays)
ds2_totdrinkdays <- data.frame(mean = apply(ds2_totdrinkdays, 2, mean),
sd = apply(ds2_totdrinkdays, 2, sd), n = colSums(!is.na(ds2_totdrinkdays)))

# Combined
dboth_totdrinkdays <- rbind(ds1_totdrinkdays, ds2_totdrinkdays)
rownames(dboth_totdrinkdays) <- c("Study 1", "Study 2")
dboth_totdrinkdays

##             mean       sd   n
## Study 1 10.43714 9.070579 350
## Study 2 12.39175 8.752347 582

# Welch Two Sample t-test
(dboth_totdrinkdays <- t.test(totdrinkdays ~ study, data=dboth))

##
##  Welch Two Sample t-test
##
## data:  totdrinkdays by study
## t = -3.2278, df = 714.65, p-value = 0.001304
```

```
## alternative hypothesis: true difference in means is not equal to 0
## 95 percent confidence interval:
##  -3.1434843 -0.7657351
## sample estimates:
## mean in group 1 mean in group 2
##        10.43714        12.39175
```

Drinks per drinking day

```
# Study 1
ds1_avgdrinksday <- subset(ds1, select = c(avgdrinksday))
ds1_avgdrinksday <- na.omit(ds1_avgdrinksday)
ds1_avgdrinksday <- data.frame(mean = apply(ds1_avgdrinksday, 2, mean),
sd = apply(ds1_avgdrinksday, 2, sd), n = colSums(!is.na(ds1_avgdrinksday)))

# Study 2
ds2_avgdrinksday <- subset(ds2, select = c(avgdrinksday))
ds2_avgdrinksday <- na.omit(ds2_avgdrinksday)
ds2_avgdrinksday <- data.frame(mean = apply(ds2_avgdrinksday, 2, mean),
sd = apply(ds2_avgdrinksday, 2, sd), n = colSums(!is.na(ds2_avgdrinksday)))

# Combined
dboth_avgdrinksday <- rbind(ds1_avgdrinksday, ds2_avgdrinksday)
rownames(dboth_avgdrinksday) <- c("Study 1", "Study 2")
dboth_avgdrinksday

##              mean       sd   n
## Study 1 4.818182 4.252064 319
## Study 2 4.738977 3.285413 567

# Welch Two Sample t-test
(dboth_avgdrinksday <- t.test(avgdrinksday ~ study, data=dboth))

##
##  Welch Two Sample t-test
##
## data:  avgdrinksday by study
## t = 0.28785, df = 533.67, p-value = 0.7736
## alternative hypothesis: true difference in means is not equal to 0
## 95 percent confidence interval:
##  -0.4613290  0.6197385
## sample estimates:
## mean in group 1 mean in group 2
##        4.818182        4.738977
```

Heavy drinking occasions

```
# Study 1
ds1_heavydrink <- subset(ds1, select = c(heavydrink))
ds1_heavydrink <- na.omit(ds1_heavydrink)
ds1_heavydrink <- data.frame(mean = apply(ds1_heavydrink, 2, mean), sd =
apply(ds1_heavydrink, 2, sd), n = colSums(!is.na(ds1_heavydrink)))

# Study 2
ds2_heavydrink <- subset(ds2, select = c(heavydrink))
ds2_heavydrink <- na.omit(ds2_heavydrink)
ds2_heavydrink <- data.frame(mean = apply(ds2_heavydrink, 2, mean), sd =
apply(ds2_heavydrink, 2, sd), n = colSums(!is.na(ds2_heavydrink)))

# Combined
dboth_heavydrink <- rbind(ds1_heavydrink, ds2_heavydrink)
rownames(dboth_heavydrink) <- c("Study 1", "Study 2")
dboth_heavydrink

##              mean       sd   n
## Study 1 4.637500 6.192307 320
## Study 2 5.784832 7.160422 567

# Welch Two Sample t-test
(dboth_heavydrink <- t.test(heavydrink ~ study, data=dboth))

##
##  Welch Two Sample t-test
##
## data:  heavydrink by study
## t = -2.5022, df = 743.49, p-value = 0.01256
## alternative hypothesis: true difference in means is not equal to 0
## 95 percent confidence interval:
##  -2.0475076 -0.2471573
## sample estimates:
## mean in group 1 mean in group 2
##        4.637500        5.784832
```

Peak drinks on a drinking day

```
# Study 1
ds1_peakdrink <- subset(ds1, select = c(peakdrink))
ds1_peakdrink <- na.omit(ds1_peakdrink)
ds1_peakdrink <- data.frame(mean = apply(ds1_peakdrink, 2, mean), sd =
apply(ds1_peakdrink, 2, sd), n = colSums(!is.na(ds1_peakdrink)))

# Study 2
ds2_peakdrink <- subset(ds2, select = c(peakdrink))
ds2_peakdrink <- na.omit(ds2_peakdrink)
ds2_peakdrink <- data.frame(mean = apply(ds2_peakdrink, 2, mean), sd =
```

```
apply(ds2_peakdrink, 2, sd), n = colSums(!is.na(ds2_peakdrink)))

# Combined
dboth_peakdrink <- rbind(ds1_peakdrink, ds2_peakdrink)
rownames(dboth_peakdrink) <- c("Study 1", "Study 2")
dboth_peakdrink

##             mean       sd   n
## Study 1 8.479495 5.955828 317
## Study 2 9.389771 6.044719 567

# Welch Two Sample t-test
(dboth_peakdrink <- t.test(peakdrink ~ study, data=dboth))

## 
##  Welch Two Sample t-test
## 
## data:  peakdrink by study
## t = -2.1677, df = 662.16, p-value = 0.03054
## alternative hypothesis: true difference in means is not equal to 0
## 95 percent confidence interval:
##  -1.73482915 -0.08572176
## sample estimates:
## mean in group 1 mean in group 2 
##        8.479495        9.389771
```

Alcohol consequences

```
# Study 1
ds1_BYAACQ <- subset(ds1, select = c(BYAACQ))
ds1_BYAACQ <- na.omit(ds1_BYAACQ)
ds1_BYAACQ <- data.frame(mean = apply(ds1_BYAACQ, 2, mean), sd =
apply(ds1_BYAACQ, 2, sd), n = colSums(!is.na(ds1_BYAACQ)))

# Study 2
ds2_BYAACQ <- subset(ds2, select = c(BYAACQ))
ds2_BYAACQ <- na.omit(ds2_BYAACQ)
ds2_BYAACQ <- data.frame(mean = apply(ds2_BYAACQ, 2, mean), sd =
apply(ds2_BYAACQ, 2, sd), n = colSums(!is.na(ds2_BYAACQ)))

# Combined
dboth_BYAACQ <- rbind(ds1_BYAACQ, ds2_BYAACQ)
rownames(dboth_BYAACQ) <- c("Study 1", "Study 2")
dboth_BYAACQ

##             mean       sd   n
## Study 1 7.818750 7.128355 320
## Study 2 7.546392 6.790731 582
```

```
# Welch Two Sample t-test
(dboth_BYAACQ <- t.test(BYAACQ ~ study, data=dboth))
```

```
##
##  Welch Two Sample t-test
##
## data:  BYAACQ by study
## t = 0.55825, df = 630.57, p-value = 0.5769
## alternative hypothesis: true difference in means is not equal to 0
## 95 percent confidence interval:
##  -0.6857055  1.2304220
## sample estimates:
## mean in group 1 mean in group 2
##        7.818750        7.546392
```

Any cannabis use in past 6 months

```
# Percentages
attach(dboth)
dboth_anycannabis <- table(study,anycannabis)
colnames(dboth_anycannabis) <- c("No", "Yes")
prop.table(dboth_anycannabis, 1)
```

```
##      anycannabis
## study        No       Yes
##     1 0.5810277 0.4189723
##     2 0.7096220 0.2903780
```

```
# Sample Sizes
margin.table(dboth_anycannabis, 1)
```

```
## study
##   1   2
## 253 582
```

```
# Chi-squared test
chisq.test(dboth_anycannabis)
```

```
##
##  Pearson's Chi-squared test with Yates' continuity correction
##
## data:  dboth_anycannabis
## X-squared = 12.627, df = 1, p-value = 0.0003803
```

Total cannabis use days in past 30 days

```
# Study 1
ds1_totcannabis <- subset(ds1, select = c(totcannabis))
ds1_totcannabis <- na.omit(ds1_totcannabis)
```

```r
ds1_totcannabis <- data.frame(mean = apply(ds1_totcannabis, 2, mean), sd
= apply(ds1_totcannabis, 2, sd), n = colSums(!is.na(ds1_totcannabis)))

# Study 2
ds2_totcannabis <- subset(ds2, select = c(totcannabis))
ds2_totcannabis <- na.omit(ds2_totcannabis)
ds2_totcannabis <- data.frame(mean = apply(ds2_totcannabis, 2, mean), sd
= apply(ds2_totcannabis, 2, sd), n = colSums(!is.na(ds2_totcannabis)))

# Combined
dboth_totcannabis <- rbind(ds1_totcannabis, ds2_totcannabis)
rownames(dboth_totcannabis) <- c("Study 1", "Study 2")
dboth_totcannabis

##             mean        sd   n
## Study 1 9.872093 11.428341  86
## Study 2 3.347079  8.584288 582

# Welch Two Sample t-test
(dboth_totcannabis <- t.test(totcannabis ~ study, data=dboth))

##
##  Welch Two Sample t-test
##
## data:  totcannabis by study
## t = 5.087, df = 99.663, p-value = 1.713e-06
## alternative hypothesis: true difference in means is not equal to 0
## 95 percent confidence interval:
##  3.980082 9.069946
## sample estimates:
## mean in group 1 mean in group 2
##        9.872093        3.347079
```

Care Receipt

Behavioral Health - Discharge

```r
# Percentages
attach(dboth)
dboth_anydis <- table(study,anydis)
colnames(dboth_anydis) <- c("No", "Yes")
prop.table(dboth_anydis, 1)

##      anydis
## study        No       Yes
##     1 0.4771429 0.5228571
##     2 0.4192440 0.5807560
```

```
# Sample Sizes
margin.table(dboth_anydis, 1)

## study
##   1   2
## 350 582

# Chi-squared test
chisq.test(dboth_anydis)

##
##  Pearson's Chi-squared test with Yates' continuity correction
##
## data:  dboth_anydis
## X-squared = 2.7419, df = 1, p-value = 0.09775
```

Behavioral Health - Year

```
# Percentages
attach(dboth)
dboth_anyyear <- table(study,anyyear)
colnames(dboth_anyyear) <- c("No", "Yes")
prop.table(dboth_anyyear, 1)

##      anyyear
## study        No       Yes
##     1 0.6600000 0.3400000
##     2 0.5910653 0.4089347

# Sample Sizes
margin.table(dboth_anyyear, 1)

## study
##   1   2
## 350 582

# Chi-squared test
chisq.test(dboth_anyyear)

##
##  Pearson's Chi-squared test with Yates' continuity correction
##
## data:  dboth_anyyear
## X-squared = 4.108, df = 1, p-value = 0.04268
```

Behavioral Health - Month

```
# Percentages
attach(dboth)
dboth_anymonth <- table(study,anymonth)
```

```
colnames(dboth_anymonth) <- c("No", "Yes")
prop.table(dboth_anymonth, 1)

##      anymonth
## study        No       Yes
##     1 0.8800000 0.1200000
##     2 0.8264605 0.1735395

# Sample Sizes
margin.table(dboth_anymonth, 1)

## study
##   1   2
## 350 582

# Chi-squared test
chisq.test(dboth_anymonth)

##
##  Pearson's Chi-squared test with Yates' continuity correction
##
## data:  dboth_anymonth
## X-squared = 4.4199, df = 1, p-value = 0.03552
```

Mental Health - Discharge

```
# Percentages
attach(dboth)
dboth_mhdis <- table(study,mhdis)
colnames(dboth_mhdis) <- c("No", "Yes")
prop.table(dboth_mhdis, 1)

##      mhdis
## study        No       Yes
##     1 0.5000000 0.5000000
##     2 0.4501718 0.5498282

# Sample Sizes
margin.table(dboth_mhdis, 1)

## study
##   1   2
## 350 582

# Chi-squared test
chisq.test(dboth_mhdis)

##
##  Pearson's Chi-squared test with Yates' continuity correction
##
```

```
## data:  dboth_mhdis
## X-squared = 1.9836, df = 1, p-value = 0.159
```

Mental Health - Year

```
# Percentages
attach(dboth)
dboth_mhyear <- table(study,mhyear)
colnames(dboth_mhyear) <- c("No", "Yes")
prop.table(dboth_mhyear, 1)

##      mhyear
## study        No       Yes
##     1 0.6685714 0.3314286
##     2 0.6082474 0.3917526

# Sample Sizes
margin.table(dboth_mhyear, 1)

## study
##   1   2
## 350 582

# Chi-squared test
chisq.test(dboth_mhyear)

##
##  Pearson's Chi-squared test with Yates' continuity correction
##
## data:  dboth_mhyear
## X-squared = 3.1613, df = 1, p-value = 0.0754
```

Mental Health - Month

```
# Percentages
attach(dboth)
dboth_mhmonth <- table(study,mhmonth)
colnames(dboth_mhmonth) <- c("No", "Yes")
prop.table(dboth_mhmonth, 1)

##      mhmonth
## study        No       Yes
##     1 0.8800000 0.1200000
##     2 0.8316151 0.1683849

# Sample Sizes
margin.table(dboth_mhmonth, 1)
```

```
## study
##   1   2
## 350 582
```

```r
# Chi-squared test
chisq.test(dboth_mhmonth)
```

```
##
##  Pearson's Chi-squared test with Yates' continuity correction
##
## data:  dboth_mhmonth
## X-squared = 3.6383, df = 1, p-value = 0.05646
```

Substance Use - Discharge

```r
# Percentages
attach(dboth)
dboth_alcsuddis <- table(study,alcsuddis)
colnames(dboth_alcsuddis) <- c("No", "Yes")
prop.table(dboth_alcsuddis, 1)
```

```
##      alcsuddis
## study        No       Yes
##     1 0.8085714 0.1914286
##     2 0.7920962 0.2079038
```

```r
# Sample Sizes
margin.table(dboth_alcsuddis, 1)
```

```
## study
##   1   2
## 350 582
```

```r
# Chi-squared test
chisq.test(dboth_alcsuddis)
```

```
##
##  Pearson's Chi-squared test with Yates' continuity correction
##
## data:  dboth_alcsuddis
## X-squared = 0.27321, df = 1, p-value = 0.6012
```

Substance Use - Year

```r
# Percentages
attach(dboth)
dboth_alcsudyear <- table(study,alcsudyear)
colnames(dboth_alcsudyear) <- c("No", "Yes")
prop.table(dboth_alcsudyear, 1)
```

```
##      alcsudyear
## study         No        Yes
##     1 0.91142857 0.08857143
##     2 0.88659794 0.11340206

# Sample Sizes
margin.table(dboth_alcsudyear, 1)

## study
##   1   2
## 350 582

# Chi-squared test
chisq.test(dboth_alcsudyear)

##
##  Pearson's Chi-squared test with Yates' continuity correction
##
## data:  dboth_alcsudyear
## X-squared = 1.1912, df = 1, p-value = 0.2751
```

Substance Use - Month

```
# Percentages
attach(dboth)
dboth_alcsudmonth <- table(study,alcsudmonth)
colnames(dboth_alcsudmonth) <- c("No", "Yes")
prop.table(dboth_alcsudmonth, 1)

##      alcsudmonth
## study          No         Yes
##     1 0.997142857 0.002857143
##     2 0.969072165 0.030927835

# Sample Sizes
margin.table(dboth_alcsudmonth, 1)

## study
##   1   2
## 350 582

# Chi-squared test
chisq.test(dboth_alcsudmonth)

##
##  Pearson's Chi-squared test with Yates' continuity correction
##
## data:  dboth_alcsudmonth
## X-squared = 7.2753, df = 1, p-value = 0.006991
```

Video Game Behavior

Total hours spent playing per day

```
# Study 1
ds1_vghrsday <- subset(ds1, select = c(vghrsday))
ds1_vghrsday <- na.omit(ds1_vghrsday)
ds1_vghrsday <- data.frame(mean = apply(ds1_vghrsday, 2, mean), sd =
apply(ds1_vghrsday, 2, sd), n = colSums(!is.na(ds1_vghrsday)))

# Study 2
ds2_vghrsday <- subset(ds2, select = c(vghrsday))
ds2_vghrsday <- na.omit(ds2_vghrsday)
ds2_vghrsday <- data.frame(mean = apply(ds2_vghrsday, 2, mean), sd =
apply(ds2_vghrsday, 2, sd), n = colSums(!is.na(ds2_vghrsday)))

# Combined
dboth_vghrsday <- rbind(ds1_vghrsday, ds2_vghrsday)
rownames(dboth_vghrsday) <- c("Study 1", "Study 2")
dboth_vghrsday

##              mean      sd   n
## Study 1 2.311429 1.80659 350
## Study 2 3.541237 3.24939 582

# Welch Two Sample t-test
(dboth_vghrsday <- t.test(vghrsday ~ study, data=dboth))

##
##  Welch Two Sample t-test
##
## data:  vghrsday by study
## t = -7.4205, df = 924.95, p-value = 2.641e-13
## alternative hypothesis: true difference in means is not equal to 0
## 95 percent confidence interval:
##  -1.5550615 -0.9045556
## sample estimates:
## mean in group 1 mean in group 2
##        2.311429        3.541237
```

Total hours spent playing per week

```
# Study 1
ds1_vghrsweek <- subset(ds1, select = c(vghrsweek))
ds1_vghrsweek <- na.omit(ds1_vghrsweek)
ds1_vghrsweek <- data.frame(mean = apply(ds1_vghrsweek, 2, mean), sd =
apply(ds1_vghrsweek, 2, sd), n = colSums(!is.na(ds1_vghrsweek)))

# Study 2
```

```r
ds2_vghrsweek <- subset(ds2, select = c(vghrsweek))
ds2_vghrsweek <- na.omit(ds2_vghrsweek)
ds2_vghrsweek <- data.frame(mean = apply(ds2_vghrsweek, 2, mean), sd =
apply(ds2_vghrsweek, 2, sd), n = colSums(!is.na(ds2_vghrsweek)))

# Combined
dboth_vghrsweek <- rbind(ds1_vghrsweek, ds2_vghrsweek)
rownames(dboth_vghrsweek) <- c("Study 1", "Study 2")
dboth_vghrsweek
```

```
##             mean       sd   n
## Study 1 12.80857 13.50625 350
## Study 2 18.36426 21.89675 582
```

```r
# Welch Two Sample t-test
(dboth_vghrsweek <- t.test(vghrsweek ~ study, data=dboth))
```

```
##
##  Welch Two Sample t-test
##
## data:  vghrsweek by study
## t = -4.7904, df = 929.41, p-value = 1.936e-06
## alternative hypothesis: true difference in means is not equal to 0
## 95 percent confidence interval:
##  -7.831726 -3.279654
## sample estimates:
## mean in group 1 mean in group 2
##        12.80857        18.36426
```

Total days spent playing per week

```r
# Study 1
ds1_vgdaysweek <- subset(ds1, select = c(vgdaysweek))
ds1_vgdaysweek <- na.omit(ds1_vgdaysweek)
ds1_vgdaysweek <- data.frame(mean = apply(ds1_vgdaysweek, 2, mean), sd
= apply(ds1_vgdaysweek, 2, sd), n = colSums(!is.na(ds1_vgdaysweek)))

# Study 2
ds2_vgdaysweek <- subset(ds2, select = c(vgdaysweek))
ds2_vgdaysweek <- na.omit(ds2_vgdaysweek)
ds2_vgdaysweek <- data.frame(mean = apply(ds2_vgdaysweek, 2, mean), sd
= apply(ds2_vgdaysweek, 2, sd), n = colSums(!is.na(ds2_vgdaysweek)))

# Combined
dboth_vgdaysweek <- rbind(ds1_vgdaysweek, ds2_vgdaysweek)
rownames(dboth_vgdaysweek) <- c("Study 1", "Study 2")
dboth_vgdaysweek
```

```
##             mean       sd   n
## Study 1 5.028571 2.283436 350
## Study 2 4.723368 2.214202 582
```

```
# Welch Two Sample t-test
(dboth_vgdaysweek <- t.test(vgdaysweek ~ study, data=dboth))
```

```
##
##  Welch Two Sample t-test
##
## data:  vgdaysweek by study
## t = 1.9985, df = 717.48, p-value = 0.04603
## alternative hypothesis: true difference in means is not equal to 0
## 95 percent confidence interval:
##  0.005386123 0.605021339
## sample estimates:
## mean in group 1 mean in group 2
##        5.028571        4.723368
```

Any positive screen

Replicated Effect

Hours per day

Study 1

```
(vghrsday_anyscreen_ds1 <- t.test(vghrsday ~ anyscreen, data=ds1))
```

```
##
##  Welch Two Sample t-test
##
## data:  vghrsday by anyscreen
## t = -1.1898, df = 137.56, p-value = 0.2362
## alternative hypothesis: true difference in means is not equal to 0
## 95 percent confidence interval:
##  -0.7367650  0.1832053
## sample estimates:
## mean in group 0 mean in group 1
##        2.103448        2.380228
```

Study 2

```
(vghrsday_anyscreen_ds2 <- t.test(vghrsday ~ anyscreen, data=ds2))
```

```
##
##  Welch Two Sample t-test
##
```

```
## data:  vghrsday by anyscreen
## t = -1.3004, df = 260.62, p-value = 0.1946
## alternative hypothesis: true difference in means is not equal to 0
## 95 percent confidence interval:
##  -0.947307  0.193740
## sample estimates:
## mean in group 0 mean in group 1
##        3.251852        3.628635
```

Hours per week

Study 1

```
(vghrsweek_anyscreen_ds1 <- t.test(vghrsweek ~ anyscreen, data=ds1))
```

```
##
##  Welch Two Sample t-test
##
## data:  vghrsweek by anyscreen
## t = -1.4503, df = 142.17, p-value = 0.1492
## alternative hypothesis: true difference in means is not equal to 0
## 95 percent confidence interval:
##  -5.8319900  0.8959295
## sample estimates:
## mean in group 0 mean in group 1
##        10.95402        13.42205
```

Study 2

```
(vghrsweek_anyscreen_ds2 <- t.test(vghrsweek ~ anyscreen, data=ds2))
```

```
##
##  Welch Two Sample t-test
##
## data:  vghrsweek by anyscreen
## t = -1.4505, df = 270.15, p-value = 0.1481
## alternative hypothesis: true difference in means is not equal to 0
## 95 percent confidence interval:
##  -6.5517454  0.9931076
## sample estimates:
## mean in group 0 mean in group 1
##        16.22963        19.00895
```

Days per week

Study 1

```
(vgdaysweek_anyscreen_ds1 <- t.test(vgdaysweek ~ anyscreen, data=ds1))
```

```
##
##  Welch Two Sample t-test
##
## data:  vgdaysweek by anyscreen
## t = -1.799, df = 137.54, p-value = 0.07421
## alternative hypothesis: true difference in means is not equal to 0
## 95 percent confidence interval:
##  -1.10732457  0.05230076
## sample estimates:
## mean in group 0 mean in group 1
##        4.632184        5.159696
```

Study 2

```
(vgdaysweek_anyscreen_ds2 <- t.test(vgdaysweek ~ anyscreen, data=ds2))
```

```
##
##  Welch Two Sample t-test
##
## data:  vgdaysweek by anyscreen
## t = -0.90454, df = 216.96, p-value = 0.3667
## alternative hypothesis: true difference in means is not equal to 0
## 95 percent confidence interval:
##  -0.6332657  0.2348565
## sample estimates:
## mean in group 0 mean in group 1
##        4.570370        4.769575
```

MD

Hours per day

```
#Study 1
anyscreen_yes_ds1_vghrsday <- subset(anyscreen_yes_ds1, select =
c(vghrsday)) #Subset 1i data
anyscreen_vghrsday_m1i <- apply(anyscreen_yes_ds1_vghrsday, 2, mean)
#Calculate m1i
anyscreen_vghrsday_sd1i <- apply(anyscreen_yes_ds1_vghrsday, 2, sd)
#Calculate sd1i
anyscreen_vghrsday_n1i <- colSums(!is.na(anyscreen_yes_ds1_vghrsday))
#Calculate n1i
anyscreen_no_ds1_vghrsday <- subset(anyscreen_no_ds1, select =
c(vghrsday)) #Subset 2i data
anyscreen_vghrsday_m2i <- apply(anyscreen_no_ds1_vghrsday, 2, mean)
#Calculate m2i
anyscreen_vghrsday_sd2i <- apply(anyscreen_no_ds1_vghrsday, 2, sd)
#Calculate sd2i
anyscreen_vghrsday_n2i <- colSums(!is.na(anyscreen_no_ds1_vghrsday))
```

```
#Calculate n2i
ds1anyscreen_meta <- data.frame(m1i = anyscreen_vghrsday_m1i, sd1i =
anyscreen_vghrsday_sd1i, n1i = anyscreen_vghrsday_n1i, m2i =
anyscreen_vghrsday_m2i, sd2i = anyscreen_vghrsday_sd2i, n2i =
anyscreen_vghrsday_n2i) #MA dataframe
ds1anyscreen_meta <- escalc(measure="MD", m1i = m1i, sd1i = sd1i, n1i =
n1i, m2i = m2i, sd2i = sd2i, n2i = n2i, data=ds1anyscreen_meta) #Calculate
yi and vi

#Study 2
anyscreen_yes_ds2_vghrsday <- subset(anyscreen_yes_ds2, select =
c(vghrsday)) #Subset 1i data
anyscreen_vghrsday_m1i <- apply(anyscreen_yes_ds2_vghrsday, 2, mean)
#Calculate m1i
anyscreen_vghrsday_sd1i <- apply(anyscreen_yes_ds2_vghrsday, 2, sd)
#Calculate sd1i
anyscreen_vghrsday_n1i <- colSums(!is.na(anyscreen_yes_ds2_vghrsday))
#Calculate n1i
anyscreen_no_ds2_vghrsday <- subset(anyscreen_no_ds2, select =
c(vghrsday)) #Subset 2i data
anyscreen_vghrsday_m2i <- apply(anyscreen_no_ds2_vghrsday, 2, mean)
#Calculate m2i
anyscreen_vghrsday_sd2i <- apply(anyscreen_no_ds2_vghrsday, 2, sd)
#Calculate sd2i
anyscreen_vghrsday_n2i <- colSums(!is.na(anyscreen_no_ds2_vghrsday))
#Calculate n2i
ds2anyscreen_meta <- data.frame(m1i = anyscreen_vghrsday_m1i, sd1i =
anyscreen_vghrsday_sd1i, n1i = anyscreen_vghrsday_n1i, m2i =
anyscreen_vghrsday_m2i, sd2i = anyscreen_vghrsday_sd2i, n2i =
anyscreen_vghrsday_n2i) #MA dataframe
ds2anyscreen_meta <- escalc(measure="MD", m1i = m1i, sd1i = sd1i, n1i =
n1i, m2i = m2i, sd2i = sd2i, n2i = n2i, data=ds2anyscreen_meta) #Calculate
yi and vi

#FE meta-analysis
(anyscreen_meta <- rbind(ds1anyscreen_meta, ds2anyscreen_meta))

##        m1i     sd1i n1i      m2i     sd2i n2i     yi     vi
## 1 2.380228 1.766932 263 2.103448 1.917086  87 0.2768 0.0541
## 2 3.628635 3.368395 447 3.251852 2.811805 135 0.3768 0.0839

(any_vghrsday_meta <- rma(yi=yi, vi=vi, measure="MD", method="FE",
data=anyscreen_meta))

##
## Fixed-Effects Model (k = 2)
##
## Test for Heterogeneity:
## Q(df = 1) = 0.0724, p-val = 0.7878
##
## Model Results:
```

```
##
## estimate      se    zval    pval   ci.lb   ci.ub
##   0.3160  0.1814  1.7419  0.0815  -0.0395  0.6715      .
##
## ---
## Signif. codes:  0 '***' 0.001 '**' 0.01 '*' 0.05 '.' 0.1 ' ' 1
```

Hours per week

```
#Study 1
anyscreen_yes_ds1_vghrsweek <- subset(anyscreen_yes_ds1, select =
c(vghrsweek)) #Subset 1i data
anyscreen_vghrsweek_m1i <- apply(anyscreen_yes_ds1_vghrsweek, 2, mean)
#Calculate m1i
anyscreen_vghrsweek_sd1i <- apply(anyscreen_yes_ds1_vghrsweek, 2, sd)
#Calculate sd1i
anyscreen_vghrsweek_n1i <- colSums(!is.na(anyscreen_yes_ds1_vghrsweek))
#Calculate n1i
anyscreen_no_ds1_vghrsweek <- subset(anyscreen_no_ds1, select =
c(vghrsweek)) #Subset 2i data
anyscreen_vghrsweek_m2i <- apply(anyscreen_no_ds1_vghrsweek, 2, mean)
#Calculate m2i
anyscreen_vghrsweek_sd2i <- apply(anyscreen_no_ds1_vghrsweek, 2, sd)
#Calculate sd2i
anyscreen_vghrsweek_n2i <- colSums(!is.na(anyscreen_no_ds1_vghrsweek))
#Calculate n2i
ds1anyscreen_meta <- data.frame(m1i = anyscreen_vghrsweek_m1i, sd1i =
anyscreen_vghrsweek_sd1i, n1i = anyscreen_vghrsweek_n1i, m2i =
anyscreen_vghrsweek_m2i, sd2i = anyscreen_vghrsweek_sd2i, n2i =
anyscreen_vghrsweek_n2i) #MA dataframe
ds1anyscreen_meta <- escalc(measure="MD", m1i = m1i, sd1i = sd1i, n1i =
n1i, m2i = m2i, sd2i = sd2i, n2i = n2i, data=ds1anyscreen_meta) #Calculate
yi and vi

#Study 2
anyscreen_yes_ds2_vghrsweek <- subset(anyscreen_yes_ds2, select =
c(vghrsweek)) #Subset 1i data
anyscreen_vghrsweek_m1i <- apply(anyscreen_yes_ds2_vghrsweek, 2, mean)
#Calculate m1i
anyscreen_vghrsweek_sd1i <- apply(anyscreen_yes_ds2_vghrsweek, 2, sd)
#Calculate sd1i
anyscreen_vghrsweek_n1i <- colSums(!is.na(anyscreen_yes_ds2_vghrsweek))
#Calculate n1i
anyscreen_no_ds2_vghrsweek <- subset(anyscreen_no_ds2, select =
c(vghrsweek)) #Subset 2i data
anyscreen_vghrsweek_m2i <- apply(anyscreen_no_ds2_vghrsweek, 2, mean)
#Calculate m2i
anyscreen_vghrsweek_sd2i <- apply(anyscreen_no_ds2_vghrsweek, 2, sd)
#Calculate sd2i
```

```
anyscreen_vghrsweek_n2i <- colSums(!is.na(anyscreen_no_ds2_vghrsweek))
#Calculate n2i
ds2anyscreen_meta <- data.frame(m1i = anyscreen_vghrsweek_m1i, sd1i =
anyscreen_vghrsweek_sd1i, n1i = anyscreen_vghrsweek_n1i, m2i =
anyscreen_vghrsweek_m2i, sd2i = anyscreen_vghrsweek_sd2i, n2i =
anyscreen_vghrsweek_n2i) #MA dataframe
ds2anyscreen_meta <- escalc(measure="MD", m1i = m1i, sd1i = sd1i, n1i =
n1i, m2i = m2i, sd2i = sd2i, n2i = n2i, data=ds2anyscreen_meta) #Calculate
yi and vi

#FE meta-analysis
(anyscreen_meta <- rbind(ds1anyscreen_meta, ds2anyscreen_meta))
```

```
##        m1i     sd1i n1i      m2i     sd2i n2i     yi     vi
## 1 13.42205 13.34598 263 10.95402 13.89320  87 2.4680 2.8959
## 2 19.00895 22.82925 447 16.22963 18.39165 135 2.7793 3.6715
```

```
(any_vghrsweek_meta <- rma(yi=yi, vi=vi, measure="MD", method="FE",
data=anyscreen_meta))
```

```
##
## Fixed-Effects Model (k = 2)
##
## Test for Heterogeneity:
## Q(df = 1) = 0.0148, p-val = 0.9033
##
## Model Results:
##
## estimate      se     zval     pval    ci.lb    ci.ub
##   2.6053   1.2724   2.0476   0.0406   0.1115   5.0991       *
##
## ---
## Signif. codes:  0 '***' 0.001 '**' 0.01 '*' 0.05 '.' 0.1 ' ' 1
```

Days per week

```
#Study 1
anyscreen_yes_ds1_vgdaysweek <- subset(anyscreen_yes_ds1, select =
c(vgdaysweek)) #Subset 1i data
anyscreen_vgdaysweek_m1i <- apply(anyscreen_yes_ds1_vgdaysweek, 2,
mean) #Calculate m1i
anyscreen_vgdaysweek_sd1i <- apply(anyscreen_yes_ds1_vgdaysweek, 2,
sd) #Calculate sd1i
anyscreen_vgdaysweek_n1i <- colSums(!
is.na(anyscreen_yes_ds1_vgdaysweek)) #Calculate n1i
anyscreen_no_ds1_vgdaysweek <- subset(anyscreen_no_ds1, select =
c(vgdaysweek)) #Subset 2i data
anyscreen_vgdaysweek_m2i <- apply(anyscreen_no_ds1_vgdaysweek, 2,
mean) #Calculate m2i
anyscreen_vgdaysweek_sd2i <- apply(anyscreen_no_ds1_vgdaysweek, 2, sd)
```

```
#Calculate sd2i
anyscreen_vgdaysweek_n2i <- colSums(!
is.na(anyscreen_no_ds1_vgdaysweek)) #Calculate n2i
ds1anyscreen_meta <- data.frame(m1i = anyscreen_vgdaysweek_m1i, sd1i
= anyscreen_vgdaysweek_sd1i, n1i = anyscreen_vgdaysweek_n1i, m2i =
anyscreen_vgdaysweek_m2i, sd2i = anyscreen_vgdaysweek_sd2i, n2i =
anyscreen_vgdaysweek_n2i) #MA dataframe
ds1anyscreen_meta <- escalc(measure="MD", m1i = m1i, sd1i = sd1i, n1i =
n1i, m2i = m2i, sd2i = sd2i, n2i = n2i, data=ds1anyscreen_meta) #Calculate
yi and vi

#Study 2
anyscreen_yes_ds2_vgdaysweek <- subset(anyscreen_yes_ds2, select =
c(vgdaysweek)) #Subset 1i data
anyscreen_vgdaysweek_m1i <- apply(anyscreen_yes_ds2_vgdaysweek, 2,
mean) #Calculate m1i
anyscreen_vgdaysweek_sd1i <- apply(anyscreen_yes_ds2_vgdaysweek, 2,
sd) #Calculate sd1i
anyscreen_vgdaysweek_n1i <- colSums(!
is.na(anyscreen_yes_ds2_vgdaysweek)) #Calculate n1i
anyscreen_no_ds2_vgdaysweek <- subset(anyscreen_no_ds2, select =
c(vgdaysweek)) #Subset 2i data
anyscreen_vgdaysweek_m2i <- apply(anyscreen_no_ds2_vgdaysweek, 2,
mean) #Calculate m2i
anyscreen_vgdaysweek_sd2i <- apply(anyscreen_no_ds2_vgdaysweek, 2, sd)
#Calculate sd2i
anyscreen_vgdaysweek_n2i <- colSums(!
is.na(anyscreen_no_ds2_vgdaysweek)) #Calculate n2i
ds2anyscreen_meta <- data.frame(m1i = anyscreen_vgdaysweek_m1i, sd1i
= anyscreen_vgdaysweek_sd1i, n1i = anyscreen_vgdaysweek_n1i, m2i =
anyscreen_vgdaysweek_m2i, sd2i = anyscreen_vgdaysweek_sd2i, n2i =
anyscreen_vgdaysweek_n2i) #MA dataframe
ds2anyscreen_meta <- escalc(measure="MD", m1i = m1i, sd1i = sd1i, n1i =
n1i, m2i = m2i, sd2i = sd2i, n2i = n2i, data=ds2anyscreen_meta) #Calculate
yi and vi

#FE meta-analysis
(anyscreen_meta <- rbind(ds1anyscreen_meta, ds2anyscreen_meta))

##        m1i     sd1i n1i      m2i     sd2i n2i     yi     vi
## 1 5.159696 2.226911 263 4.632184 2.416585  87 0.5275 0.0860
## 2 4.769575 2.202356 447 4.570370 2.254484 135 0.1992 0.0485

(any_vgdaysweek_meta <- rma(yi=yi, vi=vi, measure="MD", method="FE",
data=anyscreen_meta))

##
## Fixed-Effects Model (k = 2)
##
## Test for Heterogeneity:
## Q(df = 1) = 0.8015, p-val = 0.3706
```

```
##
## Model Results:
##
## estimate      se     zval     pval    ci.lb    ci.ub
##   0.3176   0.1761   1.8036   0.0713  -0.0275   0.6627       .
##
## ---
## Signif. codes:  0 '***' 0.001 '**' 0.01 '*' 0.05 '.' 0.1 ' ' 1
```

Any mental health screen

Replicated Effect

Hours per day

Study 1

```
(vghrsday_anymhscreen_ds1 <- t.test(vghrsday ~ anymhscreen, data=ds1))

##
##  Welch Two Sample t-test
##
## data:  vghrsday by anymhscreen
## t = -2.9955, df = 324.01, p-value = 0.002951
## alternative hypothesis: true difference in means is not equal to 0
## 95 percent confidence interval:
##  -0.9548135 -0.1978231
## sample estimates:
## mean in group 0 mean in group 1
##        2.033149        2.609467
```

Study 2

```
(vghrsday_anymhscreen_ds2 <- t.test(vghrsday ~ anymhscreen, data=ds2))

##
##  Welch Two Sample t-test
##
## data:  vghrsday by anymhscreen
## t = -2.5118, df = 577.59, p-value = 0.01228
## alternative hypothesis: true difference in means is not equal to 0
## 95 percent confidence interval:
##  -1.1973751 -0.1465138
## sample estimates:
## mean in group 0 mean in group 1
##        3.197183        3.869128
```

Hours per week

Study 1

```
(vghrsweek_anymhscreen_ds1 <- t.test(vghrsweek ~ anymhscreen, data=ds1))

##
##  Welch Two Sample t-test
##
## data:  vghrsweek by anymhscreen
## t = -3.1385, df = 320.04, p-value = 0.001856
## alternative hypothesis: true difference in means is not equal to 0
## 95 percent confidence interval:
##  -7.340716 -1.683639
## sample estimates:
## mean in group 0 mean in group 1
##        10.62983        15.14201
```

Study 2

```
(vghrsweek_anymhscreen_ds2 <- t.test(vghrsweek ~ anymhscreen, data=ds2))

##
##  Welch Two Sample t-test
##
## data:  vghrsweek by anymhscreen
## t = -2.8329, df = 570.62, p-value = 0.004776
## alternative hypothesis: true difference in means is not equal to 0
## 95 percent confidence interval:
##  -8.622322 -1.561581
## sample estimates:
## mean in group 0 mean in group 1
##        15.75704        20.84899
```

Days per week

Study 1

```
(vgdaysweek_anymhscreen_ds1 <- t.test(vgdaysweek ~ anymhscreen, data=ds1))

##
##  Welch Two Sample t-test
##
## data:  vgdaysweek by anymhscreen
## t = -2.2251, df = 347.68, p-value = 0.02672
```

```
## alternative hypothesis: true difference in means is not equal to 0
## 95 percent confidence interval:
##  -1.01682148 -0.06265154
## sample estimates:
## mean in group 0 mean in group 1
##        4.767956        5.307692
```

Study 2

```
(vgdaysweek_anymhscreen_ds2 <- t.test(vgdaysweek ~ anymhscreen,
data=ds2))

##
##  Welch Two Sample t-test
##
## data:  vgdaysweek by anymhscreen
## t = -1.8544, df = 577.07, p-value = 0.0642
## alternative hypothesis: true difference in means is not equal to 0
## 95 percent confidence interval:
##  -0.70004822  0.02011627
## sample estimates:
## mean in group 0 mean in group 1
##        4.549296        4.889262
```

MD

Hours per day

```
#Study 1
anymhscreen_yes_ds1_vghrsday <- subset(anymhscreen_yes_ds1, select =
c(vghrsday)) #Subset 1i data
anymhscreen_vghrsday_m1i <- apply(anymhscreen_yes_ds1_vghrsday, 2,
mean) #Calculate m1i
anymhscreen_vghrsday_sd1i <- apply(anymhscreen_yes_ds1_vghrsday, 2,
sd) #Calculate sd1i
anymhscreen_vghrsday_n1i <- colSums(!
is.na(anymhscreen_yes_ds1_vghrsday)) #Calculate n1i
anymhscreen_no_ds1_vghrsday <- subset(anymhscreen_no_ds1, select =
c(vghrsday)) #Subset 2i data
anymhscreen_vghrsday_m2i <- apply(anymhscreen_no_ds1_vghrsday, 2,
mean) #Calculate m2i
anymhscreen_vghrsday_sd2i <- apply(anymhscreen_no_ds1_vghrsday, 2, sd)
#Calculate sd2i
anymhscreen_vghrsday_n2i <- colSums(!
is.na(anymhscreen_no_ds1_vghrsday)) #Calculate n2i
ds1anymhscreen_meta <- data.frame(m1i = anymhscreen_vghrsday_m1i,
sd1i = anymhscreen_vghrsday_sd1i, n1i = anymhscreen_vghrsday_n1i, m2i
= anymhscreen_vghrsday_m2i, sd2i = anymhscreen_vghrsday_sd2i, n2i =
```

```r
anymhscreen_vghrsday_n2i) #MA dataframe
ds1anymhscreen_meta <- escalc(measure="MD", m1i = m1i, sd1i = sd1i, n1i
= n1i, m2i = m2i, sd2i = sd2i, n2i = n2i, data=ds1anymhscreen_meta)
#Calculate yi and vi

#Study 2
anymhscreen_yes_ds2_vghrsday <- subset(anymhscreen_yes_ds2, select =
c(vghrsday)) #Subset 1i data
anymhscreen_vghrsday_m1i <- apply(anymhscreen_yes_ds2_vghrsday, 2,
mean) #Calculate m1i
anymhscreen_vghrsday_sd1i <- apply(anymhscreen_yes_ds2_vghrsday, 2,
sd) #Calculate sd1i
anymhscreen_vghrsday_n1i <- colSums(!
is.na(anymhscreen_yes_ds2_vghrsday)) #Calculate n1i
anymhscreen_no_ds2_vghrsday <- subset(anymhscreen_no_ds2, select =
c(vghrsday)) #Subset 2i data
anymhscreen_vghrsday_m2i <- apply(anymhscreen_no_ds2_vghrsday, 2,
mean) #Calculate m2i
anymhscreen_vghrsday_sd2i <- apply(anymhscreen_no_ds2_vghrsday, 2, sd)
#Calculate sd2i
anymhscreen_vghrsday_n2i <- colSums(!
is.na(anymhscreen_no_ds2_vghrsday)) #Calculate n2i
ds2anymhscreen_meta <- data.frame(m1i = anymhscreen_vghrsday_m1i,
sd1i = anymhscreen_vghrsday_sd1i, n1i = anymhscreen_vghrsday_n1i, m2i
= anymhscreen_vghrsday_m2i, sd2i = anymhscreen_vghrsday_sd2i, n2i =
anymhscreen_vghrsday_n2i) #MA dataframe
ds2anymhscreen_meta <- escalc(measure="MD", m1i = m1i, sd1i = sd1i, n1i
= n1i, m2i = m2i, sd2i = sd2i, n2i = n2i, data=ds2anymhscreen_meta)
#Calculate yi and vi

#FE meta-analysis
(anymhscreen_meta <- rbind(ds1anymhscreen_meta,
ds2anymhscreen_meta))

##        m1i    sd1i n1i      m2i     sd2i n2i     yi     vi
## 1 2.609467 1.96733 169 2.033149 1.598265 181 0.5763 0.0370
## 2 3.869128 3.40721 298 3.197183 3.043244 284 0.6719 0.0716

(any_vghrsday_meta <- rma(yi=yi, vi=vi, measure="MD", method="FE",
data=anymhscreen_meta))

##
## Fixed-Effects Model (k = 2)
##
## Test for Heterogeneity:
## Q(df = 1) = 0.0842, p-val = 0.7717
##
## Model Results:
##
## estimate      se     zval     pval    ci.lb    ci.ub
##   0.6089   0.1562   3.8985   <.0001   0.3028   0.9151      ***
```

```
##
## ---
## Signif. codes:  0 '***' 0.001 '**' 0.01 '*' 0.05 '.' 0.1 ' ' 1
```

Hours per week

```
#Study 1
anymhscreen_yes_ds1_vghrsweek <- subset(anymhscreen_yes_ds1, select =
c(vghrsweek)) #Subset 1i data
anymhscreen_vghrsweek_m1i <- apply(anymhscreen_yes_ds1_vghrsweek, 2,
mean) #Calculate m1i
anymhscreen_vghrsweek_sd1i <- apply(anymhscreen_yes_ds1_vghrsweek, 2,
sd) #Calculate sd1i
anymhscreen_vghrsweek_n1i <- colSums(!
is.na(anymhscreen_yes_ds1_vghrsweek)) #Calculate n1i
anymhscreen_no_ds1_vghrsweek <- subset(anymhscreen_no_ds1, select =
c(vghrsweek)) #Subset 2i data
anymhscreen_vghrsweek_m2i <- apply(anymhscreen_no_ds1_vghrsweek, 2,
mean) #Calculate m2i
anymhscreen_vghrsweek_sd2i <- apply(anymhscreen_no_ds1_vghrsweek, 2,
sd) #Calculate sd2i
anymhscreen_vghrsweek_n2i <- colSums(!
is.na(anymhscreen_no_ds1_vghrsweek)) #Calculate n2i
ds1anymhscreen_meta <- data.frame(m1i = anymhscreen_vghrsweek_m1i,
sd1i = anymhscreen_vghrsweek_sd1i, n1i = anymhscreen_vghrsweek_n1i,
m2i = anymhscreen_vghrsweek_m2i, sd2i = anymhscreen_vghrsweek_sd2i,
n2i = anymhscreen_vghrsweek_n2i) #MA dataframe
ds1anymhscreen_meta <- escalc(measure="MD", m1i = m1i, sd1i = sd1i, n1i
= n1i, m2i = m2i, sd2i = sd2i, n2i = n2i, data=ds1anymhscreen_meta)
#Calculate yi and vi

#Study 2
anymhscreen_yes_ds2_vghrsweek <- subset(anymhscreen_yes_ds2, select =
c(vghrsweek)) #Subset 1i data
anymhscreen_vghrsweek_m1i <- apply(anymhscreen_yes_ds2_vghrsweek, 2,
mean) #Calculate m1i
anymhscreen_vghrsweek_sd1i <- apply(anymhscreen_yes_ds2_vghrsweek, 2,
sd) #Calculate sd1i
anymhscreen_vghrsweek_n1i <- colSums(!
is.na(anymhscreen_yes_ds2_vghrsweek)) #Calculate n1i
anymhscreen_no_ds2_vghrsweek <- subset(anymhscreen_no_ds2, select =
c(vghrsweek)) #Subset 2i data
anymhscreen_vghrsweek_m2i <- apply(anymhscreen_no_ds2_vghrsweek, 2,
mean) #Calculate m2i
anymhscreen_vghrsweek_sd2i <- apply(anymhscreen_no_ds2_vghrsweek, 2,
sd) #Calculate sd2i
anymhscreen_vghrsweek_n2i <- colSums(!
is.na(anymhscreen_no_ds2_vghrsweek)) #Calculate n2i
ds2anymhscreen_meta <- data.frame(m1i = anymhscreen_vghrsweek_m1i,
```

```
sd1i = anymhscreen_vghrsweek_sd1i, n1i = anymhscreen_vghrsweek_n1i,
m2i = anymhscreen_vghrsweek_m2i, sd2i = anymhscreen_vghrsweek_sd2i,
n2i = anymhscreen_vghrsweek_n2i) #MA dataframe
ds2anymhscreen_meta <- escalc(measure="MD", m1i = m1i, sd1i = sd1i, n1i
= n1i, m2i = m2i, sd2i = sd2i, n2i = n2i, data=ds2anymhscreen_meta)
#Calculate yi and vi

#FE meta-analysis
(anymhscreen_meta <- rbind(ds1anymhscreen_meta,
ds2anymhscreen_meta))

##        m1i     sd1i n1i      m2i     sd2i n2i     yi     vi
## 1 15.14201 14.84014 169 10.62983 11.75826 181 4.5122 2.0670
## 2 20.84899 23.55195 298 15.75704 19.72033 284 5.0920 3.2307

(any_vghrsweek_meta <- rma(yi=yi, vi=vi, measure="MD", method="FE",
data=anymhscreen_meta))

##
## Fixed-Effects Model (k = 2)
##
## Test for Heterogeneity:
## Q(df = 1) = 0.0634, p-val = 0.8011
##
## Model Results:
##
## estimate      se     zval     pval    ci.lb    ci.ub
##   4.7384   1.1227   4.2204   <.0001   2.5379   6.9389      ***
##
## ---
## Signif. codes:  0 '***' 0.001 '**' 0.01 '*' 0.05 '.' 0.1 ' ' 1
```

Days per week

```
#Study 1
anymhscreen_yes_ds1_vgdaysweek <- subset(anymhscreen_yes_ds1, select
= c(vgdaysweek)) #Subset 1i data
anymhscreen_vgdaysweek_m1i <- apply(anymhscreen_yes_ds1_vgdaysweek,
2, mean) #Calculate m1i
anymhscreen_vgdaysweek_sd1i <-
apply(anymhscreen_yes_ds1_vgdaysweek, 2, sd) #Calculate sd1i
anymhscreen_vgdaysweek_n1i <- colSums(!
is.na(anymhscreen_yes_ds1_vgdaysweek)) #Calculate n1i
anymhscreen_no_ds1_vgdaysweek <- subset(anymhscreen_no_ds1, select =
c(vgdaysweek)) #Subset 2i data
anymhscreen_vgdaysweek_m2i <- apply(anymhscreen_no_ds1_vgdaysweek,
2, mean) #Calculate m2i
anymhscreen_vgdaysweek_sd2i <- apply(anymhscreen_no_ds1_vgdaysweek,
2, sd) #Calculate sd2i
anymhscreen_vgdaysweek_n2i <- colSums(!
```

```
is.na(anymhscreen_no_ds1_vgdaysweek)) #Calculate n2i
ds1anymhscreen_meta <- data.frame(m1i = anymhscreen_vgdaysweek_m1i,
sd1i = anymhscreen_vgdaysweek_sd1i, n1i = anymhscreen_vgdaysweek_n1i,
m2i = anymhscreen_vgdaysweek_m2i, sd2i =
anymhscreen_vgdaysweek_sd2i, n2i = anymhscreen_vgdaysweek_n2i) #MA
dataframe
ds1anymhscreen_meta <- escalc(measure="MD", m1i = m1i, sd1i = sd1i, n1i
= n1i, m2i = m2i, sd2i = sd2i, n2i = n2i, data=ds1anymhscreen_meta)
#Calculate yi and vi

#Study 2
anymhscreen_yes_ds2_vgdaysweek <- subset(anymhscreen_yes_ds2, select
= c(vgdaysweek)) #Subset 1i data
anymhscreen_vgdaysweek_m1i <- apply(anymhscreen_yes_ds2_vgdaysweek,
2, mean) #Calculate m1i
anymhscreen_vgdaysweek_sd1i <-
apply(anymhscreen_yes_ds2_vgdaysweek, 2, sd) #Calculate sd1i
anymhscreen_vgdaysweek_n1i <- colSums(!
is.na(anymhscreen_yes_ds2_vgdaysweek)) #Calculate n1i
anymhscreen_no_ds2_vgdaysweek <- subset(anymhscreen_no_ds2, select =
c(vgdaysweek)) #Subset 2i data
anymhscreen_vgdaysweek_m2i <- apply(anymhscreen_no_ds2_vgdaysweek,
2, mean) #Calculate m2i
anymhscreen_vgdaysweek_sd2i <- apply(anymhscreen_no_ds2_vgdaysweek,
2, sd) #Calculate sd2i
anymhscreen_vgdaysweek_n2i <- colSums(!
is.na(anymhscreen_no_ds2_vgdaysweek)) #Calculate n2i
ds2anymhscreen_meta <- data.frame(m1i = anymhscreen_vgdaysweek_m1i,
sd1i = anymhscreen_vgdaysweek_sd1i, n1i = anymhscreen_vgdaysweek_n1i,
m2i = anymhscreen_vgdaysweek_m2i, sd2i =
anymhscreen_vgdaysweek_sd2i, n2i = anymhscreen_vgdaysweek_n2i) #MA
dataframe
ds2anymhscreen_meta <- escalc(measure="MD", m1i = m1i, sd1i = sd1i, n1i
= n1i, m2i = m2i, sd2i = sd2i, n2i = n2i, data=ds2anymhscreen_meta)
#Calculate yi and vi

#FE meta-analysis
(anymhscreen_meta <- rbind(ds1anymhscreen_meta,
ds2anymhscreen_meta))

##        m1i     sd1i n1i      m2i     sd2i n2i     yi     vi
## 1 5.307692 2.225395 169 4.767956 2.312111 181 0.5397 0.0588
## 2 4.889262 2.184538 298 4.549296 2.235523 284 0.3400 0.0336

(any_vgdaysweek_meta <- rma(yi=yi, vi=vi, measure="MD", method="FE",
data=anymhscreen_meta))

##
## Fixed-Effects Model (k = 2)
##
## Test for Heterogeneity:
```

```
## Q(df = 1) = 0.4317, p-val = 0.5112
##
## Model Results:
##
## estimate      se     zval     pval    ci.lb    ci.ub
##   0.4126  0.1463  2.8210  0.0048  0.1259  0.6993     **
##
## ---
## Signif. codes:  0 '***' 0.001 '**' 0.01 '*' 0.05 '.' 0.1 ' ' 1
```

PTSD screen

Replicated Effect

Hours per day

Study 1

```
(vghrsday_ptsd_ds1 <- t.test(vghrsday ~ ptsd, data=ds1))

##
##  Welch Two Sample t-test
##
## data:  vghrsday by ptsd
## t = -2.252, df = 280.54, p-value = 0.02509
## alternative hypothesis: true difference in means is not equal to 0
## 95 percent confidence interval:
##  -0.84264162 -0.05661409
## sample estimates:
## mean in group 0 mean in group 1
##        2.116162        2.565789
```

Study 2

```
(vghrsday_ptsd_ds2 <- t.test(vghrsday ~ ptsd, data=ds2))

##
##  Welch Two Sample t-test
##
## data:  vghrsday by ptsd
## t = -2.9187, df = 394.62, p-value = 0.003717
## alternative hypothesis: true difference in means is not equal to 0
## 95 percent confidence interval:
##  -1.4158620 -0.2761402
## sample estimates:
## mean in group 0 mean in group 1
##        3.211268        4.057269
```

Hours per week

Study 1

```
(vghrsweek_ptsd_ds1 <- t.test(vghrsweek ~ ptsd, data=ds1))

##
##  Welch Two Sample t-test
##
## data:  vghrsweek by ptsd
## t = -2.2043, df = 274.48, p-value = 0.02833
## alternative hypothesis: true difference in means is not equal to 0
## 95 percent confidence interval:
##  -6.2545188 -0.3532696
## sample estimates:
## mean in group 0 mean in group 1
##        11.37374        14.67763
```

Study 2

```
(vghrsweek_ptsd_ds2 <- t.test(vghrsweek ~ ptsd, data=ds2))

##
##  Welch Two Sample t-test
##
## data:  vghrsweek by ptsd
## t = -3.5232, df = 376.41, p-value = 0.0004787
## alternative hypothesis: true difference in means is not equal to 0
## 95 percent confidence interval:
##  -10.817470  -3.068092
## sample estimates:
## mean in group 0 mean in group 1
##        15.65634        22.59912
```

Days per week

Study 1

```
(vgdaysweek_ptsd_ds1 <- t.test(vgdaysweek ~ ptsd, data=ds1))

##
##  Welch Two Sample t-test
##
## data:  vgdaysweek by ptsd
## t = -0.92919, df = 326.33, p-value = 0.3535
## alternative hypothesis: true difference in means is not equal to 0
## 95 percent confidence interval:
##  -0.7125949  0.2553912
```

```
## sample estimates:
## mean in group 0 mean in group 1
##        4.929293        5.157895
```

Study 2

```
(vgdaysweek_ptsd_ds2 <- t.test(vgdaysweek ~ ptsd, data=ds2))

##
##  Welch Two Sample t-test
##
## data:  vgdaysweek by ptsd
## t = -2.6387, df = 495.96, p-value = 0.008584
## alternative hypothesis: true difference in means is not equal to 0
## 95 percent confidence interval:
##  -0.8542093 -0.1250549
## sample estimates:
## mean in group 0 mean in group 1
##        4.532394        5.022026
```

MD

Hours per day

```
#Study 1
ptsd_yes_ds1_vghrsday <- subset(ptsd_yes_ds1, select = c(vghrsday))
#Subset 1i data
ptsd_vghrsday_m1i <- apply(ptsd_yes_ds1_vghrsday, 2, mean) #Calculate
m1i
ptsd_vghrsday_sd1i <- apply(ptsd_yes_ds1_vghrsday, 2, sd) #Calculate sd1i
ptsd_vghrsday_n1i <- colSums(!is.na(ptsd_yes_ds1_vghrsday)) #Calculate
n1i
ptsd_no_ds1_vghrsday <- subset(ptsd_no_ds1, select = c(vghrsday)) #Subset
2i data
ptsd_vghrsday_m2i <- apply(ptsd_no_ds1_vghrsday, 2, mean) #Calculate
m2i
ptsd_vghrsday_sd2i <- apply(ptsd_no_ds1_vghrsday, 2, sd) #Calculate sd2i
ptsd_vghrsday_n2i <- colSums(!is.na(ptsd_no_ds1_vghrsday)) #Calculate n2i
ds1ptsd_meta <- data.frame(m1i = ptsd_vghrsday_m1i, sd1i =
ptsd_vghrsday_sd1i, n1i = ptsd_vghrsday_n1i, m2i = ptsd_vghrsday_m2i,
sd2i = ptsd_vghrsday_sd2i, n2i = ptsd_vghrsday_n2i) #MA dataframe
ds1ptsd_meta <- escalc(measure="MD", m1i = m1i, sd1i = sd1i, n1i = n1i,
m2i = m2i, sd2i = sd2i, n2i = n2i, data=ds1ptsd_meta) #Calculate yi and vi

#Study 2
ptsd_yes_ds2_vghrsday <- subset(ptsd_yes_ds2, select = c(vghrsday))
#Subset 1i data
ptsd_vghrsday_m1i <- apply(ptsd_yes_ds2_vghrsday, 2, mean) #Calculate
```

```
m1i
ptsd_vghrsday_sd1i <- apply(ptsd_yes_ds2_vghrsday, 2, sd) #Calculate sd1i
ptsd_vghrsday_n1i <- colSums(!is.na(ptsd_yes_ds2_vghrsday)) #Calculate
n1i
ptsd_no_ds2_vghrsday <- subset(ptsd_no_ds2, select = c(vghrsday)) #Subset
2i data
ptsd_vghrsday_m2i <- apply(ptsd_no_ds2_vghrsday, 2, mean) #Calculate
m2i
ptsd_vghrsday_sd2i <- apply(ptsd_no_ds2_vghrsday, 2, sd) #Calculate sd2i
ptsd_vghrsday_n2i <- colSums(!is.na(ptsd_no_ds2_vghrsday)) #Calculate n2i
ds2ptsd_meta <- data.frame(m1i = ptsd_vghrsday_m1i, sd1i =
ptsd_vghrsday_sd1i, n1i = ptsd_vghrsday_n1i, m2i = ptsd_vghrsday_m2i,
sd2i = ptsd_vghrsday_sd2i, n2i = ptsd_vghrsday_n2i) #MA dataframe
ds2ptsd_meta <- escalc(measure="MD", m1i = m1i, sd1i = sd1i, n1i = n1i,
m2i = m2i, sd2i = sd2i, n2i = n2i, data=ds2ptsd_meta) #Calculate yi and vi

#FE meta-analysis
(ptsd_meta <- rbind(ds1ptsd_meta, ds2ptsd_meta))

##        m1i     sd1i n1i      m2i     sd2i n2i     yi     vi
## 1 2.565789 2.025242 152 2.116162 1.596837 198 0.4496 0.0399
## 2 4.057269 3.715702 227 3.211268 2.869652 355 0.8460 0.0840

(any_vghrsday_meta <- rma(yi=yi, vi=vi, measure="MD", method="FE",
data=ptsd_meta))

##
## Fixed-Effects Model (k = 2)
##
## Test for Heterogeneity:
## Q(df = 1) = 1.2683, p-val = 0.2601
##
## Model Results:
##
## estimate       se     zval     pval    ci.lb    ci.ub
##   0.5772   0.1644   3.5103   0.0004   0.2549   0.8994      ***
##
## ---
## Signif. codes:  0 '***' 0.001 '**' 0.01 '*' 0.05 '.' 0.1 ' ' 1
```

Hours per week

```
#Study 1
ptsd_yes_ds1_vghrsweek <- subset(ptsd_yes_ds1, select = c(vghrsweek))
#Subset 1i data
ptsd_vghrsweek_m1i <- apply(ptsd_yes_ds1_vghrsweek, 2, mean) #Calculate
m1i
ptsd_vghrsweek_sd1i <- apply(ptsd_yes_ds1_vghrsweek, 2, sd) #Calculate
sd1i
ptsd_vghrsweek_n1i <- colSums(!is.na(ptsd_yes_ds1_vghrsweek)) #Calculate
```

```
n1i
ptsd_no_ds1_vghrsweek <- subset(ptsd_no_ds1, select = c(vghrsweek))
#Subset 2i data
ptsd_vghrsweek_m2i <- apply(ptsd_no_ds1_vghrsweek, 2, mean) #Calculate
m2i
ptsd_vghrsweek_sd2i <- apply(ptsd_no_ds1_vghrsweek, 2, sd) #Calculate
sd2i
ptsd_vghrsweek_n2i <- colSums(!is.na(ptsd_no_ds1_vghrsweek)) #Calculate
n2i
ds1ptsd_meta <- data.frame(m1i = ptsd_vghrsweek_m1i, sd1i =
ptsd_vghrsweek_sd1i, n1i = ptsd_vghrsweek_n1i, m2i =
ptsd_vghrsweek_m2i, sd2i = ptsd_vghrsweek_sd2i, n2i =
ptsd_vghrsweek_n2i) #MA dataframe
ds1ptsd_meta <- escalc(measure="MD", m1i = m1i, sd1i = sd1i, n1i = n1i,
m2i = m2i, sd2i = sd2i, n2i = n2i, data=ds1ptsd_meta) #Calculate yi and vi

#Study 2
ptsd_yes_ds2_vghrsweek <- subset(ptsd_yes_ds2, select = c(vghrsweek))
#Subset 1i data
ptsd_vghrsweek_m1i <- apply(ptsd_yes_ds2_vghrsweek, 2, mean) #Calculate
m1i
ptsd_vghrsweek_sd1i <- apply(ptsd_yes_ds2_vghrsweek, 2, sd) #Calculate
sd1i
ptsd_vghrsweek_n1i <- colSums(!is.na(ptsd_yes_ds2_vghrsweek)) #Calculate
n1i
ptsd_no_ds2_vghrsweek <- subset(ptsd_no_ds2, select = c(vghrsweek))
#Subset 2i data
ptsd_vghrsweek_m2i <- apply(ptsd_no_ds2_vghrsweek, 2, mean) #Calculate
m2i
ptsd_vghrsweek_sd2i <- apply(ptsd_no_ds2_vghrsweek, 2, sd) #Calculate
sd2i
ptsd_vghrsweek_n2i <- colSums(!is.na(ptsd_no_ds2_vghrsweek)) #Calculate
n2i
ds2ptsd_meta <- data.frame(m1i = ptsd_vghrsweek_m1i, sd1i =
ptsd_vghrsweek_sd1i, n1i = ptsd_vghrsweek_n1i, m2i =
ptsd_vghrsweek_m2i, sd2i = ptsd_vghrsweek_sd2i, n2i =
ptsd_vghrsweek_n2i) #MA dataframe
ds2ptsd_meta <- escalc(measure="MD", m1i = m1i, sd1i = sd1i, n1i = n1i,
m2i = m2i, sd2i = sd2i, n2i = n2i, data=ds2ptsd_meta) #Calculate yi and vi

#FE meta-analysis
(ptsd_meta <- rbind(ds1ptsd_meta, ds2ptsd_meta))

##        m1i      sd1i n1i      m2i      sd2i n2i     yi     vi
## 1 14.67763 15.35393 152 11.37374 11.73488 198 3.3039 2.2464
## 2 22.59912 25.68276 227 15.65634 18.62703 355 6.9428 3.8831

(any_vghrsweek_meta <- rma(yi=yi, vi=vi, measure="MD", method="FE",
data=ptsd_meta))
```

```
##
## Fixed-Effects Model (k = 2)
##
## Test for Heterogeneity:
## Q(df = 1) = 2.1603, p-val = 0.1416
##
## Model Results:
##
## estimate      se     zval     pval    ci.lb    ci.ub
##   4.6375   1.1930   3.8874   0.0001   2.2994   6.9757      ***
##
## ---
## Signif. codes:  0 '***' 0.001 '**' 0.01 '*' 0.05 '.' 0.1 ' ' 1
```

Days per week

```
#Study 1
ptsd_yes_ds1_vgdaysweek <- subset(ptsd_yes_ds1, select = c(vgdaysweek))
#Subset 1i data
ptsd_vgdaysweek_m1i <- apply(ptsd_yes_ds1_vgdaysweek, 2, mean)
#Calculate m1i
ptsd_vgdaysweek_sd1i <- apply(ptsd_yes_ds1_vgdaysweek, 2, sd) #Calculate
sd1i
ptsd_vgdaysweek_n1i <- colSums(!is.na(ptsd_yes_ds1_vgdaysweek))
#Calculate n1i
ptsd_no_ds1_vgdaysweek <- subset(ptsd_no_ds1, select = c(vgdaysweek))
#Subset 2i data
ptsd_vgdaysweek_m2i <- apply(ptsd_no_ds1_vgdaysweek, 2, mean)
#Calculate m2i
ptsd_vgdaysweek_sd2i <- apply(ptsd_no_ds1_vgdaysweek, 2, sd) #Calculate
sd2i
ptsd_vgdaysweek_n2i <- colSums(!is.na(ptsd_no_ds1_vgdaysweek))
#Calculate n2i
ds1ptsd_meta <- data.frame(m1i = ptsd_vgdaysweek_m1i, sd1i =
ptsd_vgdaysweek_sd1i, n1i = ptsd_vgdaysweek_n1i, m2i =
ptsd_vgdaysweek_m2i, sd2i = ptsd_vgdaysweek_sd2i, n2i =
ptsd_vgdaysweek_n2i) #MA dataframe
ds1ptsd_meta <- escalc(measure="MD", m1i = m1i, sd1i = sd1i, n1i = n1i,
m2i = m2i, sd2i = sd2i, n2i = n2i, data=ds1ptsd_meta) #Calculate yi and vi

#Study 2
ptsd_yes_ds2_vgdaysweek <- subset(ptsd_yes_ds2, select = c(vgdaysweek))
#Subset 1i data
ptsd_vgdaysweek_m1i <- apply(ptsd_yes_ds2_vgdaysweek, 2, mean)
#Calculate m1i
ptsd_vgdaysweek_sd1i <- apply(ptsd_yes_ds2_vgdaysweek, 2, sd) #Calculate
sd1i
ptsd_vgdaysweek_n1i <- colSums(!is.na(ptsd_yes_ds2_vgdaysweek))
#Calculate n1i
```

```
ptsd_no_ds2_vgdaysweek <- subset(ptsd_no_ds2, select = c(vgdaysweek))
#Subset 2i data
ptsd_vgdaysweek_m2i <- apply(ptsd_no_ds2_vgdaysweek, 2, mean)
#Calculate m2i
ptsd_vgdaysweek_sd2i <- apply(ptsd_no_ds2_vgdaysweek, 2, sd) #Calculate
sd2i
ptsd_vgdaysweek_n2i <- colSums(!is.na(ptsd_no_ds2_vgdaysweek))
#Calculate n2i
ds2ptsd_meta <- data.frame(m1i = ptsd_vgdaysweek_m1i, sd1i =
ptsd_vgdaysweek_sd1i, n1i = ptsd_vgdaysweek_n1i, m2i =
ptsd_vgdaysweek_m2i, sd2i = ptsd_vgdaysweek_sd2i, n2i =
ptsd_vgdaysweek_n2i) #MA dataframe
ds2ptsd_meta <- escalc(measure="MD", m1i = m1i, sd1i = sd1i, n1i = n1i,
m2i = m2i, sd2i = sd2i, n2i = n2i, data=ds2ptsd_meta) #Calculate yi and vi

#FE meta-analysis
(ptsd_meta <- rbind(ds1ptsd_meta, ds2ptsd_meta))

##        m1i     sd1i n1i      m2i     sd2i n2i     yi     vi
## 1 5.157895 2.273094 152 4.929293 2.292130 198 0.2286 0.0605
## 2 5.022026 2.148152 227 4.532394 2.237569 355 0.4896 0.0344

(any_vgdaysweek_meta <- rma(yi=yi, vi=vi, measure="MD", method="FE",
data=ptsd_meta))

##
## Fixed-Effects Model (k = 2)
##
## Test for Heterogeneity:
## Q(df = 1) = 0.7175, p-val = 0.3970
##
## Model Results:
##
## estimate      se     zval     pval    ci.lb    ci.ub
##   0.3950   0.1481   2.6662   0.0077   0.1046   0.6853      **
##
## ---
## Signif. codes:  0 '***' 0.001 '**' 0.01 '*' 0.05 '.' 0.1 ' ' 1
```

Depression screen

Replicated Effect

Hours per day

Study 1

```
(vghrsday_depression_ds1 <- t.test(vghrsday ~ depression, data=ds1))
```

```
##
##  Welch Two Sample t-test
##
## data:  vghrsday by depression
## t = -2.9955, df = 324.01, p-value = 0.002951
## alternative hypothesis: true difference in means is not equal to 0
## 95 percent confidence interval:
##  -0.9548135 -0.1978231
## sample estimates:
## mean in group 0 mean in group 1
##        2.033149        2.609467
```

Study 2

```
(vghrsday_depression_ds2 <- t.test(vghrsday ~ depression, data=ds2))
```

```
##
##  Welch Two Sample t-test
##
## data:  vghrsday by depression
## t = -2.496, df = 529.18, p-value = 0.01286
## alternative hypothesis: true difference in means is not equal to 0
## 95 percent confidence interval:
##  -1.2133616 -0.1445957
## sample estimates:
## mean in group 0 mean in group 1
##        3.225080        3.904059
```

Hours per week

Study 1

```
(vghrsweek_depression_ds1 <- t.test(vghrsweek ~ depression, data=ds1))
```

```
##
##  Welch Two Sample t-test
##
## data:  vghrsweek by depression
## t = -3.1385, df = 320.04, p-value = 0.001856
## alternative hypothesis: true difference in means is not equal to 0
## 95 percent confidence interval:
##  -7.340716 -1.683639
## sample estimates:
## mean in group 0 mean in group 1
##        10.62983        15.14201
```

Study 2

```
(vghrsweek_depression_ds2 <- t.test(vghrsweek ~ depression, data=ds2))

##
##  Welch Two Sample t-test
##
## data:  vghrsweek by depression
## t = -2.8314, df = 512.1, p-value = 0.004818
## alternative hypothesis: true difference in means is not equal to 0
## 95 percent confidence interval:
##  -8.811186 -1.592392
## sample estimates:
## mean in group 0 mean in group 1
##        15.94212        21.14391
```

Days per week

Study 1

```
(vgdaysweek_depression_ds1 <- t.test(vgdaysweek ~ depression, data=ds1))

##
##  Welch Two Sample t-test
##
## data:  vgdaysweek by depression
## t = -2.2251, df = 347.68, p-value = 0.02672
## alternative hypothesis: true difference in means is not equal to 0
## 95 percent confidence interval:
##  -1.01682148 -0.06265154
## sample estimates:
## mean in group 0 mean in group 1
##        4.767956        5.307692
```

Study 2

```
(vgdaysweek_depression_ds2 <- t.test(vgdaysweek ~ depression, data=ds2))

##
##  Welch Two Sample t-test
##
## data:  vgdaysweek by depression
## t = -1.6935, df = 573.01, p-value = 0.0909
## alternative hypothesis: true difference in means is not equal to 0
## 95 percent confidence interval:
##  -0.67065343  0.04961192
## sample estimates:
## mean in group 0 mean in group 1
##        4.578778        4.889299
```

MD

Hours per day

```
#Study 1
depression_yes_ds1_vghrsday <- subset(depression_yes_ds1, select =
c(vghrsday)) #Subset 1i data
depression_vghrsday_m1i <- apply(depression_yes_ds1_vghrsday, 2, mean)
#Calculate m1i
depression_vghrsday_sd1i <- apply(depression_yes_ds1_vghrsday, 2, sd)
#Calculate sd1i
depression_vghrsday_n1i <- colSums(!is.na(depression_yes_ds1_vghrsday))
#Calculate n1i
depression_no_ds1_vghrsday <- subset(depression_no_ds1, select =
c(vghrsday)) #Subset 2i data
depression_vghrsday_m2i <- apply(depression_no_ds1_vghrsday, 2, mean)
#Calculate m2i
depression_vghrsday_sd2i <- apply(depression_no_ds1_vghrsday, 2, sd)
#Calculate sd2i
depression_vghrsday_n2i <- colSums(!is.na(depression_no_ds1_vghrsday))
#Calculate n2i
ds1depression_meta <- data.frame(m1i = depression_vghrsday_m1i, sd1i =
depression_vghrsday_sd1i, n1i = depression_vghrsday_n1i, m2i =
depression_vghrsday_m2i, sd2i = depression_vghrsday_sd2i, n2i =
depression_vghrsday_n2i) #MA dataframe
ds1depression_meta <- escalc(measure="MD", m1i = m1i, sd1i = sd1i, n1i =
n1i, m2i = m2i, sd2i = sd2i, n2i = n2i, data=ds1depression_meta)
#Calculate yi and vi

#Study 2
depression_yes_ds2_vghrsday <- subset(depression_yes_ds2, select =
c(vghrsday)) #Subset 1i data
depression_vghrsday_m1i <- apply(depression_yes_ds2_vghrsday, 2, mean)
#Calculate m1i
depression_vghrsday_sd1i <- apply(depression_yes_ds2_vghrsday, 2, sd)
#Calculate sd1i
depression_vghrsday_n1i <- colSums(!is.na(depression_yes_ds2_vghrsday))
#Calculate n1i
depression_no_ds2_vghrsday <- subset(depression_no_ds2, select =
c(vghrsday)) #Subset 2i data
depression_vghrsday_m2i <- apply(depression_no_ds2_vghrsday, 2, mean)
#Calculate m2i
depression_vghrsday_sd2i <- apply(depression_no_ds2_vghrsday, 2, sd)
#Calculate sd2i
depression_vghrsday_n2i <- colSums(!is.na(depression_no_ds2_vghrsday))
#Calculate n2i
ds2depression_meta <- data.frame(m1i = depression_vghrsday_m1i, sd1i =
depression_vghrsday_sd1i, n1i = depression_vghrsday_n1i, m2i =
depression_vghrsday_m2i, sd2i = depression_vghrsday_sd2i, n2i =
```

```
depression_vghrsday_n2i) #MA dataframe
ds2depression_meta <- escalc(measure="MD", m1i = m1i, sd1i = sd1i, n1i =
n1i, m2i = m2i, sd2i = sd2i, n2i = n2i, data=ds2depression_meta)
#Calculate yi and vi

#FE meta-analysis
(depression_meta <- rbind(ds1depression_meta, ds2depression_meta))
```

```
##        m1i     sd1i n1i      m2i     sd2i n2i     yi     vi
## 1 2.609467 1.967330 169 2.033149 1.598265 181 0.5763 0.0370
## 2 3.904059 3.526359 271 3.225080 2.956810 311 0.6790 0.0740
```

```
(any_vghrsday_meta <- rma(yi=yi, vi=vi, measure="MD", method="FE",
data=depression_meta))
```

```
##
## Fixed-Effects Model (k = 2)
##
## Test for Heterogeneity:
## Q(df = 1) = 0.0949, p-val = 0.7580
##
## Model Results:
##
## estimate      se     zval     pval    ci.lb    ci.ub
##   0.6105   0.1571   3.8870   0.0001   0.3027   0.9184      ***
##
## ---
## Signif. codes:  0 '***' 0.001 '**' 0.01 '*' 0.05 '.' 0.1 ' ' 1
```

Hours per week

```
#Study 1
depression_yes_ds1_vghrsweek <- subset(depression_yes_ds1, select =
c(vghrsweek)) #Subset 1i data
depression_vghrsweek_m1i <- apply(depression_yes_ds1_vghrsweek, 2,
mean) #Calculate m1i
depression_vghrsweek_sd1i <- apply(depression_yes_ds1_vghrsweek, 2, sd)
#Calculate sd1i
depression_vghrsweek_n1i <- colSums(!
is.na(depression_yes_ds1_vghrsweek)) #Calculate n1i
depression_no_ds1_vghrsweek <- subset(depression_no_ds1, select =
c(vghrsweek)) #Subset 2i data
depression_vghrsweek_m2i <- apply(depression_no_ds1_vghrsweek, 2,
mean) #Calculate m2i
depression_vghrsweek_sd2i <- apply(depression_no_ds1_vghrsweek, 2, sd)
#Calculate sd2i
depression_vghrsweek_n2i <- colSums(!is.na(depression_no_ds1_vghrsweek))
#Calculate n2i
ds1depression_meta <- data.frame(m1i = depression_vghrsweek_m1i, sd1i =
depression_vghrsweek_sd1i, n1i = depression_vghrsweek_n1i, m2i =
```

```
depression_vghrsweek_m2i, sd2i = depression_vghrsweek_sd2i, n2i =
depression_vghrsweek_n2i) #MA dataframe
ds1depression_meta <- escalc(measure="MD", m1i = m1i, sd1i = sd1i, n1i =
n1i, m2i = m2i, sd2i = sd2i, n2i = n2i, data=ds1depression_meta)
#Calculate yi and vi

#Study 2
depression_yes_ds2_vghrsweek <- subset(depression_yes_ds2, select =
c(vghrsweek)) #Subset 1i data
depression_vghrsweek_m1i <- apply(depression_yes_ds2_vghrsweek, 2,
mean) #Calculate m1i
depression_vghrsweek_sd1i <- apply(depression_yes_ds2_vghrsweek, 2, sd)
#Calculate sd1i
depression_vghrsweek_n1i <- colSums(!
is.na(depression_yes_ds2_vghrsweek)) #Calculate n1i
depression_no_ds2_vghrsweek <- subset(depression_no_ds2, select =
c(vghrsweek)) #Subset 2i data
depression_vghrsweek_m2i <- apply(depression_no_ds2_vghrsweek, 2,
mean) #Calculate m2i
depression_vghrsweek_sd2i <- apply(depression_no_ds2_vghrsweek, 2, sd)
#Calculate sd2i
depression_vghrsweek_n2i <- colSums(!is.na(depression_no_ds2_vghrsweek))
#Calculate n2i
ds2depression_meta <- data.frame(m1i = depression_vghrsweek_m1i, sd1i =
depression_vghrsweek_sd1i, n1i = depression_vghrsweek_n1i, m2i =
depression_vghrsweek_m2i, sd2i = depression_vghrsweek_sd2i, n2i =
depression_vghrsweek_n2i) #MA dataframe
ds2depression_meta <- escalc(measure="MD", m1i = m1i, sd1i = sd1i, n1i =
n1i, m2i = m2i, sd2i = sd2i, n2i = n2i, data=ds2depression_meta)
#Calculate yi and vi

#FE meta-analysis
(depression_meta <- rbind(ds1depression_meta, ds2depression_meta))

##        m1i     sd1i n1i      m2i     sd2i n2i     yi     vi
## 1 15.14201 14.84014 169 10.62983 11.75826 181 4.5122 2.0670
## 2 21.14391 24.33024 271 15.94212 19.24569 311 5.2018 3.3753

(any_vghrsweek_meta <- rma(yi=yi, vi=vi, measure="MD", method="FE",
data=depression_meta))

##
## Fixed-Effects Model (k = 2)
##
## Test for Heterogeneity:
## Q(df = 1) = 0.0874, p-val = 0.7675
##
## Model Results:
##
## estimate      se     zval     pval    ci.lb    ci.ub
##   4.7741   1.1322   4.2165   <.0001   2.5550   6.9932      ***
```

```
##
## ---
## Signif. codes:  0 '***' 0.001 '**' 0.01 '*' 0.05 '.' 0.1 ' ' 1
```

Days per week

```
#Study 1
depression_yes_ds1_vgdaysweek <- subset(depression_yes_ds1, select =
c(vgdaysweek)) #Subset 1i data
depression_vgdaysweek_m1i <- apply(depression_yes_ds1_vgdaysweek, 2,
mean) #Calculate m1i
depression_vgdaysweek_sd1i <- apply(depression_yes_ds1_vgdaysweek, 2,
sd) #Calculate sd1i
depression_vgdaysweek_n1i <- colSums(!
is.na(depression_yes_ds1_vgdaysweek)) #Calculate n1i
depression_no_ds1_vgdaysweek <- subset(depression_no_ds1, select =
c(vgdaysweek)) #Subset 2i data
depression_vgdaysweek_m2i <- apply(depression_no_ds1_vgdaysweek, 2,
mean) #Calculate m2i
depression_vgdaysweek_sd2i <- apply(depression_no_ds1_vgdaysweek, 2,
sd) #Calculate sd2i
depression_vgdaysweek_n2i <- colSums(!
is.na(depression_no_ds1_vgdaysweek)) #Calculate n2i
ds1depression_meta <- data.frame(m1i = depression_vgdaysweek_m1i, sd1i
= depression_vgdaysweek_sd1i, n1i = depression_vgdaysweek_n1i, m2i =
depression_vgdaysweek_m2i, sd2i = depression_vgdaysweek_sd2i, n2i =
depression_vgdaysweek_n2i) #MA dataframe
ds1depression_meta <- escalc(measure="MD", m1i = m1i, sd1i = sd1i, n1i =
n1i, m2i = m2i, sd2i = sd2i, n2i = n2i, data=ds1depression_meta)
#Calculate yi and vi

#Study 2
depression_yes_ds2_vgdaysweek <- subset(depression_yes_ds2, select =
c(vgdaysweek)) #Subset 1i data
depression_vgdaysweek_m1i <- apply(depression_yes_ds2_vgdaysweek, 2,
mean) #Calculate m1i
depression_vgdaysweek_sd1i <- apply(depression_yes_ds2_vgdaysweek, 2,
sd) #Calculate sd1i
depression_vgdaysweek_n1i <- colSums(!
is.na(depression_yes_ds2_vgdaysweek)) #Calculate n1i
depression_no_ds2_vgdaysweek <- subset(depression_no_ds2, select =
c(vgdaysweek)) #Subset 2i data
depression_vgdaysweek_m2i <- apply(depression_no_ds2_vgdaysweek, 2,
mean) #Calculate m2i
depression_vgdaysweek_sd2i <- apply(depression_no_ds2_vgdaysweek, 2,
sd) #Calculate sd2i
depression_vgdaysweek_n2i <- colSums(!
is.na(depression_no_ds2_vgdaysweek)) #Calculate n2i
ds2depression_meta <- data.frame(m1i = depression_vgdaysweek_m1i, sd1i
```

```
= depression_vgdaysweek_sd1i, n1i = depression_vgdaysweek_n1i, m2i =
depression_vgdaysweek_m2i, sd2i = depression_vgdaysweek_sd2i, n2i =
depression_vgdaysweek_n2i) #MA dataframe
ds2depression_meta <- escalc(measure="MD", m1i = m1i, sd1i = sd1i, n1i =
n1i, m2i = m2i, sd2i = sd2i, n2i = n2i, data=ds2depression_meta)
#Calculate yi and vi

#FE meta-analysis
(depression_meta <- rbind(ds1depression_meta, ds2depression_meta))

##        m1i     sd1i n1i      m2i     sd2i n2i     yi     vi
## 1 5.307692 2.225395 169 4.767956 2.312111 181 0.5397 0.0588
## 2 4.889299 2.177902 271 4.578778 2.238822 311 0.3105 0.0336

(any_vgdaysweek_meta <- rma(yi=yi, vi=vi, measure="MD", method="FE",
data=depression_meta))

##
## Fixed-Effects Model (k = 2)
##
## Test for Heterogeneity:
## Q(df = 1) = 0.5683, p-val = 0.4510
##
## Model Results:
##
## estimate      se    zval    pval   ci.lb   ci.ub
##   0.3939  0.1463  2.6927  0.0071  0.1072  0.6806      **
##
## ---
## Signif. codes:  0 '***' 0.001 '**' 0.01 '*' 0.05 '.' 0.1 ' ' 1
```

Any SUD screen

Replicated Effect

Hours per day

Study 1

```
(vghrsday_anysudscreen_ds1 <- t.test(vghrsday ~ anysudscreen, data=ds1))

##
##  Welch Two Sample t-test
##
## data:  vghrsday by anysudscreen
## t = 1.4664, df = 288.13, p-value = 0.1436
## alternative hypothesis: true difference in means is not equal to 0
## 95 percent confidence interval:
```

```
## -0.09918005  0.67875421
## sample estimates:
## mean in group 0 mean in group 1
##        2.466258        2.176471
```

Study 2

```
(vghrsday_anysudscreen_ds2 <- t.test(vghrsday ~ anysudscreen, data=ds2))

##
##  Welch Two Sample t-test
##
## data:  vghrsday by anysudscreen
## t = -0.39796, df = 522.95, p-value = 0.6908
## alternative hypothesis: true difference in means is not equal to 0
## 95 percent confidence interval:
##  -0.6467302  0.4288460
## sample estimates:
## mean in group 0 mean in group 1
##        3.490323        3.599265
```

Hours per week

Study 1

```
(vghrsweek_anysudscreen_ds1 <- t.test(vghrsweek ~ anysudscreen,
data=ds1))

##
##  Welch Two Sample t-test
##
## data:  vghrsweek by anysudscreen
## t = 1.1582, df = 288.12, p-value = 0.2477
## alternative hypothesis: true difference in means is not equal to 0
## 95 percent confidence interval:
##  -1.198254  4.624717
## sample estimates:
## mean in group 0 mean in group 1
##        13.72393        12.01070
```

Study 2

```
(vghrsweek_anysudscreen_ds2 <- t.test(vghrsweek ~ anysudscreen,
data=ds2))

##
##  Welch Two Sample t-test
##
```

```
## data:  vghrsweek by anysudscreen
## t = -0.23956, df = 530.76, p-value = 0.8108
## alternative hypothesis: true difference in means is not equal to 0
## 95 percent confidence interval:
##  -4.059154  3.176754
## sample estimates:
## mean in group 0 mean in group 1
##        18.15806        18.59926
```

Days per week

Study 1

```
(vgdaysweek_anysudscreen_ds1 <- t.test(vgdaysweek ~ anysudscreen,
data=ds1))

##
##  Welch Two Sample t-test
##
## data:  vgdaysweek by anysudscreen
## t = -0.73081, df = 333.82, p-value = 0.4654
## alternative hypothesis: true difference in means is not equal to 0
## 95 percent confidence interval:
##  -0.6637049  0.3041366
## sample estimates:
## mean in group 0 mean in group 1
##        4.932515        5.112299
```

Study 2

```
(vgdaysweek_anysudscreen_ds2 <- t.test(vgdaysweek ~ anysudscreen,
data=ds2))

##
##  Welch Two Sample t-test
##
## data:  vgdaysweek by anysudscreen
## t = -0.0091488, df = 570.67, p-value = 0.9927
## alternative hypothesis: true difference in means is not equal to 0
## 95 percent confidence interval:
##  -0.3632316  0.3598635
## sample estimates:
## mean in group 0 mean in group 1
##        4.722581        4.724265
```

MD

Hours per day

```
#Study 1
anysudscreen_yes_ds1_vghrsday <- subset(anysudscreen_yes_ds1, select =
c(vghrsday)) #Subset 1i data
anysudscreen_vghrsday_m1i <- apply(anysudscreen_yes_ds1_vghrsday, 2,
mean) #Calculate m1i
anysudscreen_vghrsday_sd1i <- apply(anysudscreen_yes_ds1_vghrsday, 2,
sd) #Calculate sd1i
anysudscreen_vghrsday_n1i <- colSums(!
is.na(anysudscreen_yes_ds1_vghrsday)) #Calculate n1i
anysudscreen_no_ds1_vghrsday <- subset(anysudscreen_no_ds1, select =
c(vghrsday)) #Subset 2i data
anysudscreen_vghrsday_m2i <- apply(anysudscreen_no_ds1_vghrsday, 2,
mean) #Calculate m2i
anysudscreen_vghrsday_sd2i <- apply(anysudscreen_no_ds1_vghrsday, 2, sd)
#Calculate sd2i
anysudscreen_vghrsday_n2i <- colSums(!
is.na(anysudscreen_no_ds1_vghrsday)) #Calculate n2i
ds1anysudscreen_meta <- data.frame(m1i = anysudscreen_vghrsday_m1i,
sd1i = anysudscreen_vghrsday_sd1i, n1i = anysudscreen_vghrsday_n1i, m2i
= anysudscreen_vghrsday_m2i, sd2i = anysudscreen_vghrsday_sd2i, n2i =
anysudscreen_vghrsday_n2i) #MA dataframe
ds1anysudscreen_meta <- escalc(measure="MD", m1i = m1i, sd1i = sd1i,
n1i = n1i, m2i = m2i, sd2i = sd2i, n2i = n2i, data=ds1anysudscreen_meta)
#Calculate yi and vi

#Study 2
anysudscreen_yes_ds2_vghrsday <- subset(anysudscreen_yes_ds2, select =
c(vghrsday)) #Subset 1i data
anysudscreen_vghrsday_m1i <- apply(anysudscreen_yes_ds2_vghrsday, 2,
mean) #Calculate m1i
anysudscreen_vghrsday_sd1i <- apply(anysudscreen_yes_ds2_vghrsday, 2,
sd) #Calculate sd1i
anysudscreen_vghrsday_n1i <- colSums(!
is.na(anysudscreen_yes_ds2_vghrsday)) #Calculate n1i
anysudscreen_no_ds2_vghrsday <- subset(anysudscreen_no_ds2, select =
c(vghrsday)) #Subset 2i data
anysudscreen_vghrsday_m2i <- apply(anysudscreen_no_ds2_vghrsday, 2,
mean) #Calculate m2i
anysudscreen_vghrsday_sd2i <- apply(anysudscreen_no_ds2_vghrsday, 2, sd)
#Calculate sd2i
anysudscreen_vghrsday_n2i <- colSums(!
is.na(anysudscreen_no_ds2_vghrsday)) #Calculate n2i
ds2anysudscreen_meta <- data.frame(m1i = anysudscreen_vghrsday_m1i,
sd1i = anysudscreen_vghrsday_sd1i, n1i = anysudscreen_vghrsday_n1i, m2i
= anysudscreen_vghrsday_m2i, sd2i = anysudscreen_vghrsday_sd2i, n2i =
anysudscreen_vghrsday_n2i) #MA dataframe
```

```
ds2anysudscreen_meta <- escalc(measure="MD", m1i = m1i, sd1i = sd1i,
n1i = n1i, m2i = m2i, sd2i = sd2i, n2i = n2i, data=ds2anysudscreen_meta)
#Calculate yi and vi

#FE meta-analysis
(anysudscreen_meta <- rbind(ds1anysudscreen_meta,
ds2anysudscreen_meta))
```

```
##        m1i     sd1i n1i      m2i     sd2i n2i      yi     vi
## 1 2.176471 1.497626 187 2.466258 2.100212 163 -0.2898 0.0391
## 2 3.599265 3.589303 272 3.490323 2.923773 310  0.1089 0.0749
```

```
(any_vghrsday_meta <- rma(yi=yi, vi=vi, measure="MD", method="FE",
data=anysudscreen_meta))
```

```
##
## Fixed-Effects Model (k = 2)
##
## Test for Heterogeneity:
## Q(df = 1) = 1.3947, p-val = 0.2376
##
## Model Results:
##
## estimate       se     zval     pval    ci.lb    ci.ub
##  -0.1532   0.1602  -0.9560   0.3391  -0.4672   0.1609
##
## ---
## Signif. codes:  0 '***' 0.001 '**' 0.01 '*' 0.05 '.' 0.1 ' ' 1
```

Hours per week

```
#Study 1
anysudscreen_yes_ds1_vghrsweek <- subset(anysudscreen_yes_ds1, select =
c(vghrsweek)) #Subset 1i data
anysudscreen_vghrsweek_m1i <- apply(anysudscreen_yes_ds1_vghrsweek, 2,
mean) #Calculate m1i
anysudscreen_vghrsweek_sd1i <- apply(anysudscreen_yes_ds1_vghrsweek,
2, sd) #Calculate sd1i
anysudscreen_vghrsweek_n1i <- colSums(!
is.na(anysudscreen_yes_ds1_vghrsweek)) #Calculate n1i
anysudscreen_no_ds1_vghrsweek <- subset(anysudscreen_no_ds1, select =
c(vghrsweek)) #Subset 2i data
anysudscreen_vghrsweek_m2i <- apply(anysudscreen_no_ds1_vghrsweek, 2,
mean) #Calculate m2i
anysudscreen_vghrsweek_sd2i <- apply(anysudscreen_no_ds1_vghrsweek, 2,
sd) #Calculate sd2i
anysudscreen_vghrsweek_n2i <- colSums(!
is.na(anysudscreen_no_ds1_vghrsweek)) #Calculate n2i
ds1anysudscreen_meta <- data.frame(m1i = anysudscreen_vghrsweek_m1i,
sd1i = anysudscreen_vghrsweek_sd1i, n1i = anysudscreen_vghrsweek_n1i,
```

```
m2i = anysudscreen_vghrsweek_m2i, sd2i = anysudscreen_vghrsweek_sd2i,
n2i = anysudscreen_vghrsweek_n2i) #MA dataframe
ds1anysudscreen_meta <- escalc(measure="MD", m1i = m1i, sd1i = sd1i,
n1i = n1i, m2i = m2i, sd2i = sd2i, n2i = n2i, data=ds1anysudscreen_meta)
#Calculate yi and vi

#Study 2
anysudscreen_yes_ds2_vghrsweek <- subset(anysudscreen_yes_ds2, select =
c(vghrsweek)) #Subset 1i data
anysudscreen_vghrsweek_m1i <- apply(anysudscreen_yes_ds2_vghrsweek, 2,
mean) #Calculate m1i
anysudscreen_vghrsweek_sd1i <- apply(anysudscreen_yes_ds2_vghrsweek,
2, sd) #Calculate sd1i
anysudscreen_vghrsweek_n1i <- colSums(!
is.na(anysudscreen_yes_ds2_vghrsweek)) #Calculate n1i
anysudscreen_no_ds2_vghrsweek <- subset(anysudscreen_no_ds2, select =
c(vghrsweek)) #Subset 2i data
anysudscreen_vghrsweek_m2i <- apply(anysudscreen_no_ds2_vghrsweek, 2,
mean) #Calculate m2i
anysudscreen_vghrsweek_sd2i <- apply(anysudscreen_no_ds2_vghrsweek, 2,
sd) #Calculate sd2i
anysudscreen_vghrsweek_n2i <- colSums(!
is.na(anysudscreen_no_ds2_vghrsweek)) #Calculate n2i
ds2anysudscreen_meta <- data.frame(m1i = anysudscreen_vghrsweek_m1i,
sd1i = anysudscreen_vghrsweek_sd1i, n1i = anysudscreen_vghrsweek_n1i,
m2i = anysudscreen_vghrsweek_m2i, sd2i = anysudscreen_vghrsweek_sd2i,
n2i = anysudscreen_vghrsweek_n2i) #MA dataframe
ds2anysudscreen_meta <- escalc(measure="MD", m1i = m1i, sd1i = sd1i,
n1i = n1i, m2i = m2i, sd2i = sd2i, n2i = n2i, data=ds2anysudscreen_meta)
#Calculate yi and vi

#FE meta-analysis
(anysudscreen_meta <- rbind(ds1anysudscreen_meta,
ds2anysudscreen_meta))

##        m1i     sd1i n1i      m2i     sd2i n2i      yi     vi
## 1 12.01070 11.20963 187 13.72393 15.72067 163 -1.7132 2.1881
## 2 18.59926 23.90150 272 18.15806 20.01013 310  0.4412 3.3919

(any_vghrsweek_meta <- rma(yi=yi, vi=vi, measure="MD", method="FE",
data=anysudscreen_meta))

##
## Fixed-Effects Model (k = 2)
##
## Test for Heterogeneity:
## Q(df = 1) = 0.8318, p-val = 0.3617
##
## Model Results:
##
## estimate      se     zval     pval    ci.lb    ci.ub
```

```
##  -0.8684   1.1533  -0.7530   0.4515  -3.1288   1.3920
##
## ---
## Signif. codes:  0 '***' 0.001 '**' 0.01 '*' 0.05 '.' 0.1 ' ' 1
```

Days per week

```
#Study 1
anysudscreen_yes_ds1_vgdaysweek <- subset(anysudscreen_yes_ds1, select
= c(vgdaysweek)) #Subset 1i data
anysudscreen_vgdaysweek_m1i <-
apply(anysudscreen_yes_ds1_vgdaysweek, 2, mean) #Calculate m1i
anysudscreen_vgdaysweek_sd1i <-
apply(anysudscreen_yes_ds1_vgdaysweek, 2, sd) #Calculate sd1i
anysudscreen_vgdaysweek_n1i <- colSums(!
is.na(anysudscreen_yes_ds1_vgdaysweek)) #Calculate n1i
anysudscreen_no_ds1_vgdaysweek <- subset(anysudscreen_no_ds1, select =
c(vgdaysweek)) #Subset 2i data
anysudscreen_vgdaysweek_m2i <- apply(anysudscreen_no_ds1_vgdaysweek,
2, mean) #Calculate m2i
anysudscreen_vgdaysweek_sd2i <- apply(anysudscreen_no_ds1_vgdaysweek,
2, sd) #Calculate sd2i
anysudscreen_vgdaysweek_n2i <- colSums(!
is.na(anysudscreen_no_ds1_vgdaysweek)) #Calculate n2i
ds1anysudscreen_meta <- data.frame(m1i =
anysudscreen_vgdaysweek_m1i, sd1i = anysudscreen_vgdaysweek_sd1i, n1i
= anysudscreen_vgdaysweek_n1i, m2i = anysudscreen_vgdaysweek_m2i,
sd2i = anysudscreen_vgdaysweek_sd2i, n2i =
anysudscreen_vgdaysweek_n2i) #MA dataframe
ds1anysudscreen_meta <- escalc(measure="MD", m1i = m1i, sd1i = sd1i,
n1i = n1i, m2i = m2i, sd2i = sd2i, n2i = n2i, data=ds1anysudscreen_meta)
#Calculate yi and vi

#Study 2
anysudscreen_yes_ds2_vgdaysweek <- subset(anysudscreen_yes_ds2, select
= c(vgdaysweek)) #Subset 1i data
anysudscreen_vgdaysweek_m1i <-
apply(anysudscreen_yes_ds2_vgdaysweek, 2, mean) #Calculate m1i
anysudscreen_vgdaysweek_sd1i <-
apply(anysudscreen_yes_ds2_vgdaysweek, 2, sd) #Calculate sd1i
anysudscreen_vgdaysweek_n1i <- colSums(!
is.na(anysudscreen_yes_ds2_vgdaysweek)) #Calculate n1i
anysudscreen_no_ds2_vgdaysweek <- subset(anysudscreen_no_ds2, select =
c(vgdaysweek)) #Subset 2i data
anysudscreen_vgdaysweek_m2i <- apply(anysudscreen_no_ds2_vgdaysweek,
2, mean) #Calculate m2i
anysudscreen_vgdaysweek_sd2i <- apply(anysudscreen_no_ds2_vgdaysweek,
2, sd) #Calculate sd2i
anysudscreen_vgdaysweek_n2i <- colSums(!
```

```
is.na(anysudscreen_no_ds2_vgdaysweek)) #Calculate n2i
ds2anysudscreen_meta <- data.frame(m1i =
anysudscreen_vgdaysweek_m1i, sd1i = anysudscreen_vgdaysweek_sd1i, n1i
= anysudscreen_vgdaysweek_n1i, m2i = anysudscreen_vgdaysweek_m2i,
sd2i = anysudscreen_vgdaysweek_sd2i, n2i =
anysudscreen_vgdaysweek_n2i) #MA dataframe
ds2anysudscreen_meta <- escalc(measure="MD", m1i = m1i, sd1i = sd1i,
n1i = n1i, m2i = m2i, sd2i = sd2i, n2i = n2i, data=ds2anysudscreen_meta)
#Calculate yi and vi

#FE meta-analysis
(anysudscreen_meta <- rbind(ds1anysudscreen_meta,
ds2anysudscreen_meta))

##        m1i     sd1i n1i      m2i     sd2i n2i     yi     vi
## 1 5.112299 2.210242 187 4.932515 2.367811 163 0.1798 0.0605
## 2 4.724265 2.212277 272 4.722581 2.219465 310 0.0017 0.0339

(any_vgdaysweek_meta <- rma(yi=yi, vi=vi, measure="MD", method="FE",
data=anysudscreen_meta))

##
## Fixed-Effects Model (k = 2)
##
## Test for Heterogeneity:
## Q(df = 1) = 0.3360, p-val = 0.5621
##
## Model Results:
##
## estimate      se    zval    pval    ci.lb   ci.ub
##   0.0656  0.1474  0.4452  0.6562  -0.2233  0.3545
##
## ---
## Signif. codes:  0 '***' 0.001 '**' 0.01 '*' 0.05 '.' 0.1 ' ' 1
```

AUD screen

Replicated Effect

Hours per day

Study 1

```
(vghrsday_audit_ds1 <- t.test(vghrsday ~ audit, data=ds1))

##
##  Welch Two Sample t-test
##
```

```
## data:  vghrsday by audit
## t = 0.43577, df = 347.95, p-value = 0.6633
## alternative hypothesis: true difference in means is not equal to 0
## 95 percent confidence interval:
##  -0.2875118  0.4511779
## sample estimates:
## mean in group 0 mean in group 1
##        2.346734        2.264901
```

Study 2

```
(vghrsday_audit_ds2 <- t.test(vghrsday ~ audit, data=ds2))
```

```
##
##  Welch Two Sample t-test
##
## data:  vghrsday by audit
## t = 0.61131, df = 319.39, p-value = 0.5414
## alternative hypothesis: true difference in means is not equal to 0
## 95 percent confidence interval:
##  -0.4032843  0.7668746
## sample estimates:
## mean in group 0 mean in group 1
##        3.595588        3.413793
```

Hours per week

Study 1

```
(vghrsweek_audit_ds1 <- t.test(vghrsweek ~ audit, data=ds1))
```

```
##
##  Welch Two Sample t-test
##
## data:  vghrsweek by audit
## t = 0.03389, df = 348, p-value = 0.973
## alternative hypothesis: true difference in means is not equal to 0
## 95 percent confidence interval:
##  -2.719887  2.815264
## sample estimates:
## mean in group 0 mean in group 1
##        12.82915        12.78146
```

Study 2

```
(vghrsweek_audit_ds2 <- t.test(vghrsweek ~ audit, data=ds2))
```

```
##
##  Welch Two Sample t-test
##
## data:  vghrsweek by audit
## t = 0.51384, df = 320.73, p-value = 0.6077
## alternative hypothesis: true difference in means is not equal to 0
## 95 percent confidence interval:
##  -2.907697  4.963478
## sample estimates:
## mean in group 0 mean in group 1
##        18.67157        17.64368
```

Days per week

Study 1

```
(vgdaysweek_audit_ds1 <- t.test(vgdaysweek ~ audit, data=ds1))

##
##  Welch Two Sample t-test
##
## data:  vgdaysweek by audit
## t = -1.3703, df = 333.43, p-value = 0.1715
## alternative hypothesis: true difference in means is not equal to 0
## 95 percent confidence interval:
##  -0.8137514  0.1455096
## sample estimates:
## mean in group 0 mean in group 1
##        4.884422        5.218543
```

Study 2

```
(vgdaysweek_audit_ds2 <- t.test(vgdaysweek ~ audit, data=ds2))

##
##  Welch Two Sample t-test
##
## data:  vgdaysweek by audit
## t = -0.58516, df = 336.54, p-value = 0.5588
## alternative hypothesis: true difference in means is not equal to 0
## 95 percent confidence interval:
##  -0.5053819  0.2736375
## sample estimates:
## mean in group 0 mean in group 1
##        4.688725        4.804598
```

MD

Hours per day

```
#Study 1
audit_yes_ds1_vghrsday <- subset(audit_yes_ds1, select = c(vghrsday)) #Subset 1i data
audit_vghrsday_m1i <- apply(audit_yes_ds1_vghrsday, 2, mean) #Calculate m1i
audit_vghrsday_sd1i <- apply(audit_yes_ds1_vghrsday, 2, sd) #Calculate sd1i
audit_vghrsday_n1i <- colSums(!is.na(audit_yes_ds1_vghrsday)) #Calculate n1i
audit_no_ds1_vghrsday <- subset(audit_no_ds1, select = c(vghrsday)) #Subset 2i data
audit_vghrsday_m2i <- apply(audit_no_ds1_vghrsday, 2, mean) #Calculate m2i
audit_vghrsday_sd2i <- apply(audit_no_ds1_vghrsday, 2, sd) #Calculate sd2i
audit_vghrsday_n2i <- colSums(!is.na(audit_no_ds1_vghrsday)) #Calculate n2i
ds1audit_meta <- data.frame(m1i = audit_vghrsday_m1i, sd1i = audit_vghrsday_sd1i, n1i = audit_vghrsday_n1i, m2i = audit_vghrsday_m2i, sd2i = audit_vghrsday_sd2i, n2i = audit_vghrsday_n2i) #MA dataframe
ds1audit_meta <- escalc(measure="MD", m1i = m1i, sd1i = sd1i, n1i = n1i, m2i = m2i, sd2i = sd2i, n2i = n2i, data=ds1audit_meta) #Calculate yi and vi

#Study 2
audit_yes_ds2_vghrsday <- subset(audit_yes_ds2, select = c(vghrsday)) #Subset 1i data
audit_vghrsday_m1i <- apply(audit_yes_ds2_vghrsday, 2, mean) #Calculate m1i
audit_vghrsday_sd1i <- apply(audit_yes_ds2_vghrsday, 2, sd) #Calculate sd1i
audit_vghrsday_n1i <- colSums(!is.na(audit_yes_ds2_vghrsday)) #Calculate n1i
audit_no_ds2_vghrsday <- subset(audit_no_ds2, select = c(vghrsday)) #Subset 2i data
audit_vghrsday_m2i <- apply(audit_no_ds2_vghrsday, 2, mean) #Calculate m2i
audit_vghrsday_sd2i <- apply(audit_no_ds2_vghrsday, 2, sd) #Calculate sd2i
audit_vghrsday_n2i <- colSums(!is.na(audit_no_ds2_vghrsday)) #Calculate n2i
ds2audit_meta <- data.frame(m1i = audit_vghrsday_m1i, sd1i = audit_vghrsday_sd1i, n1i = audit_vghrsday_n1i, m2i = audit_vghrsday_m2i, sd2i = audit_vghrsday_sd2i, n2i = audit_vghrsday_n2i) #MA dataframe
ds2audit_meta <- escalc(measure="MD", m1i = m1i, sd1i = sd1i, n1i = n1i, m2i = m2i, sd2i = sd2i, n2i = n2i, data=ds2audit_meta) #Calculate yi and vi

#FE meta-analysis
(audit_meta <- rbind(ds1audit_meta, ds2audit_meta))
```

```
##        m1i     sd1i n1i      m2i     sd2i n2i      yi     vi
## 1 2.264901 1.504225 151 2.346734 2.008914 199 -0.0818 0.0353
## 2 3.413793 3.308953 174 3.595588 3.226225 408 -0.1818 0.0884
```

```
(any_vghrsday_meta <- rma(yi=yi, vi=vi, measure="MD", method="FE",
data=audit_meta))
```

```
##
## Fixed-Effects Model (k = 2)
##
## Test for Heterogeneity:
## Q(df = 1) = 0.0808, p-val = 0.7762
##
## Model Results:
##
## estimate       se     zval     pval    ci.lb    ci.ub
##  -0.1103   0.1588  -0.6949   0.4871  -0.4215   0.2009
##
## ---
## Signif. codes:  0 '***' 0.001 '**' 0.01 '*' 0.05 '.' 0.1 ' ' 1
```

Hours per week

```
#Study 1
audit_yes_ds1_vghrsweek <- subset(audit_yes_ds1, select = c(vghrsweek))
#Subset 1i data
audit_vghrsweek_m1i <- apply(audit_yes_ds1_vghrsweek, 2, mean)
#Calculate m1i
audit_vghrsweek_sd1i <- apply(audit_yes_ds1_vghrsweek, 2, sd) #Calculate
sd1i
audit_vghrsweek_n1i <- colSums(!is.na(audit_yes_ds1_vghrsweek))
#Calculate n1i
audit_no_ds1_vghrsweek <- subset(audit_no_ds1, select = c(vghrsweek))
#Subset 2i data
audit_vghrsweek_m2i <- apply(audit_no_ds1_vghrsweek, 2, mean)
#Calculate m2i
audit_vghrsweek_sd2i <- apply(audit_no_ds1_vghrsweek, 2, sd) #Calculate
sd2i
audit_vghrsweek_n2i <- colSums(!is.na(audit_no_ds1_vghrsweek))
#Calculate n2i
ds1audit_meta <- data.frame(m1i = audit_vghrsweek_m1i, sd1i =
audit_vghrsweek_sd1i, n1i = audit_vghrsweek_n1i, m2i =
audit_vghrsweek_m2i, sd2i = audit_vghrsweek_sd2i, n2i =
audit_vghrsweek_n2i) #MA dataframe
ds1audit_meta <- escalc(measure="MD", m1i = m1i, sd1i = sd1i, n1i = n1i,
m2i = m2i, sd2i = sd2i, n2i = n2i, data=ds1audit_meta) #Calculate yi and vi

#Study 2
audit_yes_ds2_vghrsweek <- subset(audit_yes_ds2, select = c(vghrsweek))
```

```
#Subset 1i data
audit_vghrsweek_m1i <- apply(audit_yes_ds2_vghrsweek, 2, mean)
#Calculate m1i
audit_vghrsweek_sd1i <- apply(audit_yes_ds2_vghrsweek, 2, sd) #Calculate
sd1i
audit_vghrsweek_n1i <- colSums(!is.na(audit_yes_ds2_vghrsweek))
#Calculate n1i
audit_no_ds2_vghrsweek <- subset(audit_no_ds2, select = c(vghrsweek))
#Subset 2i data
audit_vghrsweek_m2i <- apply(audit_no_ds2_vghrsweek, 2, mean)
#Calculate m2i
audit_vghrsweek_sd2i <- apply(audit_no_ds2_vghrsweek, 2, sd) #Calculate
sd2i
audit_vghrsweek_n2i <- colSums(!is.na(audit_no_ds2_vghrsweek))
#Calculate n2i
ds2audit_meta <- data.frame(m1i = audit_vghrsweek_m1i, sd1i =
audit_vghrsweek_sd1i, n1i = audit_vghrsweek_n1i, m2i =
audit_vghrsweek_m2i, sd2i = audit_vghrsweek_sd2i, n2i =
audit_vghrsweek_n2i) #MA dataframe
ds2audit_meta <- escalc(measure="MD", m1i = m1i, sd1i = sd1i, n1i = n1i,
m2i = m2i, sd2i = sd2i, n2i = n2i, data=ds2audit_meta) #Calculate yi and vi

#FE meta-analysis
(audit_meta <- rbind(ds1audit_meta, ds2audit_meta))

##        m1i     sd1i n1i      m2i     sd2i n2i      yi     vi
## 1 12.78146 11.37476 151 12.82915 14.95046 199 -0.0477 1.9801
## 2 17.64368 22.22818 174 18.67157 21.77414 408 -1.0279 4.0017

(any_vghrsweek_meta <- rma(yi=yi, vi=vi, measure="MD", method="FE",
data=audit_meta))

##
## Fixed-Effects Model (k = 2)
##
## Test for Heterogeneity:
## Q(df = 1) = 0.1606, p-val = 0.6886
##
## Model Results:
##
## estimate       se     zval     pval    ci.lb    ci.ub
##  -0.3722   1.1509  -0.3234   0.7464  -2.6279   1.8836
##
## ---
## Signif. codes:  0 '***' 0.001 '**' 0.01 '*' 0.05 '.' 0.1 ' ' 1
```

Days per week

```
#Study 1
audit_yes_ds1_vgdaysweek <- subset(audit_yes_ds1, select =
```

```
c(vgdaysweek)) #Subset 1i data
audit_vgdaysweek_m1i <- apply(audit_yes_ds1_vgdaysweek, 2, mean)
#Calculate m1i
audit_vgdaysweek_sd1i <- apply(audit_yes_ds1_vgdaysweek, 2, sd)
#Calculate sd1i
audit_vgdaysweek_n1i <- colSums(!is.na(audit_yes_ds1_vgdaysweek))
#Calculate n1i
audit_no_ds1_vgdaysweek <- subset(audit_no_ds1, select = c(vgdaysweek))
#Subset 2i data
audit_vgdaysweek_m2i <- apply(audit_no_ds1_vgdaysweek, 2, mean)
#Calculate m2i
audit_vgdaysweek_sd2i <- apply(audit_no_ds1_vgdaysweek, 2, sd)
#Calculate sd2i
audit_vgdaysweek_n2i <- colSums(!is.na(audit_no_ds1_vgdaysweek))
#Calculate n2i
ds1audit_meta <- data.frame(m1i = audit_vgdaysweek_m1i, sd1i =
audit_vgdaysweek_sd1i, n1i = audit_vgdaysweek_n1i, m2i =
audit_vgdaysweek_m2i, sd2i = audit_vgdaysweek_sd2i, n2i =
audit_vgdaysweek_n2i) #MA dataframe
ds1audit_meta <- escalc(measure="MD", m1i = m1i, sd1i = sd1i, n1i = n1i,
m2i = m2i, sd2i = sd2i, n2i = n2i, data=ds1audit_meta) #Calculate yi and vi

#Study 2
audit_yes_ds2_vgdaysweek <- subset(audit_yes_ds2, select =
c(vgdaysweek)) #Subset 1i data
audit_vgdaysweek_m1i <- apply(audit_yes_ds2_vgdaysweek, 2, mean)
#Calculate m1i
audit_vgdaysweek_sd1i <- apply(audit_yes_ds2_vgdaysweek, 2, sd)
#Calculate sd1i
audit_vgdaysweek_n1i <- colSums(!is.na(audit_yes_ds2_vgdaysweek))
#Calculate n1i
audit_no_ds2_vgdaysweek <- subset(audit_no_ds2, select = c(vgdaysweek))
#Subset 2i data
audit_vgdaysweek_m2i <- apply(audit_no_ds2_vgdaysweek, 2, mean)
#Calculate m2i
audit_vgdaysweek_sd2i <- apply(audit_no_ds2_vgdaysweek, 2, sd)
#Calculate sd2i
audit_vgdaysweek_n2i <- colSums(!is.na(audit_no_ds2_vgdaysweek))
#Calculate n2i
ds2audit_meta <- data.frame(m1i = audit_vgdaysweek_m1i, sd1i =
audit_vgdaysweek_sd1i, n1i = audit_vgdaysweek_n1i, m2i =
audit_vgdaysweek_m2i, sd2i = audit_vgdaysweek_sd2i, n2i =
audit_vgdaysweek_n2i) #MA dataframe
ds2audit_meta <- escalc(measure="MD", m1i = m1i, sd1i = sd1i, n1i = n1i,
m2i = m2i, sd2i = sd2i, n2i = n2i, data=ds2audit_meta) #Calculate yi and vi

#FE meta-analysis
(audit_meta <- rbind(ds1audit_meta, ds2audit_meta))
```

```
##       m1i     sd1i n1i      m2i     sd2i n2i     yi     vi
## 1 5.218543 2.190568 151 4.884422 2.346652 199 0.3341 0.0595
## 2 4.804598 2.165623 174 4.688725 2.236325 408 0.1159 0.0392
```

```
(any_vgdaysweek_meta <- rma(yi=yi, vi=vi, measure="MD", method="FE",
data=audit_meta))
```

```
##
## Fixed-Effects Model (k = 2)
##
## Test for Heterogeneity:
## Q(df = 1) = 0.4828, p-val = 0.4872
##
## Model Results:
##
## estimate      se     zval     pval    ci.lb    ci.ub
##   0.2026   0.1537   1.3181   0.1875  -0.0987   0.5039
##
## ---
## Signif. codes:  0 '***' 0.001 '**' 0.01 '*' 0.05 '.' 0.1 ' ' 1
```

Cannabis screen

Replicated Effect

Hours per day

Study 1

```
(vghrsday_canscreen_ds1 <- t.test(vghrsday ~ canscreen, data=ds1))
```

```
##
##  Welch Two Sample t-test
##
## data:  vghrsday by canscreen
## t = 2.4308, df = 124.9, p-value = 0.01649
## alternative hypothesis: true difference in means is not equal to 0
## 95 percent confidence interval:
##  0.09212335 0.89950958
## sample estimates:
## mean in group 0 mean in group 1
##        2.403509        1.907692
```

Study 2

```
(vghrsday_canscreen_ds2 <- t.test(vghrsday ~ canscreen, data=ds2))
```

```
##
##  Welch Two Sample t-test
##
## data:  vghrsday by canscreen
## t = -1.4971, df = 161.89, p-value = 0.1363
## alternative hypothesis: true difference in means is not equal to 0
## 95 percent confidence interval:
##  -1.3548808  0.1863906
## sample estimates:
## mean in group 0 mean in group 1
##        3.415755        4.000000
```

Hours per week

Study 1

```
(vghrsweek_canscreen_ds1 <- t.test(vghrsweek ~ canscreen, data=ds1))

##
##  Welch Two Sample t-test
##
## data:  vghrsweek by canscreen
## t = 1.9612, df = 127.83, p-value = 0.05204
## alternative hypothesis: true difference in means is not equal to 0
## 95 percent confidence interval:
##  -0.02645986  5.94224934
## sample estimates:
## mean in group 0 mean in group 1
##        13.35789        10.40000
```

Study 2

```
(vghrsweek_canscreen_ds2 <- t.test(vghrsweek ~ canscreen, data=ds2))

##
##  Welch Two Sample t-test
##
## data:  vghrsweek by canscreen
## t = -1.4103, df = 160.96, p-value = 0.1604
## alternative hypothesis: true difference in means is not equal to 0
## 95 percent confidence interval:
##  -8.961668  1.494395
## sample estimates:
## mean in group 0 mean in group 1
##        17.56236        21.29600
```

Days per week

Study 1

```
(vgdaysweek_canscreen_ds1 <- t.test(vgdaysweek ~ canscreen, data=ds1))

##
##  Welch Two Sample t-test
##
## data:  vgdaysweek by canscreen
## t = -0.25413, df = 97.61, p-value = 0.7999
## alternative hypothesis: true difference in means is not equal to 0
## 95 percent confidence interval:
##  -0.6895237  0.5329785
## sample estimates:
## mean in group 0 mean in group 1
##        5.014035        5.092308
```

Study 2

```
(vgdaysweek_canscreen_ds2 <- t.test(vgdaysweek ~ canscreen, data=ds2))

##
##  Welch Two Sample t-test
##
## data:  vgdaysweek by canscreen
## t = -0.07094, df = 193.5, p-value = 0.9435
## alternative hypothesis: true difference in means is not equal to 0
## 95 percent confidence interval:
##  -0.4633574  0.4311824
## sample estimates:
## mean in group 0 mean in group 1
##        4.719912        4.736000
```

MD

Hours per day

```
#Study 1
canscreen_yes_ds1_vghrsday <- subset(canscreen_yes_ds1, select =
c(vghrsday)) #Subset 1i data
canscreen_vghrsday_m1i <- apply(canscreen_yes_ds1_vghrsday, 2, mean)
#Calculate m1i
canscreen_vghrsday_sd1i <- apply(canscreen_yes_ds1_vghrsday, 2, sd)
#Calculate sd1i
canscreen_vghrsday_n1i <- colSums(!is.na(canscreen_yes_ds1_vghrsday))
#Calculate n1i
canscreen_no_ds1_vghrsday <- subset(canscreen_no_ds1, select =
c(vghrsday)) #Subset 2i data
```

```
canscreen_vghrsday_m2i <- apply(canscreen_no_ds1_vghrsday, 2, mean)
#Calculate m2i
canscreen_vghrsday_sd2i <- apply(canscreen_no_ds1_vghrsday, 2, sd)
#Calculate sd2i
canscreen_vghrsday_n2i <- colSums(!is.na(canscreen_no_ds1_vghrsday))
#Calculate n2i
ds1canscreen_meta <- data.frame(m1i = canscreen_vghrsday_m1i, sd1i =
canscreen_vghrsday_sd1i, n1i = canscreen_vghrsday_n1i, m2i =
canscreen_vghrsday_m2i, sd2i = canscreen_vghrsday_sd2i, n2i =
canscreen_vghrsday_n2i) #MA dataframe
ds1canscreen_meta <- escalc(measure="MD", m1i = m1i, sd1i = sd1i, n1i =
n1i, m2i = m2i, sd2i = sd2i, n2i = n2i, data=ds1canscreen_meta) #Calculate
yi and vi

#Study 2
canscreen_yes_ds2_vghrsday <- subset(canscreen_yes_ds2, select =
c(vghrsday)) #Subset 1i data
canscreen_vghrsday_m1i <- apply(canscreen_yes_ds2_vghrsday, 2, mean)
#Calculate m1i
canscreen_vghrsday_sd1i <- apply(canscreen_yes_ds2_vghrsday, 2, sd)
#Calculate sd1i
canscreen_vghrsday_n1i <- colSums(!is.na(canscreen_yes_ds2_vghrsday))
#Calculate n1i
canscreen_no_ds2_vghrsday <- subset(canscreen_no_ds2, select =
c(vghrsday)) #Subset 2i data
canscreen_vghrsday_m2i <- apply(canscreen_no_ds2_vghrsday, 2, mean)
#Calculate m2i
canscreen_vghrsday_sd2i <- apply(canscreen_no_ds2_vghrsday, 2, sd)
#Calculate sd2i
canscreen_vghrsday_n2i <- colSums(!is.na(canscreen_no_ds2_vghrsday))
#Calculate n2i
ds2canscreen_meta <- data.frame(m1i = canscreen_vghrsday_m1i, sd1i =
canscreen_vghrsday_sd1i, n1i = canscreen_vghrsday_n1i, m2i =
canscreen_vghrsday_m2i, sd2i = canscreen_vghrsday_sd2i, n2i =
canscreen_vghrsday_n2i) #MA dataframe
ds2canscreen_meta <- escalc(measure="MD", m1i = m1i, sd1i = sd1i, n1i =
n1i, m2i = m2i, sd2i = sd2i, n2i = n2i, data=ds2canscreen_meta) #Calculate
yi and vi

#FE meta-analysis
(canscreen_meta <- rbind(ds1canscreen_meta, ds2canscreen_meta))

##        m1i     sd1i n1i      m2i     sd2i n2i      yi     vi
## 1 1.907692 1.377533  65 2.403509 1.880774 285 -0.4958 0.0416
## 2 4.000000 4.075893 125 3.415755 2.976887 457  0.5842 0.1523

(any_vghrsday_meta <- rma(yi=yi, vi=vi, measure="MD", method="FE",
data=canscreen_meta))

##
## Fixed-Effects Model (k = 2)
```

```
##
## Test for Heterogeneity:
## Q(df = 1) = 6.0162, p-val = 0.0142
##
## Model Results:
##
## estimate      se     zval     pval    ci.lb    ci.ub
##  -0.2641   0.1808  -1.4608   0.1441  -0.6184   0.0902
##
## ---
## Signif. codes:  0 '***' 0.001 '**' 0.01 '*' 0.05 '.' 0.1 ' ' 1
```

Hours per week

```
#Study 1
canscreen_yes_ds1_vghrsweek <- subset(canscreen_yes_ds1, select =
c(vghrsweek)) #Subset 1i data
canscreen_vghrsweek_m1i <- apply(canscreen_yes_ds1_vghrsweek, 2, mean)
#Calculate m1i
canscreen_vghrsweek_sd1i <- apply(canscreen_yes_ds1_vghrsweek, 2, sd)
#Calculate sd1i
canscreen_vghrsweek_n1i <- colSums(!is.na(canscreen_yes_ds1_vghrsweek))
#Calculate n1i
canscreen_no_ds1_vghrsweek <- subset(canscreen_no_ds1, select =
c(vghrsweek)) #Subset 2i data
canscreen_vghrsweek_m2i <- apply(canscreen_no_ds1_vghrsweek, 2, mean)
#Calculate m2i
canscreen_vghrsweek_sd2i <- apply(canscreen_no_ds1_vghrsweek, 2, sd)
#Calculate sd2i
canscreen_vghrsweek_n2i <- colSums(!is.na(canscreen_no_ds1_vghrsweek))
#Calculate n2i
ds1canscreen_meta <- data.frame(m1i = canscreen_vghrsweek_m1i, sd1i =
canscreen_vghrsweek_sd1i, n1i = canscreen_vghrsweek_n1i, m2i =
canscreen_vghrsweek_m2i, sd2i = canscreen_vghrsweek_sd2i, n2i =
canscreen_vghrsweek_n2i) #MA dataframe
ds1canscreen_meta <- escalc(measure="MD", m1i = m1i, sd1i = sd1i, n1i =
n1i, m2i = m2i, sd2i = sd2i, n2i = n2i, data=ds1canscreen_meta) #Calculate
yi and vi

#Study 2
canscreen_yes_ds2_vghrsweek <- subset(canscreen_yes_ds2, select =
c(vghrsweek)) #Subset 1i data
canscreen_vghrsweek_m1i <- apply(canscreen_yes_ds2_vghrsweek, 2, mean)
#Calculate m1i
canscreen_vghrsweek_sd1i <- apply(canscreen_yes_ds2_vghrsweek, 2, sd)
#Calculate sd1i
canscreen_vghrsweek_n1i <- colSums(!is.na(canscreen_yes_ds2_vghrsweek))
#Calculate n1i
canscreen_no_ds2_vghrsweek <- subset(canscreen_no_ds2, select =
```

```
c(vghrsweek)) #Subset 2i data
canscreen_vghrsweek_m2i <- apply(canscreen_no_ds2_vghrsweek, 2, mean)
#Calculate m2i
canscreen_vghrsweek_sd2i <- apply(canscreen_no_ds2_vghrsweek, 2, sd)
#Calculate sd2i
canscreen_vghrsweek_n2i <- colSums(!is.na(canscreen_no_ds2_vghrsweek))
#Calculate n2i
ds2canscreen_meta <- data.frame(m1i = canscreen_vghrsweek_m1i, sd1i =
canscreen_vghrsweek_sd1i, n1i = canscreen_vghrsweek_n1i, m2i =
canscreen_vghrsweek_m2i, sd2i = canscreen_vghrsweek_sd2i, n2i =
canscreen_vghrsweek_n2i) #MA dataframe
ds2canscreen_meta <- escalc(measure="MD", m1i = m1i, sd1i = sd1i, n1i =
n1i, m2i = m2i, sd2i = sd2i, n2i = n2i, data=ds2canscreen_meta) #Calculate
yi and vi

#FE meta-analysis
(canscreen_meta <- rbind(ds1canscreen_meta, ds2canscreen_meta))

##       m1i     sd1i n1i      m2i     sd2i n2i      yi     vi
## 1 10.400 10.11774  65 13.35789 14.12339 285 -2.9579 2.2748
## 2 21.296 27.69157 125 17.56236 19.98445 457  3.7336 7.0085

(any_vghrsweek_meta <- rma(yi=yi, vi=vi, measure="MD", method="FE",
data=canscreen_meta))

##
## Fixed-Effects Model (k = 2)
##
## Test for Heterogeneity:
## Q(df = 1) = 4.8234, p-val = 0.0281
##
## Model Results:
##
## estimate      se     zval    pval    ci.lb    ci.ub
##  -1.3182   1.3105  -1.0059   0.3145  -3.8867   1.2503
##
## ---
## Signif. codes:  0 '***' 0.001 '**' 0.01 '*' 0.05 '.' 0.1 ' ' 1
```

Days per week

```
#Study 1
canscreen_yes_ds1_vgdaysweek <- subset(canscreen_yes_ds1, select =
c(vgdaysweek)) #Subset 1i data
canscreen_vgdaysweek_m1i <- apply(canscreen_yes_ds1_vgdaysweek, 2,
mean) #Calculate m1i
canscreen_vgdaysweek_sd1i <- apply(canscreen_yes_ds1_vgdaysweek, 2, sd)
#Calculate sd1i
canscreen_vgdaysweek_n1i <- colSums(!
is.na(canscreen_yes_ds1_vgdaysweek)) #Calculate n1i
```

```
canscreen_no_ds1_vgdaysweek <- subset(canscreen_no_ds1, select =
c(vgdaysweek)) #Subset 2i data
canscreen_vgdaysweek_m2i <- apply(canscreen_no_ds1_vgdaysweek, 2,
mean) #Calculate m2i
canscreen_vgdaysweek_sd2i <- apply(canscreen_no_ds1_vgdaysweek, 2, sd)
#Calculate sd2i
canscreen_vgdaysweek_n2i <- colSums(!
is.na(canscreen_no_ds1_vgdaysweek)) #Calculate n2i
ds1canscreen_meta <- data.frame(m1i = canscreen_vgdaysweek_m1i, sd1i =
canscreen_vgdaysweek_sd1i, n1i = canscreen_vgdaysweek_n1i, m2i =
canscreen_vgdaysweek_m2i, sd2i = canscreen_vgdaysweek_sd2i, n2i =
canscreen_vgdaysweek_n2i) #MA dataframe
ds1canscreen_meta <- escalc(measure="MD", m1i = m1i, sd1i = sd1i, n1i =
n1i, m2i = m2i, sd2i = sd2i, n2i = n2i, data=ds1canscreen_meta) #Calculate
yi and vi

#Study 2
canscreen_yes_ds2_vgdaysweek <- subset(canscreen_yes_ds2, select =
c(vgdaysweek)) #Subset 1i data
canscreen_vgdaysweek_m1i <- apply(canscreen_yes_ds2_vgdaysweek, 2,
mean) #Calculate m1i
canscreen_vgdaysweek_sd1i <- apply(canscreen_yes_ds2_vgdaysweek, 2, sd)
#Calculate sd1i
canscreen_vgdaysweek_n1i <- colSums(!
is.na(canscreen_yes_ds2_vgdaysweek)) #Calculate n1i
canscreen_no_ds2_vgdaysweek <- subset(canscreen_no_ds2, select =
c(vgdaysweek)) #Subset 2i data
canscreen_vgdaysweek_m2i <- apply(canscreen_no_ds2_vgdaysweek, 2,
mean) #Calculate m2i
canscreen_vgdaysweek_sd2i <- apply(canscreen_no_ds2_vgdaysweek, 2, sd)
#Calculate sd2i
canscreen_vgdaysweek_n2i <- colSums(!
is.na(canscreen_no_ds2_vgdaysweek)) #Calculate n2i
ds2canscreen_meta <- data.frame(m1i = canscreen_vgdaysweek_m1i, sd1i =
canscreen_vgdaysweek_sd1i, n1i = canscreen_vgdaysweek_n1i, m2i =
canscreen_vgdaysweek_m2i, sd2i = canscreen_vgdaysweek_sd2i, n2i =
canscreen_vgdaysweek_n2i) #MA dataframe
ds2canscreen_meta <- escalc(measure="MD", m1i = m1i, sd1i = sd1i, n1i =
n1i, m2i = m2i, sd2i = sd2i, n2i = n2i, data=ds2canscreen_meta) #Calculate
yi and vi

#FE meta-analysis
(canscreen_meta <- rbind(ds1canscreen_meta, ds2canscreen_meta))

##        m1i     sd1i n1i      m2i     sd2i n2i     yi     vi
## 1 5.092308 2.227127  65 5.014035 2.299681 285 0.0783 0.0949
## 2 4.736000 2.258118 125 4.719912 2.204535 457 0.0161 0.0514

(any_vgdaysweek_meta <- rma(yi=yi, vi=vi, measure="MD", method="FE",
data=canscreen_meta))
```

```
##
## Fixed-Effects Model (k = 2)
##
## Test for Heterogeneity:
## Q(df = 1) = 0.0264, p-val = 0.8708
##
## Model Results:
##
## estimate      se     zval     pval    ci.lb    ci.ub
##   0.0379  0.1826  0.2078  0.8354  -0.3200  0.3959
##
## ---
## Signif. codes:  0 '***' 0.001 '**' 0.01 '*' 0.05 '.' 0.1 ' ' 1
```

Any care - Discharge

Replicated Effect

Hours per day

Study 1

```
(vghrsday_anycaredis_ds1 <- t.test(vghrsday ~ anydis, data=ds1anyscreen))

##
##  Welch Two Sample t-test
##
## data:  vghrsday by anydis
## t = -0.53622, df = 246.36, p-value = 0.5923
## alternative hypothesis: true difference in means is not equal to 0
## 95 percent confidence interval:
##  -0.5451046  0.3118150
## sample estimates:
## mean in group 0 mean in group 1
##        2.311927        2.428571
```

Study 2

```
(vghrsday_anycaredis_ds2 <- t.test(vghrsday ~ anydis, data=ds2anyscreen))

##
##  Welch Two Sample t-test
##
## data:  vghrsday by anydis
## t = -2.1194, df = 383.45, p-value = 0.0347
## alternative hypothesis: true difference in means is not equal to 0
## 95 percent confidence interval:
```

```
##  -1.27870365 -0.04794729
## sample estimates:
## mean in group 0 mean in group 1
##        3.201258        3.864583
```

Hours per week

Study 1

```
(vghrsweek_anycaredis_ds1 <- t.test(vghrsweek ~ anydis,
data=ds1anyscreen))

##
##  Welch Two Sample t-test
##
## data:  vghrsweek by anydis
## t = -0.54127, df = 243.38, p-value = 0.5888
## alternative hypothesis: true difference in means is not equal to 0
## 95 percent confidence interval:
##  -4.143338  2.357087
## sample estimates:
## mean in group 0 mean in group 1
##        12.89908        13.79221
```

Study 2

```
(vghrsweek_anycaredis_ds2 <- t.test(vghrsweek ~ anydis,
data=ds2anyscreen))

##
##  Welch Two Sample t-test
##
## data:  vghrsweek by anydis
## t = -2.6404, df = 400.5, p-value = 0.008604
## alternative hypothesis: true difference in means is not equal to 0
## 95 percent confidence interval:
##  -9.577712 -1.402503
## sample estimates:
## mean in group 0 mean in group 1
##        15.47170        20.96181
```

Days per week

Study 1

```
(vgdaysweek_anycaredis_ds1 <- t.test(vgdaysweek ~ anydis,
data=ds1anyscreen))
```

```
##
##  Welch Two Sample t-test
##
## data:  vgdaysweek by anydis
## t = -0.92016, df = 231.08, p-value = 0.3585
## alternative hypothesis: true difference in means is not equal to 0
## 95 percent confidence interval:
##  -0.8074883  0.2933694
## sample estimates:
## mean in group 0 mean in group 1
##        5.009174        5.266234
```

Study 2

```
(vgdaysweek_anycaredis_ds2 <- t.test(vgdaysweek ~ anydis, data=ds2anyscreen))
```

```
##
##  Welch Two Sample t-test
##
## data:  vgdaysweek by anydis
## t = -2.8132, df = 308.79, p-value = 0.005219
## alternative hypothesis: true difference in means is not equal to 0
## 95 percent confidence interval:
##  -1.0511242 -0.1859041
## sample estimates:
## mean in group 0 mean in group 1
##        4.371069        4.989583
```

MD

Hours per day

```
#Study 1
anycaredis_yes_ds1_vghrsday <- subset(anycaredis_yes_ds1, select = c(vghrsday)) #Subset 1i data
anycaredis_vghrsday_m1i <- apply(anycaredis_yes_ds1_vghrsday, 2, mean) #Calculate m1i
anycaredis_vghrsday_sd1i <- apply(anycaredis_yes_ds1_vghrsday, 2, sd) #Calculate sd1i
anycaredis_vghrsday_n1i <- colSums(!is.na(anycaredis_yes_ds1_vghrsday)) #Calculate n1i
anycaredis_no_ds1_vghrsday <- subset(anycaredis_no_ds1, select = c(vghrsday)) #Subset 2i data
anycaredis_vghrsday_m2i <- apply(anycaredis_no_ds1_vghrsday, 2, mean) #Calculate m2i
anycaredis_vghrsday_sd2i <- apply(anycaredis_no_ds1_vghrsday, 2, sd) #Calculate sd2i
```

```
anycaredis_vghrsday_n2i <- colSums(!is.na(anycaredis_no_ds1_vghrsday))
#Calculate n2i
ds1anycaredis_meta <- data.frame(m1i = anycaredis_vghrsday_m1i, sd1i =
anycaredis_vghrsday_sd1i, n1i = anycaredis_vghrsday_n1i, m2i =
anycaredis_vghrsday_m2i, sd2i = anycaredis_vghrsday_sd2i, n2i =
anycaredis_vghrsday_n2i) #MA dataframe
ds1anycaredis_meta <- escalc(measure="MD", m1i = m1i, sd1i = sd1i, n1i =
n1i, m2i = m2i, sd2i = sd2i, n2i = n2i, data=ds1anycaredis_meta)
#Calculate yi and vi

#Study 2
anycaredis_yes_ds2_vghrsday <- subset(anycaredis_yes_ds2, select =
c(vghrsday)) #Subset 1i data
anycaredis_vghrsday_m1i <- apply(anycaredis_yes_ds2_vghrsday, 2, mean)
#Calculate m1i
anycaredis_vghrsday_sd1i <- apply(anycaredis_yes_ds2_vghrsday, 2, sd)
#Calculate sd1i
anycaredis_vghrsday_n1i <- colSums(!is.na(anycaredis_yes_ds2_vghrsday))
#Calculate n1i
anycaredis_no_ds2_vghrsday <- subset(anycaredis_no_ds2, select =
c(vghrsday)) #Subset 2i data
anycaredis_vghrsday_m2i <- apply(anycaredis_no_ds2_vghrsday, 2, mean)
#Calculate m2i
anycaredis_vghrsday_sd2i <- apply(anycaredis_no_ds2_vghrsday, 2, sd)
#Calculate sd2i
anycaredis_vghrsday_n2i <- colSums(!is.na(anycaredis_no_ds2_vghrsday))
#Calculate n2i
ds2anycaredis_meta <- data.frame(m1i = anycaredis_vghrsday_m1i, sd1i =
anycaredis_vghrsday_sd1i, n1i = anycaredis_vghrsday_n1i, m2i =
anycaredis_vghrsday_m2i, sd2i = anycaredis_vghrsday_sd2i, n2i =
anycaredis_vghrsday_n2i) #MA dataframe
ds2anycaredis_meta <- escalc(measure="MD", m1i = m1i, sd1i = sd1i, n1i =
n1i, m2i = m2i, sd2i = sd2i, n2i = n2i, data=ds2anycaredis_meta)
#Calculate yi and vi

#FE meta-analysis
(anycaredis_meta <- rbind(ds1anycaredis_meta, ds2anycaredis_meta))

##        m1i     sd1i n1i      m2i     sd2i n2i     yi     vi
## 1 2.428571 1.843048 154 2.311927 1.659413 109 0.1166 0.0473
## 2 3.864583 3.575818 288 3.201258 2.918253 159 0.6633 0.0980

(anycaredis_vghrsday_meta <- rma(yi=yi, vi=vi, measure="MD",
method="FE", data=anycaredis_meta))

##
## Fixed-Effects Model (k = 2)
##
## Test for Heterogeneity:
## Q(df = 1) = 2.0571, p-val = 0.1515
##
```

```
## Model Results:
##
## estimate      se     zval     pval    ci.lb    ci.ub
##   0.2947   0.1786   1.6499   0.0990  -0.0554   0.6448       .
##
## ---
## Signif. codes:  0 '***' 0.001 '**' 0.01 '*' 0.05 '.' 0.1 ' ' 1
```

Hours per week

```
#Study 1
anycaredis_yes_ds1_vghrsweek <- subset(anycaredis_yes_ds1, select =
c(vghrsweek)) #Subset 1i data
anycaredis_vghrsweek_m1i <- apply(anycaredis_yes_ds1_vghrsweek, 2,
mean) #Calculate m1i
anycaredis_vghrsweek_sd1i <- apply(anycaredis_yes_ds1_vghrsweek, 2, sd)
#Calculate sd1i
anycaredis_vghrsweek_n1i <- colSums(!
is.na(anycaredis_yes_ds1_vghrsweek)) #Calculate n1i
anycaredis_no_ds1_vghrsweek <- subset(anycaredis_no_ds1, select =
c(vghrsweek)) #Subset 2i data
anycaredis_vghrsweek_m2i <- apply(anycaredis_no_ds1_vghrsweek, 2, mean)
#Calculate m2i
anycaredis_vghrsweek_sd2i <- apply(anycaredis_no_ds1_vghrsweek, 2, sd)
#Calculate sd2i
anycaredis_vghrsweek_n2i <- colSums(!is.na(anycaredis_no_ds1_vghrsweek))
#Calculate n2i
ds1anycaredis_meta <- data.frame(m1i = anycaredis_vghrsweek_m1i, sd1i =
anycaredis_vghrsweek_sd1i, n1i = anycaredis_vghrsweek_n1i, m2i =
anycaredis_vghrsweek_m2i, sd2i = anycaredis_vghrsweek_sd2i, n2i =
anycaredis_vghrsweek_n2i) #MA dataframe
ds1anycaredis_meta <- escalc(measure="MD", m1i = m1i, sd1i = sd1i, n1i =
n1i, m2i = m2i, sd2i = sd2i, n2i = n2i, data=ds1anycaredis_meta)
#Calculate yi and vi

#Study 2
anycaredis_yes_ds2_vghrsweek <- subset(anycaredis_yes_ds2, select =
c(vghrsweek)) #Subset 1i data
anycaredis_vghrsweek_m1i <- apply(anycaredis_yes_ds2_vghrsweek, 2,
mean) #Calculate m1i
anycaredis_vghrsweek_sd1i <- apply(anycaredis_yes_ds2_vghrsweek, 2, sd)
#Calculate sd1i
anycaredis_vghrsweek_n1i <- colSums(!
is.na(anycaredis_yes_ds2_vghrsweek)) #Calculate n1i
anycaredis_no_ds2_vghrsweek <- subset(anycaredis_no_ds2, select =
c(vghrsweek)) #Subset 2i data
anycaredis_vghrsweek_m2i <- apply(anycaredis_no_ds2_vghrsweek, 2, mean)
#Calculate m2i
anycaredis_vghrsweek_sd2i <- apply(anycaredis_no_ds2_vghrsweek, 2, sd)
```

```
#Calculate sd2i
anycaredis_vghrsweek_n2i <- colSums(!is.na(anycaredis_no_ds2_vghrsweek))
#Calculate n2i
ds2anycaredis_meta <- data.frame(m1i = anycaredis_vghrsweek_m1i, sd1i =
anycaredis_vghrsweek_sd1i, n1i = anycaredis_vghrsweek_n1i, m2i =
anycaredis_vghrsweek_m2i, sd2i = anycaredis_vghrsweek_sd2i, n2i =
anycaredis_vghrsweek_n2i) #MA dataframe
ds2anycaredis_meta <- escalc(measure="MD", m1i = m1i, sd1i = sd1i, n1i =
n1i, m2i = m2i, sd2i = sd2i, n2i = n2i, data=ds2anycaredis_meta)
#Calculate yi and vi

#FE meta-analysis
(anycaredis_meta <- rbind(ds1anycaredis_meta, ds2anycaredis_meta))

##        m1i     sd1i n1i      m2i     sd2i n2i     yi     vi
## 1 13.79221 13.79243 154 12.89908 12.73297 109 0.8931 2.7227
## 2 20.96181 24.58510 288 15.47170 18.80709 159 5.4901 4.3233

(anycaredis_vghrsweek_meta <- rma(yi=yi, vi=vi, measure="MD",
method="FE", data=anycaredis_meta))

##
## Fixed-Effects Model (k = 2)
##
## Test for Heterogeneity:
## Q(df = 1) = 2.9992, p-val = 0.0833
##
## Model Results:
##
## estimate      se     zval     pval    ci.lb    ci.ub
##   2.6695   1.2925   2.0653   0.0389   0.1362   5.2028        *
##
## ---
## Signif. codes:  0 '***' 0.001 '**' 0.01 '*' 0.05 '.' 0.1 ' ' 1
```

Days per week

```
#Study 1
anycaredis_yes_ds1_vgdaysweek <- subset(anycaredis_yes_ds1, select =
c(vgdaysweek)) #Subset 1i data
anycaredis_vgdaysweek_m1i <- apply(anycaredis_yes_ds1_vgdaysweek, 2,
mean) #Calculate m1i
anycaredis_vgdaysweek_sd1i <- apply(anycaredis_yes_ds1_vgdaysweek, 2,
sd) #Calculate sd1i
anycaredis_vgdaysweek_n1i <- colSums(!
is.na(anycaredis_yes_ds1_vgdaysweek)) #Calculate n1i
anycaredis_no_ds1_vgdaysweek <- subset(anycaredis_no_ds1, select =
c(vgdaysweek)) #Subset 2i data
anycaredis_vgdaysweek_m2i <- apply(anycaredis_no_ds1_vgdaysweek, 2,
mean) #Calculate m2i
```

```
anycaredis_vgdaysweek_sd2i <- apply(anycaredis_no_ds1_vgdaysweek, 2,
sd) #Calculate sd2i
anycaredis_vgdaysweek_n2i <- colSums(!
is.na(anycaredis_no_ds1_vgdaysweek)) #Calculate n2i
ds1anycaredis_meta <- data.frame(m1i = anycaredis_vgdaysweek_m1i, sd1i
= anycaredis_vgdaysweek_sd1i, n1i = anycaredis_vgdaysweek_n1i, m2i =
anycaredis_vgdaysweek_m2i, sd2i = anycaredis_vgdaysweek_sd2i, n2i =
anycaredis_vgdaysweek_n2i) #MA dataframe
ds1anycaredis_meta <- escalc(measure="MD", m1i = m1i, sd1i = sd1i, n1i =
n1i, m2i = m2i, sd2i = sd2i, n2i = n2i, data=ds1anycaredis_meta)
#Calculate yi and vi

#Study 2
anycaredis_yes_ds2_vgdaysweek <- subset(anycaredis_yes_ds2, select =
c(vgdaysweek)) #Subset 1i data
anycaredis_vgdaysweek_m1i <- apply(anycaredis_yes_ds2_vgdaysweek, 2,
mean) #Calculate m1i
anycaredis_vgdaysweek_sd1i <- apply(anycaredis_yes_ds2_vgdaysweek, 2,
sd) #Calculate sd1i
anycaredis_vgdaysweek_n1i <- colSums(!
is.na(anycaredis_yes_ds2_vgdaysweek)) #Calculate n1i
anycaredis_no_ds2_vgdaysweek <- subset(anycaredis_no_ds2, select =
c(vgdaysweek)) #Subset 2i data
anycaredis_vgdaysweek_m2i <- apply(anycaredis_no_ds2_vgdaysweek, 2,
mean) #Calculate m2i
anycaredis_vgdaysweek_sd2i <- apply(anycaredis_no_ds2_vgdaysweek, 2,
sd) #Calculate sd2i
anycaredis_vgdaysweek_n2i <- colSums(!
is.na(anycaredis_no_ds2_vgdaysweek)) #Calculate n2i
ds2anycaredis_meta <- data.frame(m1i = anycaredis_vgdaysweek_m1i, sd1i
= anycaredis_vgdaysweek_sd1i, n1i = anycaredis_vgdaysweek_n1i, m2i =
anycaredis_vgdaysweek_m2i, sd2i = anycaredis_vgdaysweek_sd2i, n2i =
anycaredis_vgdaysweek_n2i) #MA dataframe
ds2anycaredis_meta <- escalc(measure="MD", m1i = m1i, sd1i = sd1i, n1i =
n1i, m2i = m2i, sd2i = sd2i, n2i = n2i, data=ds2anycaredis_meta)
#Calculate yi and vi

#FE meta-analysis
(anycaredis_meta <- rbind(ds1anycaredis_meta, ds2anycaredis_meta))

##        m1i     sd1i n1i      m2i     sd2i n2i     yi     vi
## 1 5.266234 2.217112 154 5.009174 2.242252 109 0.2571 0.0780
## 2 4.989583 2.133986 288 4.371069 2.274107 159 0.6185 0.0483

(anycaredis_vgdaysweek_meta <- rma(yi=yi, vi=vi, measure="MD",
method="FE", data=anycaredis_meta))

##
## Fixed-Effects Model (k = 2)
##
## Test for Heterogeneity:
```

```
## Q(df = 1) = 1.0338, p-val = 0.3093
##
## Model Results:
##
## estimate      se     zval     pval    ci.lb    ci.ub
##   0.4803  0.1728  2.7798  0.0054  0.1416  0.8189     **
##
## ---
## Signif. codes:  0 '***' 0.001 '**' 0.01 '*' 0.05 '.' 0.1 ' ' 1
```

Any care - Year

Replicated Effect

Hours per day

Study 1

```
(vghrsday_anycareyear_ds1 <- t.test(vghrsday ~ anyyear,
data=ds1anyscreen))
```

```
##
##  Welch Two Sample t-test
##
## data:  vghrsday by anyyear
## t = 0.82714, df = 220.22, p-value = 0.4091
## alternative hypothesis: true difference in means is not equal to 0
## 95 percent confidence interval:
##  -0.2534314  0.6200125
## sample estimates:
## mean in group 0 mean in group 1
##        2.450617        2.267327
```

Study 2

```
(vghrsday_anycareyear_ds2 <- t.test(vghrsday ~ anyyear,
data=ds2anyscreen))
```

```
##
##  Welch Two Sample t-test
##
## data:  vghrsday by anyyear
## t = -1.6325, df = 404.16, p-value = 0.1034
## alternative hypothesis: true difference in means is not equal to 0
## 95 percent confidence interval:
##  -1.1610420  0.1075661
## sample estimates:
```

```
## mean in group 0 mean in group 1
##        3.382353        3.909091
```

Hours per week

Study 1

```
(vghrsweek_anycareyear_ds1 <- t.test(vghrsweek ~ anyyear, data=ds1anyscreen))
```

```
##
##  Welch Two Sample t-test
##
## data:  vghrsweek by anyyear
## t = 0.9737, df = 225.04, p-value = 0.3312
## alternative hypothesis: true difference in means is not equal to 0
## 95 percent confidence interval:
##  -1.655933  4.890868
## sample estimates:
## mean in group 0 mean in group 1
##        14.04321        12.42574
```

Study 2

```
(vghrsweek_anycareyear_ds2 <- t.test(vghrsweek ~ anyyear, data=ds2anyscreen))
```

```
##
##  Welch Two Sample t-test
##
## data:  vghrsweek by anyyear
## t = -1.6053, df = 400.13, p-value = 0.1092
## alternative hypothesis: true difference in means is not equal to 0
## 95 percent confidence interval:
##  -7.8192419  0.7895688
## sample estimates:
## mean in group 0 mean in group 1
##        17.36555        20.88038
```

Days per week

Study 1

```
(vgdaysweek_anycareyear_ds1 <- t.test(vgdaysweek ~ anyyear, data=ds1anyscreen))
```

```
##
##  Welch Two Sample t-test
```

```
##
## data:  vgdaysweek by anyyear
## t = 0.12071, df = 210.66, p-value = 0.904
## alternative hypothesis: true difference in means is not equal to 0
## 95 percent confidence interval:
##  -0.5247097  0.5931610
## sample estimates:
## mean in group 0 mean in group 1
##        5.172840        5.138614
```

Study 2

```
(vgdaysweek_anycareyear_ds2 <- t.test(vgdaysweek ~ anyyear,
data=ds2anyscreen))
```

```
##
##  Welch Two Sample t-test
##
## data:  vgdaysweek by anyyear
## t = -1.2168, df = 442.3, p-value = 0.2243
## alternative hypothesis: true difference in means is not equal to 0
## 95 percent confidence interval:
##  -0.6617582  0.1556667
## sample estimates:
## mean in group 0 mean in group 1
##        4.651261        4.904306
```

MD

Hours per day

```
#Study 1
anycareyear_yes_ds1_vghrsday <- subset(anycareyear_yes_ds1, select =
c(vghrsday)) #Subset 1i data
anycareyear_vghrsday_m1i <- apply(anycareyear_yes_ds1_vghrsday, 2,
mean) #Calculate m1i
anycareyear_vghrsday_sd1i <- apply(anycareyear_yes_ds1_vghrsday, 2, sd)
#Calculate sd1i
anycareyear_vghrsday_n1i <- colSums(!
is.na(anycareyear_yes_ds1_vghrsday)) #Calculate n1i
anycareyear_no_ds1_vghrsday <- subset(anycareyear_no_ds1, select =
c(vghrsday)) #Subset 2i data
anycareyear_vghrsday_m2i <- apply(anycareyear_no_ds1_vghrsday, 2,
mean) #Calculate m2i
anycareyear_vghrsday_sd2i <- apply(anycareyear_no_ds1_vghrsday, 2, sd)
#Calculate sd2i
anycareyear_vghrsday_n2i <- colSums(!is.na(anycareyear_no_ds1_vghrsday))
#Calculate n2i
```

```
ds1anycareyear_meta <- data.frame(m1i = anycareyear_vghrsday_m1i, sd1i
= anycareyear_vghrsday_sd1i, n1i = anycareyear_vghrsday_n1i, m2i =
anycareyear_vghrsday_m2i, sd2i = anycareyear_vghrsday_sd2i, n2i =
anycareyear_vghrsday_n2i) #MA dataframe
ds1anycareyear_meta <- escalc(measure="MD", m1i = m1i, sd1i = sd1i, n1i
= n1i, m2i = m2i, sd2i = sd2i, n2i = n2i, data=ds1anycareyear_meta)
#Calculate yi and vi

#Study 2
anycareyear_yes_ds2_vghrsday <- subset(anycareyear_yes_ds2, select =
c(vghrsday)) #Subset 1i data
anycareyear_vghrsday_m1i <- apply(anycareyear_yes_ds2_vghrsday, 2,
mean) #Calculate m1i
anycareyear_vghrsday_sd1i <- apply(anycareyear_yes_ds2_vghrsday, 2, sd)
#Calculate sd1i
anycareyear_vghrsday_n1i <- colSums(!
is.na(anycareyear_yes_ds2_vghrsday)) #Calculate n1i
anycareyear_no_ds2_vghrsday <- subset(anycareyear_no_ds2, select =
c(vghrsday)) #Subset 2i data
anycareyear_vghrsday_m2i <- apply(anycareyear_no_ds2_vghrsday, 2,
mean) #Calculate m2i
anycareyear_vghrsday_sd2i <- apply(anycareyear_no_ds2_vghrsday, 2, sd)
#Calculate sd2i
anycareyear_vghrsday_n2i <- colSums(!is.na(anycareyear_no_ds2_vghrsday))
#Calculate n2i
ds2anycareyear_meta <- data.frame(m1i = anycareyear_vghrsday_m1i, sd1i
= anycareyear_vghrsday_sd1i, n1i = anycareyear_vghrsday_n1i, m2i =
anycareyear_vghrsday_m2i, sd2i = anycareyear_vghrsday_sd2i, n2i =
anycareyear_vghrsday_n2i) #MA dataframe
ds2anycareyear_meta <- escalc(measure="MD", m1i = m1i, sd1i = sd1i, n1i
= n1i, m2i = m2i, sd2i = sd2i, n2i = n2i, data=ds2anycareyear_meta)
#Calculate yi and vi

#FE meta-analysis
(anycareyear_meta <- rbind(ds1anycareyear_meta, ds2anycareyear_meta))

##        m1i     sd1i n1i      m2i     sd2i n2i      yi     vi
## 1 2.267327 1.714008 101 2.450617 1.800802 162 -0.1833 0.0491
## 2 3.909091 3.690732 209 3.382353 3.044124 238  0.5267 0.1041

(anycareyear_vghrsday_meta <- rma(yi=yi, vi=vi, measure="MD",
method="FE", data=anycareyear_meta))

##
## Fixed-Effects Model (k = 2)
##
## Test for Heterogeneity:
## Q(df = 1) = 3.2904, p-val = 0.0697
##
## Model Results:
##
```

```
## estimate      se     zval     pval    ci.lb    ci.ub
##   0.0443   0.1827   0.2424   0.8085  -0.3137   0.4023
##
## ---
## Signif. codes:  0 '***' 0.001 '**' 0.01 '*' 0.05 '.' 0.1 ' ' 1
```

Hours per week

```
#Study 1
anycareyear_yes_ds1_vghrsweek <- subset(anycareyear_yes_ds1, select =
c(vghrsweek)) #Subset 1i data
anycareyear_vghrsweek_m1i <- apply(anycareyear_yes_ds1_vghrsweek, 2,
mean) #Calculate m1i
anycareyear_vghrsweek_sd1i <- apply(anycareyear_yes_ds1_vghrsweek, 2,
sd) #Calculate sd1i
anycareyear_vghrsweek_n1i <- colSums(!
is.na(anycareyear_yes_ds1_vghrsweek)) #Calculate n1i
anycareyear_no_ds1_vghrsweek <- subset(anycareyear_no_ds1, select =
c(vghrsweek)) #Subset 2i data
anycareyear_vghrsweek_m2i <- apply(anycareyear_no_ds1_vghrsweek, 2,
mean) #Calculate m2i
anycareyear_vghrsweek_sd2i <- apply(anycareyear_no_ds1_vghrsweek, 2,
sd) #Calculate sd2i
anycareyear_vghrsweek_n2i <- colSums(!
is.na(anycareyear_no_ds1_vghrsweek)) #Calculate n2i
ds1anycareyear_meta <- data.frame(m1i = anycareyear_vghrsweek_m1i,
sd1i = anycareyear_vghrsweek_sd1i, n1i = anycareyear_vghrsweek_n1i, m2i
= anycareyear_vghrsweek_m2i, sd2i = anycareyear_vghrsweek_sd2i, n2i =
anycareyear_vghrsweek_n2i) #MA dataframe
ds1anycareyear_meta <- escalc(measure="MD", m1i = m1i, sd1i = sd1i, n1i
= n1i, m2i = m2i, sd2i = sd2i, n2i = n2i, data=ds1anycareyear_meta)
#Calculate yi and vi

#Study 2
anycareyear_yes_ds2_vghrsweek <- subset(anycareyear_yes_ds2, select =
c(vghrsweek)) #Subset 1i data
anycareyear_vghrsweek_m1i <- apply(anycareyear_yes_ds2_vghrsweek, 2,
mean) #Calculate m1i
anycareyear_vghrsweek_sd1i <- apply(anycareyear_yes_ds2_vghrsweek, 2,
sd) #Calculate sd1i
anycareyear_vghrsweek_n1i <- colSums(!
is.na(anycareyear_yes_ds2_vghrsweek)) #Calculate n1i
anycareyear_no_ds2_vghrsweek <- subset(anycareyear_no_ds2, select =
c(vghrsweek)) #Subset 2i data
anycareyear_vghrsweek_m2i <- apply(anycareyear_no_ds2_vghrsweek, 2,
mean) #Calculate m2i
anycareyear_vghrsweek_sd2i <- apply(anycareyear_no_ds2_vghrsweek, 2,
sd) #Calculate sd2i
anycareyear_vghrsweek_n2i <- colSums(!
```

```
is.na(anycareyear_no_ds2_vghrsweek)) #Calculate n2i
ds2anycareyear_meta <- data.frame(m1i = anycareyear_vghrsweek_m1i,
sd1i = anycareyear_vghrsweek_sd1i, n1i = anycareyear_vghrsweek_n1i, m2i
= anycareyear_vghrsweek_m2i, sd2i = anycareyear_vghrsweek_sd2i, n2i =
anycareyear_vghrsweek_n2i) #MA dataframe
ds2anycareyear_meta <- escalc(measure="MD", m1i = m1i, sd1i = sd1i, n1i
= n1i, m2i = m2i, sd2i = sd2i, n2i = n2i, data=ds2anycareyear_meta)
#Calculate yi and vi

#FE meta-analysis
(anycareyear_meta <- rbind(ds1anycareyear_meta, ds2anycareyear_meta))

##        m1i     sd1i n1i      m2i     sd2i n2i      yi     vi
## 1 12.42574 12.68649 101 14.04321 13.74314 162 -1.6175 2.7594
## 2 20.88038 25.21351 209 17.36555 20.42163 238  3.5148 4.7940

(anycareyear_vghrsweek_meta <- rma(yi=yi, vi=vi, measure="MD",
method="FE", data=anycareyear_meta))

##
## Fixed-Effects Model (k = 2)
##
## Test for Heterogeneity:
## Q(df = 1) = 3.4872, p-val = 0.0618
##
## Model Results:
##
## estimate      se     zval     pval    ci.lb    ci.ub
##   0.2575   1.3234   0.1946   0.8457  -2.3363   2.8513
##
## ---
## Signif. codes:  0 '***' 0.001 '**' 0.01 '*' 0.05 '.' 0.1 ' ' 1
```

Days per week

```
#Study 1
anycareyear_yes_ds1_vgdaysweek <- subset(anycareyear_yes_ds1, select =
c(vgdaysweek)) #Subset 1i data
anycareyear_vgdaysweek_m1i <- apply(anycareyear_yes_ds1_vgdaysweek,
2, mean) #Calculate m1i
anycareyear_vgdaysweek_sd1i <- apply(anycareyear_yes_ds1_vgdaysweek,
2, sd) #Calculate sd1i
anycareyear_vgdaysweek_n1i <- colSums(!
is.na(anycareyear_yes_ds1_vgdaysweek)) #Calculate n1i
anycareyear_no_ds1_vgdaysweek <- subset(anycareyear_no_ds1, select =
c(vgdaysweek)) #Subset 2i data
anycareyear_vgdaysweek_m2i <- apply(anycareyear_no_ds1_vgdaysweek, 2,
mean) #Calculate m2i
anycareyear_vgdaysweek_sd2i <- apply(anycareyear_no_ds1_vgdaysweek, 2,
sd) #Calculate sd2i
```

```
anycareyear_vgdaysweek_n2i <- colSums(!
is.na(anycareyear_no_ds1_vgdaysweek)) #Calculate n2i
ds1anycareyear_meta <- data.frame(m1i = anycareyear_vgdaysweek_m1i,
sd1i = anycareyear_vgdaysweek_sd1i, n1i = anycareyear_vgdaysweek_n1i,
m2i = anycareyear_vgdaysweek_m2i, sd2i = anycareyear_vgdaysweek_sd2i,
n2i = anycareyear_vgdaysweek_n2i) #MA dataframe
ds1anycareyear_meta <- escalc(measure="MD", m1i = m1i, sd1i = sd1i, n1i
= n1i, m2i = m2i, sd2i = sd2i, n2i = n2i, data=ds1anycareyear_meta)
#Calculate yi and vi

#Study 2
anycareyear_yes_ds2_vgdaysweek <- subset(anycareyear_yes_ds2, select =
c(vgdaysweek)) #Subset 1i data
anycareyear_vgdaysweek_m1i <- apply(anycareyear_yes_ds2_vgdaysweek,
2, mean) #Calculate m1i
anycareyear_vgdaysweek_sd1i <- apply(anycareyear_yes_ds2_vgdaysweek,
2, sd) #Calculate sd1i
anycareyear_vgdaysweek_n1i <- colSums(!
is.na(anycareyear_yes_ds2_vgdaysweek)) #Calculate n1i
anycareyear_no_ds2_vgdaysweek <- subset(anycareyear_no_ds2, select =
c(vgdaysweek)) #Subset 2i data
anycareyear_vgdaysweek_m2i <- apply(anycareyear_no_ds2_vgdaysweek, 2,
mean) #Calculate m2i
anycareyear_vgdaysweek_sd2i <- apply(anycareyear_no_ds2_vgdaysweek, 2,
sd) #Calculate sd2i
anycareyear_vgdaysweek_n2i <- colSums(!
is.na(anycareyear_no_ds2_vgdaysweek)) #Calculate n2i
ds2anycareyear_meta <- data.frame(m1i = anycareyear_vgdaysweek_m1i,
sd1i = anycareyear_vgdaysweek_sd1i, n1i = anycareyear_vgdaysweek_n1i,
m2i = anycareyear_vgdaysweek_m2i, sd2i = anycareyear_vgdaysweek_sd2i,
n2i = anycareyear_vgdaysweek_n2i) #MA dataframe
ds2anycareyear_meta <- escalc(measure="MD", m1i = m1i, sd1i = sd1i, n1i
= n1i, m2i = m2i, sd2i = sd2i, n2i = n2i, data=ds2anycareyear_meta)
#Calculate yi and vi

#FE meta-analysis
(anycareyear_meta <- rbind(ds1anycareyear_meta, ds2anycareyear_meta))

##        m1i     sd1i n1i      m2i     sd2i n2i      yi     vi
## 1 5.138614 2.245127 101 5.172840 2.222360 162 -0.0342 0.0804
## 2 4.904306 2.139471 209 4.651261 2.253974 238  0.2530 0.0432

(anycareyear_vgdaysweek_meta <- rma(yi=yi, vi=vi, measure="MD",
method="FE", data=anycareyear_meta))

##
## Fixed-Effects Model (k = 2)
##
## Test for Heterogeneity:
## Q(df = 1) = 0.6675, p-val = 0.4139
##
```

```
## Model Results:
##
## estimate      se     zval     pval    ci.lb    ci.ub
##   0.1526   0.1677   0.9098   0.3629  -0.1761   0.4812
##
## ---
## Signif. codes:  0 '***' 0.001 '**' 0.01 '*' 0.05 '.' 0.1 ' ' 1
```

Any care - Month

Replicated Effect

Hours per day

Study 1

```
(vghrsday_anycaremonth_ds1 <- t.test(vghrsday ~ anymonth,
data=ds1anyscreen))
```

```
##
##  Welch Two Sample t-test
##
## data:  vghrsday by anymonth
## t = -1.6981, df = 42.665, p-value = 0.09678
## alternative hypothesis: true difference in means is not equal to 0
## 95 percent confidence interval:
##  -1.3598088  0.1167838
## sample estimates:
## mean in group 0 mean in group 1
##        2.295154        2.916667
```

Study 2

```
(vghrsday_anycaremonth_ds2 <- t.test(vghrsday ~ anymonth,
data=ds2anyscreen))
```

```
##
##  Welch Two Sample t-test
##
## data:  vghrsday by anymonth
## t = 0.3906, df = 172.75, p-value = 0.6966
## alternative hypothesis: true difference in means is not equal to 0
## 95 percent confidence interval:
##  -0.5400568  0.8065367
## sample estimates:
## mean in group 0 mean in group 1
##        3.655462        3.522222
```

Hours per week

Study 1

```
(vghrsweek_anycaremonth_ds1 <- t.test(vghrsweek ~ anymonth,
data=ds1anyscreen))

##
##  Welch Two Sample t-test
##
## data:  vghrsweek by anymonth
## t = -1.25, df = 42.65, p-value = 0.2181
## alternative hypothesis: true difference in means is not equal to 0
## 95 percent confidence interval:
##  -9.068652  2.129592
## sample estimates:
## mean in group 0 mean in group 1
##        12.94714        16.41667
```

Study 2

```
(vghrsweek_anycaremonth_ds2 <- t.test(vghrsweek ~ anymonth,
data=ds2anyscreen))

##
##  Welch Two Sample t-test
##
## data:  vghrsweek by anymonth
## t = 0.38076, df = 170.23, p-value = 0.7039
## alternative hypothesis: true difference in means is not equal to 0
## 95 percent confidence interval:
##  -3.714307  5.489657
## sample estimates:
## mean in group 0 mean in group 1
##        19.18768        18.30000
```

Days per week

Study 1

```
(vgdaysweek_anycaremonth_ds1 <- t.test(vgdaysweek ~ anymonth,
data=ds1anyscreen))

##
##  Welch Two Sample t-test
##
## data:  vgdaysweek by anymonth
## t = -0.25859, df = 46.433, p-value = 0.7971
```

```
## alternative hypothesis: true difference in means is not equal to 0
## 95 percent confidence interval:
##  -0.9188367  0.7095856
## sample estimates:
## mean in group 0 mean in group 1
##        5.145374        5.250000
```

Study 2

```
(vgdaysweek_anycaremonth_ds2 <- t.test(vgdaysweek ~ anymonth,
data=ds2anyscreen))
```

```
##
##  Welch Two Sample t-test
##
## data:  vgdaysweek by anymonth
## t = -1.2864, df = 147.57, p-value = 0.2003
## alternative hypothesis: true difference in means is not equal to 0
## 95 percent confidence interval:
##  -0.8023105  0.1696308
## sample estimates:
## mean in group 0 mean in group 1
##        4.705882        5.022222
```

MD

Hours per day

```
#Study 1
anycaremonth_yes_ds1_vghrsday <- subset(anycaremonth_yes_ds1, select =
c(vghrsday)) #Subset 1i data
anycaremonth_vghrsday_m1i <- apply(anycaremonth_yes_ds1_vghrsday, 2,
mean) #Calculate m1i
anycaremonth_vghrsday_sd1i <- apply(anycaremonth_yes_ds1_vghrsday, 2,
sd) #Calculate sd1i
anycaremonth_vghrsday_n1i <- colSums(!
is.na(anycaremonth_yes_ds1_vghrsday)) #Calculate n1i
anycaremonth_no_ds1_vghrsday <- subset(anycaremonth_no_ds1, select =
c(vghrsday)) #Subset 2i data
anycaremonth_vghrsday_m2i <- apply(anycaremonth_no_ds1_vghrsday, 2,
mean) #Calculate m2i
anycaremonth_vghrsday_sd2i <- apply(anycaremonth_no_ds1_vghrsday, 2,
sd) #Calculate sd2i
anycaremonth_vghrsday_n2i <- colSums(!
is.na(anycaremonth_no_ds1_vghrsday)) #Calculate n2i
ds1anycaremonth_meta <- data.frame(m1i = anycaremonth_vghrsday_m1i,
sd1i = anycaremonth_vghrsday_sd1i, n1i = anycaremonth_vghrsday_n1i, m2i
= anycaremonth_vghrsday_m2i, sd2i = anycaremonth_vghrsday_sd2i, n2i =
```

```
anycaremonth_vghrsday_n2i) #MA dataframe
ds1anycaremonth_meta <- escalc(measure="MD", m1i = m1i, sd1i = sd1i,
n1i = n1i, m2i = m2i, sd2i = sd2i, n2i = n2i, data=ds1anycaremonth_meta)
#Calculate yi and vi

#Study 2
anycaremonth_yes_ds2_vghrsday <- subset(anycaremonth_yes_ds2, select =
c(vghrsday)) #Subset 1i data
anycaremonth_vghrsday_m1i <- apply(anycaremonth_yes_ds2_vghrsday, 2,
mean) #Calculate m1i
anycaremonth_vghrsday_sd1i <- apply(anycaremonth_yes_ds2_vghrsday, 2,
sd) #Calculate sd1i
anycaremonth_vghrsday_n1i <- colSums(!
is.na(anycaremonth_yes_ds2_vghrsday)) #Calculate n1i
anycaremonth_no_ds2_vghrsday <- subset(anycaremonth_no_ds2, select =
c(vghrsday)) #Subset 2i data
anycaremonth_vghrsday_m2i <- apply(anycaremonth_no_ds2_vghrsday, 2,
mean) #Calculate m2i
anycaremonth_vghrsday_sd2i <- apply(anycaremonth_no_ds2_vghrsday, 2,
sd) #Calculate sd2i
anycaremonth_vghrsday_n2i <- colSums(!
is.na(anycaremonth_no_ds2_vghrsday)) #Calculate n2i
ds2anycaremonth_meta <- data.frame(m1i = anycaremonth_vghrsday_m1i,
sd1i = anycaremonth_vghrsday_sd1i, n1i = anycaremonth_vghrsday_n1i, m2i
= anycaremonth_vghrsday_m2i, sd2i = anycaremonth_vghrsday_sd2i, n2i =
anycaremonth_vghrsday_n2i) #MA dataframe
ds2anycaremonth_meta <- escalc(measure="MD", m1i = m1i, sd1i = sd1i,
n1i = n1i, m2i = m2i, sd2i = sd2i, n2i = n2i, data=ds2anycaremonth_meta)
#Calculate yi and vi

#FE meta-analysis
(anycaremonth_meta <- rbind(ds1anycaremonth_meta,
ds2anycaremonth_meta))

##        m1i     sd1i n1i      m2i     sd2i n2i      yi     vi
## 1 2.916667 2.089087  36 2.295154 1.700110 227  0.6215 0.1340
## 2 3.522222 2.711721  90 3.655462 3.517461 357 -0.1332 0.1164

(anycaremonth_vghrsday_meta <- rma(yi=yi, vi=vi, measure="MD",
method="FE", data=anycaremonth_meta))

##
## Fixed-Effects Model (k = 2)
##
## Test for Heterogeneity:
## Q(df = 1) = 2.2756, p-val = 0.1314
##
## Model Results:
##
## estimate      se     zval     pval    ci.lb    ci.ub
##   0.2176  0.2495   0.8720   0.3832  -0.2715   0.7067
```

```
##
## ---
## Signif. codes:  0 '***' 0.001 '**' 0.01 '*' 0.05 '.' 0.1 ' ' 1
```

Hours per week

```
#Study 1
anycaremonth_yes_ds1_vghrsweek <- subset(anycaremonth_yes_ds1, select
= c(vghrsweek)) #Subset 1i data
anycaremonth_vghrsweek_m1i <- apply(anycaremonth_yes_ds1_vghrsweek,
2, mean) #Calculate m1i
anycaremonth_vghrsweek_sd1i <- apply(anycaremonth_yes_ds1_vghrsweek,
2, sd) #Calculate sd1i
anycaremonth_vghrsweek_n1i <- colSums(!
is.na(anycaremonth_yes_ds1_vghrsweek)) #Calculate n1i
anycaremonth_no_ds1_vghrsweek <- subset(anycaremonth_no_ds1, select =
c(vghrsweek)) #Subset 2i data
anycaremonth_vghrsweek_m2i <- apply(anycaremonth_no_ds1_vghrsweek,
2, mean) #Calculate m2i
anycaremonth_vghrsweek_sd2i <- apply(anycaremonth_no_ds1_vghrsweek,
2, sd) #Calculate sd2i
anycaremonth_vghrsweek_n2i <- colSums(!
is.na(anycaremonth_no_ds1_vghrsweek)) #Calculate n2i
ds1anycaremonth_meta <- data.frame(m1i = anycaremonth_vghrsweek_m1i,
sd1i = anycaremonth_vghrsweek_sd1i, n1i = anycaremonth_vghrsweek_n1i,
m2i = anycaremonth_vghrsweek_m2i, sd2i = anycaremonth_vghrsweek_sd2i,
n2i = anycaremonth_vghrsweek_n2i) #MA dataframe
ds1anycaremonth_meta <- escalc(measure="MD", m1i = m1i, sd1i = sd1i,
n1i = n1i, m2i = m2i, sd2i = sd2i, n2i = n2i, data=ds1anycaremonth_meta)
#Calculate yi and vi

#Study 2
anycaremonth_yes_ds2_vghrsweek <- subset(anycaremonth_yes_ds2, select
= c(vghrsweek)) #Subset 1i data
anycaremonth_vghrsweek_m1i <- apply(anycaremonth_yes_ds2_vghrsweek,
2, mean) #Calculate m1i
anycaremonth_vghrsweek_sd1i <- apply(anycaremonth_yes_ds2_vghrsweek,
2, sd) #Calculate sd1i
anycaremonth_vghrsweek_n1i <- colSums(!
is.na(anycaremonth_yes_ds2_vghrsweek)) #Calculate n1i
anycaremonth_no_ds2_vghrsweek <- subset(anycaremonth_no_ds2, select =
c(vghrsweek)) #Subset 2i data
anycaremonth_vghrsweek_m2i <- apply(anycaremonth_no_ds2_vghrsweek,
2, mean) #Calculate m2i
anycaremonth_vghrsweek_sd2i <- apply(anycaremonth_no_ds2_vghrsweek,
2, sd) #Calculate sd2i
anycaremonth_vghrsweek_n2i <- colSums(!
is.na(anycaremonth_no_ds2_vghrsweek)) #Calculate n2i
ds2anycaremonth_meta <- data.frame(m1i = anycaremonth_vghrsweek_m1i,
```

```
sd1i = anycaremonth_vghrsweek_sd1i, n1i = anycaremonth_vghrsweek_n1i,
m2i = anycaremonth_vghrsweek_m2i, sd2i = anycaremonth_vghrsweek_sd2i,
n2i = anycaremonth_vghrsweek_n2i) #MA dataframe
ds2anycaremonth_meta <- escalc(measure="MD", m1i = m1i, sd1i = sd1i,
n1i = n1i, m2i = m2i, sd2i = sd2i, n2i = n2i, data=ds2anycaremonth_meta)
#Calculate yi and vi

#FE meta-analysis
(anycaremonth_meta <- rbind(ds1anycaremonth_meta,
ds2anycaremonth_meta))

##         m1i     sd1i n1i      m2i     sd2i n2i      yi     vi
## 1 16.41667 15.84456  36 12.94714 12.88227 227  3.4695 7.7047
## 2 18.30000 18.61222  90 19.18768 23.79432 357 -0.8877 5.4350

(anycaremonth_vghrsweek_meta <- rma(yi=yi, vi=vi, measure="MD",
method="FE", data=anycaremonth_meta))

##
## Fixed-Effects Model (k = 2)
##
## Test for Heterogeneity:
## Q(df = 1) = 1.4449, p-val = 0.2294
##
## Model Results:
##
## estimate       se     zval     pval    ci.lb    ci.ub
##   0.9146   1.7852   0.5123   0.6084  -2.5843   4.4135
##
## ---
## Signif. codes:  0 '***' 0.001 '**' 0.01 '*' 0.05 '.' 0.1 ' ' 1
```

Days per week

```
#Study 1
anycaremonth_yes_ds1_vgdaysweek <- subset(anycaremonth_yes_ds1,
select = c(vgdaysweek)) #Subset 1i data
anycaremonth_vgdaysweek_m1i <-
apply(anycaremonth_yes_ds1_vgdaysweek, 2, mean) #Calculate m1i
anycaremonth_vgdaysweek_sd1i <-
apply(anycaremonth_yes_ds1_vgdaysweek, 2, sd) #Calculate sd1i
anycaremonth_vgdaysweek_n1i <- colSums(!
is.na(anycaremonth_yes_ds1_vgdaysweek)) #Calculate n1i
anycaremonth_no_ds1_vgdaysweek <- subset(anycaremonth_no_ds1, select
= c(vgdaysweek)) #Subset 2i data
anycaremonth_vgdaysweek_m2i <-
apply(anycaremonth_no_ds1_vgdaysweek, 2, mean) #Calculate m2i
anycaremonth_vgdaysweek_sd2i <-
apply(anycaremonth_no_ds1_vgdaysweek, 2, sd) #Calculate sd2i
anycaremonth_vgdaysweek_n2i <- colSums(!
```

```
is.na(anycaremonth_no_ds1_vgdaysweek)) #Calculate n2i
ds1anycaremonth_meta <- data.frame(m1i =
anycaremonth_vgdaysweek_m1i, sd1i = anycaremonth_vgdaysweek_sd1i, n1i
= anycaremonth_vgdaysweek_n1i, m2i = anycaremonth_vgdaysweek_m2i,
sd2i = anycaremonth_vgdaysweek_sd2i, n2i =
anycaremonth_vgdaysweek_n2i) #MA dataframe
ds1anycaremonth_meta <- escalc(measure="MD", m1i = m1i, sd1i = sd1i,
n1i = n1i, m2i = m2i, sd2i = sd2i, n2i = n2i, data=ds1anycaremonth_meta)
#Calculate yi and vi

#Study 2
anycaremonth_yes_ds2_vgdaysweek <- subset(anycaremonth_yes_ds2,
select = c(vgdaysweek)) #Subset 1i data
anycaremonth_vgdaysweek_m1i <-
apply(anycaremonth_yes_ds2_vgdaysweek, 2, mean) #Calculate m1i
anycaremonth_vgdaysweek_sd1i <-
apply(anycaremonth_yes_ds2_vgdaysweek, 2, sd) #Calculate sd1i
anycaremonth_vgdaysweek_n1i <- colSums(!
is.na(anycaremonth_yes_ds2_vgdaysweek)) #Calculate n1i
anycaremonth_no_ds2_vgdaysweek <- subset(anycaremonth_no_ds2, select
= c(vgdaysweek)) #Subset 2i data
anycaremonth_vgdaysweek_m2i <-
apply(anycaremonth_no_ds2_vgdaysweek, 2, mean) #Calculate m2i
anycaremonth_vgdaysweek_sd2i <-
apply(anycaremonth_no_ds2_vgdaysweek, 2, sd) #Calculate sd2i
anycaremonth_vgdaysweek_n2i <- colSums(!
is.na(anycaremonth_no_ds2_vgdaysweek)) #Calculate n2i
ds2anycaremonth_meta <- data.frame(m1i =
anycaremonth_vgdaysweek_m1i, sd1i = anycaremonth_vgdaysweek_sd1i, n1i
= anycaremonth_vgdaysweek_n1i, m2i = anycaremonth_vgdaysweek_m2i,
sd2i = anycaremonth_vgdaysweek_sd2i, n2i =
anycaremonth_vgdaysweek_n2i) #MA dataframe
ds2anycaremonth_meta <- escalc(measure="MD", m1i = m1i, sd1i = sd1i,
n1i = n1i, m2i = m2i, sd2i = sd2i, n2i = n2i, data=ds2anycaremonth_meta)
#Calculate yi and vi

#FE meta-analysis
(anycaremonth_meta <- rbind(ds1anycaremonth_meta,
ds2anycaremonth_meta))

##        m1i     sd1i n1i      m2i     sd2i n2i     yi     vi
## 1 5.250000 2.259899  36 5.145374 2.226353 227 0.1046 0.1637
## 2 5.022222 2.044328  90 4.705882 2.238653 357 0.3163 0.0605

(anycaremonth_vgdaysweek_meta <- rma(yi=yi, vi=vi, measure="MD",
method="FE", data=anycaremonth_meta))

##
## Fixed-Effects Model (k = 2)
##
## Test for Heterogeneity:
```

```
## Q(df = 1) = 0.1999, p-val = 0.6548
##
## Model Results:
##
## estimate      se    zval    pval   ci.lb   ci.ub
##   0.2592  0.2101  1.2336  0.2174  -0.1526  0.6711
##
## ---
## Signif. codes:  0 '***' 0.001 '**' 0.01 '*' 0.05 '.' 0.1 ' ' 1
```

Mental health care - discharge

Replicated Effect

Hours per day

Study 1

```
(vghrsday_mhdis_ds1 <- t.test(vghrsday ~ mhdis, data=ds1anymhscreen))

##
##  Welch Two Sample t-test
##
## data:  vghrsday by mhdis
## t = 0.68063, df = 84.106, p-value = 0.498
## alternative hypothesis: true difference in means is not equal to 0
## 95 percent confidence interval:
##  -0.4493641  0.9170512
## sample estimates:
## mean in group 0 mean in group 1
##        2.775510        2.541667
```

Study 2

```
(vghrsday_mhdis_ds2 <- t.test(vghrsday ~ mhdis, data=ds2anymhscreen))

##
##  Welch Two Sample t-test
##
## data:  vghrsday by mhdis
## t = -2.4998, df = 205.15, p-value = 0.01321
## alternative hypothesis: true difference in means is not equal to 0
## 95 percent confidence interval:
##  -1.5912819 -0.1879708
## sample estimates:
## mean in group 0 mean in group 1
##        3.194444        4.084071
```

Hours per week

Study 1

```
(vghrsweek_mhdis_ds1 <- t.test(vghrsweek ~ mhdis, 
data=ds1anymhscreen))

## 
##  Welch Two Sample t-test
## 
## data:  vghrsweek by mhdis
## t = 0.70153, df = 82.026, p-value = 0.485
## alternative hypothesis: true difference in means is not equal to 0
## 95 percent confidence interval:
##  -3.378858  7.060150
## sample estimates:
## mean in group 0 mean in group 1 
##        16.44898        14.60833
```

Study 2

```
(vghrsweek_mhdis_ds2 <- t.test(vghrsweek ~ mhdis, 
data=ds2anymhscreen))

## 
##  Welch Two Sample t-test
## 
## data:  vghrsweek by mhdis
## t = -2.8177, df = 216.39, p-value = 0.005286
## alternative hypothesis: true difference in means is not equal to 0
## 95 percent confidence interval:
##  -11.519971  -2.036813
## sample estimates:
## mean in group 0 mean in group 1 
##        15.70833        22.48673
```

Days per week

Study 1

```
(vgdaysweek_mhdis_ds1 <- t.test(vgdaysweek ~ mhdis, 
data=ds1anymhscreen))

## 
##  Welch Two Sample t-test
## 
## data:  vgdaysweek by mhdis
## t = -0.3834, df = 88.029, p-value = 0.7024
```

```
## alternative hypothesis: true difference in means is not equal to 0
## 95 percent confidence interval:
##  -0.9022650  0.6104283
## sample estimates:
## mean in group 0 mean in group 1
##        5.204082        5.350000
```

Study 2

```
(vgdaysweek_mhdis_ds2 <- t.test(vgdaysweek ~ mhdis,
data=ds2anymhscreen))
```

```
##
##  Welch Two Sample t-test
##
## data:  vgdaysweek by mhdis
## t = -1.5956, df = 110.58, p-value = 0.1134
## alternative hypothesis: true difference in means is not equal to 0
## 95 percent confidence interval:
##  -1.1096841  0.1197628
## sample estimates:
## mean in group 0 mean in group 1
##        4.513889        5.008850
```

MD

Hours per day

```
#Study 1
mhdis_yes_ds1_vghrsday <- subset(mhdis_yes_ds1, select = c(vghrsday))
#Subset 1i data
mhdis_vghrsday_m1i <- apply(mhdis_yes_ds1_vghrsday, 2, mean)
#Calculate m1i
mhdis_vghrsday_sd1i <- apply(mhdis_yes_ds1_vghrsday, 2, sd) #Calculate
sd1i
mhdis_vghrsday_n1i <- colSums(!is.na(mhdis_yes_ds1_vghrsday)) #Calculate
n1i
mhdis_no_ds1_vghrsday <- subset(mhdis_no_ds1, select = c(vghrsday))
#Subset 2i data
mhdis_vghrsday_m2i <- apply(mhdis_no_ds1_vghrsday, 2, mean) #Calculate
m2i
mhdis_vghrsday_sd2i <- apply(mhdis_no_ds1_vghrsday, 2, sd) #Calculate
sd2i
mhdis_vghrsday_n2i <- colSums(!is.na(mhdis_no_ds1_vghrsday)) #Calculate
n2i
ds1mhdis_meta <- data.frame(m1i = mhdis_vghrsday_m1i, sd1i =
mhdis_vghrsday_sd1i, n1i = mhdis_vghrsday_n1i, m2i =
mhdis_vghrsday_m2i, sd2i = mhdis_vghrsday_sd2i, n2i =
```

```
mhdis_vghrsday_n2i) #MA dataframe
ds1mhdis_meta <- escalc(measure="MD", m1i = m1i, sd1i = sd1i, n1i = n1i,
m2i = m2i, sd2i = sd2i, n2i = n2i, data=ds1mhdis_meta) #Calculate yi and vi

#Study 2
mhdis_yes_ds2_vghrsday <- subset(mhdis_yes_ds2, select = c(vghrsday))
#Subset 1i data
mhdis_vghrsday_m1i <- apply(mhdis_yes_ds2_vghrsday, 2, mean)
#Calculate m1i
mhdis_vghrsday_sd1i <- apply(mhdis_yes_ds2_vghrsday, 2, sd) #Calculate
sd1i
mhdis_vghrsday_n1i <- colSums(!is.na(mhdis_yes_ds2_vghrsday)) #Calculate
n1i
mhdis_no_ds2_vghrsday <- subset(mhdis_no_ds2, select = c(vghrsday))
#Subset 2i data
mhdis_vghrsday_m2i <- apply(mhdis_no_ds2_vghrsday, 2, mean) #Calculate
m2i
mhdis_vghrsday_sd2i <- apply(mhdis_no_ds2_vghrsday, 2, sd) #Calculate
sd2i
mhdis_vghrsday_n2i <- colSums(!is.na(mhdis_no_ds2_vghrsday)) #Calculate
n2i
ds2mhdis_meta <- data.frame(m1i = mhdis_vghrsday_m1i, sd1i =
mhdis_vghrsday_sd1i, n1i = mhdis_vghrsday_n1i, m2i =
mhdis_vghrsday_m2i, sd2i = mhdis_vghrsday_sd2i, n2i =
mhdis_vghrsday_n2i) #MA dataframe
ds2mhdis_meta <- escalc(measure="MD", m1i = m1i, sd1i = sd1i, n1i = n1i,
m2i = m2i, sd2i = sd2i, n2i = n2i, data=ds2mhdis_meta) #Calculate yi and vi

#FE meta-analysis
(mhdis_meta <- rbind(ds1mhdis_meta, ds2mhdis_meta))

##        m1i     sd1i n1i      m2i     sd2i n2i      yi     vi
## 1 2.541667 1.931150 120 2.775510 2.064232  49 -0.2338 0.1180
## 2 4.084071 3.691072 226 3.194444 2.185992  72  0.8896 0.1267

(mhdis_vghrsday_meta <- rma(yi=yi, vi=vi, measure="MD", method="FE",
data=mhdis_meta))

##
## Fixed-Effects Model (k = 2)
##
## Test for Heterogeneity:
## Q(df = 1) = 5.1583, p-val = 0.0231
##
## Model Results:
##
## estimate      se     zval     pval    ci.lb    ci.ub
##   0.3081   0.2472   1.2465   0.2126  -0.1763   0.7926
##
## ---
## Signif. codes:  0 '***' 0.001 '**' 0.01 '*' 0.05 '.' 0.1 ' ' 1
```

Hours per week

```
#Study 1
mhdis_yes_ds1_vghrsweek <- subset(mhdis_yes_ds1, select = c(vghrsweek))
#Subset 1i data
mhdis_vghrsweek_m1i <- apply(mhdis_yes_ds1_vghrsweek, 2, mean)
#Calculate m1i
mhdis_vghrsweek_sd1i <- apply(mhdis_yes_ds1_vghrsweek, 2, sd)
#Calculate sd1i
mhdis_vghrsweek_n1i <- colSums(!is.na(mhdis_yes_ds1_vghrsweek))
#Calculate n1i
mhdis_no_ds1_vghrsweek <- subset(mhdis_no_ds1, select = c(vghrsweek))
#Subset 2i data
mhdis_vghrsweek_m2i <- apply(mhdis_no_ds1_vghrsweek, 2, mean)
#Calculate m2i
mhdis_vghrsweek_sd2i <- apply(mhdis_no_ds1_vghrsweek, 2, sd) #Calculate
sd2i
mhdis_vghrsweek_n2i <- colSums(!is.na(mhdis_no_ds1_vghrsweek))
#Calculate n2i
ds1mhdis_meta <- data.frame(m1i = mhdis_vghrsweek_m1i, sd1i =
mhdis_vghrsweek_sd1i, n1i = mhdis_vghrsweek_n1i, m2i =
mhdis_vghrsweek_m2i, sd2i = mhdis_vghrsweek_sd2i, n2i =
mhdis_vghrsweek_n2i) #MA dataframe
ds1mhdis_meta <- escalc(measure="MD", m1i = m1i, sd1i = sd1i, n1i = n1i,
m2i = m2i, sd2i = sd2i, n2i = n2i, data=ds1mhdis_meta) #Calculate yi and vi

#Study 2
mhdis_yes_ds2_vghrsweek <- subset(mhdis_yes_ds2, select = c(vghrsweek))
#Subset 1i data
mhdis_vghrsweek_m1i <- apply(mhdis_yes_ds2_vghrsweek, 2, mean)
#Calculate m1i
mhdis_vghrsweek_sd1i <- apply(mhdis_yes_ds2_vghrsweek, 2, sd)
#Calculate sd1i
mhdis_vghrsweek_n1i <- colSums(!is.na(mhdis_yes_ds2_vghrsweek))
#Calculate n1i
mhdis_no_ds2_vghrsweek <- subset(mhdis_no_ds2, select = c(vghrsweek))
#Subset 2i data
mhdis_vghrsweek_m2i <- apply(mhdis_no_ds2_vghrsweek, 2, mean)
#Calculate m2i
mhdis_vghrsweek_sd2i <- apply(mhdis_no_ds2_vghrsweek, 2, sd) #Calculate
sd2i
mhdis_vghrsweek_n2i <- colSums(!is.na(mhdis_no_ds2_vghrsweek))
#Calculate n2i
ds2mhdis_meta <- data.frame(m1i = mhdis_vghrsweek_m1i, sd1i =
mhdis_vghrsweek_sd1i, n1i = mhdis_vghrsweek_n1i, m2i =
mhdis_vghrsweek_m2i, sd2i = mhdis_vghrsweek_sd2i, n2i =
mhdis_vghrsweek_n2i) #MA dataframe
ds2mhdis_meta <- escalc(measure="MD", m1i = m1i, sd1i = sd1i, n1i = n1i,
m2i = m2i, sd2i = sd2i, n2i = n2i, data=ds2mhdis_meta) #Calculate yi and vi
```

```
#FE meta-analysis
(mhdis_meta <- rbind(ds1mhdis_meta, ds2mhdis_meta))

##        m1i     sd1i n1i      m2i     sd2i n2i      yi     vi
## 1 14.60833 14.42704 120 16.44898 15.88508  49 -1.8406 6.8842
## 2 22.48673 25.60178 226 15.70833 14.41775  72  6.7784 5.7873

(mhdis_vghrsweek_meta <- rma(yi=yi, vi=vi, measure="MD", method="FE",
data=mhdis_meta))

##
## Fixed-Effects Model (k = 2)
##
## Test for Heterogeneity:
## Q(df = 1) = 5.8626, p-val = 0.0155
##
## Model Results:
##
## estimate      se     zval     pval    ci.lb    ci.ub
##   2.8419  1.7732   1.6027   0.1090  -0.6334   6.3173
##
## ---
## Signif. codes:  0 '***' 0.001 '**' 0.01 '*' 0.05 '.' 0.1 ' ' 1
```

Days per week

```
#Study 1
mhdis_yes_ds1_vgdaysweek <- subset(mhdis_yes_ds1, select =
c(vgdaysweek)) #Subset 1i data
mhdis_vgdaysweek_m1i <- apply(mhdis_yes_ds1_vgdaysweek, 2, mean)
#Calculate m1i
mhdis_vgdaysweek_sd1i <- apply(mhdis_yes_ds1_vgdaysweek, 2, sd)
#Calculate sd1i
mhdis_vgdaysweek_n1i <- colSums(!is.na(mhdis_yes_ds1_vgdaysweek))
#Calculate n1i
mhdis_no_ds1_vgdaysweek <- subset(mhdis_no_ds1, select =
c(vgdaysweek)) #Subset 2i data
mhdis_vgdaysweek_m2i <- apply(mhdis_no_ds1_vgdaysweek, 2, mean)
#Calculate m2i
mhdis_vgdaysweek_sd2i <- apply(mhdis_no_ds1_vgdaysweek, 2, sd)
#Calculate sd2i
mhdis_vgdaysweek_n2i <- colSums(!is.na(mhdis_no_ds1_vgdaysweek))
#Calculate n2i
ds1mhdis_meta <- data.frame(m1i = mhdis_vgdaysweek_m1i, sd1i =
mhdis_vgdaysweek_sd1i, n1i = mhdis_vgdaysweek_n1i, m2i =
mhdis_vgdaysweek_m2i, sd2i = mhdis_vgdaysweek_sd2i, n2i =
mhdis_vgdaysweek_n2i) #MA dataframe
ds1mhdis_meta <- escalc(measure="MD", m1i = m1i, sd1i = sd1i, n1i = n1i,
m2i = m2i, sd2i = sd2i, n2i = n2i, data=ds1mhdis_meta) #Calculate yi and vi
```

```
#Study 2
mhdis_yes_ds2_vgdaysweek <- subset(mhdis_yes_ds2, select =
c(vgdaysweek)) #Subset 1i data
mhdis_vgdaysweek_m1i <- apply(mhdis_yes_ds2_vgdaysweek, 2, mean)
#Calculate m1i
mhdis_vgdaysweek_sd1i <- apply(mhdis_yes_ds2_vgdaysweek, 2, sd)
#Calculate sd1i
mhdis_vgdaysweek_n1i <- colSums(!is.na(mhdis_yes_ds2_vgdaysweek))
#Calculate n1i
mhdis_no_ds2_vgdaysweek <- subset(mhdis_no_ds2, select =
c(vgdaysweek)) #Subset 2i data
mhdis_vgdaysweek_m2i <- apply(mhdis_no_ds2_vgdaysweek, 2, mean)
#Calculate m2i
mhdis_vgdaysweek_sd2i <- apply(mhdis_no_ds2_vgdaysweek, 2, sd)
#Calculate sd2i
mhdis_vgdaysweek_n2i <- colSums(!is.na(mhdis_no_ds2_vgdaysweek))
#Calculate n2i
ds2mhdis_meta <- data.frame(m1i = mhdis_vgdaysweek_m1i, sd1i =
mhdis_vgdaysweek_sd1i, n1i = mhdis_vgdaysweek_n1i, m2i =
mhdis_vgdaysweek_m2i, sd2i = mhdis_vgdaysweek_sd2i, n2i =
mhdis_vgdaysweek_n2i) #MA dataframe
ds2mhdis_meta <- escalc(measure="MD", m1i = m1i, sd1i = sd1i, n1i = n1i,
m2i = m2i, sd2i = sd2i, n2i = n2i, data=ds2mhdis_meta) #Calculate yi and vi

#FE meta-analysis
(mhdis_meta <- rbind(ds1mhdis_meta, ds2mhdis_meta))

##       m1i     sd1i n1i      m2i     sd2i n2i     yi     vi
## 1 5.35000 2.221552 120 5.204082 2.254436  49 0.1459 0.1449
## 2 5.00885 2.122873 226 4.513889 2.343664  72 0.4950 0.0962

(mhdis_vgdaysweek_meta <- rma(yi=yi, vi=vi, measure="MD",
method="FE", data=mhdis_meta))

##
## Fixed-Effects Model (k = 2)
##
## Test for Heterogeneity:
## Q(df = 1) = 0.5054, p-val = 0.4772
##
## Model Results:
##
## estimate      se     zval     pval    ci.lb    ci.ub
##   0.3556   0.2405   1.4790   0.1391  -0.1156   0.8269
##
## ---
## Signif. codes:  0 '***' 0.001 '**' 0.01 '*' 0.05 '.' 0.1 ' ' 1
```

Mental health care - year

Replicated Effect

Hours per day

Study 1

```
(vghrsday_mhyear_ds1 <- t.test(vghrsday ~ mhyear, 
data=ds1anymhscreen))

## 
##  Welch Two Sample t-test
## 
## data:  vghrsday by mhyear
## t = 1.37, df = 166.67, p-value = 0.1725
## alternative hypothesis: true difference in means is not equal to 0
## 95 percent confidence interval:
##  -0.1816303  1.0051432
## sample estimates:
## mean in group 0 mean in group 1 
##        2.806818        2.395062
```

Study 2

```
(vghrsday_mhyear_ds2 <- t.test(vghrsday ~ mhyear, 
data=ds2anymhscreen))

## 
##  Welch Two Sample t-test
## 
## data:  vghrsday by mhyear
## t = -1.123, df = 292.23, p-value = 0.2624
## alternative hypothesis: true difference in means is not equal to 0
## 95 percent confidence interval:
##  -1.1855396  0.3241387
## sample estimates:
## mean in group 0 mean in group 1 
##        3.614754        4.045455
```

Hours per week

Study 1

```
(vghrsweek_mhyear_ds1 <- t.test(vghrsweek ~ mhyear, 
data=ds1anymhscreen))

## 
##  Welch Two Sample t-test
```

```
##
## data:  vghrsweek by mhyear
## t = 1.378, df = 165.58, p-value = 0.1701
## alternative hypothesis: true difference in means is not equal to 0
## 95 percent confidence interval:
##  -1.34943  7.58512
## sample estimates:
## mean in group 0 mean in group 1
##        16.63636        13.51852
```

Study 2

```
(vghrsweek_mhyear_ds2 <- t.test(vghrsweek ~ mhyear,
data=ds2anymhscreen))

##
##  Welch Two Sample t-test
##
## data:  vghrsweek by mhyear
## t = -0.88549, df = 288.72, p-value = 0.3766
## alternative hypothesis: true difference in means is not equal to 0
## 95 percent confidence interval:
##  -7.629388  2.894664
## sample estimates:
## mean in group 0 mean in group 1
##        19.45082        21.81818
```

Days per week

Study 1

```
(vgdaysweek_mhyear_ds1 <- t.test(vgdaysweek ~ mhyear,
data=ds1anymhscreen))

##
##  Welch Two Sample t-test
##
## data:  vgdaysweek by mhyear
## t = -0.14358, df = 166.81, p-value = 0.886
## alternative hypothesis: true difference in means is not equal to 0
## 95 percent confidence interval:
##  -0.7263392  0.6278543
## sample estimates:
## mean in group 0 mean in group 1
##        5.284091        5.333333
```

Study 2

```
(vgdaysweek_mhyear_ds2 <- t.test(vgdaysweek ~ mhyear,
data=ds2anymhscreen))

##
##  Welch Two Sample t-test
##
## data:  vgdaysweek by mhyear
## t = -0.45046, df = 246.26, p-value = 0.6528
## alternative hypothesis: true difference in means is not equal to 0
## 95 percent confidence interval:
##  -0.6330343  0.3973785
## sample estimates:
## mean in group 0 mean in group 1
##        4.819672        4.937500
```

MD

Hours per day

```
#Study 1
mhyear_yes_ds1_vghrsday <- subset(mhyear_yes_ds1, select = c(vghrsday))
#Subset 1i data
mhyear_vghrsday_m1i <- apply(mhyear_yes_ds1_vghrsday, 2, mean)
#Calculate m1i
mhyear_vghrsday_sd1i <- apply(mhyear_yes_ds1_vghrsday, 2, sd)
#Calculate sd1i
mhyear_vghrsday_n1i <- colSums(!is.na(mhyear_yes_ds1_vghrsday))
#Calculate n1i
mhyear_no_ds1_vghrsday <- subset(mhyear_no_ds1, select = c(vghrsday))
#Subset 2i data
mhyear_vghrsday_m2i <- apply(mhyear_no_ds1_vghrsday, 2, mean)
#Calculate m2i
mhyear_vghrsday_sd2i <- apply(mhyear_no_ds1_vghrsday, 2, sd) #Calculate
sd2i
mhyear_vghrsday_n2i <- colSums(!is.na(mhyear_no_ds1_vghrsday))
#Calculate n2i
ds1mhyear_meta <- data.frame(m1i = mhyear_vghrsday_m1i, sd1i =
mhyear_vghrsday_sd1i, n1i = mhyear_vghrsday_n1i, m2i =
mhyear_vghrsday_m2i, sd2i = mhyear_vghrsday_sd2i, n2i =
mhyear_vghrsday_n2i) #MA dataframe
ds1mhyear_meta <- escalc(measure="MD", m1i = m1i, sd1i = sd1i, n1i =
n1i, m2i = m2i, sd2i = sd2i, n2i = n2i, data=ds1mhyear_meta) #Calculate yi
and vi

#Study 2
mhyear_yes_ds2_vghrsday <- subset(mhyear_yes_ds2, select = c(vghrsday))
#Subset 1i data
mhyear_vghrsday_m1i <- apply(mhyear_yes_ds2_vghrsday, 2, mean)
```

```
#Calculate m1i
mhyear_vghrsday_sd1i <- apply(mhyear_yes_ds2_vghrsday, 2, sd)
#Calculate sd1i
mhyear_vghrsday_n1i <- colSums(!is.na(mhyear_yes_ds2_vghrsday))
#Calculate n1i
mhyear_no_ds2_vghrsday <- subset(mhyear_no_ds2, select = c(vghrsday))
#Subset 2i data
mhyear_vghrsday_m2i <- apply(mhyear_no_ds2_vghrsday, 2, mean)
#Calculate m2i
mhyear_vghrsday_sd2i <- apply(mhyear_no_ds2_vghrsday, 2, sd) #Calculate
sd2i
mhyear_vghrsday_n2i <- colSums(!is.na(mhyear_no_ds2_vghrsday))
#Calculate n2i
ds2mhyear_meta <- data.frame(m1i = mhyear_vghrsday_m1i, sd1i =
mhyear_vghrsday_sd1i, n1i = mhyear_vghrsday_n1i, m2i =
mhyear_vghrsday_m2i, sd2i = mhyear_vghrsday_sd2i, n2i =
mhyear_vghrsday_n2i) #MA dataframe
ds2mhyear_meta <- escalc(measure="MD", m1i = m1i, sd1i = sd1i, n1i =
n1i, m2i = m2i, sd2i = sd2i, n2i = n2i, data=ds2mhyear_meta) #Calculate yi
and vi

#FE meta-analysis
(mhyear_meta <- rbind(ds1mhyear_meta, ds2mhyear_meta))

##        m1i      sd1i n1i      m2i      sd2i n2i      yi     vi
## 1 2.395062 1.828107  81 2.806818 2.078118  88 -0.4118 0.0903
## 2 4.045455 3.723007 176 3.614754 2.887553 122  0.4307 0.1471

(mhyear_vghrsday_meta <- rma(yi=yi, vi=vi, measure="MD", method="FE",
data=mhyear_meta))

##
## Fixed-Effects Model (k = 2)
##
## Test for Heterogeneity:
## Q(df = 1) = 2.9892, p-val = 0.0838
##
## Model Results:
##
## estimate      se     zval     pval    ci.lb    ci.ub
##  -0.0912   0.2366  -0.3857   0.6998  -0.5549   0.3724
##
## ---
## Signif. codes:  0 '***' 0.001 '**' 0.01 '*' 0.05 '.' 0.1 ' ' 1
```

Hours per week

```
#Study 1
mhyear_yes_ds1_vghrsweek <- subset(mhyear_yes_ds1, select =
c(vghrsweek)) #Subset 1i data
```

```
mhyear_vghrsweek_m1i <- apply(mhyear_yes_ds1_vghrsweek, 2, mean)
#Calculate m1i
mhyear_vghrsweek_sd1i <- apply(mhyear_yes_ds1_vghrsweek, 2, sd)
#Calculate sd1i
mhyear_vghrsweek_n1i <- colSums(!is.na(mhyear_yes_ds1_vghrsweek))
#Calculate n1i
mhyear_no_ds1_vghrsweek <- subset(mhyear_no_ds1, select =
c(vghrsweek)) #Subset 2i data
mhyear_vghrsweek_m2i <- apply(mhyear_no_ds1_vghrsweek, 2, mean)
#Calculate m2i
mhyear_vghrsweek_sd2i <- apply(mhyear_no_ds1_vghrsweek, 2, sd)
#Calculate sd2i
mhyear_vghrsweek_n2i <- colSums(!is.na(mhyear_no_ds1_vghrsweek))
#Calculate n2i
ds1mhyear_meta <- data.frame(m1i = mhyear_vghrsweek_m1i, sd1i =
mhyear_vghrsweek_sd1i, n1i = mhyear_vghrsweek_n1i, m2i =
mhyear_vghrsweek_m2i, sd2i = mhyear_vghrsweek_sd2i, n2i =
mhyear_vghrsweek_n2i) #MA dataframe
ds1mhyear_meta <- escalc(measure="MD", m1i = m1i, sd1i = sd1i, n1i =
n1i, m2i = m2i, sd2i = sd2i, n2i = n2i, data=ds1mhyear_meta) #Calculate yi
and vi

#Study 2
mhyear_yes_ds2_vghrsweek <- subset(mhyear_yes_ds2, select =
c(vghrsweek)) #Subset 1i data
mhyear_vghrsweek_m1i <- apply(mhyear_yes_ds2_vghrsweek, 2, mean)
#Calculate m1i
mhyear_vghrsweek_sd1i <- apply(mhyear_yes_ds2_vghrsweek, 2, sd)
#Calculate sd1i
mhyear_vghrsweek_n1i <- colSums(!is.na(mhyear_yes_ds2_vghrsweek))
#Calculate n1i
mhyear_no_ds2_vghrsweek <- subset(mhyear_no_ds2, select =
c(vghrsweek)) #Subset 2i data
mhyear_vghrsweek_m2i <- apply(mhyear_no_ds2_vghrsweek, 2, mean)
#Calculate m2i
mhyear_vghrsweek_sd2i <- apply(mhyear_no_ds2_vghrsweek, 2, sd)
#Calculate sd2i
mhyear_vghrsweek_n2i <- colSums(!is.na(mhyear_no_ds2_vghrsweek))
#Calculate n2i
ds2mhyear_meta <- data.frame(m1i = mhyear_vghrsweek_m1i, sd1i =
mhyear_vghrsweek_sd1i, n1i = mhyear_vghrsweek_n1i, m2i =
mhyear_vghrsweek_m2i, sd2i = mhyear_vghrsweek_sd2i, n2i =
mhyear_vghrsweek_n2i) #MA dataframe
ds2mhyear_meta <- escalc(measure="MD", m1i = m1i, sd1i = sd1i, n1i =
n1i, m2i = m2i, sd2i = sd2i, n2i = n2i, data=ds2mhyear_meta) #Calculate yi
and vi

#FE meta-analysis
(mhyear_meta <- rbind(ds1mhyear_meta, ds2mhyear_meta))
```

```
##        m1i     sd1i n1i      m2i     sd2i n2i      yi     vi
## 1 13.51852 13.39600  81 16.63636 15.98576  88 -3.1178 5.1194
## 2 21.81818 25.40801 176 19.45082 20.60374 122  2.3674 7.1476
```

```
(mhyear_vghrsweek_meta <- rma(yi=yi, vi=vi, measure="MD",
method="FE", data=mhyear_meta))
```

```
##
## Fixed-Effects Model (k = 2)
##
## Test for Heterogeneity:
## Q(df = 1) = 2.4527, p-val = 0.1173
##
## Model Results:
##
## estimate       se     zval     pval    ci.lb    ci.ub
##  -0.8287   1.7271  -0.4798   0.6314  -4.2138   2.5564
##
## ---
## Signif. codes:  0 '***' 0.001 '**' 0.01 '*' 0.05 '.' 0.1 ' ' 1
```

Days per week

```
#Study 1
mhyear_yes_ds1_vgdaysweek <- subset(mhyear_yes_ds1, select =
c(vgdaysweek)) #Subset 1i data
mhyear_vgdaysweek_m1i <- apply(mhyear_yes_ds1_vgdaysweek, 2, mean)
#Calculate m1i
mhyear_vgdaysweek_sd1i <- apply(mhyear_yes_ds1_vgdaysweek, 2, sd)
#Calculate sd1i
mhyear_vgdaysweek_n1i <- colSums(!is.na(mhyear_yes_ds1_vgdaysweek))
#Calculate n1i
mhyear_no_ds1_vgdaysweek <- subset(mhyear_no_ds1, select =
c(vgdaysweek)) #Subset 2i data
mhyear_vgdaysweek_m2i <- apply(mhyear_no_ds1_vgdaysweek, 2, mean)
#Calculate m2i
mhyear_vgdaysweek_sd2i <- apply(mhyear_no_ds1_vgdaysweek, 2, sd)
#Calculate sd2i
mhyear_vgdaysweek_n2i <- colSums(!is.na(mhyear_no_ds1_vgdaysweek))
#Calculate n2i
ds1mhyear_meta <- data.frame(m1i = mhyear_vgdaysweek_m1i, sd1i =
mhyear_vgdaysweek_sd1i, n1i = mhyear_vgdaysweek_n1i, m2i =
mhyear_vgdaysweek_m2i, sd2i = mhyear_vgdaysweek_sd2i, n2i =
mhyear_vgdaysweek_n2i) #MA dataframe
ds1mhyear_meta <- escalc(measure="MD", m1i = m1i, sd1i = sd1i, n1i =
n1i, m2i = m2i, sd2i = sd2i, n2i = n2i, data=ds1mhyear_meta) #Calculate yi
and vi

#Study 2
```

```
mhyear_yes_ds2_vgdaysweek <- subset(mhyear_yes_ds2, select =
c(vgdaysweek)) #Subset 1i data
mhyear_vgdaysweek_m1i <- apply(mhyear_yes_ds2_vgdaysweek, 2, mean)
#Calculate m1i
mhyear_vgdaysweek_sd1i <- apply(mhyear_yes_ds2_vgdaysweek, 2, sd)
#Calculate sd1i
mhyear_vgdaysweek_n1i <- colSums(!is.na(mhyear_yes_ds2_vgdaysweek))
#Calculate n1i
mhyear_no_ds2_vgdaysweek <- subset(mhyear_no_ds2, select =
c(vgdaysweek)) #Subset 2i data
mhyear_vgdaysweek_m2i <- apply(mhyear_no_ds2_vgdaysweek, 2, mean)
#Calculate m2i
mhyear_vgdaysweek_sd2i <- apply(mhyear_no_ds2_vgdaysweek, 2, sd)
#Calculate sd2i
mhyear_vgdaysweek_n2i <- colSums(!is.na(mhyear_no_ds2_vgdaysweek))
#Calculate n2i
ds2mhyear_meta <- data.frame(m1i = mhyear_vgdaysweek_m1i, sd1i =
mhyear_vgdaysweek_sd1i, n1i = mhyear_vgdaysweek_n1i, m2i =
mhyear_vgdaysweek_m2i, sd2i = mhyear_vgdaysweek_sd2i, n2i =
mhyear_vgdaysweek_n2i) #MA dataframe
ds2mhyear_meta <- escalc(measure="MD", m1i = m1i, sd1i = sd1i, n1i =
n1i, m2i = m2i, sd2i = sd2i, n2i = n2i, data=ds2mhyear_meta) #Calculate yi
and vi

#FE meta-analysis
(mhyear_meta <- rbind(ds1mhyear_meta, ds2mhyear_meta))

##        m1i     sd1i n1i      m2i     sd2i n2i     yi     vi
## 1 5.333333 2.173707  81 5.284091 2.284122  88 0.0492 0.1176
## 2 4.937500 2.111618 176 4.819672 2.292699 122 0.1178 0.0684

(mhyear_vgdaysweek_meta <- rma(yi=yi, vi=vi, measure="MD",
method="FE", data=mhyear_meta))

##
## Fixed-Effects Model (k = 2)
##
## Test for Heterogeneity:
## Q(df = 1) = 0.0253, p-val = 0.8737
##
## Model Results:
##
## estimate      se     zval     pval    ci.lb    ci.ub
##   0.0926   0.2080   0.4452   0.6561  -0.3150   0.5002
##
## ---
## Signif. codes:  0 '***' 0.001 '**' 0.01 '*' 0.05 '.' 0.1 ' ' 1
```

Mental health care - month

Replicated Effect

Hours per day

Study 1

```
(vghrsday_mhmonth_ds1 <- t.test(vghrsday ~ mhmonth,
data=ds1anymhscreen))

##
##  Welch Two Sample t-test
##
## data:  vghrsday by mhmonth
## t = -1.4666, df = 38.807, p-value = 0.1505
## alternative hypothesis: true difference in means is not equal to 0
## 95 percent confidence interval:
##  -1.5155007  0.2416398
## sample estimates:
## mean in group 0 mean in group 1
##        2.496403        3.133333
```

Study 2

```
(vghrsday_mhmonth_ds2 <- t.test(vghrsday ~ mhmonth,
data=ds2anymhscreen))

##
##  Welch Two Sample t-test
##
## data:  vghrsday by mhmonth
## t = 0.73059, df = 164.41, p-value = 0.4661
## alternative hypothesis: true difference in means is not equal to 0
## 95 percent confidence interval:
##  -0.5045699  1.0972713
## sample estimates:
## mean in group 0 mean in group 1
##        3.945701        3.649351
```

Hours per week

Study 1

```
(vghrsweek_mhmonth_ds1 <- t.test(vghrsweek ~ mhmonth,
data=ds1anymhscreen))

##
##  Welch Two Sample t-test
```

```
##
## data:  vghrsweek by mhmonth
## t = -1.2268, df = 39.024, p-value = 0.2272
## alternative hypothesis: true difference in means is not equal to 0
## 95 percent confidence interval:
##  -10.59930   2.59594
## sample estimates:
## mean in group 0 mean in group 1
##        14.43165        18.43333
```

Study 2

```
(vghrsweek_mhmonth_ds2 <- t.test(vghrsweek ~ mhmonth,
data=ds2anymhscreen))
```

```
##
##  Welch Two Sample t-test
##
## data:  vghrsweek by mhmonth
## t = 1.1423, df = 165.09, p-value = 0.255
## alternative hypothesis: true difference in means is not equal to 0
## 95 percent confidence interval:
##  -2.326368  8.713746
## sample estimates:
## mean in group 0 mean in group 1
##        21.67421        18.48052
```

Days per week

Study 1

```
(vgdaysweek_mhmonth_ds1 <- t.test(vgdaysweek ~ mhmonth,
data=ds1anymhscreen))
```

```
##
##  Welch Two Sample t-test
##
## data:  vgdaysweek by mhmonth
## t = -0.71491, df = 43.295, p-value = 0.4785
## alternative hypothesis: true difference in means is not equal to 0
## 95 percent confidence interval:
##  -1.2029034  0.5731672
## sample estimates:
## mean in group 0 mean in group 1
##        5.251799        5.566667
```

Study 2

```
(vgdaysweek_mhmonth_ds2 <- t.test(vgdaysweek ~ mhmonth,
data=ds2anymhscreen))

##
##  Welch Two Sample t-test
##
## data:  vgdaysweek by mhmonth
## t = 0.21881, df = 143.21, p-value = 0.8271
## alternative hypothesis: true difference in means is not equal to 0
## 95 percent confidence interval:
##  -0.4886324  0.6102754
## sample estimates:
## mean in group 0 mean in group 1
##        4.904977        4.844156
```

MD

Hours per day

```
#Study 1
mhmonth_yes_ds1_vghrsday <- subset(mhmonth_yes_ds1, select =
c(vghrsday)) #Subset 1i data
mhmonth_vghrsday_m1i <- apply(mhmonth_yes_ds1_vghrsday, 2, mean)
#Calculate m1i
mhmonth_vghrsday_sd1i <- apply(mhmonth_yes_ds1_vghrsday, 2, sd)
#Calculate sd1i
mhmonth_vghrsday_n1i <- colSums(!is.na(mhmonth_yes_ds1_vghrsday))
#Calculate n1i
mhmonth_no_ds1_vghrsday <- subset(mhmonth_no_ds1, select =
c(vghrsday)) #Subset 2i data
mhmonth_vghrsday_m2i <- apply(mhmonth_no_ds1_vghrsday, 2, mean)
#Calculate m2i
mhmonth_vghrsday_sd2i <- apply(mhmonth_no_ds1_vghrsday, 2, sd)
#Calculate sd2i
mhmonth_vghrsday_n2i <- colSums(!is.na(mhmonth_no_ds1_vghrsday))
#Calculate n2i
ds1mhmonth_meta <- data.frame(m1i = mhmonth_vghrsday_m1i, sd1i =
mhmonth_vghrsday_sd1i, n1i = mhmonth_vghrsday_n1i, m2i =
mhmonth_vghrsday_m2i, sd2i = mhmonth_vghrsday_sd2i, n2i =
mhmonth_vghrsday_n2i) #MA dataframe
ds1mhmonth_meta <- escalc(measure="MD", m1i = m1i, sd1i = sd1i, n1i =
n1i, m2i = m2i, sd2i = sd2i, n2i = n2i, data=ds1mhmonth_meta) #Calculate
yi and vi

#Study 2
mhmonth_yes_ds2_vghrsday <- subset(mhmonth_yes_ds2, select =
c(vghrsday)) #Subset 1i data
mhmonth_vghrsday_m1i <- apply(mhmonth_yes_ds2_vghrsday, 2, mean)
```

```
#Calculate m1i
mhmonth_vghrsday_sd1i <- apply(mhmonth_yes_ds2_vghrsday, 2, sd)
#Calculate sd1i
mhmonth_vghrsday_n1i <- colSums(!is.na(mhmonth_yes_ds2_vghrsday))
#Calculate n1i
mhmonth_no_ds2_vghrsday <- subset(mhmonth_no_ds2, select =
c(vghrsday)) #Subset 2i data
mhmonth_vghrsday_m2i <- apply(mhmonth_no_ds2_vghrsday, 2, mean)
#Calculate m2i
mhmonth_vghrsday_sd2i <- apply(mhmonth_no_ds2_vghrsday, 2, sd)
#Calculate sd2i
mhmonth_vghrsday_n2i <- colSums(!is.na(mhmonth_no_ds2_vghrsday))
#Calculate n2i
ds2mhmonth_meta <- data.frame(m1i = mhmonth_vghrsday_m1i, sd1i =
mhmonth_vghrsday_sd1i, n1i = mhmonth_vghrsday_n1i, m2i =
mhmonth_vghrsday_m2i, sd2i = mhmonth_vghrsday_sd2i, n2i =
mhmonth_vghrsday_n2i) #MA dataframe
ds2mhmonth_meta <- escalc(measure="MD", m1i = m1i, sd1i = sd1i, n1i =
n1i, m2i = m2i, sd2i = sd2i, n2i = n2i, data=ds2mhmonth_meta) #Calculate
yi and vi

#FE meta-analysis
(mhmonth_meta <- rbind(ds1mhmonth_meta, ds2mhmonth_meta))

##        m1i      sd1i n1i      m2i      sd2i n2i      yi     vi
## 1 3.133333 2.208656  30 2.496403 1.901083 139  0.6369 0.1886
## 2 3.649351 2.864328  77 3.945701 3.579835 221 -0.2964 0.1645

(mhmonth_vghrsday_meta <- rma(yi=yi, vi=vi, measure="MD",
method="FE", data=mhmonth_meta))

##
## Fixed-Effects Model (k = 2)
##
## Test for Heterogeneity:
## Q(df = 1) = 2.4665, p-val = 0.1163
##
## Model Results:
##
## estimate      se     zval     pval    ci.lb    ci.ub
##   0.1385  0.2964   0.4672   0.6404  -0.4425   0.7195
##
## ---
## Signif. codes:  0 '***' 0.001 '**' 0.01 '*' 0.05 '.' 0.1 ' ' 1
```

Hours per week

```
#Study 1
mhmonth_yes_ds1_vghrsweek <- subset(mhmonth_yes_ds1, select =
c(vghrsweek)) #Subset 1i data
```

```
mhmonth_vghrsweek_m1i <- apply(mhmonth_yes_ds1_vghrsweek, 2, mean)
#Calculate m1i
mhmonth_vghrsweek_sd1i <- apply(mhmonth_yes_ds1_vghrsweek, 2, sd)
#Calculate sd1i
mhmonth_vghrsweek_n1i <- colSums(!is.na(mhmonth_yes_ds1_vghrsweek))
#Calculate n1i
mhmonth_no_ds1_vghrsweek <- subset(mhmonth_no_ds1, select =
c(vghrsweek)) #Subset 2i data
mhmonth_vghrsweek_m2i <- apply(mhmonth_no_ds1_vghrsweek, 2, mean)
#Calculate m2i
mhmonth_vghrsweek_sd2i <- apply(mhmonth_no_ds1_vghrsweek, 2, sd)
#Calculate sd2i
mhmonth_vghrsweek_n2i <- colSums(!is.na(mhmonth_no_ds1_vghrsweek))
#Calculate n2i
ds1mhmonth_meta <- data.frame(m1i = mhmonth_vghrsweek_m1i, sd1i =
mhmonth_vghrsweek_sd1i, n1i = mhmonth_vghrsweek_n1i, m2i =
mhmonth_vghrsweek_m2i, sd2i = mhmonth_vghrsweek_sd2i, n2i =
mhmonth_vghrsweek_n2i) #MA dataframe
ds1mhmonth_meta <- escalc(measure="MD", m1i = m1i, sd1i = sd1i, n1i =
n1i, m2i = m2i, sd2i = sd2i, n2i = n2i, data=ds1mhmonth_meta) #Calculate
yi and vi

#Study 2
mhmonth_yes_ds2_vghrsweek <- subset(mhmonth_yes_ds2, select =
c(vghrsweek)) #Subset 1i data
mhmonth_vghrsweek_m1i <- apply(mhmonth_yes_ds2_vghrsweek, 2, mean)
#Calculate m1i
mhmonth_vghrsweek_sd1i <- apply(mhmonth_yes_ds2_vghrsweek, 2, sd)
#Calculate sd1i
mhmonth_vghrsweek_n1i <- colSums(!is.na(mhmonth_yes_ds2_vghrsweek))
#Calculate n1i
mhmonth_no_ds2_vghrsweek <- subset(mhmonth_no_ds2, select =
c(vghrsweek)) #Subset 2i data
mhmonth_vghrsweek_m2i <- apply(mhmonth_no_ds2_vghrsweek, 2, mean)
#Calculate m2i
mhmonth_vghrsweek_sd2i <- apply(mhmonth_no_ds2_vghrsweek, 2, sd)
#Calculate sd2i
mhmonth_vghrsweek_n2i <- colSums(!is.na(mhmonth_no_ds2_vghrsweek))
#Calculate n2i
ds2mhmonth_meta <- data.frame(m1i = mhmonth_vghrsweek_m1i, sd1i =
mhmonth_vghrsweek_sd1i, n1i = mhmonth_vghrsweek_n1i, m2i =
mhmonth_vghrsweek_m2i, sd2i = mhmonth_vghrsweek_sd2i, n2i =
mhmonth_vghrsweek_n2i) #MA dataframe
ds2mhmonth_meta <- escalc(measure="MD", m1i = m1i, sd1i = sd1i, n1i =
n1i, m2i = m2i, sd2i = sd2i, n2i = n2i, data=ds2mhmonth_meta) #Calculate
yi and vi

#FE meta-analysis
(mhmonth_meta <- rbind(ds1mhmonth_meta, ds2mhmonth_meta))
```

```
##        m1i     sd1i n1i      m2i     sd2i n2i      yi      vi
## 1 18.43333 16.56478  30 14.43165 14.40765 139  4.0017 10.6398
## 2 18.48052 19.71415  77 21.67421 24.73721 221 -3.1937  7.8163
```

```
(mhmonth_vghrsweek_meta <- rma(yi=yi, vi=vi, measure="MD",
method="FE", data=mhmonth_meta))
```

```
##
## Fixed-Effects Model (k = 2)
##
## Test for Heterogeneity:
## Q(df = 1) = 2.8052, p-val = 0.0940
##
## Model Results:
##
## estimate      se     zval    pval    ci.lb    ci.ub
##  -0.1464  2.1227  -0.0690  0.9450  -4.3069   4.0141
##
## ---
## Signif. codes:  0 '***' 0.001 '**' 0.01 '*' 0.05 '.' 0.1 ' ' 1
```

Days per week

```
#Study 1
mhmonth_yes_ds1_vgdaysweek <- subset(mhmonth_yes_ds1, select =
c(vgdaysweek)) #Subset 1i data
mhmonth_vgdaysweek_m1i <- apply(mhmonth_yes_ds1_vgdaysweek, 2,
mean) #Calculate m1i
mhmonth_vgdaysweek_sd1i <- apply(mhmonth_yes_ds1_vgdaysweek, 2, sd)
#Calculate sd1i
mhmonth_vgdaysweek_n1i <- colSums(!
is.na(mhmonth_yes_ds1_vgdaysweek)) #Calculate n1i
mhmonth_no_ds1_vgdaysweek <- subset(mhmonth_no_ds1, select =
c(vgdaysweek)) #Subset 2i data
mhmonth_vgdaysweek_m2i <- apply(mhmonth_no_ds1_vgdaysweek, 2,
mean) #Calculate m2i
mhmonth_vgdaysweek_sd2i <- apply(mhmonth_no_ds1_vgdaysweek, 2, sd)
#Calculate sd2i
mhmonth_vgdaysweek_n2i <- colSums(!
is.na(mhmonth_no_ds1_vgdaysweek)) #Calculate n2i
ds1mhmonth_meta <- data.frame(m1i = mhmonth_vgdaysweek_m1i, sd1i =
mhmonth_vgdaysweek_sd1i, n1i = mhmonth_vgdaysweek_n1i, m2i =
mhmonth_vgdaysweek_m2i, sd2i = mhmonth_vgdaysweek_sd2i, n2i =
mhmonth_vgdaysweek_n2i) #MA dataframe
ds1mhmonth_meta <- escalc(measure="MD", m1i = m1i, sd1i = sd1i, n1i =
n1i, m2i = m2i, sd2i = sd2i, n2i = n2i, data=ds1mhmonth_meta) #Calculate
yi and vi

#Study 2
```

```
mhmonth_yes_ds2_vgdaysweek <- subset(mhmonth_yes_ds2, select =
c(vgdaysweek)) #Subset 1i data
mhmonth_vgdaysweek_m1i <- apply(mhmonth_yes_ds2_vgdaysweek, 2,
mean) #Calculate m1i
mhmonth_vgdaysweek_sd1i <- apply(mhmonth_yes_ds2_vgdaysweek, 2, sd)
#Calculate sd1i
mhmonth_vgdaysweek_n1i <- colSums(!
is.na(mhmonth_yes_ds2_vgdaysweek)) #Calculate n1i
mhmonth_no_ds2_vgdaysweek <- subset(mhmonth_no_ds2, select =
c(vgdaysweek)) #Subset 2i data
mhmonth_vgdaysweek_m2i <- apply(mhmonth_no_ds2_vgdaysweek, 2,
mean) #Calculate m2i
mhmonth_vgdaysweek_sd2i <- apply(mhmonth_no_ds2_vgdaysweek, 2, sd)
#Calculate sd2i
mhmonth_vgdaysweek_n2i <- colSums(!
is.na(mhmonth_no_ds2_vgdaysweek)) #Calculate n2i
ds2mhmonth_meta <- data.frame(m1i = mhmonth_vgdaysweek_m1i, sd1i =
mhmonth_vgdaysweek_sd1i, n1i = mhmonth_vgdaysweek_n1i, m2i =
mhmonth_vgdaysweek_m2i, sd2i = mhmonth_vgdaysweek_sd2i, n2i =
mhmonth_vgdaysweek_n2i) #MA dataframe
ds2mhmonth_meta <- escalc(measure="MD", m1i = m1i, sd1i = sd1i, n1i =
n1i, m2i = m2i, sd2i = sd2i, n2i = n2i, data=ds2mhmonth_meta) #Calculate
yi and vi

#FE meta-analysis
(mhmonth_meta <- rbind(ds1mhmonth_meta, ds2mhmonth_meta))

##        m1i     sd1i n1i      m2i     sd2i n2i      yi     vi
## 1 5.566667 2.176415  30 5.251799 2.239609 139  0.3149 0.1940
## 2 4.844156 2.052373  77 4.904977 2.233021 221 -0.0608 0.0773

(mhmonth_vgdaysweek_meta <- rma(yi=yi, vi=vi, measure="MD",
method="FE", data=mhmonth_meta))

##
## Fixed-Effects Model (k = 2)
##
## Test for Heterogeneity:
## Q(df = 1) = 0.5204, p-val = 0.4707
##
## Model Results:
##
## estimate      se     zval     pval    ci.lb    ci.ub
##   0.0462   0.2351   0.1965   0.8442  -0.4145   0.5069
##
## ---
## Signif. codes:  0 '***' 0.001 '**' 0.01 '*' 0.05 '.' 0.1 ' ' 1
```

PTSD care - discharge

Replicated Effect

Hours per day

Study 1

```
(vghrsday_ptsddis_ds1 <- t.test(vghrsday ~ mhdis, data=ds1ptsd))

##
##  Welch Two Sample t-test
##
## data:  vghrsday by mhdis
## t = 0.66454, df = 64.56, p-value = 0.5087
## alternative hypothesis: true difference in means is not equal to 0
## 95 percent confidence interval:
##  -0.5226871  1.0438913
## sample estimates:
## mean in group 0 mean in group 1
##        2.756098        2.495495
```

Study 2

```
(vghrsday_ptsddis_ds2 <- t.test(vghrsday ~ mhdis, data=ds2ptsd))

##
##  Welch Two Sample t-test
##
## data:  vghrsday by mhdis
## t = -1.9646, df = 93.121, p-value = 0.05244
## alternative hypothesis: true difference in means is not equal to 0
## 95 percent confidence interval:
##  -1.90364795  0.01021166
## sample estimates:
## mean in group 0 mean in group 1
##        3.285714        4.232432
```

Hours per week

Study 1

```
(vghrsweek_ptsddis_ds1 <- t.test(vghrsweek ~ mhdis, data=ds1ptsd))

##
##  Welch Two Sample t-test
##
## data:  vghrsweek by mhdis
## t = 0.6953, df = 62.304, p-value = 0.4895
```

```
## alternative hypothesis: true difference in means is not equal to 0
## 95 percent confidence interval:
##  -3.958218  8.181026
## sample estimates:
## mean in group 0 mean in group 1
##        16.21951        14.10811
```

Study 2

```
(vghrsweek_ptsddis_ds2 <- t.test(vghrsweek ~ mhdis, data=ds2ptsd))

##
##  Welch Two Sample t-test
##
## data:  vghrsweek by mhdis
## t = -2.4482, df = 104.85, p-value = 0.01602
## alternative hypothesis: true difference in means is not equal to 0
## 95 percent confidence interval:
##  -13.96804  -1.46697
## sample estimates:
## mean in group 0 mean in group 1
##        16.30952        24.02703
```

Days per week

Study 1

```
(vgdaysweek_ptsddis_ds1 <- t.test(vgdaysweek ~ mhdis, data=ds1ptsd))

##
##  Welch Two Sample t-test
##
## data:  vgdaysweek by mhdis
## t = -0.67344, df = 70.145, p-value = 0.5029
## alternative hypothesis: true difference in means is not equal to 0
## 95 percent confidence interval:
##  -1.121152  0.555123
## sample estimates:
## mean in group 0 mean in group 1
##        4.951220        5.234234
```

Study 2

```
(vgdaysweek_ptsddis_ds2 <- t.test(vgdaysweek ~ mhdis, data=ds2ptsd))

##
##  Welch Two Sample t-test
##
```

```
## data:  vgdaysweek by mhdis
## t = -1.3444, df = 57.315, p-value = 0.1841
## alternative hypothesis: true difference in means is not equal to 0
## 95 percent confidence interval:
##  -1.3035798  0.2562181
## sample estimates:
## mean in group 0 mean in group 1
##        4.595238        5.118919
```

MD

Hours per day

```
#Study 1
ptsddis_yes_ds1_vghrsday <- subset(ptsddis_yes_ds1, select = c(vghrsday))
#Subset 1i data
ptsddis_vghrsday_m1i <- apply(ptsddis_yes_ds1_vghrsday, 2, mean)
#Calculate m1i
ptsddis_vghrsday_sd1i <- apply(ptsddis_yes_ds1_vghrsday, 2, sd) #Calculate
sd1i
ptsddis_vghrsday_n1i <- colSums(!is.na(ptsddis_yes_ds1_vghrsday))
#Calculate n1i
ptsddis_no_ds1_vghrsday <- subset(ptsddis_no_ds1, select = c(vghrsday))
#Subset 2i data
ptsddis_vghrsday_m2i <- apply(ptsddis_no_ds1_vghrsday, 2, mean)
#Calculate m2i
ptsddis_vghrsday_sd2i <- apply(ptsddis_no_ds1_vghrsday, 2, sd) #Calculate
sd2i
ptsddis_vghrsday_n2i <- colSums(!is.na(ptsddis_no_ds1_vghrsday))
#Calculate n2i
ds1ptsddis_meta <- data.frame(m1i = ptsddis_vghrsday_m1i, sd1i =
ptsddis_vghrsday_sd1i, n1i = ptsddis_vghrsday_n1i, m2i =
ptsddis_vghrsday_m2i, sd2i = ptsddis_vghrsday_sd2i, n2i =
ptsddis_vghrsday_n2i) #MA dataframe
ds1ptsddis_meta <- escalc(measure="MD", m1i = m1i, sd1i = sd1i, n1i =
n1i, m2i = m2i, sd2i = sd2i, n2i = n2i, data=ds1ptsddis_meta) #Calculate yi
and vi

#Study 2
ptsddis_yes_ds2_vghrsday <- subset(ptsddis_yes_ds2, select = c(vghrsday))
#Subset 1i data
ptsddis_vghrsday_m1i <- apply(ptsddis_yes_ds2_vghrsday, 2, mean)
#Calculate m1i
ptsddis_vghrsday_sd1i <- apply(ptsddis_yes_ds2_vghrsday, 2, sd) #Calculate
sd1i
ptsddis_vghrsday_n1i <- colSums(!is.na(ptsddis_yes_ds2_vghrsday))
#Calculate n1i
ptsddis_no_ds2_vghrsday <- subset(ptsddis_no_ds2, select = c(vghrsday))
#Subset 2i data
```

```
ptsddis_vghrsday_m2i <- apply(ptsddis_no_ds2_vghrsday, 2, mean)
#Calculate m2i
ptsddis_vghrsday_sd2i <- apply(ptsddis_no_ds2_vghrsday, 2, sd) #Calculate
sd2i
ptsddis_vghrsday_n2i <- colSums(!is.na(ptsddis_no_ds2_vghrsday))
#Calculate n2i
ds2ptsddis_meta <- data.frame(m1i = ptsddis_vghrsday_m1i, sd1i =
ptsddis_vghrsday_sd1i, n1i = ptsddis_vghrsday_n1i, m2i =
ptsddis_vghrsday_m2i, sd2i = ptsddis_vghrsday_sd2i, n2i =
ptsddis_vghrsday_n2i) #MA dataframe
ds2ptsddis_meta <- escalc(measure="MD", m1i = m1i, sd1i = sd1i, n1i =
n1i, m2i = m2i, sd2i = sd2i, n2i = n2i, data=ds2ptsddis_meta) #Calculate yi
and vi

#FE meta-analysis
(ptsddis_meta <- rbind(ds1ptsddis_meta, ds2ptsddis_meta))

##        m1i      sd1i n1i      m2i      sd2i n2i      yi     vi
## 1 2.495495 1.958078 111 2.756098 2.211114  41 -0.2606 0.1538
## 2 4.232432 3.923872 185 3.285714 2.501567  42  0.9467 0.2322

(ptsddis_vghrsday_meta <- rma(yi=yi, vi=vi, measure="MD", method="FE",
data=ptsddis_meta))

##
## Fixed-Effects Model (k = 2)
##
## Test for Heterogeneity:
## Q(df = 1) = 3.7761, p-val = 0.0520
##
## Model Results:
##
## estimate       se     zval     pval    ci.lb    ci.ub
##   0.2204   0.3042   0.7246   0.4687  -0.3758   0.8166
##
## ---
## Signif. codes:  0 '***' 0.001 '**' 0.01 '*' 0.05 '.' 0.1 ' ' 1
```

Hours per week

```
#Study 1
ptsddis_yes_ds1_vghrsweek <- subset(ptsddis_yes_ds1, select =
c(vghrsweek)) #Subset 1i data
ptsddis_vghrsweek_m1i <- apply(ptsddis_yes_ds1_vghrsweek, 2, mean)
#Calculate m1i
ptsddis_vghrsweek_sd1i <- apply(ptsddis_yes_ds1_vghrsweek, 2, sd)
#Calculate sd1i
ptsddis_vghrsweek_n1i <- colSums(!is.na(ptsddis_yes_ds1_vghrsweek))
#Calculate n1i
ptsddis_no_ds1_vghrsweek <- subset(ptsddis_no_ds1, select = c(vghrsweek))
```

```
#Subset 2i data
ptsddis_vghrsweek_m2i <- apply(ptsddis_no_ds1_vghrsweek, 2, mean)
#Calculate m2i
ptsddis_vghrsweek_sd2i <- apply(ptsddis_no_ds1_vghrsweek, 2, sd)
#Calculate sd2i
ptsddis_vghrsweek_n2i <- colSums(!is.na(ptsddis_no_ds1_vghrsweek))
#Calculate n2i
ds1ptsddis_meta <- data.frame(m1i = ptsddis_vghrsweek_m1i, sd1i =
ptsddis_vghrsweek_sd1i, n1i = ptsddis_vghrsweek_n1i, m2i =
ptsddis_vghrsweek_m2i, sd2i = ptsddis_vghrsweek_sd2i, n2i =
ptsddis_vghrsweek_n2i) #MA dataframe
ds1ptsddis_meta <- escalc(measure="MD", m1i = m1i, sd1i = sd1i, n1i =
n1i, m2i = m2i, sd2i = sd2i, n2i = n2i, data=ds1ptsddis_meta) #Calculate yi
and vi

#Study 2
ptsddis_yes_ds2_vghrsweek <- subset(ptsddis_yes_ds2, select =
c(vghrsweek)) #Subset 1i data
ptsddis_vghrsweek_m1i <- apply(ptsddis_yes_ds2_vghrsweek, 2, mean)
#Calculate m1i
ptsddis_vghrsweek_sd1i <- apply(ptsddis_yes_ds2_vghrsweek, 2, sd)
#Calculate sd1i
ptsddis_vghrsweek_n1i <- colSums(!is.na(ptsddis_yes_ds2_vghrsweek))
#Calculate n1i
ptsddis_no_ds2_vghrsweek <- subset(ptsddis_no_ds2, select = c(vghrsweek))
#Subset 2i data
ptsddis_vghrsweek_m2i <- apply(ptsddis_no_ds2_vghrsweek, 2, mean)
#Calculate m2i
ptsddis_vghrsweek_sd2i <- apply(ptsddis_no_ds2_vghrsweek, 2, sd)
#Calculate sd2i
ptsddis_vghrsweek_n2i <- colSums(!is.na(ptsddis_no_ds2_vghrsweek))
#Calculate n2i
ds2ptsddis_meta <- data.frame(m1i = ptsddis_vghrsweek_m1i, sd1i =
ptsddis_vghrsweek_sd1i, n1i = ptsddis_vghrsweek_n1i, m2i =
ptsddis_vghrsweek_m2i, sd2i = ptsddis_vghrsweek_sd2i, n2i =
ptsddis_vghrsweek_n2i) #MA dataframe
ds2ptsddis_meta <- escalc(measure="MD", m1i = m1i, sd1i = sd1i, n1i =
n1i, m2i = m2i, sd2i = sd2i, n2i = n2i, data=ds2ptsddis_meta) #Calculate yi
and vi

#FE meta-analysis
(ptsddis_meta <- rbind(ds1ptsddis_meta, ds2ptsddis_meta))

##        m1i     sd1i n1i      m2i     sd2i n2i      yi     vi
## 1 14.10811 14.61528 111 16.21951 17.29669  41 -2.1114 9.2213
## 2 24.02703 27.27107 185 16.30952 15.76425  42  7.7175 9.9370

(ptsddis_vghrsweek_meta <- rma(yi=yi, vi=vi, measure="MD", method="FE",
data=ptsddis_meta))
```

```
##
## Fixed-Effects Model (k = 2)
##
## Test for Heterogeneity:
## Q(df = 1) = 5.0426, p-val = 0.0247
##
## Model Results:
##
## estimate      se    zval    pval    ci.lb   ci.ub
##   2.6195  2.1870  1.1978  0.2310  -1.6669  6.9059
##
## ---
## Signif. codes:  0 '***' 0.001 '**' 0.01 '*' 0.05 '.' 0.1 ' ' 1
```

Days per week

```
#Study 1
ptsddis_yes_ds1_vgdaysweek <- subset(ptsddis_yes_ds1, select =
c(vgdaysweek)) #Subset 1i data
ptsddis_vgdaysweek_m1i <- apply(ptsddis_yes_ds1_vgdaysweek, 2, mean)
#Calculate m1i
ptsddis_vgdaysweek_sd1i <- apply(ptsddis_yes_ds1_vgdaysweek, 2, sd)
#Calculate sd1i
ptsddis_vgdaysweek_n1i <- colSums(!is.na(ptsddis_yes_ds1_vgdaysweek))
#Calculate n1i
ptsddis_no_ds1_vgdaysweek <- subset(ptsddis_no_ds1, select =
c(vgdaysweek)) #Subset 2i data
ptsddis_vgdaysweek_m2i <- apply(ptsddis_no_ds1_vgdaysweek, 2, mean)
#Calculate m2i
ptsddis_vgdaysweek_sd2i <- apply(ptsddis_no_ds1_vgdaysweek, 2, sd)
#Calculate sd2i
ptsddis_vgdaysweek_n2i <- colSums(!is.na(ptsddis_no_ds1_vgdaysweek))
#Calculate n2i
ds1ptsddis_meta <- data.frame(m1i = ptsddis_vgdaysweek_m1i, sd1i =
ptsddis_vgdaysweek_sd1i, n1i = ptsddis_vgdaysweek_n1i, m2i =
ptsddis_vgdaysweek_m2i, sd2i = ptsddis_vgdaysweek_sd2i, n2i =
ptsddis_vgdaysweek_n2i) #MA dataframe
ds1ptsddis_meta <- escalc(measure="MD", m1i = m1i, sd1i = sd1i, n1i =
n1i, m2i = m2i, sd2i = sd2i, n2i = n2i, data=ds1ptsddis_meta) #Calculate yi
and vi

#Study 2
ptsddis_yes_ds2_vgdaysweek <- subset(ptsddis_yes_ds2, select =
c(vgdaysweek)) #Subset 1i data
ptsddis_vgdaysweek_m1i <- apply(ptsddis_yes_ds2_vgdaysweek, 2, mean)
#Calculate m1i
ptsddis_vgdaysweek_sd1i <- apply(ptsddis_yes_ds2_vgdaysweek, 2, sd)
#Calculate sd1i
ptsddis_vgdaysweek_n1i <- colSums(!is.na(ptsddis_yes_ds2_vgdaysweek))
```

```
#Calculate n1i
ptsddis_no_ds2_vgdaysweek <- subset(ptsddis_no_ds2, select =
c(vgdaysweek)) #Subset 2i data
ptsddis_vgdaysweek_m2i <- apply(ptsddis_no_ds2_vgdaysweek, 2, mean)
#Calculate m2i
ptsddis_vgdaysweek_sd2i <- apply(ptsddis_no_ds2_vgdaysweek, 2, sd)
#Calculate sd2i
ptsddis_vgdaysweek_n2i <- colSums(!is.na(ptsddis_no_ds2_vgdaysweek))
#Calculate n2i
ds2ptsddis_meta <- data.frame(m1i = ptsddis_vgdaysweek_m1i, sd1i =
ptsddis_vgdaysweek_sd1i, n1i = ptsddis_vgdaysweek_n1i, m2i =
ptsddis_vgdaysweek_m2i, sd2i = ptsddis_vgdaysweek_sd2i, n2i =
ptsddis_vgdaysweek_n2i) #MA dataframe
ds2ptsddis_meta <- escalc(measure="MD", m1i = m1i, sd1i = sd1i, n1i =
n1i, m2i = m2i, sd2i = sd2i, n2i = n2i, data=ds2ptsddis_meta) #Calculate yi
and vi

#FE meta-analysis
(ptsddis_meta <- rbind(ds1ptsddis_meta, ds2ptsddis_meta))
```

```
##        m1i      sd1i n1i      m2i      sd2i n2i     yi     vi
## 1 5.234234 2.264167 111 4.951220 2.312479  41 0.2830 0.1766
## 2 5.118919 2.102514 185 4.595238 2.317058  42 0.5237 0.1517
```

```
(ptsddis_vgdaysweek_meta <- rma(yi=yi, vi=vi, measure="MD",
method="FE", data=ptsddis_meta))
```

```
##
## Fixed-Effects Model (k = 2)
##
## Test for Heterogeneity:
## Q(df = 1) = 0.1764, p-val = 0.6745
##
## Model Results:
##
## estimate      se     zval     pval    ci.lb    ci.ub
##   0.4125   0.2857   1.4438   0.1488  -0.1474   0.9724
##
## ---
## Signif. codes:  0 '***' 0.001 '**' 0.01 '*' 0.05 '.' 0.1 ' ' 1
```

PTSD care - year

Replicated Effect

Hours per day

Study 1

```
(vghrsday_ptsdyear_ds1 <- t.test(vghrsday ~ mhyear, data=ds1ptsd))

##
##  Welch Two Sample t-test
##
## data:  vghrsday by mhyear
## t = 1.4862, df = 146.23, p-value = 0.1394
## alternative hypothesis: true difference in means is not equal to 0
## 95 percent confidence interval:
##  -0.1599992  1.1303888
## sample estimates:
## mean in group 0 mean in group 1
##        2.805195        2.320000
```

Study 2

```
(vghrsday_ptsdyear_ds2 <- t.test(vghrsday ~ mhyear, data=ds2ptsd))

##
##  Welch Two Sample t-test
##
## data:  vghrsday by mhyear
## t = -0.33394, df = 177.32, p-value = 0.7388
## alternative hypothesis: true difference in means is not equal to 0
## 95 percent confidence interval:
##  -1.1420259  0.8114631
## sample estimates:
## mean in group 0 mean in group 1
##        3.948052        4.113333
```

Hours per week

Study 1

```
(vghrsweek_ptsdyear_ds1 <- t.test(vghrsweek ~ mhyear, data=ds1ptsd))

##
##  Welch Two Sample t-test
##
## data:  vghrsweek by mhyear
## t = 1.4665, df = 143.08, p-value = 0.1447
```

```
## alternative hypothesis: true difference in means is not equal to 0
## 95 percent confidence interval:
##  -1.261923  8.516988
## sample estimates:
## mean in group 0 mean in group 1
##        16.46753        12.84000
```

Study 2

```
(vghrsweek_ptsdyear_ds2 <- t.test(vghrsweek ~ mhyear, data=ds2ptsd))

##
##  Welch Two Sample t-test
##
## data:  vghrsweek by mhyear
## t = 0.044351, df = 168.57, p-value = 0.9647
## alternative hypothesis: true difference in means is not equal to 0
## 95 percent confidence interval:
##  -6.728306  7.037570
## sample estimates:
## mean in group 0 mean in group 1
##        22.70130        22.54667
```

Days per week

Study 1

```
(vgdaysweek_ptsdyear_ds1 <- t.test(vgdaysweek ~ mhyear, data=ds1ptsd))

##
##  Welch Two Sample t-test
##
## data:  vgdaysweek by mhyear
## t = -0.36734, df = 149.88, p-value = 0.7139
## alternative hypothesis: true difference in means is not equal to 0
## 95 percent confidence interval:
##  -0.8659945  0.5944794
## sample estimates:
## mean in group 0 mean in group 1
##        5.090909        5.226667
```

Study 2

```
(vgdaysweek_ptsdyear_ds2 <- t.test(vgdaysweek ~ mhyear, data=ds2ptsd))

##
##  Welch Two Sample t-test
##
```

```
## data:  vgdaysweek by mhyear
## t = 0.14589, df = 142.23, p-value = 0.8842
## alternative hypothesis: true difference in means is not equal to 0
## 95 percent confidence interval:
##  -0.5682531  0.6588159
## sample estimates:
## mean in group 0 mean in group 1
##        5.051948        5.006667
```

MD

Hours per day

```
#Study 1
ptsdyear_yes_ds1_vghrsday <- subset(ptsdyear_yes_ds1, select =
c(vghrsday)) #Subset 1i data
ptsdyear_vghrsday_m1i <- apply(ptsdyear_yes_ds1_vghrsday, 2, mean)
#Calculate m1i
ptsdyear_vghrsday_sd1i <- apply(ptsdyear_yes_ds1_vghrsday, 2, sd)
#Calculate sd1i
ptsdyear_vghrsday_n1i <- colSums(!is.na(ptsdyear_yes_ds1_vghrsday))
#Calculate n1i
ptsdyear_no_ds1_vghrsday <- subset(ptsdyear_no_ds1, select = c(vghrsday))
#Subset 2i data
ptsdyear_vghrsday_m2i <- apply(ptsdyear_no_ds1_vghrsday, 2, mean)
#Calculate m2i
ptsdyear_vghrsday_sd2i <- apply(ptsdyear_no_ds1_vghrsday, 2, sd)
#Calculate sd2i
ptsdyear_vghrsday_n2i <- colSums(!is.na(ptsdyear_no_ds1_vghrsday))
#Calculate n2i
ds1ptsdyear_meta <- data.frame(m1i = ptsdyear_vghrsday_m1i, sd1i =
ptsdyear_vghrsday_sd1i, n1i = ptsdyear_vghrsday_n1i, m2i =
ptsdyear_vghrsday_m2i, sd2i = ptsdyear_vghrsday_sd2i, n2i =
ptsdyear_vghrsday_n2i) #MA dataframe
ds1ptsdyear_meta <- escalc(measure="MD", m1i = m1i, sd1i = sd1i, n1i =
n1i, m2i = m2i, sd2i = sd2i, n2i = n2i, data=ds1ptsdyear_meta) #Calculate
yi and vi

#Study 2
ptsdyear_yes_ds2_vghrsday <- subset(ptsdyear_yes_ds2, select =
c(vghrsday)) #Subset 1i data
ptsdyear_vghrsday_m1i <- apply(ptsdyear_yes_ds2_vghrsday, 2, mean)
#Calculate m1i
ptsdyear_vghrsday_sd1i <- apply(ptsdyear_yes_ds2_vghrsday, 2, sd)
#Calculate sd1i
ptsdyear_vghrsday_n1i <- colSums(!is.na(ptsdyear_yes_ds2_vghrsday))
#Calculate n1i
ptsdyear_no_ds2_vghrsday <- subset(ptsdyear_no_ds2, select = c(vghrsday))
#Subset 2i data
```

```
ptsdyear_vghrsday_m2i <- apply(ptsdyear_no_ds2_vghrsday, 2, mean)
#Calculate m2i
ptsdyear_vghrsday_sd2i <- apply(ptsdyear_no_ds2_vghrsday, 2, sd)
#Calculate sd2i
ptsdyear_vghrsday_n2i <- colSums(!is.na(ptsdyear_no_ds2_vghrsday))
#Calculate n2i
ds2ptsdyear_meta <- data.frame(m1i = ptsdyear_vghrsday_m1i, sd1i =
ptsdyear_vghrsday_sd1i, n1i = ptsdyear_vghrsday_n1i, m2i =
ptsdyear_vghrsday_m2i, sd2i = ptsdyear_vghrsday_sd2i, n2i =
ptsdyear_vghrsday_n2i) #MA dataframe
ds2ptsdyear_meta <- escalc(measure="MD", m1i = m1i, sd1i = sd1i, n1i =
n1i, m2i = m2i, sd2i = sd2i, n2i = n2i, data=ds2ptsdyear_meta) #Calculate
yi and vi

#FE meta-analysis
(ptsdyear_meta <- rbind(ds1ptsdyear_meta, ds2ptsdyear_meta))

##        m1i      sd1i n1i      m2i      sd2i n2i      yi     vi
## 1 2.320000 1.817036  75 2.805195 2.194724  77 -0.4852 0.1066
## 2 4.113333 3.914414 150 3.948052 3.316213  77  0.1653 0.2450

(ptsdyear_vghrsday_meta <- rma(yi=yi, vi=vi, measure="MD", method="FE",
data=ptsdyear_meta))

##
## Fixed-Effects Model (k = 2)
##
## Test for Heterogeneity:
## Q(df = 1) = 1.2036, p-val = 0.2726
##
## Model Results:
##
## estimate      se     zval     pval    ci.lb    ci.ub
##  -0.2880   0.2725  -1.0568   0.2906  -0.8221   0.2461
##
## ---
## Signif. codes:  0 '***' 0.001 '**' 0.01 '*' 0.05 '.' 0.1 ' ' 1
```

Hours per week

```
#Study 1
ptsdyear_yes_ds1_vghrsweek <- subset(ptsdyear_yes_ds1, select =
c(vghrsweek)) #Subset 1i data
ptsdyear_vghrsweek_m1i <- apply(ptsdyear_yes_ds1_vghrsweek, 2, mean)
#Calculate m1i
ptsdyear_vghrsweek_sd1i <- apply(ptsdyear_yes_ds1_vghrsweek, 2, sd)
#Calculate sd1i
ptsdyear_vghrsweek_n1i <- colSums(!is.na(ptsdyear_yes_ds1_vghrsweek))
#Calculate n1i
ptsdyear_no_ds1_vghrsweek <- subset(ptsdyear_no_ds1, select =
```

```
c(vghrsweek)) #Subset 2i data
ptsdyear_vghrsweek_m2i <- apply(ptsdyear_no_ds1_vghrsweek, 2, mean)
#Calculate m2i
ptsdyear_vghrsweek_sd2i <- apply(ptsdyear_no_ds1_vghrsweek, 2, sd)
#Calculate sd2i
ptsdyear_vghrsweek_n2i <- colSums(!is.na(ptsdyear_no_ds1_vghrsweek))
#Calculate n2i
ds1ptsdyear_meta <- data.frame(m1i = ptsdyear_vghrsweek_m1i, sd1i =
ptsdyear_vghrsweek_sd1i, n1i = ptsdyear_vghrsweek_n1i, m2i =
ptsdyear_vghrsweek_m2i, sd2i = ptsdyear_vghrsweek_sd2i, n2i =
ptsdyear_vghrsweek_n2i) #MA dataframe
ds1ptsdyear_meta <- escalc(measure="MD", m1i = m1i, sd1i = sd1i, n1i =
n1i, m2i = m2i, sd2i = sd2i, n2i = n2i, data=ds1ptsdyear_meta) #Calculate
yi and vi

#Study 2
ptsdyear_yes_ds2_vghrsweek <- subset(ptsdyear_yes_ds2, select =
c(vghrsweek)) #Subset 1i data
ptsdyear_vghrsweek_m1i <- apply(ptsdyear_yes_ds2_vghrsweek, 2, mean)
#Calculate m1i
ptsdyear_vghrsweek_sd1i <- apply(ptsdyear_yes_ds2_vghrsweek, 2, sd)
#Calculate sd1i
ptsdyear_vghrsweek_n1i <- colSums(!is.na(ptsdyear_yes_ds2_vghrsweek))
#Calculate n1i
ptsdyear_no_ds2_vghrsweek <- subset(ptsdyear_no_ds2, select =
c(vghrsweek)) #Subset 2i data
ptsdyear_vghrsweek_m2i <- apply(ptsdyear_no_ds2_vghrsweek, 2, mean)
#Calculate m2i
ptsdyear_vghrsweek_sd2i <- apply(ptsdyear_no_ds2_vghrsweek, 2, sd)
#Calculate sd2i
ptsdyear_vghrsweek_n2i <- colSums(!is.na(ptsdyear_no_ds2_vghrsweek))
#Calculate n2i
ds2ptsdyear_meta <- data.frame(m1i = ptsdyear_vghrsweek_m1i, sd1i =
ptsdyear_vghrsweek_sd1i, n1i = ptsdyear_vghrsweek_n1i, m2i =
ptsdyear_vghrsweek_m2i, sd2i = ptsdyear_vghrsweek_sd2i, n2i =
ptsdyear_vghrsweek_n2i) #MA dataframe
ds2ptsdyear_meta <- escalc(measure="MD", m1i = m1i, sd1i = sd1i, n1i =
n1i, m2i = m2i, sd2i = sd2i, n2i = n2i, data=ds2ptsdyear_meta) #Calculate
yi and vi

#FE meta-analysis
(ptsdyear_meta <- rbind(ds1ptsdyear_meta, ds2ptsdyear_meta))

##        m1i     sd1i n1i      m2i     sd2i n2i      yi      vi
## 1 12.84000 13.26348  75 16.46753 17.04451  77 -3.6275  6.1185
## 2 22.54667 26.61876 150 22.70130 23.92261  77 -0.1546 12.1561

(ptsdyear_vghrsweek_meta <- rma(yi=yi, vi=vi, measure="MD",
method="FE", data=ptsdyear_meta))
```

```
##
## Fixed-Effects Model (k = 2)
##
## Test for Heterogeneity:
## Q(df = 1) = 0.6600, p-val = 0.4166
##
## Model Results:
##
## estimate      se     zval     pval    ci.lb    ci.ub
##  -2.4648   2.0174  -1.2217   0.2218  -6.4188   1.4893
##
## ---
## Signif. codes:  0 '***' 0.001 '**' 0.01 '*' 0.05 '.' 0.1 ' ' 1
```

Days per week

```
#Study 1
ptsdyear_yes_ds1_vgdaysweek <- subset(ptsdyear_yes_ds1, select =
c(vgdaysweek)) #Subset 1i data
ptsdyear_vgdaysweek_m1i <- apply(ptsdyear_yes_ds1_vgdaysweek, 2,
mean) #Calculate m1i
ptsdyear_vgdaysweek_sd1i <- apply(ptsdyear_yes_ds1_vgdaysweek, 2, sd)
#Calculate sd1i
ptsdyear_vgdaysweek_n1i <- colSums(!is.na(ptsdyear_yes_ds1_vgdaysweek))
#Calculate n1i
ptsdyear_no_ds1_vgdaysweek <- subset(ptsdyear_no_ds1, select =
c(vgdaysweek)) #Subset 2i data
ptsdyear_vgdaysweek_m2i <- apply(ptsdyear_no_ds1_vgdaysweek, 2, mean)
#Calculate m2i
ptsdyear_vgdaysweek_sd2i <- apply(ptsdyear_no_ds1_vgdaysweek, 2, sd)
#Calculate sd2i
ptsdyear_vgdaysweek_n2i <- colSums(!is.na(ptsdyear_no_ds1_vgdaysweek))
#Calculate n2i
ds1ptsdyear_meta <- data.frame(m1i = ptsdyear_vgdaysweek_m1i, sd1i =
ptsdyear_vgdaysweek_sd1i, n1i = ptsdyear_vgdaysweek_n1i, m2i =
ptsdyear_vgdaysweek_m2i, sd2i = ptsdyear_vgdaysweek_sd2i, n2i =
ptsdyear_vgdaysweek_n2i) #MA dataframe
ds1ptsdyear_meta <- escalc(measure="MD", m1i = m1i, sd1i = sd1i, n1i =
n1i, m2i = m2i, sd2i = sd2i, n2i = n2i, data=ds1ptsdyear_meta) #Calculate
yi and vi

#Study 2
ptsdyear_yes_ds2_vgdaysweek <- subset(ptsdyear_yes_ds2, select =
c(vgdaysweek)) #Subset 1i data
ptsdyear_vgdaysweek_m1i <- apply(ptsdyear_yes_ds2_vgdaysweek, 2,
mean) #Calculate m1i
ptsdyear_vgdaysweek_sd1i <- apply(ptsdyear_yes_ds2_vgdaysweek, 2, sd)
#Calculate sd1i
ptsdyear_vgdaysweek_n1i <- colSums(!is.na(ptsdyear_yes_ds2_vgdaysweek))
```

```r
#Calculate n1i
ptsdyear_no_ds2_vgdaysweek <- subset(ptsdyear_no_ds2, select =
c(vgdaysweek)) #Subset 2i data
ptsdyear_vgdaysweek_m2i <- apply(ptsdyear_no_ds2_vgdaysweek, 2, mean)
#Calculate m2i
ptsdyear_vgdaysweek_sd2i <- apply(ptsdyear_no_ds2_vgdaysweek, 2, sd)
#Calculate sd2i
ptsdyear_vgdaysweek_n2i <- colSums(!is.na(ptsdyear_no_ds2_vgdaysweek))
#Calculate n2i
ds2ptsdyear_meta <- data.frame(m1i = ptsdyear_vgdaysweek_m1i, sd1i =
ptsdyear_vgdaysweek_sd1i, n1i = ptsdyear_vgdaysweek_n1i, m2i =
ptsdyear_vgdaysweek_m2i, sd2i = ptsdyear_vgdaysweek_sd2i, n2i =
ptsdyear_vgdaysweek_n2i) #MA dataframe
ds2ptsdyear_meta <- escalc(measure="MD", m1i = m1i, sd1i = sd1i, n1i =
n1i, m2i = m2i, sd2i = sd2i, n2i = n2i, data=ds2ptsdyear_meta) #Calculate
yi and vi

#FE meta-analysis
(ptsdyear_meta <- rbind(ds1ptsdyear_meta, ds2ptsdyear_meta))

##        m1i     sd1i n1i      m2i     sd2i n2i      yi     vi
## 1 5.226667 2.215262  75 5.090909 2.340613  77  0.1358 0.1366
## 2 5.006667 2.087022 150 5.051948 2.276286  77 -0.0453 0.0963

(ptsdyear_vgdaysweek_meta <- rma(yi=yi, vi=vi, measure="MD",
method="FE", data=ptsdyear_meta))

##
## Fixed-Effects Model (k = 2)
##
## Test for Heterogeneity:
## Q(df = 1) = 0.1407, p-val = 0.7076
##
## Model Results:
##
## estimate      se     zval     pval    ci.lb    ci.ub
##   0.0296   0.2377   0.1245   0.9009  -0.4362   0.4954
##
## ---
## Signif. codes:  0 '***' 0.001 '**' 0.01 '*' 0.05 '.' 0.1 ' ' 1
```

PTSD care - month

Replicated Effect

Hours per day

Study 1

```
(vghrsday_ptsdmonth_ds1 <- t.test(vghrsday ~ mhmonth, data=ds1ptsd))

##
##  Welch Two Sample t-test
##
## data:  vghrsday by mhmonth
## t = -1.2971, df = 38.121, p-value = 0.2024
## alternative hypothesis: true difference in means is not equal to 0
## 95 percent confidence interval:
##  -1.4749509  0.3228772
## sample estimates:
## mean in group 0 mean in group 1
##        2.459677        3.035714
```

Study 2

```
(vghrsday_ptsdmonth_ds2 <- t.test(vghrsday ~ mhmonth, data=ds2ptsd))

##
##  Welch Two Sample t-test
##
## data:  vghrsday by mhmonth
## t = 0.95147, df = 175.14, p-value = 0.3427
## alternative hypothesis: true difference in means is not equal to 0
## 95 percent confidence interval:
##  -0.4883524  1.3975426
## sample estimates:
## mean in group 0 mean in group 1
##        4.197452        3.742857
```

Hours per week

Study 1

```
(vghrsweek_ptsdmonth_ds1 <- t.test(vghrsweek ~ mhmonth, data=ds1ptsd))

##
##  Welch Two Sample t-test
##
## data:  vghrsweek by mhmonth
## t = -1.0484, df = 38.484, p-value = 0.301
```

```
## alternative hypothesis: true difference in means is not equal to 0
## 95 percent confidence interval:
##  -10.265701   3.258788
## sample estimates:
## mean in group 0 mean in group 1
##        14.03226        17.53571
```

Study 2

```
(vghrsweek_ptsdmonth_ds2 <- t.test(vghrsweek ~ mhmonth, data=ds2ptsd))
```

```
##
##  Welch Two Sample t-test
##
## data:  vghrsweek by mhmonth
## t = 1.6154, df = 174.39, p-value = 0.108
## alternative hypothesis: true difference in means is not equal to 0
## 95 percent confidence interval:
##  -1.18153 11.83704
## sample estimates:
## mean in group 0 mean in group 1
##        24.24204        18.91429
```

Days per week

Study 1

```
(vgdaysweek_ptsdmonth_ds1 <- t.test(vgdaysweek ~ mhmonth,
data=ds1ptsd))
```

```
##
##  Welch Two Sample t-test
##
## data:  vgdaysweek by mhmonth
## t = -0.8042, df = 41.002, p-value = 0.4259
## alternative hypothesis: true difference in means is not equal to 0
## 95 percent confidence interval:
##  -1.3187332  0.5675812
## sample estimates:
## mean in group 0 mean in group 1
##        5.088710        5.464286
```

Study 2

```
(vgdaysweek_ptsdmonth_ds2 <- t.test(vgdaysweek ~ mhmonth,
data=ds2ptsd))
```

```
##
##  Welch Two Sample t-test
##
## data:  vgdaysweek by mhmonth
## t = 1.1234, df = 137.24, p-value = 0.2632
## alternative hypothesis: true difference in means is not equal to 0
## 95 percent confidence interval:
##  -0.2597604  0.9431089
## sample estimates:
## mean in group 0 mean in group 1
##        5.127389        4.785714
```

MD

Hours per day

```
#Study 1
ptsdmonth_yes_ds1_vghrsday <- subset(ptsdmonth_yes_ds1, select =
c(vghrsday)) #Subset 1i data
ptsdmonth_vghrsday_m1i <- apply(ptsdmonth_yes_ds1_vghrsday, 2, mean)
#Calculate m1i
ptsdmonth_vghrsday_sd1i <- apply(ptsdmonth_yes_ds1_vghrsday, 2, sd)
#Calculate sd1i
ptsdmonth_vghrsday_n1i <- colSums(!is.na(ptsdmonth_yes_ds1_vghrsday))
#Calculate n1i
ptsdmonth_no_ds1_vghrsday <- subset(ptsdmonth_no_ds1, select =
c(vghrsday)) #Subset 2i data
ptsdmonth_vghrsday_m2i <- apply(ptsdmonth_no_ds1_vghrsday, 2, mean)
#Calculate m2i
ptsdmonth_vghrsday_sd2i <- apply(ptsdmonth_no_ds1_vghrsday, 2, sd)
#Calculate sd2i
ptsdmonth_vghrsday_n2i <- colSums(!is.na(ptsdmonth_no_ds1_vghrsday))
#Calculate n2i
ds1ptsdmonth_meta <- data.frame(m1i = ptsdmonth_vghrsday_m1i, sd1i =
ptsdmonth_vghrsday_sd1i, n1i = ptsdmonth_vghrsday_n1i, m2i =
ptsdmonth_vghrsday_m2i, sd2i = ptsdmonth_vghrsday_sd2i, n2i =
ptsdmonth_vghrsday_n2i) #MA dataframe
ds1ptsdmonth_meta <- escalc(measure="MD", m1i = m1i, sd1i = sd1i, n1i =
n1i, m2i = m2i, sd2i = sd2i, n2i = n2i, data=ds1ptsdmonth_meta) #Calculate
yi and vi

#Study 2
ptsdmonth_yes_ds2_vghrsday <- subset(ptsdmonth_yes_ds2, select =
c(vghrsday)) #Subset 1i data
ptsdmonth_vghrsday_m1i <- apply(ptsdmonth_yes_ds2_vghrsday, 2, mean)
#Calculate m1i
ptsdmonth_vghrsday_sd1i <- apply(ptsdmonth_yes_ds2_vghrsday, 2, sd)
#Calculate sd1i
ptsdmonth_vghrsday_n1i <- colSums(!is.na(ptsdmonth_yes_ds2_vghrsday))
```

```
#Calculate n1i
ptsdmonth_no_ds2_vghrsday <- subset(ptsdmonth_no_ds2, select =
c(vghrsday)) #Subset 2i data
ptsdmonth_vghrsday_m2i <- apply(ptsdmonth_no_ds2_vghrsday, 2, mean)
#Calculate m2i
ptsdmonth_vghrsday_sd2i <- apply(ptsdmonth_no_ds2_vghrsday, 2, sd)
#Calculate sd2i
ptsdmonth_vghrsday_n2i <- colSums(!is.na(ptsdmonth_no_ds2_vghrsday))
#Calculate n2i
ds2ptsdmonth_meta <- data.frame(m1i = ptsdmonth_vghrsday_m1i, sd1i =
ptsdmonth_vghrsday_sd1i, n1i = ptsdmonth_vghrsday_n1i, m2i =
ptsdmonth_vghrsday_m2i, sd2i = ptsdmonth_vghrsday_sd2i, n2i =
ptsdmonth_vghrsday_n2i) #MA dataframe
ds2ptsdmonth_meta <- escalc(measure="MD", m1i = m1i, sd1i = sd1i, n1i =
n1i, m2i = m2i, sd2i = sd2i, n2i = n2i, data=ds2ptsdmonth_meta) #Calculate
yi and vi

#FE meta-analysis
(ptsdmonth_meta <- rbind(ds1ptsdmonth_meta, ds2ptsdmonth_meta))

##        m1i      sd1i n1i      m2i      sd2i n2i      yi     vi
## 1 3.035714 2.151350  28 2.459677 1.989400 124  0.5760 0.1972
## 2 3.742857 2.971779  70 4.197452 4.003908 157 -0.4546 0.2283

(ptsdmonth_vghrsday_meta <- rma(yi=yi, vi=vi, measure="MD",
method="FE", data=ptsdmonth_meta))

##
## Fixed-Effects Model (k = 2)
##
## Test for Heterogeneity:
## Q(df = 1) = 2.4964, p-val = 0.1141
##
## Model Results:
##
## estimate      se     zval     pval    ci.lb    ci.ub
##   0.0983   0.3253   0.3023   0.7624  -0.5392   0.7359
##
## ---
## Signif. codes:  0 '***' 0.001 '**' 0.01 '*' 0.05 '.' 0.1 ' ' 1
```

Hours per week

```
#Study 1
ptsdmonth_yes_ds1_vghrsweek <- subset(ptsdmonth_yes_ds1, select =
c(vghrsweek)) #Subset 1i data
ptsdmonth_vghrsweek_m1i <- apply(ptsdmonth_yes_ds1_vghrsweek, 2,
mean) #Calculate m1i
ptsdmonth_vghrsweek_sd1i <- apply(ptsdmonth_yes_ds1_vghrsweek, 2, sd)
#Calculate sd1i
```

```
ptsdmonth_vghrsweek_n1i <- colSums(!
is.na(ptsdmonth_yes_ds1_vghrsweek)) #Calculate n1i
ptsdmonth_no_ds1_vghrsweek <- subset(ptsdmonth_no_ds1, select =
c(vghrsweek)) #Subset 2i data
ptsdmonth_vghrsweek_m2i <- apply(ptsdmonth_no_ds1_vghrsweek, 2, mean)
#Calculate m2i
ptsdmonth_vghrsweek_sd2i <- apply(ptsdmonth_no_ds1_vghrsweek, 2, sd)
#Calculate sd2i
ptsdmonth_vghrsweek_n2i <- colSums(!is.na(ptsdmonth_no_ds1_vghrsweek))
#Calculate n2i
ds1ptsdmonth_meta <- data.frame(m1i = ptsdmonth_vghrsweek_m1i, sd1i =
ptsdmonth_vghrsweek_sd1i, n1i = ptsdmonth_vghrsweek_n1i, m2i =
ptsdmonth_vghrsweek_m2i, sd2i = ptsdmonth_vghrsweek_sd2i, n2i =
ptsdmonth_vghrsweek_n2i) #MA dataframe
ds1ptsdmonth_meta <- escalc(measure="MD", m1i = m1i, sd1i = sd1i, n1i =
n1i, m2i = m2i, sd2i = sd2i, n2i = n2i, data=ds1ptsdmonth_meta) #Calculate
yi and vi

#Study 2
ptsdmonth_yes_ds2_vghrsweek <- subset(ptsdmonth_yes_ds2, select =
c(vghrsweek)) #Subset 1i data
ptsdmonth_vghrsweek_m1i <- apply(ptsdmonth_yes_ds2_vghrsweek, 2,
mean) #Calculate m1i
ptsdmonth_vghrsweek_sd1i <- apply(ptsdmonth_yes_ds2_vghrsweek, 2, sd)
#Calculate sd1i
ptsdmonth_vghrsweek_n1i <- colSums(!
is.na(ptsdmonth_yes_ds2_vghrsweek)) #Calculate n1i
ptsdmonth_no_ds2_vghrsweek <- subset(ptsdmonth_no_ds2, select =
c(vghrsweek)) #Subset 2i data
ptsdmonth_vghrsweek_m2i <- apply(ptsdmonth_no_ds2_vghrsweek, 2, mean)
#Calculate m2i
ptsdmonth_vghrsweek_sd2i <- apply(ptsdmonth_no_ds2_vghrsweek, 2, sd)
#Calculate sd2i
ptsdmonth_vghrsweek_n2i <- colSums(!is.na(ptsdmonth_no_ds2_vghrsweek))
#Calculate n2i
ds2ptsdmonth_meta <- data.frame(m1i = ptsdmonth_vghrsweek_m1i, sd1i =
ptsdmonth_vghrsweek_sd1i, n1i = ptsdmonth_vghrsweek_n1i, m2i =
ptsdmonth_vghrsweek_m2i, sd2i = ptsdmonth_vghrsweek_sd2i, n2i =
ptsdmonth_vghrsweek_n2i) #MA dataframe
ds2ptsdmonth_meta <- escalc(measure="MD", m1i = m1i, sd1i = sd1i, n1i =
n1i, m2i = m2i, sd2i = sd2i, n2i = n2i, data=ds2ptsdmonth_meta) #Calculate
yi and vi

#FE meta-analysis
(ptsdmonth_meta <- rbind(ds1ptsdmonth_meta, ds2ptsdmonth_meta))

##        m1i     sd1i n1i      m2i     sd2i n2i      yi      vi
## 1 17.53571 16.14857  28 14.03226 15.16196 124  3.5035 11.1674
## 2 18.91429 20.55797  70 24.24204 27.56517 157 -5.3278 10.8773
```

```
(ptsdmonth_vghrsweek_meta <- rma(yi=yi, vi=vi, measure="MD",
method="FE", data=ptsdmonth_meta))

##
## Fixed-Effects Model (k = 2)
##
## Test for Heterogeneity:
## Q(df = 1) = 3.5378, p-val = 0.0600
##
## Model Results:
##
## estimate      se     zval    pval    ci.lb    ci.ub
##  -0.9702  2.3474  -0.4133  0.6794  -5.5710   3.6305
##
## ---
## Signif. codes:  0 '***' 0.001 '**' 0.01 '*' 0.05 '.' 0.1 ' ' 1
```

Days per week

```
#Study 1
ptsdmonth_yes_ds1_vgdaysweek <- subset(ptsdmonth_yes_ds1, select =
c(vgdaysweek)) #Subset 1i data
ptsdmonth_vgdaysweek_m1i <- apply(ptsdmonth_yes_ds1_vgdaysweek, 2,
mean) #Calculate m1i
ptsdmonth_vgdaysweek_sd1i <- apply(ptsdmonth_yes_ds1_vgdaysweek, 2,
sd) #Calculate sd1i
ptsdmonth_vgdaysweek_n1i <- colSums(!
is.na(ptsdmonth_yes_ds1_vgdaysweek)) #Calculate n1i
ptsdmonth_no_ds1_vgdaysweek <- subset(ptsdmonth_no_ds1, select =
c(vgdaysweek)) #Subset 2i data
ptsdmonth_vgdaysweek_m2i <- apply(ptsdmonth_no_ds1_vgdaysweek, 2,
mean) #Calculate m2i
ptsdmonth_vgdaysweek_sd2i <- apply(ptsdmonth_no_ds1_vgdaysweek, 2,
sd) #Calculate sd2i
ptsdmonth_vgdaysweek_n2i <- colSums(!
is.na(ptsdmonth_no_ds1_vgdaysweek)) #Calculate n2i
ds1ptsdmonth_meta <- data.frame(m1i = ptsdmonth_vgdaysweek_m1i, sd1i
= ptsdmonth_vgdaysweek_sd1i, n1i = ptsdmonth_vgdaysweek_n1i, m2i =
ptsdmonth_vgdaysweek_m2i, sd2i = ptsdmonth_vgdaysweek_sd2i, n2i =
ptsdmonth_vgdaysweek_n2i) #MA dataframe
ds1ptsdmonth_meta <- escalc(measure="MD", m1i = m1i, sd1i = sd1i, n1i =
n1i, m2i = m2i, sd2i = sd2i, n2i = n2i, data=ds1ptsdmonth_meta) #Calculate
yi and vi

#Study 2
ptsdmonth_yes_ds2_vgdaysweek <- subset(ptsdmonth_yes_ds2, select =
c(vgdaysweek)) #Subset 1i data
ptsdmonth_vgdaysweek_m1i <- apply(ptsdmonth_yes_ds2_vgdaysweek, 2,
mean) #Calculate m1i
```

```r
ptsdmonth_vgdaysweek_sd1i <- apply(ptsdmonth_yes_ds2_vgdaysweek, 2,
sd) #Calculate sd1i
ptsdmonth_vgdaysweek_n1i <- colSums(!
is.na(ptsdmonth_yes_ds2_vgdaysweek)) #Calculate n1i
ptsdmonth_no_ds2_vgdaysweek <- subset(ptsdmonth_no_ds2, select =
c(vgdaysweek)) #Subset 2i data
ptsdmonth_vgdaysweek_m2i <- apply(ptsdmonth_no_ds2_vgdaysweek, 2,
mean) #Calculate m2i
ptsdmonth_vgdaysweek_sd2i <- apply(ptsdmonth_no_ds2_vgdaysweek, 2,
sd) #Calculate sd2i
ptsdmonth_vgdaysweek_n2i <- colSums(!
is.na(ptsdmonth_no_ds2_vgdaysweek)) #Calculate n2i
ds2ptsdmonth_meta <- data.frame(m1i = ptsdmonth_vgdaysweek_m1i, sd1i
= ptsdmonth_vgdaysweek_sd1i, n1i = ptsdmonth_vgdaysweek_n1i, m2i =
ptsdmonth_vgdaysweek_m2i, sd2i = ptsdmonth_vgdaysweek_sd2i, n2i =
ptsdmonth_vgdaysweek_n2i) #MA dataframe
ds2ptsdmonth_meta <- escalc(measure="MD", m1i = m1i, sd1i = sd1i, n1i =
n1i, m2i = m2i, sd2i = sd2i, n2i = n2i, data=ds2ptsdmonth_meta) #Calculate
yi and vi

#FE meta-analysis
(ptsdmonth_meta <- rbind(ds1ptsdmonth_meta, ds2ptsdmonth_meta))

##        m1i     sd1i n1i      m2i     sd2i n2i      yi     vi
## 1 5.464286 2.219145  28 5.088710 2.288224 124  0.3756 0.2181
## 2 4.785714 2.091341  70 5.127389 2.171276 157 -0.3417 0.0925

(ptsdmonth_vgdaysweek_meta <- rma(yi=yi, vi=vi, measure="MD",
method="FE", data=ptsdmonth_meta))

##
## Fixed-Effects Model (k = 2)
##
## Test for Heterogeneity:
## Q(df = 1) = 1.6562, p-val = 0.1981
##
## Model Results:
##
## estimate      se     zval     pval    ci.lb    ci.ub
##  -0.1281   0.2549  -0.5024   0.6154  -0.6276   0.3715
##
## ---
## Signif. codes:  0 '***' 0.001 '**' 0.01 '*' 0.05 '.' 0.1 ' ' 1
```

Depression care - discharge

Replicated Effect

Hours per day

Study 1

```
(vghrsday_depressiondis_ds1 <- t.test(vghrsday ~ mhdis,
data=ds1depression))

##
##  Welch Two Sample t-test
##
## data:  vghrsday by mhdis
## t = 0.68063, df = 84.106, p-value = 0.498
## alternative hypothesis: true difference in means is not equal to 0
## 95 percent confidence interval:
##  -0.4493641  0.9170512
## sample estimates:
## mean in group 0 mean in group 1
##        2.775510        2.541667
```

Study 2

```
(vghrsday_depressiondis_ds2 <- t.test(vghrsday ~ mhdis,
data=ds2depression))

##
##  Welch Two Sample t-test
##
## data:  vghrsday by mhdis
## t = -2.3638, df = 179.93, p-value = 0.01916
## alternative hypothesis: true difference in means is not equal to 0
## 95 percent confidence interval:
##  -1.6678833 -0.1501875
## sample estimates:
## mean in group 0 mean in group 1
##        3.206349        4.115385
```

Hours per week

Study 1

```
(vghrsweek_depressiondis_ds1 <- t.test(vghrsweek ~ mhdis,
data=ds1depression))

##
##  Welch Two Sample t-test
```

```
##
## data:  vghrsweek by mhdis
## t = 0.70153, df = 82.026, p-value = 0.485
## alternative hypothesis: true difference in means is not equal to 0
## 95 percent confidence interval:
##  -3.378858  7.060150
## sample estimates:
## mean in group 0 mean in group 1
##        16.44898        14.60833
```

Study 2

```
(vghrsweek_depressiondis_ds2 <- t.test(vghrsweek ~ mhdis,
data=ds2depression))
```

```
##
##  Welch Two Sample t-test
##
## data:  vghrsweek by mhdis
## t = -2.3401, df = 185.02, p-value = 0.02034
## alternative hypothesis: true difference in means is not equal to 0
## 95 percent confidence interval:
##  -11.3229771  -0.9641109
## sample estimates:
## mean in group 0 mean in group 1
##        16.42857        22.57212
```

Days per week

Study 1

```
(vgdaysweek_depressiondis_ds1 <- t.test(vgdaysweek ~ mhdis,
data=ds1depression))
```

```
##
##  Welch Two Sample t-test
##
## data:  vgdaysweek by mhdis
## t = -0.3834, df = 88.029, p-value = 0.7024
## alternative hypothesis: true difference in means is not equal to 0
## 95 percent confidence interval:
##  -0.9022650  0.6104283
## sample estimates:
## mean in group 0 mean in group 1
##        5.204082        5.350000
```

Study 2

```
(vgdaysweek_depressiondis_ds2 <- t.test(vgdaysweek ~ mhdis,
data=ds2depression))

##
##  Welch Two Sample t-test
##
## data:  vgdaysweek by mhdis
## t = -0.81695, df = 95.151, p-value = 0.416
## alternative hypothesis: true difference in means is not equal to 0
## 95 percent confidence interval:
##  -0.9239930  0.3852262
## sample estimates:
## mean in group 0 mean in group 1
##        4.682540        4.951923
```

MD

Hours per day

```
#Study 1
depressiondis_yes_ds1_vghrsday <- subset(depressiondis_yes_ds1, select =
c(vghrsday)) #Subset 1i data
depressiondis_vghrsday_m1i <- apply(depressiondis_yes_ds1_vghrsday, 2,
mean) #Calculate m1i
depressiondis_vghrsday_sd1i <- apply(depressiondis_yes_ds1_vghrsday, 2,
sd) #Calculate sd1i
depressiondis_vghrsday_n1i <- colSums(!
is.na(depressiondis_yes_ds1_vghrsday)) #Calculate n1i
depressiondis_no_ds1_vghrsday <- subset(depressiondis_no_ds1, select =
c(vghrsday)) #Subset 2i data
depressiondis_vghrsday_m2i <- apply(depressiondis_no_ds1_vghrsday, 2,
mean) #Calculate m2i
depressiondis_vghrsday_sd2i <- apply(depressiondis_no_ds1_vghrsday, 2, sd)
#Calculate sd2i
depressiondis_vghrsday_n2i <- colSums(!
is.na(depressiondis_no_ds1_vghrsday)) #Calculate n2i
ds1depressiondis_meta <- data.frame(m1i = depressiondis_vghrsday_m1i,
sd1i = depressiondis_vghrsday_sd1i, n1i = depressiondis_vghrsday_n1i, m2i
= depressiondis_vghrsday_m2i, sd2i = depressiondis_vghrsday_sd2i, n2i =
depressiondis_vghrsday_n2i) #MA dataframe
ds1depressiondis_meta <- escalc(measure="MD", m1i = m1i, sd1i = sd1i, n1i
= n1i, m2i = m2i, sd2i = sd2i, n2i = n2i, data=ds1depressiondis_meta)
#Calculate yi and vi

#Study 2
depressiondis_yes_ds2_vghrsday <- subset(depressiondis_yes_ds2, select =
c(vghrsday)) #Subset 1i data
depressiondis_vghrsday_m1i <- apply(depressiondis_yes_ds2_vghrsday, 2,
```

```
mean) #Calculate m1i
depressiondis_vghrsday_sd1i <- apply(depressiondis_yes_ds2_vghrsday, 2,
sd) #Calculate sd1i
depressiondis_vghrsday_n1i <- colSums(!
is.na(depressiondis_yes_ds2_vghrsday)) #Calculate n1i
depressiondis_no_ds2_vghrsday <- subset(depressiondis_no_ds2, select =
c(vghrsday)) #Subset 2i data
depressiondis_vghrsday_m2i <- apply(depressiondis_no_ds2_vghrsday, 2,
mean) #Calculate m2i
depressiondis_vghrsday_sd2i <- apply(depressiondis_no_ds2_vghrsday, 2, sd)
#Calculate sd2i
depressiondis_vghrsday_n2i <- colSums(!
is.na(depressiondis_no_ds2_vghrsday)) #Calculate n2i
ds2depressiondis_meta <- data.frame(m1i = depressiondis_vghrsday_m1i,
sd1i = depressiondis_vghrsday_sd1i, n1i = depressiondis_vghrsday_n1i, m2i
= depressiondis_vghrsday_m2i, sd2i = depressiondis_vghrsday_sd2i, n2i =
depressiondis_vghrsday_n2i) #MA dataframe
ds2depressiondis_meta <- escalc(measure="MD", m1i = m1i, sd1i = sd1i, n1i
= n1i, m2i = m2i, sd2i = sd2i, n2i = n2i, data=ds2depressiondis_meta)
#Calculate yi and vi

#FE meta-analysis
(depressiondis_meta <- rbind(ds1depressiondis_meta,
ds2depressiondis_meta))

##        m1i    sd1i n1i      m2i     sd2i n2i      yi     vi
## 1 2.541667 1.93115 120 2.775510 2.064232  49 -0.2338 0.1180
## 2 4.115385 3.81532 208 3.206349 2.215479  63  0.9090 0.1479

(depressiondis_vghrsday_meta <- rma(yi=yi, vi=vi, measure="MD",
method="FE", data=depressiondis_meta))

##
## Fixed-Effects Model (k = 2)
##
## Test for Heterogeneity:
## Q(df = 1) = 4.9117, p-val = 0.0267
##
## Model Results:
##
## estimate      se     zval     pval    ci.lb    ci.ub
##   0.2734   0.2562   1.0672   0.2859  -0.2287   0.7756
##
## ---
## Signif. codes:  0 '***' 0.001 '**' 0.01 '*' 0.05 '.' 0.1 ' ' 1
```

Hours per week

```
#Study 1
depressiondis_yes_ds1_vghrsweek <- subset(depressiondis_yes_ds1, select =
```

```
c(vghrsweek)) #Subset 1i data
depressiondis_vghrsweek_m1i <- apply(depressiondis_yes_ds1_vghrsweek, 2,
mean) #Calculate m1i
depressiondis_vghrsweek_sd1i <- apply(depressiondis_yes_ds1_vghrsweek,
2, sd) #Calculate sd1i
depressiondis_vghrsweek_n1i <- colSums(!
is.na(depressiondis_yes_ds1_vghrsweek)) #Calculate n1i
depressiondis_no_ds1_vghrsweek <- subset(depressiondis_no_ds1, select =
c(vghrsweek)) #Subset 2i data
depressiondis_vghrsweek_m2i <- apply(depressiondis_no_ds1_vghrsweek, 2,
mean) #Calculate m2i
depressiondis_vghrsweek_sd2i <- apply(depressiondis_no_ds1_vghrsweek, 2,
sd) #Calculate sd2i
depressiondis_vghrsweek_n2i <- colSums(!
is.na(depressiondis_no_ds1_vghrsweek)) #Calculate n2i
ds1depressiondis_meta <- data.frame(m1i = depressiondis_vghrsweek_m1i,
sd1i = depressiondis_vghrsweek_sd1i, n1i = depressiondis_vghrsweek_n1i,
m2i = depressiondis_vghrsweek_m2i, sd2i = depressiondis_vghrsweek_sd2i,
n2i = depressiondis_vghrsweek_n2i) #MA dataframe
ds1depressiondis_meta <- escalc(measure="MD", m1i = m1i, sd1i = sd1i, n1i
= n1i, m2i = m2i, sd2i = sd2i, n2i = n2i, data=ds1depressiondis_meta)
#Calculate yi and vi

#Study 2
depressiondis_yes_ds2_vghrsweek <- subset(depressiondis_yes_ds2, select =
c(vghrsweek)) #Subset 1i data
depressiondis_vghrsweek_m1i <- apply(depressiondis_yes_ds2_vghrsweek, 2,
mean) #Calculate m1i
depressiondis_vghrsweek_sd1i <- apply(depressiondis_yes_ds2_vghrsweek,
2, sd) #Calculate sd1i
depressiondis_vghrsweek_n1i <- colSums(!
is.na(depressiondis_yes_ds2_vghrsweek)) #Calculate n1i
depressiondis_no_ds2_vghrsweek <- subset(depressiondis_no_ds2, select =
c(vghrsweek)) #Subset 2i data
depressiondis_vghrsweek_m2i <- apply(depressiondis_no_ds2_vghrsweek, 2,
mean) #Calculate m2i
depressiondis_vghrsweek_sd2i <- apply(depressiondis_no_ds2_vghrsweek, 2,
sd) #Calculate sd2i
depressiondis_vghrsweek_n2i <- colSums(!
is.na(depressiondis_no_ds2_vghrsweek)) #Calculate n2i
ds2depressiondis_meta <- data.frame(m1i = depressiondis_vghrsweek_m1i,
sd1i = depressiondis_vghrsweek_sd1i, n1i = depressiondis_vghrsweek_n1i,
m2i = depressiondis_vghrsweek_m2i, sd2i = depressiondis_vghrsweek_sd2i,
n2i = depressiondis_vghrsweek_n2i) #MA dataframe
ds2depressiondis_meta <- escalc(measure="MD", m1i = m1i, sd1i = sd1i, n1i
= n1i, m2i = m2i, sd2i = sd2i, n2i = n2i, data=ds2depressiondis_meta)
#Calculate yi and vi

#FE meta-analysis
```

```
(depressiondis_meta <- rbind(ds1depressiondis_meta,
ds2depressiondis_meta))

##        m1i     sd1i n1i      m2i     sd2i n2i      yi     vi
## 1 14.60833 14.42704 120 16.44898 15.88508  49 -1.8406 6.8842
## 2 22.57212 26.38995 208 16.42857 14.94259  63  6.1435 6.8924

(depressiondis_vghrsweek_meta <- rma(yi=yi, vi=vi, measure="MD",
method="FE", data=depressiondis_meta))

##
## Fixed-Effects Model (k = 2)
##
## Test for Heterogeneity:
## Q(df = 1) = 4.6272, p-val = 0.0315
##
## Model Results:
##
## estimate      se     zval     pval    ci.lb    ci.ub
##   2.1491   1.8558   1.1580   0.2469  -1.4883   5.7865
##
## ---
## Signif. codes:  0 '***' 0.001 '**' 0.01 '*' 0.05 '.' 0.1 ' ' 1
```

Days per week

```
#Study 1
depressiondis_yes_ds1_vgdaysweek <- subset(depressiondis_yes_ds1, select
= c(vgdaysweek)) #Subset 1i data
depressiondis_vgdaysweek_m1i <-
apply(depressiondis_yes_ds1_vgdaysweek, 2, mean) #Calculate m1i
depressiondis_vgdaysweek_sd1i <-
apply(depressiondis_yes_ds1_vgdaysweek, 2, sd) #Calculate sd1i
depressiondis_vgdaysweek_n1i <- colSums(!
is.na(depressiondis_yes_ds1_vgdaysweek)) #Calculate n1i
depressiondis_no_ds1_vgdaysweek <- subset(depressiondis_no_ds1, select =
c(vgdaysweek)) #Subset 2i data
depressiondis_vgdaysweek_m2i <- apply(depressiondis_no_ds1_vgdaysweek,
2, mean) #Calculate m2i
depressiondis_vgdaysweek_sd2i <- apply(depressiondis_no_ds1_vgdaysweek,
2, sd) #Calculate sd2i
depressiondis_vgdaysweek_n2i <- colSums(!
is.na(depressiondis_no_ds1_vgdaysweek)) #Calculate n2i
ds1depressiondis_meta <- data.frame(m1i = depressiondis_vgdaysweek_m1i,
sd1i = depressiondis_vgdaysweek_sd1i, n1i =
depressiondis_vgdaysweek_n1i, m2i = depressiondis_vgdaysweek_m2i, sd2i
= depressiondis_vgdaysweek_sd2i, n2i = depressiondis_vgdaysweek_n2i)
#MA dataframe
ds1depressiondis_meta <- escalc(measure="MD", m1i = m1i, sd1i = sd1i, n1i
= n1i, m2i = m2i, sd2i = sd2i, n2i = n2i, data=ds1depressiondis_meta)
```

```
#Calculate yi and vi

#Study 2
depressiondis_yes_ds2_vgdaysweek <- subset(depressiondis_yes_ds2, select
= c(vgdaysweek)) #Subset 1i data
depressiondis_vgdaysweek_m1i <-
apply(depressiondis_yes_ds2_vgdaysweek, 2, mean) #Calculate m1i
depressiondis_vgdaysweek_sd1i <-
apply(depressiondis_yes_ds2_vgdaysweek, 2, sd) #Calculate sd1i
depressiondis_vgdaysweek_n1i <- colSums(!
is.na(depressiondis_yes_ds2_vgdaysweek)) #Calculate n1i
depressiondis_no_ds2_vgdaysweek <- subset(depressiondis_no_ds2, select =
c(vgdaysweek)) #Subset 2i data
depressiondis_vgdaysweek_m2i <- apply(depressiondis_no_ds2_vgdaysweek,
2, mean) #Calculate m2i
depressiondis_vgdaysweek_sd2i <- apply(depressiondis_no_ds2_vgdaysweek,
2, sd) #Calculate sd2i
depressiondis_vgdaysweek_n2i <- colSums(!
is.na(depressiondis_no_ds2_vgdaysweek)) #Calculate n2i
ds2depressiondis_meta <- data.frame(m1i = depressiondis_vgdaysweek_m1i,
sd1i = depressiondis_vgdaysweek_sd1i, n1i =
depressiondis_vgdaysweek_n1i, m2i = depressiondis_vgdaysweek_m2i, sd2i
= depressiondis_vgdaysweek_sd2i, n2i = depressiondis_vgdaysweek_n2i)
#MA dataframe
ds2depressiondis_meta <- escalc(measure="MD", m1i = m1i, sd1i = sd1i, n1i
= n1i, m2i = m2i, sd2i = sd2i, n2i = n2i, data=ds2depressiondis_meta)
#Calculate yi and vi

#FE meta-analysis
(depressiondis_meta <- rbind(ds1depressiondis_meta,
ds2depressiondis_meta))

##        m1i     sd1i n1i      m2i     sd2i n2i     yi     vi
## 1 5.350000 2.221552 120 5.204082 2.254436  49 0.1459 0.1449
## 2 4.951923 2.128163 208 4.682540 2.340564  63 0.2694 0.1087

(depressiondis_vgdaysweek_meta <- rma(yi=yi, vi=vi, measure="MD",
method="FE", data=depressiondis_meta))

##
## Fixed-Effects Model (k = 2)
##
## Test for Heterogeneity:
## Q(df = 1) = 0.0601, p-val = 0.8063
##
## Model Results:
##
## estimate      se     zval     pval    ci.lb    ci.ub
##   0.2164   0.2492   0.8685   0.3851  -0.2720   0.7049
##
```

```
## ---
## Signif. codes:  0 '***' 0.001 '**' 0.01 '*' 0.05 '.' 0.1 ' ' 1
```

Depression care - year

Replicated Effect

Hours per day

Study 1

```
(vghrsday_depressionyear_ds1 <- t.test(vghrsday ~ mhyear, data=ds1depression))
```

```
##
##  Welch Two Sample t-test
##
## data:  vghrsday by mhyear
## t = 1.37, df = 166.67, p-value = 0.1725
## alternative hypothesis: true difference in means is not equal to 0
## 95 percent confidence interval:
##  -0.1816303  1.0051432
## sample estimates:
## mean in group 0 mean in group 1
##        2.806818        2.395062
```

Study 2

```
(vghrsday_depressionyear_ds2 <- t.test(vghrsday ~ mhyear, data=ds2depression))
```

```
##
##  Welch Two Sample t-test
##
## data:  vghrsday by mhyear
## t = -1.1833, df = 258.01, p-value = 0.2378
## alternative hypothesis: true difference in means is not equal to 0
## 95 percent confidence interval:
##  -1.313930  0.327538
## sample estimates:
## mean in group 0 mean in group 1
##        3.603774        4.096970
```

Hours per week

Study 1

```
(vghrsweek_depressionyear_ds1 <- t.test(vghrsweek ~ mhyear,
data=ds1depression))
```

```
##
##  Welch Two Sample t-test
##
## data:  vghrsweek by mhyear
## t = 1.378, df = 165.58, p-value = 0.1701
## alternative hypothesis: true difference in means is not equal to 0
## 95 percent confidence interval:
##  -1.34943  7.58512
## sample estimates:
## mean in group 0 mean in group 1
##        16.63636        13.51852
```

Study 2

```
(vghrsweek_depressionyear_ds2 <- t.test(vghrsweek ~ mhyear,
data=ds2depression))
```

```
##
##  Welch Two Sample t-test
##
## data:  vghrsweek by mhyear
## t = -0.92386, df = 252.8, p-value = 0.3564
## alternative hypothesis: true difference in means is not equal to 0
## 95 percent confidence interval:
##  -8.407068  3.038057
## sample estimates:
## mean in group 0 mean in group 1
##        19.50943        22.19394
```

Days per week

Study 1

```
(vgdaysweek_depressionyear_ds1 <- t.test(vgdaysweek ~ mhyear,
data=ds1depression))
```

```
##
##  Welch Two Sample t-test
##
## data:  vgdaysweek by mhyear
## t = -0.14358, df = 166.81, p-value = 0.886
## alternative hypothesis: true difference in means is not equal to 0
## 95 percent confidence interval:
```

```
##  -0.7263392  0.6278543
## sample estimates:
## mean in group 0 mean in group 1
##        5.284091        5.333333
```

Study 2

```
(vgdaysweek_depressionyear_ds2 <- t.test(vgdaysweek ~ mhyear,
data=ds2depression))

##
##  Welch Two Sample t-test
##
## data:  vgdaysweek by mhyear
## t = -0.4071, df = 210.3, p-value = 0.6843
## alternative hypothesis: true difference in means is not equal to 0
## 95 percent confidence interval:
##  -0.6577168  0.4325596
## sample estimates:
## mean in group 0 mean in group 1
##        4.820755        4.933333
```

MD

Hours per day

```
#Study 1
depressionyear_yes_ds1_vghrsday <- subset(depressionyear_yes_ds1, select
= c(vghrsday)) #Subset 1i data
depressionyear_vghrsday_m1i <- apply(depressionyear_yes_ds1_vghrsday, 2,
mean) #Calculate m1i
depressionyear_vghrsday_sd1i <- apply(depressionyear_yes_ds1_vghrsday,
2, sd) #Calculate sd1i
depressionyear_vghrsday_n1i <- colSums(!
is.na(depressionyear_yes_ds1_vghrsday)) #Calculate n1i
depressionyear_no_ds1_vghrsday <- subset(depressionyear_no_ds1, select =
c(vghrsday)) #Subset 2i data
depressionyear_vghrsday_m2i <- apply(depressionyear_no_ds1_vghrsday, 2,
mean) #Calculate m2i
depressionyear_vghrsday_sd2i <- apply(depressionyear_no_ds1_vghrsday, 2,
sd) #Calculate sd2i
depressionyear_vghrsday_n2i <- colSums(!
is.na(depressionyear_no_ds1_vghrsday)) #Calculate n2i
ds1depressionyear_meta <- data.frame(m1i = depressionyear_vghrsday_m1i,
sd1i = depressionyear_vghrsday_sd1i, n1i = depressionyear_vghrsday_n1i,
m2i = depressionyear_vghrsday_m2i, sd2i = depressionyear_vghrsday_sd2i,
n2i = depressionyear_vghrsday_n2i) #MA dataframe
ds1depressionyear_meta <- escalc(measure="MD", m1i = m1i, sd1i = sd1i,
```

```
n1i = n1i, m2i = m2i, sd2i = sd2i, n2i = n2i, data=ds1depressionyear_meta)
#Calculate yi and vi

#Study 2
depressionyear_yes_ds2_vghrsday <- subset(depressionyear_yes_ds2, select
= c(vghrsday)) #Subset 1i data
depressionyear_vghrsday_m1i <- apply(depressionyear_yes_ds2_vghrsday, 2,
mean) #Calculate m1i
depressionyear_vghrsday_sd1i <- apply(depressionyear_yes_ds2_vghrsday,
2, sd) #Calculate sd1i
depressionyear_vghrsday_n1i <- colSums(!
is.na(depressionyear_yes_ds2_vghrsday)) #Calculate n1i
depressionyear_no_ds2_vghrsday <- subset(depressionyear_no_ds2, select =
c(vghrsday)) #Subset 2i data
depressionyear_vghrsday_m2i <- apply(depressionyear_no_ds2_vghrsday, 2,
mean) #Calculate m2i
depressionyear_vghrsday_sd2i <- apply(depressionyear_no_ds2_vghrsday, 2,
sd) #Calculate sd2i
depressionyear_vghrsday_n2i <- colSums(!
is.na(depressionyear_no_ds2_vghrsday)) #Calculate n2i
ds2depressionyear_meta <- data.frame(m1i = depressionyear_vghrsday_m1i,
sd1i = depressionyear_vghrsday_sd1i, n1i = depressionyear_vghrsday_n1i,
m2i = depressionyear_vghrsday_m2i, sd2i = depressionyear_vghrsday_sd2i,
n2i = depressionyear_vghrsday_n2i) #MA dataframe
ds2depressionyear_meta <- escalc(measure="MD", m1i = m1i, sd1i = sd1i,
n1i = n1i, m2i = m2i, sd2i = sd2i, n2i = n2i, data=ds2depressionyear_meta)
#Calculate yi and vi

#FE meta-analysis
(depressionyear_meta <- rbind(ds1depressionyear_meta,
ds2depressionyear_meta))

##        m1i     sd1i n1i      m2i     sd2i n2i      yi     vi
## 1 2.395062 1.828107  81 2.806818 2.078118  88 -0.4118 0.0903
## 2 4.096970 3.819437 165 3.603774 3.006910 106  0.4932 0.1737

(depressionyear_vghrsday_meta <- rma(yi=yi, vi=vi, measure="MD",
method="FE", data=depressionyear_meta))

##
## Fixed-Effects Model (k = 2)
##
## Test for Heterogeneity:
## Q(df = 1) = 3.1015, p-val = 0.0782
##
## Model Results:
##
## estimate       se     zval     pval    ci.lb    ci.ub
##  -0.1022   0.2438  -0.4191   0.6752  -0.5800   0.3756
##
```

```
## ---
## Signif. codes:  0 '***' 0.001 '**' 0.01 '*' 0.05 '.' 0.1 ' ' 1
```

Hours per week

```
#Study 1
depressionyear_yes_ds1_vghrsweek <- subset(depressionyear_yes_ds1,
select = c(vghrsweek)) #Subset 1i data
depressionyear_vghrsweek_m1i <-
apply(depressionyear_yes_ds1_vghrsweek, 2, mean) #Calculate m1i
depressionyear_vghrsweek_sd1i <-
apply(depressionyear_yes_ds1_vghrsweek, 2, sd) #Calculate sd1i
depressionyear_vghrsweek_n1i <- colSums(!
is.na(depressionyear_yes_ds1_vghrsweek)) #Calculate n1i
depressionyear_no_ds1_vghrsweek <- subset(depressionyear_no_ds1, select
= c(vghrsweek)) #Subset 2i data
depressionyear_vghrsweek_m2i <- apply(depressionyear_no_ds1_vghrsweek,
2, mean) #Calculate m2i
depressionyear_vghrsweek_sd2i <- apply(depressionyear_no_ds1_vghrsweek,
2, sd) #Calculate sd2i
depressionyear_vghrsweek_n2i <- colSums(!
is.na(depressionyear_no_ds1_vghrsweek)) #Calculate n2i
ds1depressionyear_meta <- data.frame(m1i =
depressionyear_vghrsweek_m1i, sd1i = depressionyear_vghrsweek_sd1i, n1i
= depressionyear_vghrsweek_n1i, m2i = depressionyear_vghrsweek_m2i,
sd2i = depressionyear_vghrsweek_sd2i, n2i =
depressionyear_vghrsweek_n2i) #MA dataframe
ds1depressionyear_meta <- escalc(measure="MD", m1i = m1i, sd1i = sd1i,
n1i = n1i, m2i = m2i, sd2i = sd2i, n2i = n2i, data=ds1depressionyear_meta)
#Calculate yi and vi

#Study 2
depressionyear_yes_ds2_vghrsweek <- subset(depressionyear_yes_ds2,
select = c(vghrsweek)) #Subset 1i data
depressionyear_vghrsweek_m1i <-
apply(depressionyear_yes_ds2_vghrsweek, 2, mean) #Calculate m1i
depressionyear_vghrsweek_sd1i <-
apply(depressionyear_yes_ds2_vghrsweek, 2, sd) #Calculate sd1i
depressionyear_vghrsweek_n1i <- colSums(!
is.na(depressionyear_yes_ds2_vghrsweek)) #Calculate n1i
depressionyear_no_ds2_vghrsweek <- subset(depressionyear_no_ds2, select
= c(vghrsweek)) #Subset 2i data
depressionyear_vghrsweek_m2i <- apply(depressionyear_no_ds2_vghrsweek,
2, mean) #Calculate m2i
depressionyear_vghrsweek_sd2i <- apply(depressionyear_no_ds2_vghrsweek,
2, sd) #Calculate sd2i
depressionyear_vghrsweek_n2i <- colSums(!
is.na(depressionyear_no_ds2_vghrsweek)) #Calculate n2i
ds2depressionyear_meta <- data.frame(m1i =
```

```
depressionyear_vghrsweek_m1i, sd1i = depressionyear_vghrsweek_sd1i, n1i
= depressionyear_vghrsweek_n1i, m2i = depressionyear_vghrsweek_m2i,
sd2i = depressionyear_vghrsweek_sd2i, n2i =
depressionyear_vghrsweek_n2i) #MA dataframe
ds2depressionyear_meta <- escalc(measure="MD", m1i = m1i, sd1i = sd1i,
n1i = n1i, m2i = m2i, sd2i = sd2i, n2i = n2i, data=ds2depressionyear_meta)
#Calculate yi and vi

#FE meta-analysis
(depressionyear_meta <- rbind(ds1depressionyear_meta,
ds2depressionyear_meta))
```

```
##        m1i     sd1i n1i      m2i     sd2i n2i      yi     vi
## 1 13.51852 13.39600  81 16.63636 15.98576  88 -3.1178 5.1194
## 2 22.19394 26.02482 165 19.50943 21.44506 106  2.6845 8.4434
```

```
(depressionyear_vghrsweek_meta <- rma(yi=yi, vi=vi, measure="MD",
method="FE", data=depressionyear_meta))
```

```
##
## Fixed-Effects Model (k = 2)
##
## Test for Heterogeneity:
## Q(df = 1) = 2.4823, p-val = 0.1151
##
## Model Results:
##
## estimate      se     zval     pval    ci.lb    ci.ub
##  -0.9277   1.7852  -0.5197   0.6033  -4.4267   2.5713
##
## ---
## Signif. codes:  0 '***' 0.001 '**' 0.01 '*' 0.05 '.' 0.1 ' ' 1
```

Days per week

```
#Study 1
depressionyear_yes_ds1_vgdaysweek <- subset(depressionyear_yes_ds1,
select = c(vgdaysweek)) #Subset 1i data
depressionyear_vgdaysweek_m1i <-
apply(depressionyear_yes_ds1_vgdaysweek, 2, mean) #Calculate m1i
depressionyear_vgdaysweek_sd1i <-
apply(depressionyear_yes_ds1_vgdaysweek, 2, sd) #Calculate sd1i
depressionyear_vgdaysweek_n1i <- colSums(!
is.na(depressionyear_yes_ds1_vgdaysweek)) #Calculate n1i
depressionyear_no_ds1_vgdaysweek <- subset(depressionyear_no_ds1,
select = c(vgdaysweek)) #Subset 2i data
depressionyear_vgdaysweek_m2i <-
apply(depressionyear_no_ds1_vgdaysweek, 2, mean) #Calculate m2i
depressionyear_vgdaysweek_sd2i <-
apply(depressionyear_no_ds1_vgdaysweek, 2, sd) #Calculate sd2i
```

```
depressionyear_vgdaysweek_n2i <- colSums(!
is.na(depressionyear_no_ds1_vgdaysweek)) #Calculate n2i
ds1depressionyear_meta <- data.frame(m1i =
depressionyear_vgdaysweek_m1i, sd1i = depressionyear_vgdaysweek_sd1i,
n1i = depressionyear_vgdaysweek_n1i, m2i =
depressionyear_vgdaysweek_m2i, sd2i = depressionyear_vgdaysweek_sd2i,
n2i = depressionyear_vgdaysweek_n2i) #MA dataframe
ds1depressionyear_meta <- escalc(measure="MD", m1i = m1i, sd1i = sd1i,
n1i = n1i, m2i = m2i, sd2i = sd2i, n2i = n2i, data=ds1depressionyear_meta)
#Calculate yi and vi

#Study 2
depressionyear_yes_ds2_vgdaysweek <- subset(depressionyear_yes_ds2,
select = c(vgdaysweek)) #Subset 1i data
depressionyear_vgdaysweek_m1i <-
apply(depressionyear_yes_ds2_vgdaysweek, 2, mean) #Calculate m1i
depressionyear_vgdaysweek_sd1i <-
apply(depressionyear_yes_ds2_vgdaysweek, 2, sd) #Calculate sd1i
depressionyear_vgdaysweek_n1i <- colSums(!
is.na(depressionyear_yes_ds2_vgdaysweek)) #Calculate n1i
depressionyear_no_ds2_vgdaysweek <- subset(depressionyear_no_ds2,
select = c(vgdaysweek)) #Subset 2i data
depressionyear_vgdaysweek_m2i <-
apply(depressionyear_no_ds2_vgdaysweek, 2, mean) #Calculate m2i
depressionyear_vgdaysweek_sd2i <-
apply(depressionyear_no_ds2_vgdaysweek, 2, sd) #Calculate sd2i
depressionyear_vgdaysweek_n2i <- colSums(!
is.na(depressionyear_no_ds2_vgdaysweek)) #Calculate n2i
ds2depressionyear_meta <- data.frame(m1i =
depressionyear_vgdaysweek_m1i, sd1i = depressionyear_vgdaysweek_sd1i,
n1i = depressionyear_vgdaysweek_n1i, m2i =
depressionyear_vgdaysweek_m2i, sd2i = depressionyear_vgdaysweek_sd2i,
n2i = depressionyear_vgdaysweek_n2i) #MA dataframe
ds2depressionyear_meta <- escalc(measure="MD", m1i = m1i, sd1i = sd1i,
n1i = n1i, m2i = m2i, sd2i = sd2i, n2i = n2i, data=ds2depressionyear_meta)
#Calculate yi and vi

#FE meta-analysis
(depressionyear_meta <- rbind(ds1depressionyear_meta,
ds2depressionyear_meta))

##        m1i     sd1i n1i      m2i     sd2i n2i     yi     vi
## 1 5.333333 2.173707  81 5.284091 2.284122  88 0.0492 0.1176
## 2 4.933333 2.107285 165 4.820755 2.292003 106 0.1126 0.0765

(depressionyear_vgdaysweek_meta <- rma(yi=yi, vi=vi, measure="MD",
method="FE", data=depressionyear_meta))

##
## Fixed-Effects Model (k = 2)
##
```

```
## Test for Heterogeneity:
## Q(df = 1) = 0.0207, p-val = 0.8857
##
## Model Results:
##
## estimate      se     zval     pval    ci.lb    ci.ub
##   0.0876   0.2153   0.4070   0.6840  -0.3343   0.5096
##
## ---
## Signif. codes:  0 '***' 0.001 '**' 0.01 '*' 0.05 '.' 0.1 ' ' 1
```

Depression care - month

Replicated Effect

Hours per day

Study 1

```
(vghrsday_depressionmonth_ds1 <- t.test(vghrsday ~ mhmonth,
data=ds1depression))
```

```
##
##  Welch Two Sample t-test
##
## data:  vghrsday by mhmonth
## t = -1.4666, df = 38.807, p-value = 0.1505
## alternative hypothesis: true difference in means is not equal to 0
## 95 percent confidence interval:
##  -1.5155007  0.2416398
## sample estimates:
## mean in group 0 mean in group 1
##        2.496403        3.133333
```

Study 2

```
(vghrsday_depressionmonth_ds2 <- t.test(vghrsday ~ mhmonth,
data=ds2depression))
```

```
##
##  Welch Two Sample t-test
##
## data:  vghrsday by mhmonth
## t = 0.70652, df = 154.02, p-value = 0.4809
## alternative hypothesis: true difference in means is not equal to 0
## 95 percent confidence interval:
##  -0.5548928  1.1727801
```

```
## sample estimates:
## mean in group 0 mean in group 1
##        3.985000        3.676056
```

Hours per week

Study 1

```
(vghrsweek_depressionmonth_ds1 <- t.test(vghrsweek ~ mhmonth, data=ds1depression))
```

```
##
##  Welch Two Sample t-test
##
## data:  vghrsweek by mhmonth
## t = -1.2268, df = 39.024, p-value = 0.2272
## alternative hypothesis: true difference in means is not equal to 0
## 95 percent confidence interval:
##  -10.59930   2.59594
## sample estimates:
## mean in group 0 mean in group 1
##        14.43165        18.43333
```

Study 2

```
(vghrsweek_depressionmonth_ds2 <- t.test(vghrsweek ~ mhmonth, data=ds2depression))
```

```
##
##  Welch Two Sample t-test
##
## data:  vghrsweek by mhmonth
## t = 1.0685, df = 155.14, p-value = 0.287
## alternative hypothesis: true difference in means is not equal to 0
## 95 percent confidence interval:
##  -2.724875  9.145579
## sample estimates:
## mean in group 0 mean in group 1
##        21.98500        18.77465
```

Days per week

Study 1

```
(vgdaysweek_depressionmonth_ds1 <- t.test(vgdaysweek ~ mhmonth, data=ds1depression))
```

```
##
##  Welch Two Sample t-test
##
## data:  vgdaysweek by mhmonth
## t = -0.71491, df = 43.295, p-value = 0.4785
## alternative hypothesis: true difference in means is not equal to 0
## 95 percent confidence interval:
##  -1.2029034  0.5731672
## sample estimates:
## mean in group 0 mean in group 1
##        5.251799        5.566667
```

Study 2

```
(vgdaysweek_depressionmonth_ds2 <- t.test(vgdaysweek ~ mhmonth,
data=ds2depression))
```

```
##
##  Welch Two Sample t-test
##
## data:  vgdaysweek by mhmonth
## t = 0.074803, df = 131.7, p-value = 0.9405
## alternative hypothesis: true difference in means is not equal to 0
## 95 percent confidence interval:
##  -0.5536870  0.5972082
## sample estimates:
## mean in group 0 mean in group 1
##        4.895000        4.873239
```

MD

Hours per day

```
#Study 1
depressionmonth_yes_ds1_vghrsday <- subset(depressionmonth_yes_ds1,
select = c(vghrsday)) #Subset 1i data
depressionmonth_vghrsday_m1i <-
apply(depressionmonth_yes_ds1_vghrsday, 2, mean) #Calculate m1i
depressionmonth_vghrsday_sd1i <-
apply(depressionmonth_yes_ds1_vghrsday, 2, sd) #Calculate sd1i
depressionmonth_vghrsday_n1i <- colSums(!
is.na(depressionmonth_yes_ds1_vghrsday)) #Calculate n1i
depressionmonth_no_ds1_vghrsday <- subset(depressionmonth_no_ds1,
select = c(vghrsday)) #Subset 2i data
depressionmonth_vghrsday_m2i <-
apply(depressionmonth_no_ds1_vghrsday, 2, mean) #Calculate m2i
depressionmonth_vghrsday_sd2i <-
apply(depressionmonth_no_ds1_vghrsday, 2, sd) #Calculate sd2i
```

```
depressionmonth_vghrsday_n2i <- colSums(!
is.na(depressionmonth_no_ds1_vghrsday)) #Calculate n2i
ds1depressionmonth_meta <- data.frame(m1i =
depressionmonth_vghrsday_m1i, sd1i = depressionmonth_vghrsday_sd1i, n1i
= depressionmonth_vghrsday_n1i, m2i = depressionmonth_vghrsday_m2i,
sd2i = depressionmonth_vghrsday_sd2i, n2i =
depressionmonth_vghrsday_n2i) #MA dataframe
ds1depressionmonth_meta <- escalc(measure="MD", m1i = m1i, sd1i = sd1i,
n1i = n1i, m2i = m2i, sd2i = sd2i, n2i = n2i,
data=ds1depressionmonth_meta) #Calculate yi and vi

#Study 2
depressionmonth_yes_ds2_vghrsday <- subset(depressionmonth_yes_ds2,
select = c(vghrsday)) #Subset 1i data
depressionmonth_vghrsday_m1i <-
apply(depressionmonth_yes_ds2_vghrsday, 2, mean) #Calculate m1i
depressionmonth_vghrsday_sd1i <-
apply(depressionmonth_yes_ds2_vghrsday, 2, sd) #Calculate sd1i
depressionmonth_vghrsday_n1i <- colSums(!
is.na(depressionmonth_yes_ds2_vghrsday)) #Calculate n1i
depressionmonth_no_ds2_vghrsday <- subset(depressionmonth_no_ds2,
select = c(vghrsday)) #Subset 2i data
depressionmonth_vghrsday_m2i <-
apply(depressionmonth_no_ds2_vghrsday, 2, mean) #Calculate m2i
depressionmonth_vghrsday_sd2i <-
apply(depressionmonth_no_ds2_vghrsday, 2, sd) #Calculate sd2i
depressionmonth_vghrsday_n2i <- colSums(!
is.na(depressionmonth_no_ds2_vghrsday)) #Calculate n2i
ds2depressionmonth_meta <- data.frame(m1i =
depressionmonth_vghrsday_m1i, sd1i = depressionmonth_vghrsday_sd1i, n1i
= depressionmonth_vghrsday_n1i, m2i = depressionmonth_vghrsday_m2i,
sd2i = depressionmonth_vghrsday_sd2i, n2i =
depressionmonth_vghrsday_n2i) #MA dataframe
ds2depressionmonth_meta <- escalc(measure="MD", m1i = m1i, sd1i = sd1i,
n1i = n1i, m2i = m2i, sd2i = sd2i, n2i = n2i,
data=ds2depressionmonth_meta) #Calculate yi and vi

#FE meta-analysis
(depressionmonth_meta <- rbind(ds1depressionmonth_meta,
ds2depressionmonth_meta))

##        m1i     sd1i n1i      m2i     sd2i n2i      yi     vi
## 1 3.133333 2.208656  30 2.496403 1.901083 139  0.6369 0.1886
## 2 3.676056 2.946061  71 3.985000 3.713993 200 -0.3089 0.1912

(depressionmonth_vghrsday_meta <- rma(yi=yi, vi=vi, measure="MD",
method="FE", data=depressionmonth_meta))

##
## Fixed-Effects Model (k = 2)
##
```

```
## Test for Heterogeneity:
## Q(df = 1) = 2.3555, p-val = 0.1248
##
## Model Results:
##
## estimate       se     zval     pval    ci.lb    ci.ub
##   0.1672   0.3081   0.5427   0.5873  -0.4367   0.7712
##
## ---
## Signif. codes:  0 '***' 0.001 '**' 0.01 '*' 0.05 '.' 0.1 ' ' 1
```

Hours per week

```
#Study 1
depressionmonth_yes_ds1_vghrsweek <- subset(depressionmonth_yes_ds1,
select = c(vghrsweek)) #Subset 1i data
depressionmonth_vghrsweek_m1i <-
apply(depressionmonth_yes_ds1_vghrsweek, 2, mean) #Calculate m1i
depressionmonth_vghrsweek_sd1i <-
apply(depressionmonth_yes_ds1_vghrsweek, 2, sd) #Calculate sd1i
depressionmonth_vghrsweek_n1i <- colSums(!
is.na(depressionmonth_yes_ds1_vghrsweek)) #Calculate n1i
depressionmonth_no_ds1_vghrsweek <- subset(depressionmonth_no_ds1,
select = c(vghrsweek)) #Subset 2i data
depressionmonth_vghrsweek_m2i <-
apply(depressionmonth_no_ds1_vghrsweek, 2, mean) #Calculate m2i
depressionmonth_vghrsweek_sd2i <-
apply(depressionmonth_no_ds1_vghrsweek, 2, sd) #Calculate sd2i
depressionmonth_vghrsweek_n2i <- colSums(!
is.na(depressionmonth_no_ds1_vghrsweek)) #Calculate n2i
ds1depressionmonth_meta <- data.frame(m1i =
depressionmonth_vghrsweek_m1i, sd1i = depressionmonth_vghrsweek_sd1i,
n1i = depressionmonth_vghrsweek_n1i, m2i =
depressionmonth_vghrsweek_m2i, sd2i = depressionmonth_vghrsweek_sd2i,
n2i = depressionmonth_vghrsweek_n2i) #MA dataframe
ds1depressionmonth_meta <- escalc(measure="MD", m1i = m1i, sd1i = sd1i,
n1i = n1i, m2i = m2i, sd2i = sd2i, n2i = n2i,
data=ds1depressionmonth_meta) #Calculate yi and vi

#Study 2
depressionmonth_yes_ds2_vghrsweek <- subset(depressionmonth_yes_ds2,
select = c(vghrsweek)) #Subset 1i data
depressionmonth_vghrsweek_m1i <-
apply(depressionmonth_yes_ds2_vghrsweek, 2, mean) #Calculate m1i
depressionmonth_vghrsweek_sd1i <-
apply(depressionmonth_yes_ds2_vghrsweek, 2, sd) #Calculate sd1i
depressionmonth_vghrsweek_n1i <- colSums(!
is.na(depressionmonth_yes_ds2_vghrsweek)) #Calculate n1i
depressionmonth_no_ds2_vghrsweek <- subset(depressionmonth_no_ds2,
```

```
select = c(vghrsweek)) #Subset 2i data
depressionmonth_vghrsweek_m2i <-
apply(depressionmonth_no_ds2_vghrsweek, 2, mean) #Calculate m2i
depressionmonth_vghrsweek_sd2i <-
apply(depressionmonth_no_ds2_vghrsweek, 2, sd) #Calculate sd2i
depressionmonth_vghrsweek_n2i <- colSums(!
is.na(depressionmonth_no_ds2_vghrsweek)) #Calculate n2i
ds2depressionmonth_meta <- data.frame(m1i =
depressionmonth_vghrsweek_m1i, sd1i = depressionmonth_vghrsweek_sd1i,
n1i = depressionmonth_vghrsweek_n1i, m2i =
depressionmonth_vghrsweek_m2i, sd2i = depressionmonth_vghrsweek_sd2i,
n2i = depressionmonth_vghrsweek_n2i) #MA dataframe
ds2depressionmonth_meta <- escalc(measure="MD", m1i = m1i, sd1i = sd1i,
n1i = n1i, m2i = m2i, sd2i = sd2i, n2i = n2i,
data=ds2depressionmonth_meta) #Calculate yi and vi

#FE meta-analysis
(depressionmonth_meta <- rbind(ds1depressionmonth_meta,
ds2depressionmonth_meta))

##        m1i      sd1i n1i      m2i      sd2i n2i      yi      vi
## 1 18.43333 16.56478  30 14.43165 14.40765 139  4.0017 10.6398
## 2 18.77465 20.19208  71 21.98500 25.63259 200 -3.2104  9.0277

(depressionmonth_vghrsweek_meta <- rma(yi=yi, vi=vi, measure="MD",
method="FE", data=depressionmonth_meta))

##
## Fixed-Effects Model (k = 2)
##
## Test for Heterogeneity:
## Q(df = 1) = 2.6446, p-val = 0.1039
##
## Model Results:
##
## estimate      se     zval     pval    ci.lb    ci.ub
##   0.1001   2.2099   0.0453   0.9639  -4.2313   4.4315
##
## ---
## Signif. codes:  0 '***' 0.001 '**' 0.01 '*' 0.05 '.' 0.1 ' ' 1
```

Days per week

```
#Study 1
depressionmonth_yes_ds1_vgdaysweek <- subset(depressionmonth_yes_ds1,
select = c(vgdaysweek)) #Subset 1i data
depressionmonth_vgdaysweek_m1i <-
apply(depressionmonth_yes_ds1_vgdaysweek, 2, mean) #Calculate m1i
depressionmonth_vgdaysweek_sd1i <-
apply(depressionmonth_yes_ds1_vgdaysweek, 2, sd) #Calculate sd1i
```

```
depressionmonth_vgdaysweek_n1i <- colSums(!
is.na(depressionmonth_yes_ds1_vgdaysweek)) #Calculate n1i
depressionmonth_no_ds1_vgdaysweek <- subset(depressionmonth_no_ds1,
select = c(vgdaysweek)) #Subset 2i data
depressionmonth_vgdaysweek_m2i <-
apply(depressionmonth_no_ds1_vgdaysweek, 2, mean) #Calculate m2i
depressionmonth_vgdaysweek_sd2i <-
apply(depressionmonth_no_ds1_vgdaysweek, 2, sd) #Calculate sd2i
depressionmonth_vgdaysweek_n2i <- colSums(!
is.na(depressionmonth_no_ds1_vgdaysweek)) #Calculate n2i
ds1depressionmonth_meta <- data.frame(m1i =
depressionmonth_vgdaysweek_m1i, sd1i =
depressionmonth_vgdaysweek_sd1i, n1i =
depressionmonth_vgdaysweek_n1i, m2i =
depressionmonth_vgdaysweek_m2i, sd2i =
depressionmonth_vgdaysweek_sd2i, n2i =
depressionmonth_vgdaysweek_n2i) #MA dataframe
ds1depressionmonth_meta <- escalc(measure="MD", m1i = m1i, sd1i = sd1i,
n1i = n1i, m2i = m2i, sd2i = sd2i, n2i = n2i,
data=ds1depressionmonth_meta) #Calculate yi and vi

#Study 2
depressionmonth_yes_ds2_vgdaysweek <- subset(depressionmonth_yes_ds2,
select = c(vgdaysweek)) #Subset 1i data
depressionmonth_vgdaysweek_m1i <-
apply(depressionmonth_yes_ds2_vgdaysweek, 2, mean) #Calculate m1i
depressionmonth_vgdaysweek_sd1i <-
apply(depressionmonth_yes_ds2_vgdaysweek, 2, sd) #Calculate sd1i
depressionmonth_vgdaysweek_n1i <- colSums(!
is.na(depressionmonth_yes_ds2_vgdaysweek)) #Calculate n1i
depressionmonth_no_ds2_vgdaysweek <- subset(depressionmonth_no_ds2,
select = c(vgdaysweek)) #Subset 2i data
depressionmonth_vgdaysweek_m2i <-
apply(depressionmonth_no_ds2_vgdaysweek, 2, mean) #Calculate m2i
depressionmonth_vgdaysweek_sd2i <-
apply(depressionmonth_no_ds2_vgdaysweek, 2, sd) #Calculate sd2i
depressionmonth_vgdaysweek_n2i <- colSums(!
is.na(depressionmonth_no_ds2_vgdaysweek)) #Calculate n2i
ds2depressionmonth_meta <- data.frame(m1i =
depressionmonth_vgdaysweek_m1i, sd1i =
depressionmonth_vgdaysweek_sd1i, n1i =
depressionmonth_vgdaysweek_n1i, m2i =
depressionmonth_vgdaysweek_m2i, sd2i =
depressionmonth_vgdaysweek_sd2i, n2i =
depressionmonth_vgdaysweek_n2i) #MA dataframe
ds2depressionmonth_meta <- escalc(measure="MD", m1i = m1i, sd1i = sd1i,
n1i = n1i, m2i = m2i, sd2i = sd2i, n2i = n2i,
data=ds2depressionmonth_meta) #Calculate yi and vi

#FE meta-analysis
```

```
(depressionmonth_meta <- rbind(ds1depressionmonth_meta,
ds2depressionmonth_meta))
```

```
##        m1i     sd1i n1i      m2i     sd2i n2i      yi     vi
## 1 5.566667 2.176415  30 5.251799 2.239609 139  0.3149 0.1940
## 2 4.873239 2.062797  71 4.895000 2.222311 200 -0.0218 0.0846
```

```
(depressionmonth_vgdaysweek_meta <- rma(yi=yi, vi=vi, measure="MD",
method="FE", data=depressionmonth_meta))
```

```
##
## Fixed-Effects Model (k = 2)
##
## Test for Heterogeneity:
## Q(df = 1) = 0.4067, p-val = 0.5236
##
## Model Results:
##
## estimate      se     zval     pval    ci.lb    ci.ub
##   0.0805   0.2427   0.3316   0.7402  -0.3953   0.5562
##
## ---
## Signif. codes:  0 '***' 0.001 '**' 0.01 '*' 0.05 '.' 0.1 ' ' 1
```

Any SUD - discharge

Replicated Effect

Hours per day

Study 1

```
(vghrsday_anysudscreendis_ds1 <- t.test(vghrsday ~ alcsuddis,
data=ds1anysudscreen))
```

```
##
##  Welch Two Sample t-test
##
## data:  vghrsday by alcsuddis
## t = -1.3424, df = 58.109, p-value = 0.1847
## alternative hypothesis: true difference in means is not equal to 0
## 95 percent confidence interval:
##  -1.0248867  0.2020385
## sample estimates:
## mean in group 0 mean in group 1
##        2.077465        2.488889
```

Study 2

```
(vghrsday_anysudscreendis_ds2 <- t.test(vghrsday ~ alcsuddis,
data=ds2anysudscreen))

##
##  Welch Two Sample t-test
##
## data:  vghrsday by alcsuddis
## t = 0.98905, df = 132.12, p-value = 0.3244
## alternative hypothesis: true difference in means is not equal to 0
## 95 percent confidence interval:
##  -0.371894  1.115702
## sample estimates:
## mean in group 0 mean in group 1
##        3.660793        3.288889
```

Hours per week

Study 1

```
(vghrsweek_anysudscreendis_ds1 <- t.test(vghrsweek ~ alcsuddis,
data=ds1anysudscreen))

##
##  Welch Two Sample t-test
##
## data:  vghrsweek by alcsuddis
## t = -2.1523, df = 59.465, p-value = 0.03544
## alternative hypothesis: true difference in means is not equal to 0
## 95 percent confidence interval:
##  -9.2333783 -0.3372008
## sample estimates:
## mean in group 0 mean in group 1
##        10.85915        15.64444
```

Study 2

```
(vghrsweek_anysudscreendis_ds2 <- t.test(vghrsweek ~ alcsuddis,
data=ds2anysudscreen))

##
##  Welch Two Sample t-test
##
## data:  vghrsweek by alcsuddis
## t = 0.94109, df = 143.29, p-value = 0.3482
## alternative hypothesis: true difference in means is not equal to 0
## 95 percent confidence interval:
##  -2.518879  7.097048
## sample estimates:
```

```
## mean in group 0 mean in group 1
##        18.97797        16.68889
```

Days per week

Study 1

```
(vgdaysweek_anysudscreendis_ds1 <- t.test(vgdaysweek ~ alcsuddis,
data=ds1anysudscreen))

##
##  Welch Two Sample t-test
##
## data:  vgdaysweek by alcsuddis
## t = -3.4102, df = 93.98, p-value = 0.0009583
## alternative hypothesis: true difference in means is not equal to 0
## 95 percent confidence interval:
##  -1.7570534 -0.4639169
## sample estimates:
## mean in group 0 mean in group 1
##        4.845070        5.955556
```

Study 2

```
(vgdaysweek_anysudscreendis_ds2 <- t.test(vgdaysweek ~ alcsuddis,
data=ds2anysudscreen))

##
##  Welch Two Sample t-test
##
## data:  vgdaysweek by alcsuddis
## t = -0.95005, df = 65.057, p-value = 0.3456
## alternative hypothesis: true difference in means is not equal to 0
## 95 percent confidence interval:
##  -1.0249259  0.3641329
## sample estimates:
## mean in group 0 mean in group 1
##        4.669604        5.000000
```

MD

Hours per day

```
#Study 1
anysudscreendis_yes_ds1_vghrsday <- subset(anysudscreendis_yes_ds1,
select = c(vghrsday)) #Subset 1i data
anysudscreendis_vghrsday_m1i <- apply(anysudscreendis_yes_ds1_vghrsday,
2, mean) #Calculate m1i
```

```
anysudscreendis_vghrsday_sd1i <-
apply(anysudscreendis_yes_ds1_vghrsday, 2, sd) #Calculate sd1i
anysudscreendis_vghrsday_n1i <- colSums(!
is.na(anysudscreendis_yes_ds1_vghrsday)) #Calculate n1i
anysudscreendis_no_ds1_vghrsday <- subset(anysudscreendis_no_ds1, select
= c(vghrsday)) #Subset 2i data
anysudscreendis_vghrsday_m2i <- apply(anysudscreendis_no_ds1_vghrsday,
2, mean) #Calculate m2i
anysudscreendis_vghrsday_sd2i <- apply(anysudscreendis_no_ds1_vghrsday,
2, sd) #Calculate sd2i
anysudscreendis_vghrsday_n2i <- colSums(!
is.na(anysudscreendis_no_ds1_vghrsday)) #Calculate n2i
ds1anysudscreendis_meta <- data.frame(m1i =
anysudscreendis_vghrsday_m1i, sd1i = anysudscreendis_vghrsday_sd1i, n1i
= anysudscreendis_vghrsday_n1i, m2i = anysudscreendis_vghrsday_m2i,
sd2i = anysudscreendis_vghrsday_sd2i, n2i = anysudscreendis_vghrsday_n2i)
#MA dataframe
ds1anysudscreendis_meta <- escalc(measure="MD", m1i = m1i, sd1i = sd1i,
n1i = n1i, m2i = m2i, sd2i = sd2i, n2i = n2i,
data=ds1anysudscreendis_meta) #Calculate yi and vi

#Study 2
anysudscreendis_yes_ds2_vghrsday <- subset(anysudscreendis_yes_ds2,
select = c(vghrsday)) #Subset 1i data
anysudscreendis_vghrsday_m1i <- apply(anysudscreendis_yes_ds2_vghrsday,
2, mean) #Calculate m1i
anysudscreendis_vghrsday_sd1i <-
apply(anysudscreendis_yes_ds2_vghrsday, 2, sd) #Calculate sd1i
anysudscreendis_vghrsday_n1i <- colSums(!
is.na(anysudscreendis_yes_ds2_vghrsday)) #Calculate n1i
anysudscreendis_no_ds2_vghrsday <- subset(anysudscreendis_no_ds2, select
= c(vghrsday)) #Subset 2i data
anysudscreendis_vghrsday_m2i <- apply(anysudscreendis_no_ds2_vghrsday,
2, mean) #Calculate m2i
anysudscreendis_vghrsday_sd2i <- apply(anysudscreendis_no_ds2_vghrsday,
2, sd) #Calculate sd2i
anysudscreendis_vghrsday_n2i <- colSums(!
is.na(anysudscreendis_no_ds2_vghrsday)) #Calculate n2i
ds2anysudscreendis_meta <- data.frame(m1i =
anysudscreendis_vghrsday_m1i, sd1i = anysudscreendis_vghrsday_sd1i, n1i
= anysudscreendis_vghrsday_n1i, m2i = anysudscreendis_vghrsday_m2i,
sd2i = anysudscreendis_vghrsday_sd2i, n2i = anysudscreendis_vghrsday_n2i)
#MA dataframe
ds2anysudscreendis_meta <- escalc(measure="MD", m1i = m1i, sd1i = sd1i,
n1i = n1i, m2i = m2i, sd2i = sd2i, n2i = n2i,
data=ds2anysudscreendis_meta) #Calculate yi and vi

#FE meta-analysis
(anysudscreendis_meta <- rbind(ds1anysudscreendis_meta,
ds2anysudscreendis_meta))
```

```
##        m1i     sd1i n1i      m2i     sd2i n2i      yi     vi
## 1 2.488889 1.914327  45 2.077465 1.331953 142  0.4114 0.0939
## 2 3.288889 1.854016  45 3.660793 3.841368 227 -0.3719 0.1414
```

```
(anysudscreendis_vghrsday_meta <- rma(yi=yi, vi=vi, measure="MD",
method="FE", data=anysudscreendis_meta))
```

```
##
## Fixed-Effects Model (k = 2)
##
## Test for Heterogeneity:
## Q(df = 1) = 2.6075, p-val = 0.1064
##
## Model Results:
##
## estimate       se     zval     pval    ci.lb    ci.ub
##   0.0988   0.2376   0.4157   0.6776  -0.3669   0.5644
##
## ---
## Signif. codes:  0 '***' 0.001 '**' 0.01 '*' 0.05 '.' 0.1 ' ' 1
```

Hours per week

```
#Study 1
anysudscreendis_yes_ds1_vghrsweek <- subset(anysudscreendis_yes_ds1,
select = c(vghrsweek)) #Subset 1i data
anysudscreendis_vghrsweek_m1i <-
apply(anysudscreendis_yes_ds1_vghrsweek, 2, mean) #Calculate m1i
anysudscreendis_vghrsweek_sd1i <-
apply(anysudscreendis_yes_ds1_vghrsweek, 2, sd) #Calculate sd1i
anysudscreendis_vghrsweek_n1i <- colSums(!
is.na(anysudscreendis_yes_ds1_vghrsweek)) #Calculate n1i
anysudscreendis_no_ds1_vghrsweek <- subset(anysudscreendis_no_ds1,
select = c(vghrsweek)) #Subset 2i data
anysudscreendis_vghrsweek_m2i <-
apply(anysudscreendis_no_ds1_vghrsweek, 2, mean) #Calculate m2i
anysudscreendis_vghrsweek_sd2i <-
apply(anysudscreendis_no_ds1_vghrsweek, 2, sd) #Calculate sd2i
anysudscreendis_vghrsweek_n2i <- colSums(!
is.na(anysudscreendis_no_ds1_vghrsweek)) #Calculate n2i
ds1anysudscreendis_meta <- data.frame(m1i =
anysudscreendis_vghrsweek_m1i, sd1i = anysudscreendis_vghrsweek_sd1i,
n1i = anysudscreendis_vghrsweek_n1i, m2i =
anysudscreendis_vghrsweek_m2i, sd2i = anysudscreendis_vghrsweek_sd2i,
n2i = anysudscreendis_vghrsweek_n2i) #MA dataframe
ds1anysudscreendis_meta <- escalc(measure="MD", m1i = m1i, sd1i = sd1i,
n1i = n1i, m2i = m2i, sd2i = sd2i, n2i = n2i,
data=ds1anysudscreendis_meta) #Calculate yi and vi
```

```r
#Study 2
anysudscreendis_yes_ds2_vghrsweek <- subset(anysudscreendis_yes_ds2,
select = c(vghrsweek)) #Subset 1i data
anysudscreendis_vghrsweek_m1i <-
apply(anysudscreendis_yes_ds2_vghrsweek, 2, mean) #Calculate m1i
anysudscreendis_vghrsweek_sd1i <-
apply(anysudscreendis_yes_ds2_vghrsweek, 2, sd) #Calculate sd1i
anysudscreendis_vghrsweek_n1i <- colSums(!
is.na(anysudscreendis_yes_ds2_vghrsweek)) #Calculate n1i
anysudscreendis_no_ds2_vghrsweek <- subset(anysudscreendis_no_ds2,
select = c(vghrsweek)) #Subset 2i data
anysudscreendis_vghrsweek_m2i <-
apply(anysudscreendis_no_ds2_vghrsweek, 2, mean) #Calculate m2i
anysudscreendis_vghrsweek_sd2i <-
apply(anysudscreendis_no_ds2_vghrsweek, 2, sd) #Calculate sd2i
anysudscreendis_vghrsweek_n2i <- colSums(!
is.na(anysudscreendis_no_ds2_vghrsweek)) #Calculate n2i
ds2anysudscreendis_meta <- data.frame(m1i =
anysudscreendis_vghrsweek_m1i, sd1i = anysudscreendis_vghrsweek_sd1i,
n1i = anysudscreendis_vghrsweek_n1i, m2i =
anysudscreendis_vghrsweek_m2i, sd2i = anysudscreendis_vghrsweek_sd2i,
n2i = anysudscreendis_vghrsweek_n2i) #MA dataframe
ds2anysudscreendis_meta <- escalc(measure="MD", m1i = m1i, sd1i = sd1i,
n1i = n1i, m2i = m2i, sd2i = sd2i, n2i = n2i,
data=ds2anysudscreendis_meta) #Calculate yi and vi

#FE meta-analysis
(anysudscreendis_meta <- rbind(ds1anysudscreendis_meta,
ds2anysudscreendis_meta))

##        m1i     sd1i n1i      m2i     sd2i n2i      yi     vi
## 1 15.64444 13.80243  45 10.85915 10.03794 142  4.7853 4.9431
## 2 16.68889 11.65571  45 18.97797 25.64589 227 -2.2891 5.9164

(anysudscreendis_vghrsweek_meta <- rma(yi=yi, vi=vi, measure="MD",
method="FE", data=anysudscreendis_meta))

##
## Fixed-Effects Model (k = 2)
##
## Test for Heterogeneity:
## Q(df = 1) = 4.6086, p-val = 0.0318
##
## Model Results:
##
## estimate       se     zval     pval    ci.lb    ci.ub
##   1.5651   1.6411   0.9537   0.3402  -1.6513   4.7816
##
## ---
## Signif. codes:  0 '***' 0.001 '**' 0.01 '*' 0.05 '.' 0.1 ' ' 1
```

Days per week

```
#Study 1
anysudscreendis_yes_ds1_vgdaysweek <- subset(anysudscreendis_yes_ds1,
select = c(vgdaysweek)) #Subset 1i data
anysudscreendis_vgdaysweek_m1i <-
apply(anysudscreendis_yes_ds1_vgdaysweek, 2, mean) #Calculate m1i
anysudscreendis_vgdaysweek_sd1i <-
apply(anysudscreendis_yes_ds1_vgdaysweek, 2, sd) #Calculate sd1i
anysudscreendis_vgdaysweek_n1i <- colSums(!
is.na(anysudscreendis_yes_ds1_vgdaysweek)) #Calculate n1i
anysudscreendis_no_ds1_vgdaysweek <- subset(anysudscreendis_no_ds1,
select = c(vgdaysweek)) #Subset 2i data
anysudscreendis_vgdaysweek_m2i <-
apply(anysudscreendis_no_ds1_vgdaysweek, 2, mean) #Calculate m2i
anysudscreendis_vgdaysweek_sd2i <-
apply(anysudscreendis_no_ds1_vgdaysweek, 2, sd) #Calculate sd2i
anysudscreendis_vgdaysweek_n2i <- colSums(!
is.na(anysudscreendis_no_ds1_vgdaysweek)) #Calculate n2i
ds1anysudscreendis_meta <- data.frame(m1i =
anysudscreendis_vgdaysweek_m1i, sd1i =
anysudscreendis_vgdaysweek_sd1i, n1i = anysudscreendis_vgdaysweek_n1i,
m2i = anysudscreendis_vgdaysweek_m2i, sd2i =
anysudscreendis_vgdaysweek_sd2i, n2i = anysudscreendis_vgdaysweek_n2i)
#MA dataframe
ds1anysudscreendis_meta <- escalc(measure="MD", m1i = m1i, sd1i = sd1i,
n1i = n1i, m2i = m2i, sd2i = sd2i, n2i = n2i,
data=ds1anysudscreendis_meta) #Calculate yi and vi

#Study 2
anysudscreendis_yes_ds2_vgdaysweek <- subset(anysudscreendis_yes_ds2,
select = c(vgdaysweek)) #Subset 1i data
anysudscreendis_vgdaysweek_m1i <-
apply(anysudscreendis_yes_ds2_vgdaysweek, 2, mean) #Calculate m1i
anysudscreendis_vgdaysweek_sd1i <-
apply(anysudscreendis_yes_ds2_vgdaysweek, 2, sd) #Calculate sd1i
anysudscreendis_vgdaysweek_n1i <- colSums(!
is.na(anysudscreendis_yes_ds2_vgdaysweek)) #Calculate n1i
anysudscreendis_no_ds2_vgdaysweek <- subset(anysudscreendis_no_ds2,
select = c(vgdaysweek)) #Subset 2i data
anysudscreendis_vgdaysweek_m2i <-
apply(anysudscreendis_no_ds2_vgdaysweek, 2, mean) #Calculate m2i
anysudscreendis_vgdaysweek_sd2i <-
apply(anysudscreendis_no_ds2_vgdaysweek, 2, sd) #Calculate sd2i
anysudscreendis_vgdaysweek_n2i <- colSums(!
is.na(anysudscreendis_no_ds2_vgdaysweek)) #Calculate n2i
ds2anysudscreendis_meta <- data.frame(m1i =
anysudscreendis_vgdaysweek_m1i, sd1i =
anysudscreendis_vgdaysweek_sd1i, n1i = anysudscreendis_vgdaysweek_n1i,
```

```
m2i = anysudscreendis_vgdaysweek_m2i, sd2i =
anysudscreendis_vgdaysweek_sd2i, n2i = anysudscreendis_vgdaysweek_n2i)
#MA dataframe
ds2anysudscreendis_meta <- escalc(measure="MD", m1i = m1i, sd1i = sd1i,
n1i = n1i, m2i = m2i, sd2i = sd2i, n2i = n2i,
data=ds2anysudscreendis_meta) #Calculate yi and vi

#FE meta-analysis
(anysudscreendis_meta <- rbind(ds1anysudscreendis_meta,
ds2anysudscreendis_meta))

##        m1i     sd1i n1i      m2i     sd2i n2i     yi     vi
## 1 5.955556 1.770408  45 4.845070 2.273173 142 1.1105 0.1060
## 2 5.000000 2.110579  45 4.669604 2.232325 227 0.3304 0.1209

(anysudscreendis_vgdaysweek_meta <- rma(yi=yi, vi=vi, measure="MD",
method="FE", data=anysudscreendis_meta))

##
## Fixed-Effects Model (k = 2)
##
## Test for Heterogeneity:
## Q(df = 1) = 2.6810, p-val = 0.1016
##
## Model Results:
##
## estimate      se     zval     pval    ci.lb    ci.ub
##   0.7460   0.2377   3.1386   0.0017   0.2802   1.2119      **
##
## ---
## Signif. codes:  0 '***' 0.001 '**' 0.01 '*' 0.05 '.' 0.1 ' ' 1
```

Any SUD - year

Replicated Effect

Hours per day

Study 1

```
(vghrsday_anysudscreenyear_ds1 <- t.test(vghrsday ~ alcsudyear,
data=ds1anysudscreen))

##
##  Welch Two Sample t-test
##
## data:  vghrsday by alcsudyear
## t = 0.0080699, df = 27.265, p-value = 0.9936
```

```
## alternative hypothesis: true difference in means is not equal to 0
## 95 percent confidence interval:
##  -0.7382208  0.7440532
## sample estimates:
## mean in group 0 mean in group 1
##        2.176829        2.173913
```

Study 2

```
(vghrsday_anysudscreenyear_ds2 <- t.test(vghrsday ~ alcsudyear,
data=ds2anysudscreen))
```

```
##
##  Welch Two Sample t-test
##
## data:  vghrsday by alcsudyear
## t = 1.5875, df = 51.494, p-value = 0.1185
## alternative hypothesis: true difference in means is not equal to 0
## 95 percent confidence interval:
##  -0.1744497  1.4942878
## sample estimates:
## mean in group 0 mean in group 1
##        3.659919        3.000000
```

Hours per week

Study 1

```
(vghrsweek_anysudscreenyear_ds1 <- t.test(vghrsweek ~ alcsudyear,
data=ds1anysudscreen))
```

```
##
##  Welch Two Sample t-test
##
## data:  vghrsweek by alcsudyear
## t = -0.51735, df = 27.504, p-value = 0.609
## alternative hypothesis: true difference in means is not equal to 0
## 95 percent confidence interval:
##  -6.828211  4.076355
## sample estimates:
## mean in group 0 mean in group 1
##        11.84146        13.21739
```

Study 2

```
(vghrsweek_anysudscreenyear_ds2 <- t.test(vghrsweek ~ alcsudyear,
data=ds2anysudscreen))
```

```
##
##  Welch Two Sample t-test
##
## data:  vghrsweek by alcsudyear
## t = 1.6334, df = 45.81, p-value = 0.1092
## alternative hypothesis: true difference in means is not equal to 0
## 95 percent confidence interval:
##  -1.126245 10.815314
## sample estimates:
## mean in group 0 mean in group 1
##        19.04453        14.20000
```

Days per week

Study 1

```
(vgdaysweek_anysudscreenyear_ds1 <- t.test(vgdaysweek ~ alcsudyear, data=ds1anysudscreen))
```

```
##
##  Welch Two Sample t-test
##
## data:  vgdaysweek by alcsudyear
## t = -2.2487, df = 33.046, p-value = 0.03132
## alternative hypothesis: true difference in means is not equal to 0
## 95 percent confidence interval:
##  -1.73909379 -0.08699316
## sample estimates:
## mean in group 0 mean in group 1
##        5.000000        5.913043
```

Study 2

```
(vgdaysweek_anysudscreenyear_ds2 <- t.test(vgdaysweek ~ alcsudyear, data=ds2anysudscreen))
```

```
##
##  Welch Two Sample t-test
##
## data:  vgdaysweek by alcsudyear
## t = 0.4689, df = 28.688, p-value = 0.6427
## alternative hypothesis: true difference in means is not equal to 0
## 95 percent confidence interval:
##  -0.7566627  1.2065413
## sample estimates:
## mean in group 0 mean in group 1
##        4.744939        4.520000
```

MD

Hours per day

```
#Study 1
anysudscreenyear_yes_ds1_vghrsday <- subset(anysudscreenyear_yes_ds1,
select = c(vghrsday)) #Subset 1i data
anysudscreenyear_vghrsday_m1i <-
apply(anysudscreenyear_yes_ds1_vghrsday, 2, mean) #Calculate m1i
anysudscreenyear_vghrsday_sd1i <-
apply(anysudscreenyear_yes_ds1_vghrsday, 2, sd) #Calculate sd1i
anysudscreenyear_vghrsday_n1i <- colSums(!
is.na(anysudscreenyear_yes_ds1_vghrsday)) #Calculate n1i
anysudscreenyear_no_ds1_vghrsday <- subset(anysudscreenyear_no_ds1,
select = c(vghrsday)) #Subset 2i data
anysudscreenyear_vghrsday_m2i <-
apply(anysudscreenyear_no_ds1_vghrsday, 2, mean) #Calculate m2i
anysudscreenyear_vghrsday_sd2i <-
apply(anysudscreenyear_no_ds1_vghrsday, 2, sd) #Calculate sd2i
anysudscreenyear_vghrsday_n2i <- colSums(!
is.na(anysudscreenyear_no_ds1_vghrsday)) #Calculate n2i
ds1anysudscreenyear_meta <- data.frame(m1i =
anysudscreenyear_vghrsday_m1i, sd1i = anysudscreenyear_vghrsday_sd1i,
n1i = anysudscreenyear_vghrsday_n1i, m2i =
anysudscreenyear_vghrsday_m2i, sd2i = anysudscreenyear_vghrsday_sd2i,
n2i = anysudscreenyear_vghrsday_n2i) #MA dataframe
ds1anysudscreenyear_meta <- escalc(measure="MD", m1i = m1i, sd1i =
sd1i, n1i = n1i, m2i = m2i, sd2i = sd2i, n2i = n2i,
data=ds1anysudscreenyear_meta) #Calculate yi and vi

#Study 2
anysudscreenyear_yes_ds2_vghrsday <- subset(anysudscreenyear_yes_ds2,
select = c(vghrsday)) #Subset 1i data
anysudscreenyear_vghrsday_m1i <-
apply(anysudscreenyear_yes_ds2_vghrsday, 2, mean) #Calculate m1i
anysudscreenyear_vghrsday_sd1i <-
apply(anysudscreenyear_yes_ds2_vghrsday, 2, sd) #Calculate sd1i
anysudscreenyear_vghrsday_n1i <- colSums(!
is.na(anysudscreenyear_yes_ds2_vghrsday)) #Calculate n1i
anysudscreenyear_no_ds2_vghrsday <- subset(anysudscreenyear_no_ds2,
select = c(vghrsday)) #Subset 2i data
anysudscreenyear_vghrsday_m2i <-
apply(anysudscreenyear_no_ds2_vghrsday, 2, mean) #Calculate m2i
anysudscreenyear_vghrsday_sd2i <-
apply(anysudscreenyear_no_ds2_vghrsday, 2, sd) #Calculate sd2i
anysudscreenyear_vghrsday_n2i <- colSums(!
is.na(anysudscreenyear_no_ds2_vghrsday)) #Calculate n2i
ds2anysudscreenyear_meta <- data.frame(m1i =
anysudscreenyear_vghrsday_m1i, sd1i = anysudscreenyear_vghrsday_sd1i,
n1i = anysudscreenyear_vghrsday_n1i, m2i =
```

```
anysudscreenyear_vghrsday_m2i, sd2i = anysudscreenyear_vghrsday_sd2i,
n2i = anysudscreenyear_vghrsday_n2i) #MA dataframe
ds2anysudscreenyear_meta <- escalc(measure="MD", m1i = m1i, sd1i =
sd1i, n1i = n1i, m2i = m2i, sd2i = sd2i, n2i = n2i,
data=ds2anysudscreenyear_meta) #Calculate yi and vi

#FE meta-analysis
(anysudscreenyear_meta <- rbind(ds1anysudscreenyear_meta,
ds2anysudscreenyear_meta))
```

```
##        m1i      sd1i n1i      m2i      sd2i n2i      yi     vi
## 1 2.173913 1.641844  23 2.176829 1.481733 164 -0.0029 0.1306
## 2 3.000000 1.707825  25 3.659919 3.723925 247 -0.6599 0.1728
```

```
(anysudscreenyear_vghrsday_meta <- rma(yi=yi, vi=vi, measure="MD",
method="FE", data=anysudscreenyear_meta))
```

```
##
## Fixed-Effects Model (k = 2)
##
## Test for Heterogeneity:
## Q(df = 1) = 1.4227, p-val = 0.2330
##
## Model Results:
##
## estimate      se     zval     pval    ci.lb    ci.ub
##  -0.2857  0.2727  -1.0476   0.2948  -0.8202   0.2488
##
## ---
## Signif. codes:  0 '***' 0.001 '**' 0.01 '*' 0.05 '.' 0.1 ' ' 1
```

Hours per week

```
#Study 1
anysudscreenyear_yes_ds1_vghrsweek <- subset(anysudscreenyear_yes_ds1,
select = c(vghrsweek)) #Subset 1i data
anysudscreenyear_vghrsweek_m1i <-
apply(anysudscreenyear_yes_ds1_vghrsweek, 2, mean) #Calculate m1i
anysudscreenyear_vghrsweek_sd1i <-
apply(anysudscreenyear_yes_ds1_vghrsweek, 2, sd) #Calculate sd1i
anysudscreenyear_vghrsweek_n1i <- colSums(!
is.na(anysudscreenyear_yes_ds1_vghrsweek)) #Calculate n1i
anysudscreenyear_no_ds1_vghrsweek <- subset(anysudscreenyear_no_ds1,
select = c(vghrsweek)) #Subset 2i data
anysudscreenyear_vghrsweek_m2i <-
apply(anysudscreenyear_no_ds1_vghrsweek, 2, mean) #Calculate m2i
anysudscreenyear_vghrsweek_sd2i <-
apply(anysudscreenyear_no_ds1_vghrsweek, 2, sd) #Calculate sd2i
anysudscreenyear_vghrsweek_n2i <- colSums(!
is.na(anysudscreenyear_no_ds1_vghrsweek)) #Calculate n2i
```

```
ds1anysudscreenyear_meta <- data.frame(m1i =
anysudscreenyear_vghrsweek_m1i, sd1i =
anysudscreenyear_vghrsweek_sd1i, n1i = anysudscreenyear_vghrsweek_n1i,
m2i = anysudscreenyear_vghrsweek_m2i, sd2i =
anysudscreenyear_vghrsweek_sd2i, n2i = anysudscreenyear_vghrsweek_n2i)
#MA dataframe
ds1anysudscreenyear_meta <- escalc(measure="MD", m1i = m1i, sd1i =
sd1i, n1i = n1i, m2i = m2i, sd2i = sd2i, n2i = n2i,
data=ds1anysudscreenyear_meta) #Calculate yi and vi

#Study 2
anysudscreenyear_yes_ds2_vghrsweek <- subset(anysudscreenyear_yes_ds2,
select = c(vghrsweek)) #Subset 1i data
anysudscreenyear_vghrsweek_m1i <-
apply(anysudscreenyear_yes_ds2_vghrsweek, 2, mean) #Calculate m1i
anysudscreenyear_vghrsweek_sd1i <-
apply(anysudscreenyear_yes_ds2_vghrsweek, 2, sd) #Calculate sd1i
anysudscreenyear_vghrsweek_n1i <- colSums(!
is.na(anysudscreenyear_yes_ds2_vghrsweek)) #Calculate n1i
anysudscreenyear_no_ds2_vghrsweek <- subset(anysudscreenyear_no_ds2,
select = c(vghrsweek)) #Subset 2i data
anysudscreenyear_vghrsweek_m2i <-
apply(anysudscreenyear_no_ds2_vghrsweek, 2, mean) #Calculate m2i
anysudscreenyear_vghrsweek_sd2i <-
apply(anysudscreenyear_no_ds2_vghrsweek, 2, sd) #Calculate sd2i
anysudscreenyear_vghrsweek_n2i <- colSums(!
is.na(anysudscreenyear_no_ds2_vghrsweek)) #Calculate n2i
ds2anysudscreenyear_meta <- data.frame(m1i =
anysudscreenyear_vghrsweek_m1i, sd1i =
anysudscreenyear_vghrsweek_sd1i, n1i = anysudscreenyear_vghrsweek_n1i,
m2i = anysudscreenyear_vghrsweek_m2i, sd2i =
anysudscreenyear_vghrsweek_sd2i, n2i = anysudscreenyear_vghrsweek_n2i)
#MA dataframe
ds2anysudscreenyear_meta <- escalc(measure="MD", m1i = m1i, sd1i =
sd1i, n1i = n1i, m2i = m2i, sd2i = sd2i, n2i = n2i,
data=ds2anysudscreenyear_meta) #Calculate yi and vi

#FE meta-analysis
(anysudscreenyear_meta <- rbind(ds1anysudscreenyear_meta,
ds2anysudscreenyear_meta))

##        m1i     sd1i n1i      m2i     sd2i n2i      yi     vi
## 1 13.21739 12.05652  23 11.84146 11.11454 164  1.3759 7.0732
## 2 14.20000 12.56981  25 19.04453 24.73375 247 -4.8445 8.7968

(anysudscreenyear_vghrsweek_meta <- rma(yi=yi, vi=vi, measure="MD",
method="FE", data=anysudscreenyear_meta))

##
## Fixed-Effects Model (k = 2)
##
```

```
## Test for Heterogeneity:
## Q(df = 1) = 2.4382, p-val = 0.1184
##
## Model Results:
##
## estimate      se     zval     pval    ci.lb    ci.ub
##  -1.3965  1.9801  -0.7053   0.4806  -5.2774   2.4844
##
## ---
## Signif. codes:  0 '***' 0.001 '**' 0.01 '*' 0.05 '.' 0.1 ' ' 1
```

Days per week

```
#Study 1
anysudscreenyear_yes_ds1_vgdaysweek <-
subset(anysudscreenyear_yes_ds1, select = c(vgdaysweek)) #Subset 1i data
anysudscreenyear_vgdaysweek_m1i <-
apply(anysudscreenyear_yes_ds1_vgdaysweek, 2, mean) #Calculate m1i
anysudscreenyear_vgdaysweek_sd1i <-
apply(anysudscreenyear_yes_ds1_vgdaysweek, 2, sd) #Calculate sd1i
anysudscreenyear_vgdaysweek_n1i <- colSums(!
is.na(anysudscreenyear_yes_ds1_vgdaysweek)) #Calculate n1i
anysudscreenyear_no_ds1_vgdaysweek <- subset(anysudscreenyear_no_ds1,
select = c(vgdaysweek)) #Subset 2i data
anysudscreenyear_vgdaysweek_m2i <-
apply(anysudscreenyear_no_ds1_vgdaysweek, 2, mean) #Calculate m2i
anysudscreenyear_vgdaysweek_sd2i <-
apply(anysudscreenyear_no_ds1_vgdaysweek, 2, sd) #Calculate sd2i
anysudscreenyear_vgdaysweek_n2i <- colSums(!
is.na(anysudscreenyear_no_ds1_vgdaysweek)) #Calculate n2i
ds1anysudscreenyear_meta <- data.frame(m1i =
anysudscreenyear_vgdaysweek_m1i, sd1i =
anysudscreenyear_vgdaysweek_sd1i, n1i =
anysudscreenyear_vgdaysweek_n1i, m2i =
anysudscreenyear_vgdaysweek_m2i, sd2i =
anysudscreenyear_vgdaysweek_sd2i, n2i =
anysudscreenyear_vgdaysweek_n2i) #MA dataframe
ds1anysudscreenyear_meta <- escalc(measure="MD", m1i = m1i, sd1i =
sd1i, n1i = n1i, m2i = m2i, sd2i = sd2i, n2i = n2i,
data=ds1anysudscreenyear_meta) #Calculate yi and vi

#Study 2
anysudscreenyear_yes_ds2_vgdaysweek <-
subset(anysudscreenyear_yes_ds2, select = c(vgdaysweek)) #Subset 1i data
anysudscreenyear_vgdaysweek_m1i <-
apply(anysudscreenyear_yes_ds2_vgdaysweek, 2, mean) #Calculate m1i
anysudscreenyear_vgdaysweek_sd1i <-
apply(anysudscreenyear_yes_ds2_vgdaysweek, 2, sd) #Calculate sd1i
anysudscreenyear_vgdaysweek_n1i <- colSums(!
```

```
is.na(anysudscreenyear_yes_ds2_vgdaysweek)) #Calculate n1i
anysudscreenyear_no_ds2_vgdaysweek <- subset(anysudscreenyear_no_ds2,
select = c(vgdaysweek)) #Subset 2i data
anysudscreenyear_vgdaysweek_m2i <-
apply(anysudscreenyear_no_ds2_vgdaysweek, 2, mean) #Calculate m2i
anysudscreenyear_vgdaysweek_sd2i <-
apply(anysudscreenyear_no_ds2_vgdaysweek, 2, sd) #Calculate sd2i
anysudscreenyear_vgdaysweek_n2i <- colSums(!
is.na(anysudscreenyear_no_ds2_vgdaysweek)) #Calculate n2i
ds2anysudscreenyear_meta <- data.frame(m1i =
anysudscreenyear_vgdaysweek_m1i, sd1i =
anysudscreenyear_vgdaysweek_sd1i, n1i =
anysudscreenyear_vgdaysweek_n1i, m2i =
anysudscreenyear_vgdaysweek_m2i, sd2i =
anysudscreenyear_vgdaysweek_sd2i, n2i =
anysudscreenyear_vgdaysweek_n2i) #MA dataframe
ds2anysudscreenyear_meta <- escalc(measure="MD", m1i = m1i, sd1i =
sd1i, n1i = n1i, m2i = m2i, sd2i = sd2i, n2i = n2i,
data=ds2anysudscreenyear_meta) #Calculate yi and vi

#FE meta-analysis
(anysudscreenyear_meta <- rbind(ds1anysudscreenyear_meta,
ds2anysudscreenyear_meta))

##        m1i     sd1i n1i      m2i     sd2i n2i      yi     vi
## 1 5.913043 1.755848  23 5.000000 2.248380 164  0.9130 0.1649
## 2 4.520000 2.293469  25 4.744939 2.207647 247 -0.2249 0.2301

(anysudscreenyear_vgdaysweek_meta <- rma(yi=yi, vi=vi, measure="MD",
method="FE", data=anysudscreenyear_meta))

##
## Fixed-Effects Model (k = 2)
##
## Test for Heterogeneity:
## Q(df = 1) = 3.2785, p-val = 0.0702
##
## Model Results:
##
## estimate      se     zval     pval    ci.lb    ci.ub
##   0.4381   0.3099   1.4134   0.1575  -0.1694   1.0455
##
## ---
## Signif. codes:  0 '***' 0.001 '**' 0.01 '*' 0.05 '.' 0.1 ' ' 1
```

Any SUD - month

Replicated Effect

Hours per day

Study 1 - CANNOT CALCULATE

Study 2

```
(vghrsday_anysudscreenmonth_ds2 <- t.test(vghrsday ~ alcsudmonth,
data=ds2anysudscreen))

##
##  Welch Two Sample t-test
##
## data:  vghrsday by alcsudmonth
## t = 0.73757, df = 7.7998, p-value = 0.4824
## alternative hypothesis: true difference in means is not equal to 0
## 95 percent confidence interval:
##  -1.002759  1.939686
## sample estimates:
## mean in group 0 mean in group 1
##        3.611321        3.142857
```

Hours per week

Study 1 - CANNOT CALCULATE

Study 2

```
(vghrsweek_anysudscreenmonth_ds2 <- t.test(vghrsweek ~ alcsudmonth,
data=ds2anysudscreen))

##
##  Welch Two Sample t-test
##
## data:  vghrsweek by alcsudmonth
## t = 0.44469, df = 7.4127, p-value = 0.6692
## alternative hypothesis: true difference in means is not equal to 0
## 95 percent confidence interval:
##  -8.862791 13.025594
## sample estimates:
## mean in group 0 mean in group 1
##        18.65283        16.57143
```

Days per week

Study 1 - CANNOT CALCULATE

Study 2

```
(vgdaysweek_anysudscreenmonth_ds2 <- t.test(vgdaysweek ~ alcsudmonth,
data=ds2anysudscreen))

##
##  Welch Two Sample t-test
##
## data:  vgdaysweek by alcsudmonth
## t = -0.35443, df = 6.3654, p-value = 0.7345
## alternative hypothesis: true difference in means is not equal to 0
## 95 percent confidence interval:
##  -2.210045  1.644007
## sample estimates:
## mean in group 0 mean in group 1
##        4.716981        5.000000
```

MD

Hours per day

```
#Study 1
anysudscreenmonth_yes_ds1_vghrsday <-
subset(anysudscreenmonth_yes_ds1, select = c(vghrsday)) #Subset 1i data
anysudscreenmonth_vghrsday_m1i <-
apply(anysudscreenmonth_yes_ds1_vghrsday, 2, mean) #Calculate m1i
anysudscreenmonth_vghrsday_sd1i <-
apply(anysudscreenmonth_yes_ds1_vghrsday, 2, sd) #Calculate sd1i
anysudscreenmonth_vghrsday_n1i <- colSums(!
is.na(anysudscreenmonth_yes_ds1_vghrsday)) #Calculate n1i
anysudscreenmonth_no_ds1_vghrsday <-
subset(anysudscreenmonth_no_ds1, select = c(vghrsday)) #Subset 2i data
anysudscreenmonth_vghrsday_m2i <-
apply(anysudscreenmonth_no_ds1_vghrsday, 2, mean) #Calculate m2i
anysudscreenmonth_vghrsday_sd2i <-
apply(anysudscreenmonth_no_ds1_vghrsday, 2, sd) #Calculate sd2i
anysudscreenmonth_vghrsday_n2i <- colSums(!
is.na(anysudscreenmonth_no_ds1_vghrsday)) #Calculate n2i
ds1anysudscreenmonth_meta <- data.frame(m1i =
anysudscreenmonth_vghrsday_m1i, sd1i =
anysudscreenmonth_vghrsday_sd1i, n1i = anysudscreenmonth_vghrsday_n1i,
m2i = anysudscreenmonth_vghrsday_m2i, sd2i =
anysudscreenmonth_vghrsday_sd2i, n2i =
anysudscreenmonth_vghrsday_n2i) #MA dataframe
ds1anysudscreenmonth_meta <- escalc(measure="MD", m1i = m1i, sd1i =
sd1i, n1i = n1i, m2i = m2i, sd2i = sd2i, n2i = n2i,
```

```
data=ds1anysudscreenmonth_meta) #Calculate yi and vi

#Study 2
anysudscreenmonth_yes_ds2_vghrsday <-
subset(anysudscreenmonth_yes_ds2, select = c(vghrsday)) #Subset 1i data
anysudscreenmonth_vghrsday_m1i <-
apply(anysudscreenmonth_yes_ds2_vghrsday, 2, mean) #Calculate m1i
anysudscreenmonth_vghrsday_sd1i <-
apply(anysudscreenmonth_yes_ds2_vghrsday, 2, sd) #Calculate sd1i
anysudscreenmonth_vghrsday_n1i <- colSums(!
is.na(anysudscreenmonth_yes_ds2_vghrsday)) #Calculate n1i
anysudscreenmonth_no_ds2_vghrsday <-
subset(anysudscreenmonth_no_ds2, select = c(vghrsday)) #Subset 2i data
anysudscreenmonth_vghrsday_m2i <-
apply(anysudscreenmonth_no_ds2_vghrsday, 2, mean) #Calculate m2i
anysudscreenmonth_vghrsday_sd2i <-
apply(anysudscreenmonth_no_ds2_vghrsday, 2, sd) #Calculate sd2i
anysudscreenmonth_vghrsday_n2i <- colSums(!
is.na(anysudscreenmonth_no_ds2_vghrsday)) #Calculate n2i
ds2anysudscreenmonth_meta <- data.frame(m1i =
anysudscreenmonth_vghrsday_m1i, sd1i =
anysudscreenmonth_vghrsday_sd1i, n1i = anysudscreenmonth_vghrsday_n1i,
m2i = anysudscreenmonth_vghrsday_m2i, sd2i =
anysudscreenmonth_vghrsday_sd2i, n2i =
anysudscreenmonth_vghrsday_n2i) #MA dataframe
ds2anysudscreenmonth_meta <- escalc(measure="MD", m1i = m1i, sd1i =
sd1i, n1i = n1i, m2i = m2i, sd2i = sd2i, n2i = n2i,
data=ds2anysudscreenmonth_meta) #Calculate yi and vi

#FE meta-analysis
(anysudscreenmonth_meta <- rbind(ds1anysudscreenmonth_meta,
ds2anysudscreenmonth_meta))

##        m1i     sd1i n1i      m2i     sd2i n2i      yi     vi
## 1      NaN       NA   0 2.176471 1.497626 187      NA     NA
## 2 3.142857 1.573592   7 3.611321 3.628050 265 -0.4685 0.4034

(anysudscreenmonth_vghrsday_meta <- rma(yi=yi, vi=vi, measure="MD",
method="FE", data=anysudscreenmonth_meta))

##
## Fixed-Effects Model (k = 1)
##
## Test for Heterogeneity:
## Q(df = 0) = 0.0000, p-val = 1.0000
##
## Model Results:
##
## estimate      se     zval     pval    ci.lb    ci.ub
##  -0.4685   0.6351  -0.7376   0.4608  -1.7133   0.7764
##
```

```
## ---
## Signif. codes:  0 '***' 0.001 '**' 0.01 '*' 0.05 '.' 0.1 ' ' 1
```

Hours per week

```
#Study 1
anysudscreenmonth_yes_ds1_vghrsweek <-
subset(anysudscreenmonth_yes_ds1, select = c(vghrsweek)) #Subset 1i data
anysudscreenmonth_vghrsweek_m1i <-
apply(anysudscreenmonth_yes_ds1_vghrsweek, 2, mean) #Calculate m1i
anysudscreenmonth_vghrsweek_sd1i <-
apply(anysudscreenmonth_yes_ds1_vghrsweek, 2, sd) #Calculate sd1i
anysudscreenmonth_vghrsweek_n1i <- colSums(!
is.na(anysudscreenmonth_yes_ds1_vghrsweek)) #Calculate n1i
anysudscreenmonth_no_ds1_vghrsweek <-
subset(anysudscreenmonth_no_ds1, select = c(vghrsweek)) #Subset 2i data
anysudscreenmonth_vghrsweek_m2i <-
apply(anysudscreenmonth_no_ds1_vghrsweek, 2, mean) #Calculate m2i
anysudscreenmonth_vghrsweek_sd2i <-
apply(anysudscreenmonth_no_ds1_vghrsweek, 2, sd) #Calculate sd2i
anysudscreenmonth_vghrsweek_n2i <- colSums(!
is.na(anysudscreenmonth_no_ds1_vghrsweek)) #Calculate n2i
ds1anysudscreenmonth_meta <- data.frame(m1i =
anysudscreenmonth_vghrsweek_m1i, sd1i =
anysudscreenmonth_vghrsweek_sd1i, n1i =
anysudscreenmonth_vghrsweek_n1i, m2i =
anysudscreenmonth_vghrsweek_m2i, sd2i =
anysudscreenmonth_vghrsweek_sd2i, n2i =
anysudscreenmonth_vghrsweek_n2i) #MA dataframe
ds1anysudscreenmonth_meta <- escalc(measure="MD", m1i = m1i, sd1i =
sd1i, n1i = n1i, m2i = m2i, sd2i = sd2i, n2i = n2i,
data=ds1anysudscreenmonth_meta) #Calculate yi and vi

#Study 2
anysudscreenmonth_yes_ds2_vghrsweek <-
subset(anysudscreenmonth_yes_ds2, select = c(vghrsweek)) #Subset 1i data
anysudscreenmonth_vghrsweek_m1i <-
apply(anysudscreenmonth_yes_ds2_vghrsweek, 2, mean) #Calculate m1i
anysudscreenmonth_vghrsweek_sd1i <-
apply(anysudscreenmonth_yes_ds2_vghrsweek, 2, sd) #Calculate sd1i
anysudscreenmonth_vghrsweek_n1i <- colSums(!
is.na(anysudscreenmonth_yes_ds2_vghrsweek)) #Calculate n1i
anysudscreenmonth_no_ds2_vghrsweek <-
subset(anysudscreenmonth_no_ds2, select = c(vghrsweek)) #Subset 2i data
anysudscreenmonth_vghrsweek_m2i <-
apply(anysudscreenmonth_no_ds2_vghrsweek, 2, mean) #Calculate m2i
anysudscreenmonth_vghrsweek_sd2i <-
apply(anysudscreenmonth_no_ds2_vghrsweek, 2, sd) #Calculate sd2i
anysudscreenmonth_vghrsweek_n2i <- colSums(!
```

```
is.na(anysudscreenmonth_no_ds2_vghrsweek)) #Calculate n2i
ds2anysudscreenmonth_meta <- data.frame(m1i =
anysudscreenmonth_vghrsweek_m1i, sd1i =
anysudscreenmonth_vghrsweek_sd1i, n1i =
anysudscreenmonth_vghrsweek_n1i, m2i =
anysudscreenmonth_vghrsweek_m2i, sd2i =
anysudscreenmonth_vghrsweek_sd2i, n2i =
anysudscreenmonth_vghrsweek_n2i) #MA dataframe
ds2anysudscreenmonth_meta <- escalc(measure="MD", m1i = m1i, sd1i =
sd1i, n1i = n1i, m2i = m2i, sd2i = sd2i, n2i = n2i,
data=ds2anysudscreenmonth_meta) #Calculate yi and vi

#FE meta-analysis
(anysudscreenmonth_meta <- rbind(ds1anysudscreenmonth_meta,
ds2anysudscreenmonth_meta))

##        m1i      sd1i n1i      m2i      sd2i n2i      yi      vi
## 1      NaN       NA   0 12.01070 11.20963 187      NA      NA
## 2 16.57143 11.74531   7 18.65283 24.14916 265 -2.0814 21.9082

(anysudscreenmonth_vghrsweek_meta <- rma(yi=yi, vi=vi, measure="MD",
method="FE", data=anysudscreenmonth_meta))

##
## Fixed-Effects Model (k = 1)
##
## Test for Heterogeneity:
## Q(df = 0) = 0.0000, p-val = 1.0000
##
## Model Results:
##
## estimate       se     zval     pval    ci.lb    ci.ub
##  -2.0814   4.6806  -0.4447   0.6565 -11.2552   7.0924
##
## ---
## Signif. codes:  0 '***' 0.001 '**' 0.01 '*' 0.05 '.' 0.1 ' ' 1
```

Days per week

```
#Study 1
anysudscreenmonth_yes_ds1_vgdaysweek <-
subset(anysudscreenmonth_yes_ds1, select = c(vgdaysweek)) #Subset 1i
data
anysudscreenmonth_vgdaysweek_m1i <-
apply(anysudscreenmonth_yes_ds1_vgdaysweek, 2, mean) #Calculate m1i
anysudscreenmonth_vgdaysweek_sd1i <-
apply(anysudscreenmonth_yes_ds1_vgdaysweek, 2, sd) #Calculate sd1i
anysudscreenmonth_vgdaysweek_n1i <- colSums(!
is.na(anysudscreenmonth_yes_ds1_vgdaysweek)) #Calculate n1i
anysudscreenmonth_no_ds1_vgdaysweek <-
```

```
subset(anysudscreenmonth_no_ds1, select = c(vgdaysweek)) #Subset 2i
data
anysudscreenmonth_vgdaysweek_m2i <-
apply(anysudscreenmonth_no_ds1_vgdaysweek, 2, mean) #Calculate m2i
anysudscreenmonth_vgdaysweek_sd2i <-
apply(anysudscreenmonth_no_ds1_vgdaysweek, 2, sd) #Calculate sd2i
anysudscreenmonth_vgdaysweek_n2i <- colSums(!
is.na(anysudscreenmonth_no_ds1_vgdaysweek)) #Calculate n2i
ds1anysudscreenmonth_meta <- data.frame(m1i =
anysudscreenmonth_vgdaysweek_m1i, sd1i =
anysudscreenmonth_vgdaysweek_sd1i, n1i =
anysudscreenmonth_vgdaysweek_n1i, m2i =
anysudscreenmonth_vgdaysweek_m2i, sd2i =
anysudscreenmonth_vgdaysweek_sd2i, n2i =
anysudscreenmonth_vgdaysweek_n2i) #MA dataframe
ds1anysudscreenmonth_meta <- escalc(measure="MD", m1i = m1i, sd1i =
sd1i, n1i = n1i, m2i = m2i, sd2i = sd2i, n2i = n2i,
data=ds1anysudscreenmonth_meta) #Calculate yi and vi

#Study 2
anysudscreenmonth_yes_ds2_vgdaysweek <-
subset(anysudscreenmonth_yes_ds2, select = c(vgdaysweek)) #Subset 1i
data
anysudscreenmonth_vgdaysweek_m1i <-
apply(anysudscreenmonth_yes_ds2_vgdaysweek, 2, mean) #Calculate m1i
anysudscreenmonth_vgdaysweek_sd1i <-
apply(anysudscreenmonth_yes_ds2_vgdaysweek, 2, sd) #Calculate sd1i
anysudscreenmonth_vgdaysweek_n1i <- colSums(!
is.na(anysudscreenmonth_yes_ds2_vgdaysweek)) #Calculate n1i
anysudscreenmonth_no_ds2_vgdaysweek <-
subset(anysudscreenmonth_no_ds2, select = c(vgdaysweek)) #Subset 2i
data
anysudscreenmonth_vgdaysweek_m2i <-
apply(anysudscreenmonth_no_ds2_vgdaysweek, 2, mean) #Calculate m2i
anysudscreenmonth_vgdaysweek_sd2i <-
apply(anysudscreenmonth_no_ds2_vgdaysweek, 2, sd) #Calculate sd2i
anysudscreenmonth_vgdaysweek_n2i <- colSums(!
is.na(anysudscreenmonth_no_ds2_vgdaysweek)) #Calculate n2i
ds2anysudscreenmonth_meta <- data.frame(m1i =
anysudscreenmonth_vgdaysweek_m1i, sd1i =
anysudscreenmonth_vgdaysweek_sd1i, n1i =
anysudscreenmonth_vgdaysweek_n1i, m2i =
anysudscreenmonth_vgdaysweek_m2i, sd2i =
anysudscreenmonth_vgdaysweek_sd2i, n2i =
anysudscreenmonth_vgdaysweek_n2i) #MA dataframe
ds2anysudscreenmonth_meta <- escalc(measure="MD", m1i = m1i, sd1i =
sd1i, n1i = n1i, m2i = m2i, sd2i = sd2i, n2i = n2i,
data=ds2anysudscreenmonth_meta) #Calculate yi and vi

#FE meta-analysis
```

```
(anysudscreenmonth_meta <- rbind(ds1anysudscreenmonth_meta,
ds2anysudscreenmonth_meta))
```

```
##   m1i     sd1i n1i      m2i     sd2i n2i     yi     vi
## 1 NaN       NA   0 5.112299 2.210242 187     NA     NA
## 2   5 2.081666   7 4.716981 2.218870 265 0.2830 0.6376
```

```
(anysudscreenmonth_vgdaysweek_meta <- rma(yi=yi, vi=vi, measure="MD",
method="FE", data=anysudscreenmonth_meta))
```

```
##
## Fixed-Effects Model (k = 1)
##
## Test for Heterogeneity:
## Q(df = 0) = 0.0000, p-val = 1.0000
##
## Model Results:
##
## estimate      se     zval     pval    ci.lb    ci.ub
##   0.2830   0.7985   0.3544   0.7230  -1.2820   1.8481
##
## ---
## Signif. codes:  0 '***' 0.001 '**' 0.01 '*' 0.05 '.' 0.1 ' ' 1
```

AUD - discharge

Replicated Effect

Hours per day

Study 1

```
(vghrsday_auditdis_ds1 <- t.test(vghrsday ~ alcsuddis, data=ds1audit))
```

```
##
##  Welch Two Sample t-test
##
## data:  vghrsday by alcsuddis
## t = -1.8775, df = 35.221, p-value = 0.06875
## alternative hypothesis: true difference in means is not equal to 0
## 95 percent confidence interval:
##  -1.58705733  0.06178851
## sample estimates:
## mean in group 0 mean in group 1
##        2.108333        2.870968
```

Study 2

```
(vghrsday_auditdis_ds2 <- t.test(vghrsday ~ alcsuddis, data=ds2audit))

##
##  Welch Two Sample t-test
##
## data:  vghrsday by alcsuddis
## t = 0.2508, df = 37.667, p-value = 0.8033
## alternative hypothesis: true difference in means is not equal to 0
## 95 percent confidence interval:
##  -0.7891072  1.0122085
## sample estimates:
## mean in group 0 mean in group 1
##        3.424051        3.312500
```

Hours per week

Study 1

```
(vghrsweek_auditdis_ds1 <- t.test(vghrsweek ~ alcsuddis, data=ds1audit))

##
##  Welch Two Sample t-test
##
## data:  vghrsweek by alcsuddis
## t = -2.5308, df = 36.104, p-value = 0.01588
## alternative hypothesis: true difference in means is not equal to 0
## 95 percent confidence interval:
##  -13.290758  -1.466231
## sample estimates:
## mean in group 0 mean in group 1
##        11.26667        18.64516
```

Study 2

```
(vghrsweek_auditdis_ds2 <- t.test(vghrsweek ~ alcsuddis, data=ds2audit))

##
##  Welch Two Sample t-test
##
## data:  vghrsweek by alcsuddis
## t = 0.20146, df = 33.112, p-value = 0.8416
## alternative hypothesis: true difference in means is not equal to 0
## 95 percent confidence interval:
##  -5.822658  7.102721
## sample estimates:
## mean in group 0 mean in group 1
##        17.70253        17.06250
```

Days per week

Study 1

```
(vgdaysweek_auditdis_ds1 <- t.test(vgdaysweek ~ alcsuddis, data=ds1audit))
```

```
##
##  Welch Two Sample t-test
##
## data:  vgdaysweek by alcsuddis
## t = -3.4954, df = 61.123, p-value = 0.0008875
## alternative hypothesis: true difference in means is not equal to 0
## 95 percent confidence interval:
##  -1.992539 -0.542407
## sample estimates:
## mean in group 0 mean in group 1
##        4.958333        6.225806
```

Study 2

```
(vgdaysweek_auditdis_ds2 <- t.test(vgdaysweek ~ alcsuddis, data=ds2audit))
```

```
##
##  Welch Two Sample t-test
##
## data:  vgdaysweek by alcsuddis
## t = -0.57634, df = 19.599, p-value = 0.5709
## alternative hypothesis: true difference in means is not equal to 0
## 95 percent confidence interval:
##  -1.3133202  0.7452823
## sample estimates:
## mean in group 0 mean in group 1
##        4.778481        5.062500
```

MD

Hours per day

```
#Study 1
auditdis_yes_ds1_vghrsday <- subset(auditdis_yes_ds1, select = c(vghrsday))
#Subset 1i data
auditdis_vghrsday_m1i <- apply(auditdis_yes_ds1_vghrsday, 2, mean)
#Calculate m1i
auditdis_vghrsday_sd1i <- apply(auditdis_yes_ds1_vghrsday, 2, sd)
#Calculate sd1i
```

```
auditdis_vghrsday_n1i <- colSums(!is.na(auditdis_yes_ds1_vghrsday))
#Calculate n1i
auditdis_no_ds1_vghrsday <- subset(auditdis_no_ds1, select = c(vghrsday))
#Subset 2i data
auditdis_vghrsday_m2i <- apply(auditdis_no_ds1_vghrsday, 2, mean)
#Calculate m2i
auditdis_vghrsday_sd2i <- apply(auditdis_no_ds1_vghrsday, 2, sd) #Calculate
sd2i
auditdis_vghrsday_n2i <- colSums(!is.na(auditdis_no_ds1_vghrsday))
#Calculate n2i
ds1auditdis_meta <- data.frame(m1i = auditdis_vghrsday_m1i, sd1i =
auditdis_vghrsday_sd1i, n1i = auditdis_vghrsday_n1i, m2i =
auditdis_vghrsday_m2i, sd2i = auditdis_vghrsday_sd2i, n2i =
auditdis_vghrsday_n2i) #MA dataframe
ds1auditdis_meta <- escalc(measure="MD", m1i = m1i, sd1i = sd1i, n1i =
n1i, m2i = m2i, sd2i = sd2i, n2i = n2i, data=ds1auditdis_meta) #Calculate yi
and vi

#Study 2
auditdis_yes_ds2_vghrsday <- subset(auditdis_yes_ds2, select = c(vghrsday))
#Subset 1i data
auditdis_vghrsday_m1i <- apply(auditdis_yes_ds2_vghrsday, 2, mean)
#Calculate m1i
auditdis_vghrsday_sd1i <- apply(auditdis_yes_ds2_vghrsday, 2, sd)
#Calculate sd1i
auditdis_vghrsday_n1i <- colSums(!is.na(auditdis_yes_ds2_vghrsday))
#Calculate n1i
auditdis_no_ds2_vghrsday <- subset(auditdis_no_ds2, select = c(vghrsday))
#Subset 2i data
auditdis_vghrsday_m2i <- apply(auditdis_no_ds2_vghrsday, 2, mean)
#Calculate m2i
auditdis_vghrsday_sd2i <- apply(auditdis_no_ds2_vghrsday, 2, sd) #Calculate
sd2i
auditdis_vghrsday_n2i <- colSums(!is.na(auditdis_no_ds2_vghrsday))
#Calculate n2i
ds2auditdis_meta <- data.frame(m1i = auditdis_vghrsday_m1i, sd1i =
auditdis_vghrsday_sd1i, n1i = auditdis_vghrsday_n1i, m2i =
auditdis_vghrsday_m2i, sd2i = auditdis_vghrsday_sd2i, n2i =
auditdis_vghrsday_n2i) #MA dataframe
ds2auditdis_meta <- escalc(measure="MD", m1i = m1i, sd1i = sd1i, n1i =
n1i, m2i = m2i, sd2i = sd2i, n2i = n2i, data=ds2auditdis_meta) #Calculate yi
and vi

#FE meta-analysis
(auditdis_meta <- rbind(ds1auditdis_meta, ds2auditdis_meta))

##        m1i     sd1i n1i      m2i    sd2i n2i      yi     vi
## 1 2.870968 2.171665  31 2.108333 1.24209 120  0.7626 0.1650
## 2 3.312500 1.400893  16 3.424051 3.44621 158 -0.1116 0.1978
```

```
(auditdis_vghrsday_meta <- rma(yi=yi, vi=vi, measure="MD", method="FE",
data=auditdis_meta))

##
## Fixed-Effects Model (k = 2)
##
## Test for Heterogeneity:
## Q(df = 1) = 2.1063, p-val = 0.1467
##
## Model Results:
##
## estimate      se     zval     pval    ci.lb    ci.ub
##   0.3651   0.2999   1.2173   0.2235  -0.2228   0.9530
##
## ---
## Signif. codes:  0 '***' 0.001 '**' 0.01 '*' 0.05 '.' 0.1 ' ' 1
```

Hours per week

```
#Study 1
auditdis_yes_ds1_vghrsweek <- subset(auditdis_yes_ds1, select =
c(vghrsweek)) #Subset 1i data
auditdis_vghrsweek_m1i <- apply(auditdis_yes_ds1_vghrsweek, 2, mean)
#Calculate m1i
auditdis_vghrsweek_sd1i <- apply(auditdis_yes_ds1_vghrsweek, 2, sd)
#Calculate sd1i
auditdis_vghrsweek_n1i <- colSums(!is.na(auditdis_yes_ds1_vghrsweek))
#Calculate n1i
auditdis_no_ds1_vghrsweek <- subset(auditdis_no_ds1, select =
c(vghrsweek)) #Subset 2i data
auditdis_vghrsweek_m2i <- apply(auditdis_no_ds1_vghrsweek, 2, mean)
#Calculate m2i
auditdis_vghrsweek_sd2i <- apply(auditdis_no_ds1_vghrsweek, 2, sd)
#Calculate sd2i
auditdis_vghrsweek_n2i <- colSums(!is.na(auditdis_no_ds1_vghrsweek))
#Calculate n2i
ds1auditdis_meta <- data.frame(m1i = auditdis_vghrsweek_m1i, sd1i =
auditdis_vghrsweek_sd1i, n1i = auditdis_vghrsweek_n1i, m2i =
auditdis_vghrsweek_m2i, sd2i = auditdis_vghrsweek_sd2i, n2i =
auditdis_vghrsweek_n2i) #MA dataframe
ds1auditdis_meta <- escalc(measure="MD", m1i = m1i, sd1i = sd1i, n1i =
n1i, m2i = m2i, sd2i = sd2i, n2i = n2i, data=ds1auditdis_meta) #Calculate yi
and vi

#Study 2
auditdis_yes_ds2_vghrsweek <- subset(auditdis_yes_ds2, select =
c(vghrsweek)) #Subset 1i data
auditdis_vghrsweek_m1i <- apply(auditdis_yes_ds2_vghrsweek, 2, mean)
#Calculate m1i
```

```
auditdis_vghrsweek_sd1i <- apply(auditdis_yes_ds2_vghrsweek, 2, sd)
#Calculate sd1i
auditdis_vghrsweek_n1i <- colSums(!is.na(auditdis_yes_ds2_vghrsweek))
#Calculate n1i
auditdis_no_ds2_vghrsweek <- subset(auditdis_no_ds2, select =
c(vghrsweek)) #Subset 2i data
auditdis_vghrsweek_m2i <- apply(auditdis_no_ds2_vghrsweek, 2, mean)
#Calculate m2i
auditdis_vghrsweek_sd2i <- apply(auditdis_no_ds2_vghrsweek, 2, sd)
#Calculate sd2i
auditdis_vghrsweek_n2i <- colSums(!is.na(auditdis_no_ds2_vghrsweek))
#Calculate n2i
ds2auditdis_meta <- data.frame(m1i = auditdis_vghrsweek_m1i, sd1i =
auditdis_vghrsweek_sd1i, n1i = auditdis_vghrsweek_n1i, m2i =
auditdis_vghrsweek_m2i, sd2i = auditdis_vghrsweek_sd2i, n2i =
auditdis_vghrsweek_n2i) #MA dataframe
ds2auditdis_meta <- escalc(measure="MD", m1i = m1i, sd1i = sd1i, n1i =
n1i, m2i = m2i, sd2i = sd2i, n2i = n2i, data=ds2auditdis_meta) #Calculate yi
and vi

#FE meta-analysis
(auditdis_meta <- rbind(ds1auditdis_meta, ds2auditdis_meta))

##        m1i     sd1i n1i      m2i      sd2i n2i      yi      vi
## 1 18.64516 15.48881  31 11.26667  9.557199 120  7.3785  8.5000
## 2 17.06250 10.36320  16 17.70253 23.111612 158 -0.6400 10.0929

(auditdis_vghrsweek_meta <- rma(yi=yi, vi=vi, measure="MD",
method="FE", data=auditdis_meta))

##
## Fixed-Effects Model (k = 2)
##
## Test for Heterogeneity:
## Q(df = 1) = 3.4581, p-val = 0.0629
##
## Model Results:
##
## estimate      se     zval     pval    ci.lb    ci.ub
##   3.7127   2.1480   1.7284   0.0839  -0.4974   7.9228       .
##
## ---
## Signif. codes:  0 '***' 0.001 '**' 0.01 '*' 0.05 '.' 0.1 ' ' 1
```

Days per week

```
#Study 1
auditdis_yes_ds1_vgdaysweek <- subset(auditdis_yes_ds1, select =
c(vgdaysweek)) #Subset 1i data
auditdis_vgdaysweek_m1i <- apply(auditdis_yes_ds1_vgdaysweek, 2, mean)
```

```
#Calculate m1i
auditdis_vgdaysweek_sd1i <- apply(auditdis_yes_ds1_vgdaysweek, 2, sd)
#Calculate sd1i
auditdis_vgdaysweek_n1i <- colSums(!is.na(auditdis_yes_ds1_vgdaysweek))
#Calculate n1i
auditdis_no_ds1_vgdaysweek <- subset(auditdis_no_ds1, select =
c(vgdaysweek)) #Subset 2i data
auditdis_vgdaysweek_m2i <- apply(auditdis_no_ds1_vgdaysweek, 2, mean)
#Calculate m2i
auditdis_vgdaysweek_sd2i <- apply(auditdis_no_ds1_vgdaysweek, 2, sd)
#Calculate sd2i
auditdis_vgdaysweek_n2i <- colSums(!is.na(auditdis_no_ds1_vgdaysweek))
#Calculate n2i
ds1auditdis_meta <- data.frame(m1i = auditdis_vgdaysweek_m1i, sd1i =
auditdis_vgdaysweek_sd1i, n1i = auditdis_vgdaysweek_n1i, m2i =
auditdis_vgdaysweek_m2i, sd2i = auditdis_vgdaysweek_sd2i, n2i =
auditdis_vgdaysweek_n2i) #MA dataframe
ds1auditdis_meta <- escalc(measure="MD", m1i = m1i, sd1i = sd1i, n1i =
n1i, m2i = m2i, sd2i = sd2i, n2i = n2i, data=ds1auditdis_meta) #Calculate yi
and vi

#Study 2
auditdis_yes_ds2_vgdaysweek <- subset(auditdis_yes_ds2, select =
c(vgdaysweek)) #Subset 1i data
auditdis_vgdaysweek_m1i <- apply(auditdis_yes_ds2_vgdaysweek, 2, mean)
#Calculate m1i
auditdis_vgdaysweek_sd1i <- apply(auditdis_yes_ds2_vgdaysweek, 2, sd)
#Calculate sd1i
auditdis_vgdaysweek_n1i <- colSums(!is.na(auditdis_yes_ds2_vgdaysweek))
#Calculate n1i
auditdis_no_ds2_vgdaysweek <- subset(auditdis_no_ds2, select =
c(vgdaysweek)) #Subset 2i data
auditdis_vgdaysweek_m2i <- apply(auditdis_no_ds2_vgdaysweek, 2, mean)
#Calculate m2i
auditdis_vgdaysweek_sd2i <- apply(auditdis_no_ds2_vgdaysweek, 2, sd)
#Calculate sd2i
auditdis_vgdaysweek_n2i <- colSums(!is.na(auditdis_no_ds2_vgdaysweek))
#Calculate n2i
ds2auditdis_meta <- data.frame(m1i = auditdis_vgdaysweek_m1i, sd1i =
auditdis_vgdaysweek_sd1i, n1i = auditdis_vgdaysweek_n1i, m2i =
auditdis_vgdaysweek_m2i, sd2i = auditdis_vgdaysweek_sd2i, n2i =
auditdis_vgdaysweek_n2i) #MA dataframe
ds2auditdis_meta <- escalc(measure="MD", m1i = m1i, sd1i = sd1i, n1i =
n1i, m2i = m2i, sd2i = sd2i, n2i = n2i, data=ds2auditdis_meta) #Calculate yi
and vi

#FE meta-analysis
(auditdis_meta <- rbind(ds1auditdis_meta, ds2auditdis_meta))
```

```
##        m1i     sd1i n1i      m2i     sd2i n2i     yi     vi
## 1 6.225806 1.667527  31 4.958333 2.239432 120 1.2675 0.1315
## 2 5.062500 1.842779  16 4.778481 2.199083 158 0.2840 0.2428
```

```
(auditdis_vgdaysweek_meta <- rma(yi=yi, vi=vi, measure="MD",
method="FE", data=auditdis_meta))
```

```
##
## Fixed-Effects Model (k = 2)
##
## Test for Heterogeneity:
## Q(df = 1) = 2.5837, p-val = 0.1080
##
## Model Results:
##
## estimate      se     zval     pval    ci.lb    ci.ub
##   0.9220   0.2921   3.1569   0.0016   0.3496   1.4945      **
##
## ---
## Signif. codes:  0 '***' 0.001 '**' 0.01 '*' 0.05 '.' 0.1 ' ' 1
```

AUD - year

Replicated Effect

Hours per day

Study 1

```
(vghrsday_audityear_ds1 <- t.test(vghrsday ~ alcsudyear, data=ds1audit))
```

```
##
##  Welch Two Sample t-test
##
## data:  vghrsday by alcsudyear
## t = -0.85426, df = 11.921, p-value = 0.4098
## alternative hypothesis: true difference in means is not equal to 0
## 95 percent confidence interval:
##  -1.8720486  0.8180917
## sample estimates:
## mean in group 0 mean in group 1
##        2.223022        2.750000
```

Study 2

```
(vghrsday_audityear_ds2 <- t.test(vghrsday ~ alcsudyear, data=ds2audit))
```

```
## 
##  Welch Two Sample t-test
## 
## data:  vghrsday by alcsudyear
## t = 0.53456, df = 10.453, p-value = 0.6041
## alternative hypothesis: true difference in means is not equal to 0
## 95 percent confidence interval:
##  -0.8874763  1.4520614
## sample estimates:
## mean in group 0 mean in group 1 
##        3.425150        3.142857
```

Hours per week

Study 1

```
(vghrsweek_audityear_ds1 <- t.test(vghrsweek ~ alcsudyear, data=ds1audit))
```

```
## 
##  Welch Two Sample t-test
## 
## data:  vghrsweek by alcsudyear
## t = -1.0825, df = 11.964, p-value = 0.3003
## alternative hypothesis: true difference in means is not equal to 0
## 95 percent confidence interval:
##  -14.900581   5.010893
## sample estimates:
## mean in group 0 mean in group 1 
##        12.38849        17.33333
```

Study 2

```
(vghrsweek_audityear_ds2 <- t.test(vghrsweek ~ alcsudyear, data=ds2audit))
```

```
## 
##  Welch Two Sample t-test
## 
## data:  vghrsweek by alcsudyear
## t = 1.2297, df = 28.353, p-value = 0.2289
## alternative hypothesis: true difference in means is not equal to 0
## 95 percent confidence interval:
##  -1.930179  7.736852
## sample estimates:
## mean in group 0 mean in group 1 
##        17.76048        14.85714
```

Days per week

Study 1

```
(vgdaysweek_audityear_ds1 <- t.test(vgdaysweek ~ alcsudyear,
data=ds1audit))

##
##  Welch Two Sample t-test
##
## data:  vgdaysweek by alcsudyear
## t = -1.9119, df = 14.22, p-value = 0.07625
## alternative hypothesis: true difference in means is not equal to 0
## 95 percent confidence interval:
##  -2.1837186  0.1237666
## sample estimates:
## mean in group 0 mean in group 1
##        5.136691        6.166667
```

Study 2

```
(vgdaysweek_audityear_ds2 <- t.test(vgdaysweek ~ alcsudyear,
data=ds2audit))

##
##  Welch Two Sample t-test
##
## data:  vgdaysweek by alcsudyear
## t = -0.53747, df = 6.8852, p-value = 0.6079
## alternative hypothesis: true difference in means is not equal to 0
## 95 percent confidence interval:
##  -1.908263  1.203387
## sample estimates:
## mean in group 0 mean in group 1
##        4.790419        5.142857
```

MD

Hours per day

```
#Study 1
audityear_yes_ds1_vghrsday <- subset(audityear_yes_ds1, select =
c(vghrsday)) #Subset 1i data
audityear_vghrsday_m1i <- apply(audityear_yes_ds1_vghrsday, 2, mean)
#Calculate m1i
audityear_vghrsday_sd1i <- apply(audityear_yes_ds1_vghrsday, 2, sd)
#Calculate sd1i
audityear_vghrsday_n1i <- colSums(!is.na(audityear_yes_ds1_vghrsday))
#Calculate n1i
```

```
audityear_no_ds1_vghrsday <- subset(audityear_no_ds1, select =
c(vghrsday)) #Subset 2i data
audityear_vghrsday_m2i <- apply(audityear_no_ds1_vghrsday, 2, mean)
#Calculate m2i
audityear_vghrsday_sd2i <- apply(audityear_no_ds1_vghrsday, 2, sd)
#Calculate sd2i
audityear_vghrsday_n2i <- colSums(!is.na(audityear_no_ds1_vghrsday))
#Calculate n2i
ds1audityear_meta <- data.frame(m1i = audityear_vghrsday_m1i, sd1i =
audityear_vghrsday_sd1i, n1i = audityear_vghrsday_n1i, m2i =
audityear_vghrsday_m2i, sd2i = audityear_vghrsday_sd2i, n2i =
audityear_vghrsday_n2i) #MA dataframe
ds1audityear_meta <- escalc(measure="MD", m1i = m1i, sd1i = sd1i, n1i =
n1i, m2i = m2i, sd2i = sd2i, n2i = n2i, data=ds1audityear_meta) #Calculate
yi and vi

#Study 2
audityear_yes_ds2_vghrsday <- subset(audityear_yes_ds2, select =
c(vghrsday)) #Subset 1i data
audityear_vghrsday_m1i <- apply(audityear_yes_ds2_vghrsday, 2, mean)
#Calculate m1i
audityear_vghrsday_sd1i <- apply(audityear_yes_ds2_vghrsday, 2, sd)
#Calculate sd1i
audityear_vghrsday_n1i <- colSums(!is.na(audityear_yes_ds2_vghrsday))
#Calculate n1i
audityear_no_ds2_vghrsday <- subset(audityear_no_ds2, select =
c(vghrsday)) #Subset 2i data
audityear_vghrsday_m2i <- apply(audityear_no_ds2_vghrsday, 2, mean)
#Calculate m2i
audityear_vghrsday_sd2i <- apply(audityear_no_ds2_vghrsday, 2, sd)
#Calculate sd2i
audityear_vghrsday_n2i <- colSums(!is.na(audityear_no_ds2_vghrsday))
#Calculate n2i
ds2audityear_meta <- data.frame(m1i = audityear_vghrsday_m1i, sd1i =
audityear_vghrsday_sd1i, n1i = audityear_vghrsday_n1i, m2i =
audityear_vghrsday_m2i, sd2i = audityear_vghrsday_sd2i, n2i =
audityear_vghrsday_n2i) #MA dataframe
ds2audityear_meta <- escalc(measure="MD", m1i = m1i, sd1i = sd1i, n1i =
n1i, m2i = m2i, sd2i = sd2i, n2i = n2i, data=ds2audityear_meta) #Calculate
yi and vi

#FE meta-analysis
(audityear_meta <- rbind(ds1audityear_meta, ds2audityear_meta))

##        m1i     sd1i n1i      m2i     sd2i n2i      yi     vi
## 1 2.750000 2.094365  12 2.223022 1.444847 139  0.5270 0.3805
## 2 3.142857 1.214986   7 3.425150 3.369615 167 -0.2823 0.2789

(audityear_vghrsday_meta <- rma(yi=yi, vi=vi, measure="MD",
method="FE", data=audityear_meta))
```

```
##
## Fixed-Effects Model (k = 2)
##
## Test for Heterogeneity:
## Q(df = 1) = 0.9932, p-val = 0.3190
##
## Model Results:
##
## estimate      se    zval    pval    ci.lb    ci.ub
##   0.0600  0.4012  0.1494  0.8812  -0.7263  0.8462
##
## ---
## Signif. codes:  0 '***' 0.001 '**' 0.01 '*' 0.05 '.' 0.1 ' ' 1
```

Hours per week

```
#Study 1
audityear_yes_ds1_vghrsweek <- subset(audityear_yes_ds1, select =
c(vghrsweek)) #Subset 1i data
audityear_vghrsweek_m1i <- apply(audityear_yes_ds1_vghrsweek, 2, mean)
#Calculate m1i
audityear_vghrsweek_sd1i <- apply(audityear_yes_ds1_vghrsweek, 2, sd)
#Calculate sd1i
audityear_vghrsweek_n1i <- colSums(!is.na(audityear_yes_ds1_vghrsweek))
#Calculate n1i
audityear_no_ds1_vghrsweek <- subset(audityear_no_ds1, select =
c(vghrsweek)) #Subset 2i data
audityear_vghrsweek_m2i <- apply(audityear_no_ds1_vghrsweek, 2, mean)
#Calculate m2i
audityear_vghrsweek_sd2i <- apply(audityear_no_ds1_vghrsweek, 2, sd)
#Calculate sd2i
audityear_vghrsweek_n2i <- colSums(!is.na(audityear_no_ds1_vghrsweek))
#Calculate n2i
ds1audityear_meta <- data.frame(m1i = audityear_vghrsweek_m1i, sd1i =
audityear_vghrsweek_sd1i, n1i = audityear_vghrsweek_n1i, m2i =
audityear_vghrsweek_m2i, sd2i = audityear_vghrsweek_sd2i, n2i =
audityear_vghrsweek_n2i) #MA dataframe
ds1audityear_meta <- escalc(measure="MD", m1i = m1i, sd1i = sd1i, n1i =
n1i, m2i = m2i, sd2i = sd2i, n2i = n2i, data=ds1audityear_meta) #Calculate
yi and vi

#Study 2
audityear_yes_ds2_vghrsweek <- subset(audityear_yes_ds2, select =
c(vghrsweek)) #Subset 1i data
audityear_vghrsweek_m1i <- apply(audityear_yes_ds2_vghrsweek, 2, mean)
#Calculate m1i
audityear_vghrsweek_sd1i <- apply(audityear_yes_ds2_vghrsweek, 2, sd)
#Calculate sd1i
audityear_vghrsweek_n1i <- colSums(!is.na(audityear_yes_ds2_vghrsweek))
```

```
#Calculate n1i
audityear_no_ds2_vghrsweek <- subset(audityear_no_ds2, select =
c(vghrsweek)) #Subset 2i data
audityear_vghrsweek_m2i <- apply(audityear_no_ds2_vghrsweek, 2, mean)
#Calculate m2i
audityear_vghrsweek_sd2i <- apply(audityear_no_ds2_vghrsweek, 2, sd)
#Calculate sd2i
audityear_vghrsweek_n2i <- colSums(!is.na(audityear_no_ds2_vghrsweek))
#Calculate n2i
ds2audityear_meta <- data.frame(m1i = audityear_vghrsweek_m1i, sd1i =
audityear_vghrsweek_sd1i, n1i = audityear_vghrsweek_n1i, m2i =
audityear_vghrsweek_m2i, sd2i = audityear_vghrsweek_sd2i, n2i =
audityear_vghrsweek_n2i) #MA dataframe
ds2audityear_meta <- escalc(measure="MD", m1i = m1i, sd1i = sd1i, n1i =
n1i, m2i = m2i, sd2i = sd2i, n2i = n2i, data=ds2audityear_meta) #Calculate
yi and vi

#FE meta-analysis
(audityear_meta <- rbind(ds1audityear_meta, ds2audityear_meta))

##        m1i      sd1i n1i      m2i      sd2i n2i      yi      vi
## 1 17.33333 15.493889  12 12.38849 10.93360 139  4.9448 20.8651
## 2 14.85714  4.180453   7 17.76048 22.67056 167 -2.9033  5.5742

(audityear_vghrsweek_meta <- rma(yi=yi, vi=vi, measure="MD",
method="FE", data=audityear_meta))

##
## Fixed-Effects Model (k = 2)
##
## Test for Heterogeneity:
## Q(df = 1) = 2.3296, p-val = 0.1269
##
## Model Results:
##
## estimate      se     zval     pval    ci.lb    ci.ub
##  -1.2487   2.0974  -0.5954   0.5516  -5.3595   2.8621
##
## ---
## Signif. codes:  0 '***' 0.001 '**' 0.01 '*' 0.05 '.' 0.1 ' ' 1
```

Days per week

```
#Study 1
audityear_yes_ds1_vgdaysweek <- subset(audityear_yes_ds1, select =
c(vgdaysweek)) #Subset 1i data
audityear_vgdaysweek_m1i <- apply(audityear_yes_ds1_vgdaysweek, 2,
mean) #Calculate m1i
audityear_vgdaysweek_sd1i <- apply(audityear_yes_ds1_vgdaysweek, 2, sd)
#Calculate sd1i
```

```r
auditsyear_vgdaysweek_n1i <- colSums(!
is.na(audityear_yes_ds1_vgdaysweek)) #Calculate n1i
audityear_no_ds1_vgdaysweek <- subset(audityear_no_ds1, select =
c(vgdaysweek)) #Subset 2i data
audityear_vgdaysweek_m2i <- apply(audityear_no_ds1_vgdaysweek, 2,
mean) #Calculate m2i
audityear_vgdaysweek_sd2i <- apply(audityear_no_ds1_vgdaysweek, 2, sd)
#Calculate sd2i
audityear_vgdaysweek_n2i <- colSums(!
is.na(audityear_no_ds1_vgdaysweek)) #Calculate n2i
ds1audityear_meta <- data.frame(m1i = audityear_vgdaysweek_m1i, sd1i =
audityear_vgdaysweek_sd1i, n1i = audityear_vgdaysweek_n1i, m2i =
audityear_vgdaysweek_m2i, sd2i = audityear_vgdaysweek_sd2i, n2i =
audityear_vgdaysweek_n2i) #MA dataframe
ds1audityear_meta <- escalc(measure="MD", m1i = m1i, sd1i = sd1i, n1i =
n1i, m2i = m2i, sd2i = sd2i, n2i = n2i, data=ds1audityear_meta) #Calculate
yi and vi

#Study 2
audityear_yes_ds2_vgdaysweek <- subset(audityear_yes_ds2, select =
c(vgdaysweek)) #Subset 1i data
audityear_vgdaysweek_m1i <- apply(audityear_yes_ds2_vgdaysweek, 2,
mean) #Calculate m1i
audityear_vgdaysweek_sd1i <- apply(audityear_yes_ds2_vgdaysweek, 2, sd)
#Calculate sd1i
audityear_vgdaysweek_n1i <- colSums(!
is.na(audityear_yes_ds2_vgdaysweek)) #Calculate n1i
audityear_no_ds2_vgdaysweek <- subset(audityear_no_ds2, select =
c(vgdaysweek)) #Subset 2i data
audityear_vgdaysweek_m2i <- apply(audityear_no_ds2_vgdaysweek, 2,
mean) #Calculate m2i
audityear_vgdaysweek_sd2i <- apply(audityear_no_ds2_vgdaysweek, 2, sd)
#Calculate sd2i
audityear_vgdaysweek_n2i <- colSums(!
is.na(audityear_no_ds2_vgdaysweek)) #Calculate n2i
ds2audityear_meta <- data.frame(m1i = audityear_vgdaysweek_m1i, sd1i =
audityear_vgdaysweek_sd1i, n1i = audityear_vgdaysweek_n1i, m2i =
audityear_vgdaysweek_m2i, sd2i = audityear_vgdaysweek_sd2i, n2i =
audityear_vgdaysweek_n2i) #MA dataframe
ds2audityear_meta <- escalc(measure="MD", m1i = m1i, sd1i = sd1i, n1i =
n1i, m2i = m2i, sd2i = sd2i, n2i = n2i, data=ds2audityear_meta) #Calculate
yi and vi

#FE meta-analysis
(audityear_meta <- rbind(ds1audityear_meta, ds2audityear_meta))

##        m1i     sd1i n1i      m2i     sd2i n2i     yi     vi
## 1 6.166667 1.749459  12 5.136691 2.210651 139 1.0300 0.2902
## 2 5.142857 1.676163   7 4.790419 2.186576 167 0.3524 0.4300
```

```
(audityear_vgdaysweek_meta <- rma(yi=yi, vi=vi, measure="MD",
method="FE", data=audityear_meta))

##
## Fixed-Effects Model (k = 2)
##
## Test for Heterogeneity:
## Q(df = 1) = 0.6374, p-val = 0.4247
##
## Model Results:
##
## estimate      se    zval    pval    ci.lb   ci.ub
##   0.7570  0.4163  1.8185  0.0690  -0.0589  1.5728     .
##
## ---
## Signif. codes:  0 '***' 0.001 '**' 0.01 '*' 0.05 '.' 0.1 ' ' 1
```

AUD - month

Replicated Effect

Hours per day

Study 1 - CANNOT CALCULATE

Study 2 - CANNOT CALCULATE

Hours per week

Study 1 - CANNOT CALCULATE

Study 2 - CANNOT CALCULATE

Days per week

Study 1 - CANNOT CALCULATE

Study 2 - CANNOT CALCULATE

MD

Hours per day - CANNOT CALCULATE

Hours per week - CANNOT CALCULATE

Days per week - CANNOT CALCULATE

Cannabis - discharge

Replicated Effect

Hours per day

Study 1

```
(vghrsday_cannabisdis_ds1 <- t.test(vghrsday ~ alcsuddis,
data=ds1canscreen))

##
##  Welch Two Sample t-test
##
## data:  vghrsday by alcsuddis
## t = 0.12694, df = 50.83, p-value = 0.8995
## alternative hypothesis: true difference in means is not equal to 0
## 95 percent confidence interval:
```

```
##  -0.6667342  0.7567342
## sample estimates:
## mean in group 0 mean in group 1
##           1.925           1.880
```

Study 2

```
(vghrsday_cannabisdis_ds2 <- t.test(vghrsday ~ alcsuddis,
data=ds2canscreen))

##
##  Welch Two Sample t-test
##
## data:  vghrsday by alcsuddis
## t = 1.7056, df = 122.37, p-value = 0.09063
## alternative hypothesis: true difference in means is not equal to 0
## 95 percent confidence interval:
##  -0.1629379  2.1916520
## sample estimates:
## mean in group 0 mean in group 1
##        4.292135        3.277778
```

Hours per week

Study 1

```
(vghrsweek_cannabisdis_ds1 <- t.test(vghrsweek ~ alcsuddis,
data=ds1canscreen))

##
##  Welch Two Sample t-test
##
## data:  vghrsweek by alcsuddis
## t = -0.63036, df = 51.991, p-value = 0.5312
## alternative hypothesis: true difference in means is not equal to 0
## 95 percent confidence interval:
##  -6.797929  3.547929
## sample estimates:
## mean in group 0 mean in group 1
##           9.775          11.400
```

Study 2

```
(vghrsweek_cannabisdis_ds2 <- t.test(vghrsweek ~ alcsuddis,
data=ds2canscreen))

##
##  Welch Two Sample t-test
```

```
##
## data:  vghrsweek by alcsuddis
## t = 1.4711, df = 122.99, p-value = 0.1438
## alternative hypothesis: true difference in means is not equal to 0
## 95 percent confidence interval:
##  -2.017546 13.694824
## sample estimates:
## mean in group 0 mean in group 1
##        22.97753        17.13889
```

Days per week

Study 1

```
(vgdaysweek_cannabisdis_ds1 <- t.test(vgdaysweek ~ alcsuddis,
data=ds1canscreen))
```

```
##
##  Welch Two Sample t-test
##
## data:  vgdaysweek by alcsuddis
## t = -1.7787, df = 57.666, p-value = 0.08057
## alternative hypothesis: true difference in means is not equal to 0
## 95 percent confidence interval:
##  -2.0298917  0.1198917
## sample estimates:
## mean in group 0 mean in group 1
##           4.725           5.680
```

Study 2

```
(vgdaysweek_cannabisdis_ds2 <- t.test(vgdaysweek ~ alcsuddis,
data=ds2canscreen))
```

```
##
##  Welch Two Sample t-test
##
## data:  vgdaysweek by alcsuddis
## t = -1.2, df = 66.834, p-value = 0.2344
## alternative hypothesis: true difference in means is not equal to 0
## 95 percent confidence interval:
##  -1.4031763  0.3494934
## sample estimates:
## mean in group 0 mean in group 1
##        4.584270        5.111111
```

MD

Hours per day

```
#Study 1
cannabisdis_yes_ds1_vghrsday <- subset(cannabisdis_yes_ds1, select =
c(vghrsday)) #Subset 1i data
cannabisdis_vghrsday_m1i <- apply(cannabisdis_yes_ds1_vghrsday, 2, mean)
#Calculate m1i
cannabisdis_vghrsday_sd1i <- apply(cannabisdis_yes_ds1_vghrsday, 2, sd)
#Calculate sd1i
cannabisdis_vghrsday_n1i <- colSums(!is.na(cannabisdis_yes_ds1_vghrsday))
#Calculate n1i
cannabisdis_no_ds1_vghrsday <- subset(cannabisdis_no_ds1, select =
c(vghrsday)) #Subset 2i data
cannabisdis_vghrsday_m2i <- apply(cannabisdis_no_ds1_vghrsday, 2, mean)
#Calculate m2i
cannabisdis_vghrsday_sd2i <- apply(cannabisdis_no_ds1_vghrsday, 2, sd)
#Calculate sd2i
cannabisdis_vghrsday_n2i <- colSums(!is.na(cannabisdis_no_ds1_vghrsday))
#Calculate n2i
ds1cannabisdis_meta <- data.frame(m1i = cannabisdis_vghrsday_m1i, sd1i =
cannabisdis_vghrsday_sd1i, n1i = cannabisdis_vghrsday_n1i, m2i =
cannabisdis_vghrsday_m2i, sd2i = cannabisdis_vghrsday_sd2i, n2i =
cannabisdis_vghrsday_n2i) #MA dataframe
ds1cannabisdis_meta <- escalc(measure="MD", m1i = m1i, sd1i = sd1i, n1i
= n1i, m2i = m2i, sd2i = sd2i, n2i = n2i, data=ds1cannabisdis_meta)
#Calculate yi and vi

#Study 2
cannabisdis_yes_ds2_vghrsday <- subset(cannabisdis_yes_ds2, select =
c(vghrsday)) #Subset 1i data
cannabisdis_vghrsday_m1i <- apply(cannabisdis_yes_ds2_vghrsday, 2, mean)
#Calculate m1i
cannabisdis_vghrsday_sd1i <- apply(cannabisdis_yes_ds2_vghrsday, 2, sd)
#Calculate sd1i
cannabisdis_vghrsday_n1i <- colSums(!is.na(cannabisdis_yes_ds2_vghrsday))
#Calculate n1i
cannabisdis_no_ds2_vghrsday <- subset(cannabisdis_no_ds2, select =
c(vghrsday)) #Subset 2i data
cannabisdis_vghrsday_m2i <- apply(cannabisdis_no_ds2_vghrsday, 2, mean)
#Calculate m2i
cannabisdis_vghrsday_sd2i <- apply(cannabisdis_no_ds2_vghrsday, 2, sd)
#Calculate sd2i
cannabisdis_vghrsday_n2i <- colSums(!is.na(cannabisdis_no_ds2_vghrsday))
#Calculate n2i
ds2cannabisdis_meta <- data.frame(m1i = cannabisdis_vghrsday_m1i, sd1i =
cannabisdis_vghrsday_sd1i, n1i = cannabisdis_vghrsday_n1i, m2i =
cannabisdis_vghrsday_m2i, sd2i = cannabisdis_vghrsday_sd2i, n2i =
cannabisdis_vghrsday_n2i) #MA dataframe
```

```
ds2cannabisdis_meta <- escalc(measure="MD", m1i = m1i, sd1i = sd1i, n1i
= n1i, m2i = m2i, sd2i = sd2i, n2i = n2i, data=ds2cannabisdis_meta)
#Calculate yi and vi

#FE meta-analysis
(cannabisdis_meta <- rbind(ds1cannabisdis_meta, ds2cannabisdis_meta))

##        m1i     sd1i n1i      m2i     sd2i n2i      yi     vi
## 1 1.880000 1.394035  25 1.925000 1.384669  40 -0.0450 0.1257
## 2 3.277778 2.008711  36 4.292135 4.637305  89 -1.0144 0.3537

(cannabisdis_vghrsday_meta <- rma(yi=yi, vi=vi, measure="MD",
method="FE", data=cannabisdis_meta))

##
## Fixed-Effects Model (k = 2)
##
## Test for Heterogeneity:
## Q(df = 1) = 1.9602, p-val = 0.1615
##
## Model Results:
##
## estimate      se     zval     pval    ci.lb    ci.ub
##  -0.2991   0.3045  -0.9823   0.3260  -0.8959   0.2977
##
## ---
## Signif. codes:  0 '***' 0.001 '**' 0.01 '*' 0.05 '.' 0.1 ' ' 1
```

Hours per week

```
#Study 1
cannabisdis_yes_ds1_vghrsweek <- subset(cannabisdis_yes_ds1, select =
c(vghrsweek)) #Subset 1i data
cannabisdis_vghrsweek_m1i <- apply(cannabisdis_yes_ds1_vghrsweek, 2,
mean) #Calculate m1i
cannabisdis_vghrsweek_sd1i <- apply(cannabisdis_yes_ds1_vghrsweek, 2, sd)
#Calculate sd1i
cannabisdis_vghrsweek_n1i <- colSums(!
is.na(cannabisdis_yes_ds1_vghrsweek)) #Calculate n1i
cannabisdis_no_ds1_vghrsweek <- subset(cannabisdis_no_ds1, select =
c(vghrsweek)) #Subset 2i data
cannabisdis_vghrsweek_m2i <- apply(cannabisdis_no_ds1_vghrsweek, 2,
mean) #Calculate m2i
cannabisdis_vghrsweek_sd2i <- apply(cannabisdis_no_ds1_vghrsweek, 2, sd)
#Calculate sd2i
cannabisdis_vghrsweek_n2i <- colSums(!
is.na(cannabisdis_no_ds1_vghrsweek)) #Calculate n2i
ds1cannabisdis_meta <- data.frame(m1i = cannabisdis_vghrsweek_m1i, sd1i
= cannabisdis_vghrsweek_sd1i, n1i = cannabisdis_vghrsweek_n1i, m2i =
cannabisdis_vghrsweek_m2i, sd2i = cannabisdis_vghrsweek_sd2i, n2i =
```

```r
cannabisdis_vghrsweek_n2i) #MA dataframe
ds1cannabisdis_meta <- escalc(measure="MD", m1i = m1i, sd1i = sd1i, n1i
= n1i, m2i = m2i, sd2i = sd2i, n2i = n2i, data=ds1cannabisdis_meta)
#Calculate yi and vi

#Study 2
cannabisdis_yes_ds2_vghrsweek <- subset(cannabisdis_yes_ds2, select =
c(vghrsweek)) #Subset 1i data
cannabisdis_vghrsweek_m1i <- apply(cannabisdis_yes_ds2_vghrsweek, 2,
mean) #Calculate m1i
cannabisdis_vghrsweek_sd1i <- apply(cannabisdis_yes_ds2_vghrsweek, 2, sd)
#Calculate sd1i
cannabisdis_vghrsweek_n1i <- colSums(!
is.na(cannabisdis_yes_ds2_vghrsweek)) #Calculate n1i
cannabisdis_no_ds2_vghrsweek <- subset(cannabisdis_no_ds2, select =
c(vghrsweek)) #Subset 2i data
cannabisdis_vghrsweek_m2i <- apply(cannabisdis_no_ds2_vghrsweek, 2,
mean) #Calculate m2i
cannabisdis_vghrsweek_sd2i <- apply(cannabisdis_no_ds2_vghrsweek, 2, sd)
#Calculate sd2i
cannabisdis_vghrsweek_n2i <- colSums(!
is.na(cannabisdis_no_ds2_vghrsweek)) #Calculate n2i
ds2cannabisdis_meta <- data.frame(m1i = cannabisdis_vghrsweek_m1i, sd1i
= cannabisdis_vghrsweek_sd1i, n1i = cannabisdis_vghrsweek_n1i, m2i =
cannabisdis_vghrsweek_m2i, sd2i = cannabisdis_vghrsweek_sd2i, n2i =
cannabisdis_vghrsweek_n2i) #MA dataframe
ds2cannabisdis_meta <- escalc(measure="MD", m1i = m1i, sd1i = sd1i, n1i
= n1i, m2i = m2i, sd2i = sd2i, n2i = n2i, data=ds2cannabisdis_meta)
#Calculate yi and vi

#FE meta-analysis
(cannabisdis_meta <- rbind(ds1cannabisdis_meta, ds2cannabisdis_meta))

##        m1i     sd1i n1i      m2i     sd2i n2i      yi      vi
## 1 11.40000 10.02081  25  9.77500 10.25442  40  1.6250  6.6455
## 2 17.13889 12.63816  36 22.97753 31.73433  89 -5.8386 15.7521

(cannabisdis_vghrsweek_meta <- rma(yi=yi, vi=vi, measure="MD",
method="FE", data=cannabisdis_meta))

##
## Fixed-Effects Model (k = 2)
##
## Test for Heterogeneity:
## Q(df = 1) = 2.4871, p-val = 0.1148
##
## Model Results:
##
## estimate      se     zval     pval    ci.lb    ci.ub
##  -0.5895  2.1619  -0.2727   0.7851  -4.8267   3.6477
##
```

```
## ---
## Signif. codes:  0 '***' 0.001 '**' 0.01 '*' 0.05 '.' 0.1 ' ' 1
```

Days per week

```
#Study 1
cannabisdis_yes_ds1_vgdaysweek <- subset(cannabisdis_yes_ds1, select =
c(vgdaysweek)) #Subset 1i data
cannabisdis_vgdaysweek_m1i <- apply(cannabisdis_yes_ds1_vgdaysweek, 2,
mean) #Calculate m1i
cannabisdis_vgdaysweek_sd1i <- apply(cannabisdis_yes_ds1_vgdaysweek, 2,
sd) #Calculate sd1i
cannabisdis_vgdaysweek_n1i <- colSums(!
is.na(cannabisdis_yes_ds1_vgdaysweek)) #Calculate n1i
cannabisdis_no_ds1_vgdaysweek <- subset(cannabisdis_no_ds1, select =
c(vgdaysweek)) #Subset 2i data
cannabisdis_vgdaysweek_m2i <- apply(cannabisdis_no_ds1_vgdaysweek, 2,
mean) #Calculate m2i
cannabisdis_vgdaysweek_sd2i <- apply(cannabisdis_no_ds1_vgdaysweek, 2,
sd) #Calculate sd2i
cannabisdis_vgdaysweek_n2i <- colSums(!
is.na(cannabisdis_no_ds1_vgdaysweek)) #Calculate n2i
ds1cannabisdis_meta <- data.frame(m1i = cannabisdis_vgdaysweek_m1i,
sd1i = cannabisdis_vgdaysweek_sd1i, n1i = cannabisdis_vgdaysweek_n1i,
m2i = cannabisdis_vgdaysweek_m2i, sd2i = cannabisdis_vgdaysweek_sd2i,
n2i = cannabisdis_vgdaysweek_n2i) #MA dataframe
ds1cannabisdis_meta <- escalc(measure="MD", m1i = m1i, sd1i = sd1i, n1i
= n1i, m2i = m2i, sd2i = sd2i, n2i = n2i, data=ds1cannabisdis_meta)
#Calculate yi and vi

#Study 2
cannabisdis_yes_ds2_vgdaysweek <- subset(cannabisdis_yes_ds2, select =
c(vgdaysweek)) #Subset 1i data
cannabisdis_vgdaysweek_m1i <- apply(cannabisdis_yes_ds2_vgdaysweek, 2,
mean) #Calculate m1i
cannabisdis_vgdaysweek_sd1i <- apply(cannabisdis_yes_ds2_vgdaysweek, 2,
sd) #Calculate sd1i
cannabisdis_vgdaysweek_n1i <- colSums(!
is.na(cannabisdis_yes_ds2_vgdaysweek)) #Calculate n1i
cannabisdis_no_ds2_vgdaysweek <- subset(cannabisdis_no_ds2, select =
c(vgdaysweek)) #Subset 2i data
cannabisdis_vgdaysweek_m2i <- apply(cannabisdis_no_ds2_vgdaysweek, 2,
mean) #Calculate m2i
cannabisdis_vgdaysweek_sd2i <- apply(cannabisdis_no_ds2_vgdaysweek, 2,
sd) #Calculate sd2i
cannabisdis_vgdaysweek_n2i <- colSums(!
is.na(cannabisdis_no_ds2_vgdaysweek)) #Calculate n2i
ds2cannabisdis_meta <- data.frame(m1i = cannabisdis_vgdaysweek_m1i,
sd1i = cannabisdis_vgdaysweek_sd1i, n1i = cannabisdis_vgdaysweek_n1i,
```

```
m2i = cannabisdis_vgdaysweek_m2i, sd2i = cannabisdis_vgdaysweek_sd2i,
n2i = cannabisdis_vgdaysweek_n2i) #MA dataframe
ds2cannabisdis_meta <- escalc(measure="MD", m1i = m1i, sd1i = sd1i, n1i
= n1i, m2i = m2i, sd2i = sd2i, n2i = n2i, data=ds2cannabisdis_meta)
#Calculate yi and vi

#FE meta-analysis
(cannabisdis_meta <- rbind(ds1cannabisdis_meta, ds2cannabisdis_meta))
```

```
##        m1i     sd1i n1i     m2i     sd2i n2i     yi     vi
## 1 5.680000 1.951922  25 4.72500 2.331364  40 0.9550 0.2883
## 2 5.111111 2.201010  36 4.58427 2.275407  89 0.5268 0.1927
```

```
(cannabisdis_vgdaysweek_meta <- rma(yi=yi, vi=vi, measure="MD",
method="FE", data=cannabisdis_meta))
```

```
##
## Fixed-Effects Model (k = 2)
##
## Test for Heterogeneity:
## Q(df = 1) = 0.3811, p-val = 0.5370
##
## Model Results:
##
## estimate      se     zval     pval    ci.lb    ci.ub
##   0.6984   0.3399   2.0549   0.0399   0.0323   1.3645       *
##
## ---
## Signif. codes:  0 '***' 0.001 '**' 0.01 '*' 0.05 '.' 0.1 ' ' 1
```

Cannabis - year

Replicated Effect

Hours per day

Study 1

```
(vghrsday_cannabisyear_ds1 <- t.test(vghrsday ~ alcsudyear,
data=ds1canscreen))
```

```
##
##  Welch Two Sample t-test
##
## data:  vghrsday by alcsudyear
## t = 0.30367, df = 23.968, p-value = 0.764
## alternative hypothesis: true difference in means is not equal to 0
## 95 percent confidence interval:
```

```
##  -0.7320251  0.9845761
## sample estimates:
## mean in group 0 mean in group 1
##        1.938776        1.812500
```

Study 2

```
(vghrsday_cannabisyear_ds2 <- t.test(vghrsday ~ alcsudyear,
data=ds2canscreen))

##
##  Welch Two Sample t-test
##
## data:  vghrsday by alcsudyear
## t = 2.1796, df = 76.735, p-value = 0.03235
## alternative hypothesis: true difference in means is not equal to 0
## 95 percent confidence interval:
##  0.1087565 2.4095586
## sample estimates:
## mean in group 0 mean in group 1
##        4.211538        2.952381
```

Hours per week

Study 1

```
(vghrsweek_cannabisyear_ds1 <- t.test(vghrsweek ~ alcsudyear,
data=ds1canscreen))

##
##  Welch Two Sample t-test
##
## data:  vghrsweek by alcsudyear
## t = -0.41554, df = 25.829, p-value = 0.6812
## alternative hypothesis: true difference in means is not equal to 0
## 95 percent confidence interval:
##  -7.200108  4.779190
## sample estimates:
## mean in group 0 mean in group 1
##        10.10204        11.31250
```

Study 2

```
(vghrsweek_cannabisyear_ds2 <- t.test(vghrsweek ~ alcsudyear,
data=ds2canscreen))

##
##  Welch Two Sample t-test
```

```
##
## data:  vghrsweek by alcsudyear
## t = 2.0802, df = 64.431, p-value = 0.04149
## alternative hypothesis: true difference in means is not equal to 0
## 95 percent confidence interval:
##   0.3443176 16.9652062
## sample estimates:
## mean in group 0 mean in group 1
##        22.75000        14.09524
```

Days per week

Study 1

```
(vgdaysweek_cannabisyear_ds1 <- t.test(vgdaysweek ~ alcsudyear, data=ds1canscreen))

##
##  Welch Two Sample t-test
##
## data:  vgdaysweek by alcsudyear
## t = -2.467, df = 36.915, p-value = 0.01839
## alternative hypothesis: true difference in means is not equal to 0
## 95 percent confidence interval:
##  -2.3441132 -0.2298664
## sample estimates:
## mean in group 0 mean in group 1
##         4.77551         6.06250
```

Study 2

```
(vgdaysweek_cannabisyear_ds2 <- t.test(vgdaysweek ~ alcsudyear, data=ds2canscreen))

##
##  Welch Two Sample t-test
##
## data:  vgdaysweek by alcsudyear
## t = 0.64639, df = 27.298, p-value = 0.5234
## alternative hypothesis: true difference in means is not equal to 0
## 95 percent confidence interval:
##  -0.8028169  1.5418279
## sample estimates:
## mean in group 0 mean in group 1
##        4.798077        4.428571
```

MD

Hours per day

```
#Study 1
cannabisyear_yes_ds1_vghrsday <- subset(cannabisyear_yes_ds1, select =
c(vghrsday)) #Subset 1i data
cannabisyear_vghrsday_m1i <- apply(cannabisyear_yes_ds1_vghrsday, 2,
mean) #Calculate m1i
cannabisyear_vghrsday_sd1i <- apply(cannabisyear_yes_ds1_vghrsday, 2, sd)
#Calculate sd1i
cannabisyear_vghrsday_n1i <- colSums(!
is.na(cannabisyear_yes_ds1_vghrsday)) #Calculate n1i
cannabisyear_no_ds1_vghrsday <- subset(cannabisyear_no_ds1, select =
c(vghrsday)) #Subset 2i data
cannabisyear_vghrsday_m2i <- apply(cannabisyear_no_ds1_vghrsday, 2,
mean) #Calculate m2i
cannabisyear_vghrsday_sd2i <- apply(cannabisyear_no_ds1_vghrsday, 2, sd)
#Calculate sd2i
cannabisyear_vghrsday_n2i <- colSums(!
is.na(cannabisyear_no_ds1_vghrsday)) #Calculate n2i
ds1cannabisyear_meta <- data.frame(m1i = cannabisyear_vghrsday_m1i,
sd1i = cannabisyear_vghrsday_sd1i, n1i = cannabisyear_vghrsday_n1i, m2i =
cannabisyear_vghrsday_m2i, sd2i = cannabisyear_vghrsday_sd2i, n2i =
cannabisyear_vghrsday_n2i) #MA dataframe
ds1cannabisyear_meta <- escalc(measure="MD", m1i = m1i, sd1i = sd1i, n1i
= n1i, m2i = m2i, sd2i = sd2i, n2i = n2i, data=ds1cannabisyear_meta)
#Calculate yi and vi

#Study 2
cannabisyear_yes_ds2_vghrsday <- subset(cannabisyear_yes_ds2, select =
c(vghrsday)) #Subset 1i data
cannabisyear_vghrsday_m1i <- apply(cannabisyear_yes_ds2_vghrsday, 2,
mean) #Calculate m1i
cannabisyear_vghrsday_sd1i <- apply(cannabisyear_yes_ds2_vghrsday, 2, sd)
#Calculate sd1i
cannabisyear_vghrsday_n1i <- colSums(!
is.na(cannabisyear_yes_ds2_vghrsday)) #Calculate n1i
cannabisyear_no_ds2_vghrsday <- subset(cannabisyear_no_ds2, select =
c(vghrsday)) #Subset 2i data
cannabisyear_vghrsday_m2i <- apply(cannabisyear_no_ds2_vghrsday, 2,
mean) #Calculate m2i
cannabisyear_vghrsday_sd2i <- apply(cannabisyear_no_ds2_vghrsday, 2, sd)
#Calculate sd2i
cannabisyear_vghrsday_n2i <- colSums(!
is.na(cannabisyear_no_ds2_vghrsday)) #Calculate n2i
ds2cannabisyear_meta <- data.frame(m1i = cannabisyear_vghrsday_m1i,
sd1i = cannabisyear_vghrsday_sd1i, n1i = cannabisyear_vghrsday_n1i, m2i =
cannabisyear_vghrsday_m2i, sd2i = cannabisyear_vghrsday_sd2i, n2i =
cannabisyear_vghrsday_n2i) #MA dataframe
```

```
ds2cannabisyear_meta <- escalc(measure="MD", m1i = m1i, sd1i = sd1i, n1i
= n1i, m2i = m2i, sd2i = sd2i, n2i = n2i, data=ds2cannabisyear_meta)
#Calculate yi and vi

#FE meta-analysis
(cannabisyear_meta <- rbind(ds1cannabisyear_meta,
ds2cannabisyear_meta))
```

```
##        m1i     sd1i n1i      m2i     sd2i n2i      yi     vi
## 1 1.812500 1.470544  16 1.938776 1.360272  49 -0.1263 0.1729
## 2 2.952381 1.774153  21 4.211538 4.372627 104 -1.2592 0.3337
```

```
(cannabisyear_vghrsday_meta <- rma(yi=yi, vi=vi, measure="MD",
method="FE", data=cannabisyear_meta))
```

```
##
## Fixed-Effects Model (k = 2)
##
## Test for Heterogeneity:
## Q(df = 1) = 2.5332, p-val = 0.1115
##
## Model Results:
##
## estimate      se     zval     pval    ci.lb    ci.ub
##  -0.5129   0.3375  -1.5198   0.1286  -1.1744   0.1485
##
## ---
## Signif. codes:  0 '***' 0.001 '**' 0.01 '*' 0.05 '.' 0.1 ' ' 1
```

Hours per week

```
#Study 1
cannabisyear_yes_ds1_vghrsweek <- subset(cannabisyear_yes_ds1, select =
c(vghrsweek)) #Subset 1i data
cannabisyear_vghrsweek_m1i <- apply(cannabisyear_yes_ds1_vghrsweek, 2,
mean) #Calculate m1i
cannabisyear_vghrsweek_sd1i <- apply(cannabisyear_yes_ds1_vghrsweek, 2,
sd) #Calculate sd1i
cannabisyear_vghrsweek_n1i <- colSums(!
is.na(cannabisyear_yes_ds1_vghrsweek)) #Calculate n1i
cannabisyear_no_ds1_vghrsweek <- subset(cannabisyear_no_ds1, select =
c(vghrsweek)) #Subset 2i data
cannabisyear_vghrsweek_m2i <- apply(cannabisyear_no_ds1_vghrsweek, 2,
mean) #Calculate m2i
cannabisyear_vghrsweek_sd2i <- apply(cannabisyear_no_ds1_vghrsweek, 2,
sd) #Calculate sd2i
cannabisyear_vghrsweek_n2i <- colSums(!
is.na(cannabisyear_no_ds1_vghrsweek)) #Calculate n2i
ds1cannabisyear_meta <- data.frame(m1i = cannabisyear_vghrsweek_m1i,
sd1i = cannabisyear_vghrsweek_sd1i, n1i = cannabisyear_vghrsweek_n1i,
```

```
m2i = cannabisyear_vghrsweek_m2i, sd2i = cannabisyear_vghrsweek_sd2i,
n2i = cannabisyear_vghrsweek_n2i) #MA dataframe
ds1cannabisyear_meta <- escalc(measure="MD", m1i = m1i, sd1i = sd1i, n1i
= n1i, m2i = m2i, sd2i = sd2i, n2i = n2i, data=ds1cannabisyear_meta)
#Calculate yi and vi

#Study 2
cannabisyear_yes_ds2_vghrsweek <- subset(cannabisyear_yes_ds2, select =
c(vghrsweek)) #Subset 1i data
cannabisyear_vghrsweek_m1i <- apply(cannabisyear_yes_ds2_vghrsweek, 2,
mean) #Calculate m1i
cannabisyear_vghrsweek_sd1i <- apply(cannabisyear_yes_ds2_vghrsweek, 2,
sd) #Calculate sd1i
cannabisyear_vghrsweek_n1i <- colSums(!
is.na(cannabisyear_yes_ds2_vghrsweek)) #Calculate n1i
cannabisyear_no_ds2_vghrsweek <- subset(cannabisyear_no_ds2, select =
c(vghrsweek)) #Subset 2i data
cannabisyear_vghrsweek_m2i <- apply(cannabisyear_no_ds2_vghrsweek, 2,
mean) #Calculate m2i
cannabisyear_vghrsweek_sd2i <- apply(cannabisyear_no_ds2_vghrsweek, 2,
sd) #Calculate sd2i
cannabisyear_vghrsweek_n2i <- colSums(!
is.na(cannabisyear_no_ds2_vghrsweek)) #Calculate n2i
ds2cannabisyear_meta <- data.frame(m1i = cannabisyear_vghrsweek_m1i,
sd1i = cannabisyear_vghrsweek_sd1i, n1i = cannabisyear_vghrsweek_n1i,
m2i = cannabisyear_vghrsweek_m2i, sd2i = cannabisyear_vghrsweek_sd2i,
n2i = cannabisyear_vghrsweek_n2i) #MA dataframe
ds2cannabisyear_meta <- escalc(measure="MD", m1i = m1i, sd1i = sd1i, n1i
= n1i, m2i = m2i, sd2i = sd2i, n2i = n2i, data=ds2cannabisyear_meta)
#Calculate yi and vi

#FE meta-analysis
(cannabisyear_meta <- rbind(ds1cannabisyear_meta,
ds2cannabisyear_meta))

##        m1i     sd1i n1i      m2i     sd2i n2i      yi      vi
## 1 11.31250 10.08444  16 10.10204 10.21487  49  1.2105  8.4854
## 2 14.09524 13.67445  21 22.75000 29.56604 104 -8.6548 17.3096

(cannabisyear_vghrsweek_meta <- rma(yi=yi, vi=vi, measure="MD",
method="FE", data=cannabisyear_meta))

##
## Fixed-Effects Model (k = 2)
##
## Test for Heterogeneity:
## Q(df = 1) = 3.7729, p-val = 0.0521
##
## Model Results:
##
## estimate      se     zval     pval    ci.lb    ci.ub
```

```
## -2.0348  2.3862 -0.8527  0.3938 -6.7117  2.6422
##
## ---
## Signif. codes:  0 '***' 0.001 '**' 0.01 '*' 0.05 '.' 0.1 ' ' 1
```

Days per week

```
#Study 1
cannabisyear_yes_ds1_vgdaysweek <- subset(cannabisyear_yes_ds1, select
= c(vgdaysweek)) #Subset 1i data
cannabisyear_vgdaysweek_m1i <- apply(cannabisyear_yes_ds1_vgdaysweek,
2, mean) #Calculate m1i
cannabisyear_vgdaysweek_sd1i <- apply(cannabisyear_yes_ds1_vgdaysweek,
2, sd) #Calculate sd1i
cannabisyear_vgdaysweek_n1i <- colSums(!
is.na(cannabisyear_yes_ds1_vgdaysweek)) #Calculate n1i
cannabisyear_no_ds1_vgdaysweek <- subset(cannabisyear_no_ds1, select =
c(vgdaysweek)) #Subset 2i data
cannabisyear_vgdaysweek_m2i <- apply(cannabisyear_no_ds1_vgdaysweek,
2, mean) #Calculate m2i
cannabisyear_vgdaysweek_sd2i <- apply(cannabisyear_no_ds1_vgdaysweek,
2, sd) #Calculate sd2i
cannabisyear_vgdaysweek_n2i <- colSums(!
is.na(cannabisyear_no_ds1_vgdaysweek)) #Calculate n2i
ds1cannabisyear_meta <- data.frame(m1i = cannabisyear_vgdaysweek_m1i,
sd1i = cannabisyear_vgdaysweek_sd1i, n1i = cannabisyear_vgdaysweek_n1i,
m2i = cannabisyear_vgdaysweek_m2i, sd2i =
cannabisyear_vgdaysweek_sd2i, n2i = cannabisyear_vgdaysweek_n2i) #MA
dataframe
ds1cannabisyear_meta <- escalc(measure="MD", m1i = m1i, sd1i = sd1i, n1i
= n1i, m2i = m2i, sd2i = sd2i, n2i = n2i, data=ds1cannabisyear_meta)
#Calculate yi and vi

#Study 2
cannabisyear_yes_ds2_vgdaysweek <- subset(cannabisyear_yes_ds2, select
= c(vgdaysweek)) #Subset 1i data
cannabisyear_vgdaysweek_m1i <- apply(cannabisyear_yes_ds2_vgdaysweek,
2, mean) #Calculate m1i
cannabisyear_vgdaysweek_sd1i <- apply(cannabisyear_yes_ds2_vgdaysweek,
2, sd) #Calculate sd1i
cannabisyear_vgdaysweek_n1i <- colSums(!
is.na(cannabisyear_yes_ds2_vgdaysweek)) #Calculate n1i
cannabisyear_no_ds2_vgdaysweek <- subset(cannabisyear_no_ds2, select =
c(vgdaysweek)) #Subset 2i data
cannabisyear_vgdaysweek_m2i <- apply(cannabisyear_no_ds2_vgdaysweek,
2, mean) #Calculate m2i
cannabisyear_vgdaysweek_sd2i <- apply(cannabisyear_no_ds2_vgdaysweek,
2, sd) #Calculate sd2i
cannabisyear_vgdaysweek_n2i <- colSums(!
```

```
is.na(cannabisyear_no_ds2_vgdaysweek)) #Calculate n2i
ds2cannabisyear_meta <- data.frame(m1i = cannabisyear_vgdaysweek_m1i,
sd1i = cannabisyear_vgdaysweek_sd1i, n1i = cannabisyear_vgdaysweek_n1i,
m2i = cannabisyear_vgdaysweek_m2i, sd2i =
cannabisyear_vgdaysweek_sd2i, n2i = cannabisyear_vgdaysweek_n2i) #MA
dataframe
ds2cannabisyear_meta <- escalc(measure="MD", m1i = m1i, sd1i = sd1i, n1i
= n1i, m2i = m2i, sd2i = sd2i, n2i = n2i, data=ds2cannabisyear_meta)
#Calculate yi and vi

#FE meta-analysis
(cannabisyear_meta <- rbind(ds1cannabisyear_meta,
ds2cannabisyear_meta))

##        m1i     sd1i n1i      m2i     sd2i n2i      yi     vi
## 1 6.062500 1.611159  16 4.775510 2.320787  49  1.2870 0.2722
## 2 4.428571 2.420153  21 4.798077 2.231199 104 -0.3695 0.3268

(cannabisyear_vgdaysweek_meta <- rma(yi=yi, vi=vi, measure="MD",
method="FE", data=cannabisyear_meta))

##
## Fixed-Effects Model (k = 2)
##
## Test for Heterogeneity:
## Q(df = 1) = 4.5814, p-val = 0.0323
##
## Model Results:
##
## estimate      se     zval     pval    ci.lb    ci.ub
##   0.5343   0.3853   1.3865   0.1656  -0.2210   1.2895
##
## ---
## Signif. codes:  0 '***' 0.001 '**' 0.01 '*' 0.05 '.' 0.1 ' ' 1
```

Cannabis - month

Replicated Effect

Hours per day

Study 1 - CANNOT CALCULATE

Study 2

```
(vghrsday_cannabismonth_ds2 <- t.test(vghrsday ~ alcsudmonth,
data=ds2canscreen))
```

```
##
##  Welch Two Sample t-test
##
## data:  vghrsday by alcsudmonth
## t = 0.91169, df = 8.7714, p-value = 0.3863
## alternative hypothesis: true difference in means is not equal to 0
## 95 percent confidence interval:
##  -1.044242  2.444802
## sample estimates:
## mean in group 0 mean in group 1
##        4.033613        3.333333
```

Hours per week

Study 1 - CANNOT CALCULATE

Study 2

```
(vghrsweek_cannabismonth_ds2 <- t.test(vghrsweek ~ alcsudmonth,
data=ds2canscreen))
```

```
##
##  Welch Two Sample t-test
##
## data:  vghrsweek by alcsudmonth
## t = 0.66748, df = 7.9026, p-value = 0.5235
## alternative hypothesis: true difference in means is not equal to 0
## 95 percent confidence interval:
##  -9.386757 17.011406
## sample estimates:
## mean in group 0 mean in group 1
##        21.47899        17.66667
```

Days per week

Study 1 - CANNOT CALCULATE

Study 2

```
(vgdaysweek_cannabismonth_ds2 <- t.test(vgdaysweek ~ alcsudmonth,
data=ds2canscreen))
```

```
##
##  Welch Two Sample t-test
##
## data:  vgdaysweek by alcsudmonth
## t = -0.29073, df = 5.5096, p-value = 0.7819
## alternative hypothesis: true difference in means is not equal to 0
## 95 percent confidence interval:
```

```
##  -2.662564  2.107942
## sample estimates:
## mean in group 0 mean in group 1
##        4.722689        5.000000
```

MD

Hours per day

```
#Study 1
cannabismonth_yes_ds1_vghrsday <- subset(cannabismonth_yes_ds1, select
= c(vghrsday)) #Subset 1i data
cannabismonth_vghrsday_m1i <- apply(cannabismonth_yes_ds1_vghrsday, 2,
mean) #Calculate m1i
cannabismonth_vghrsday_sd1i <- apply(cannabismonth_yes_ds1_vghrsday,
2, sd) #Calculate sd1i
cannabismonth_vghrsday_n1i <- colSums(!
is.na(cannabismonth_yes_ds1_vghrsday)) #Calculate n1i
cannabismonth_no_ds1_vghrsday <- subset(cannabismonth_no_ds1, select =
c(vghrsday)) #Subset 2i data
cannabismonth_vghrsday_m2i <- apply(cannabismonth_no_ds1_vghrsday, 2,
mean) #Calculate m2i
cannabismonth_vghrsday_sd2i <- apply(cannabismonth_no_ds1_vghrsday, 2,
sd) #Calculate sd2i
cannabismonth_vghrsday_n2i <- colSums(!
is.na(cannabismonth_no_ds1_vghrsday)) #Calculate n2i
ds1cannabismonth_meta <- data.frame(m1i =
cannabismonth_vghrsday_m1i, sd1i = cannabismonth_vghrsday_sd1i, n1i =
cannabismonth_vghrsday_n1i, m2i = cannabismonth_vghrsday_m2i, sd2i =
cannabismonth_vghrsday_sd2i, n2i = cannabismonth_vghrsday_n2i) #MA
dataframe
ds1cannabismonth_meta <- escalc(measure="MD", m1i = m1i, sd1i = sd1i,
n1i = n1i, m2i = m2i, sd2i = sd2i, n2i = n2i, data=ds1cannabismonth_meta)
#Calculate yi and vi

#Study 2
cannabismonth_yes_ds2_vghrsday <- subset(cannabismonth_yes_ds2, select
= c(vghrsday)) #Subset 1i data
cannabismonth_vghrsday_m1i <- apply(cannabismonth_yes_ds2_vghrsday, 2,
mean) #Calculate m1i
cannabismonth_vghrsday_sd1i <- apply(cannabismonth_yes_ds2_vghrsday,
2, sd) #Calculate sd1i
cannabismonth_vghrsday_n1i <- colSums(!
is.na(cannabismonth_yes_ds2_vghrsday)) #Calculate n1i
cannabismonth_no_ds2_vghrsday <- subset(cannabismonth_no_ds2, select =
c(vghrsday)) #Subset 2i data
cannabismonth_vghrsday_m2i <- apply(cannabismonth_no_ds2_vghrsday, 2,
mean) #Calculate m2i
cannabismonth_vghrsday_sd2i <- apply(cannabismonth_no_ds2_vghrsday, 2,
```

```
sd) #Calculate sd2i
cannabismonth_vghrsday_n2i <- colSums(!
is.na(cannabismonth_no_ds2_vghrsday)) #Calculate n2i
ds2cannabismonth_meta <- data.frame(m1i =
cannabismonth_vghrsday_m1i, sd1i = cannabismonth_vghrsday_sd1i, n1i =
cannabismonth_vghrsday_n1i, m2i = cannabismonth_vghrsday_m2i, sd2i =
cannabismonth_vghrsday_sd2i, n2i = cannabismonth_vghrsday_n2i) #MA
dataframe
ds2cannabismonth_meta <- escalc(measure="MD", m1i = m1i, sd1i = sd1i,
n1i = n1i, m2i = m2i, sd2i = sd2i, n2i = n2i, data=ds2cannabismonth_meta)
#Calculate yi and vi

#FE meta-analysis
(cannabismonth_meta <- rbind(ds1cannabismonth_meta,
ds2cannabismonth_meta))

##        m1i     sd1i n1i      m2i     sd2i n2i      yi     vi
## 1      NaN       NA   0 1.907692 1.377533  65      NA     NA
## 2 3.333333 1.632993   6 4.033613 4.161838 119 -0.7003 0.5900

(cannabismonth_vghrsday_meta <- rma(yi=yi, vi=vi, measure="MD",
method="FE", data=cannabismonth_meta))

##
## Fixed-Effects Model (k = 1)
##
## Test for Heterogeneity:
## Q(df = 0) = 0.0000, p-val = 1.0000
##
## Model Results:
##
## estimate      se     zval     pval    ci.lb    ci.ub
##  -0.7003   0.7681  -0.9117   0.3619  -2.2058   0.8052
##
## ---
## Signif. codes:  0 '***' 0.001 '**' 0.01 '*' 0.05 '.' 0.1 ' ' 1
```

Hours per week

```
#Study 1
cannabismonth_yes_ds1_vghrsweek <- subset(cannabismonth_yes_ds1,
select = c(vghrsweek)) #Subset 1i data
cannabismonth_vghrsweek_m1i <-
apply(cannabismonth_yes_ds1_vghrsweek, 2, mean) #Calculate m1i
cannabismonth_vghrsweek_sd1i <-
apply(cannabismonth_yes_ds1_vghrsweek, 2, sd) #Calculate sd1i
cannabismonth_vghrsweek_n1i <- colSums(!
is.na(cannabismonth_yes_ds1_vghrsweek)) #Calculate n1i
cannabismonth_no_ds1_vghrsweek <- subset(cannabismonth_no_ds1, select
= c(vghrsweek)) #Subset 2i data
```

```
cannabismonth_vghrsweek_m2i <- apply(cannabismonth_no_ds1_vghrsweek,
2, mean) #Calculate m2i
cannabismonth_vghrsweek_sd2i <- apply(cannabismonth_no_ds1_vghrsweek,
2, sd) #Calculate sd2i
cannabismonth_vghrsweek_n2i <- colSums(!
is.na(cannabismonth_no_ds1_vghrsweek)) #Calculate n2i
ds1cannabismonth_meta <- data.frame(m1i =
cannabismonth_vghrsweek_m1i, sd1i = cannabismonth_vghrsweek_sd1i, n1i
= cannabismonth_vghrsweek_n1i, m2i = cannabismonth_vghrsweek_m2i,
sd2i = cannabismonth_vghrsweek_sd2i, n2i =
cannabismonth_vghrsweek_n2i) #MA dataframe
ds1cannabismonth_meta <- escalc(measure="MD", m1i = m1i, sd1i = sd1i,
n1i = n1i, m2i = m2i, sd2i = sd2i, n2i = n2i, data=ds1cannabismonth_meta)
#Calculate yi and vi

#Study 2
cannabismonth_yes_ds2_vghrsweek <- subset(cannabismonth_yes_ds2,
select = c(vghrsweek)) #Subset 1i data
cannabismonth_vghrsweek_m1i <-
apply(cannabismonth_yes_ds2_vghrsweek, 2, mean) #Calculate m1i
cannabismonth_vghrsweek_sd1i <-
apply(cannabismonth_yes_ds2_vghrsweek, 2, sd) #Calculate sd1i
cannabismonth_vghrsweek_n1i <- colSums(!
is.na(cannabismonth_yes_ds2_vghrsweek)) #Calculate n1i
cannabismonth_no_ds2_vghrsweek <- subset(cannabismonth_no_ds2, select
= c(vghrsweek)) #Subset 2i data
cannabismonth_vghrsweek_m2i <- apply(cannabismonth_no_ds2_vghrsweek,
2, mean) #Calculate m2i
cannabismonth_vghrsweek_sd2i <- apply(cannabismonth_no_ds2_vghrsweek,
2, sd) #Calculate sd2i
cannabismonth_vghrsweek_n2i <- colSums(!
is.na(cannabismonth_no_ds2_vghrsweek)) #Calculate n2i
ds2cannabismonth_meta <- data.frame(m1i =
cannabismonth_vghrsweek_m1i, sd1i = cannabismonth_vghrsweek_sd1i, n1i
= cannabismonth_vghrsweek_n1i, m2i = cannabismonth_vghrsweek_m2i,
sd2i = cannabismonth_vghrsweek_sd2i, n2i =
cannabismonth_vghrsweek_n2i) #MA dataframe
ds2cannabismonth_meta <- escalc(measure="MD", m1i = m1i, sd1i = sd1i,
n1i = n1i, m2i = m2i, sd2i = sd2i, n2i = n2i, data=ds2cannabismonth_meta)
#Calculate yi and vi

#FE meta-analysis
(cannabismonth_meta <- rbind(ds1cannabismonth_meta,
ds2cannabismonth_meta))

##        m1i     sd1i n1i      m2i     sd2i n2i      yi      vi
## 1      NaN       NA   0 10.40000 10.11774  65      NA      NA
## 2 17.66667 12.46863   6 21.47899 28.25815 119 -3.8123 32.6214
```

```
(cannabismonth_vghrsweek_meta <- rma(yi=yi, vi=vi, measure="MD",
method="FE", data=cannabismonth_meta))

##
## Fixed-Effects Model (k = 1)
##
## Test for Heterogeneity:
## Q(df = 0) = 0.0000, p-val = 1.0000
##
## Model Results:
##
## estimate      se     zval     pval    ci.lb    ci.ub
##  -3.8123   5.7115  -0.6675   0.5045 -15.0067   7.3820
##
## ---
## Signif. codes:  0 '***' 0.001 '**' 0.01 '*' 0.05 '.' 0.1 ' ' 1
```

Days per week

```
#Study 1
cannabismonth_yes_ds1_vgdaysweek <- subset(cannabismonth_yes_ds1,
select = c(vgdaysweek)) #Subset 1i data
cannabismonth_vgdaysweek_m1i <-
apply(cannabismonth_yes_ds1_vgdaysweek, 2, mean) #Calculate m1i
cannabismonth_vgdaysweek_sd1i <-
apply(cannabismonth_yes_ds1_vgdaysweek, 2, sd) #Calculate sd1i
cannabismonth_vgdaysweek_n1i <- colSums(!
is.na(cannabismonth_yes_ds1_vgdaysweek)) #Calculate n1i
cannabismonth_no_ds1_vgdaysweek <- subset(cannabismonth_no_ds1,
select = c(vgdaysweek)) #Subset 2i data
cannabismonth_vgdaysweek_m2i <-
apply(cannabismonth_no_ds1_vgdaysweek, 2, mean) #Calculate m2i
cannabismonth_vgdaysweek_sd2i <-
apply(cannabismonth_no_ds1_vgdaysweek, 2, sd) #Calculate sd2i
cannabismonth_vgdaysweek_n2i <- colSums(!
is.na(cannabismonth_no_ds1_vgdaysweek)) #Calculate n2i
ds1cannabismonth_meta <- data.frame(m1i =
cannabismonth_vgdaysweek_m1i, sd1i = cannabismonth_vgdaysweek_sd1i,
n1i = cannabismonth_vgdaysweek_n1i, m2i =
cannabismonth_vgdaysweek_m2i, sd2i = cannabismonth_vgdaysweek_sd2i,
n2i = cannabismonth_vgdaysweek_n2i) #MA dataframe
ds1cannabismonth_meta <- escalc(measure="MD", m1i = m1i, sd1i = sd1i,
n1i = n1i, m2i = m2i, sd2i = sd2i, n2i = n2i, data=ds1cannabismonth_meta)
#Calculate yi and vi

#Study 2
cannabismonth_yes_ds2_vgdaysweek <- subset(cannabismonth_yes_ds2,
select = c(vgdaysweek)) #Subset 1i data
cannabismonth_vgdaysweek_m1i <-
```

```
apply(cannabismonth_yes_ds2_vgdaysweek, 2, mean) #Calculate m1i
cannabismonth_vgdaysweek_sd1i <-
apply(cannabismonth_yes_ds2_vgdaysweek, 2, sd) #Calculate sd1i
cannabismonth_vgdaysweek_n1i <- colSums(!
is.na(cannabismonth_yes_ds2_vgdaysweek)) #Calculate n1i
cannabismonth_no_ds2_vgdaysweek <- subset(cannabismonth_no_ds2,
select = c(vgdaysweek)) #Subset 2i data
cannabismonth_vgdaysweek_m2i <-
apply(cannabismonth_no_ds2_vgdaysweek, 2, mean) #Calculate m2i
cannabismonth_vgdaysweek_sd2i <-
apply(cannabismonth_no_ds2_vgdaysweek, 2, sd) #Calculate sd2i
cannabismonth_vgdaysweek_n2i <- colSums(!
is.na(cannabismonth_no_ds2_vgdaysweek)) #Calculate n2i
ds2cannabismonth_meta <- data.frame(m1i =
cannabismonth_vgdaysweek_m1i, sd1i = cannabismonth_vgdaysweek_sd1i,
n1i = cannabismonth_vgdaysweek_n1i, m2i =
cannabismonth_vgdaysweek_m2i, sd2i = cannabismonth_vgdaysweek_sd2i,
n2i = cannabismonth_vgdaysweek_n2i) #MA dataframe
ds2cannabismonth_meta <- escalc(measure="MD", m1i = m1i, sd1i = sd1i,
n1i = n1i, m2i = m2i, sd2i = sd2i, n2i = n2i, data=ds2cannabismonth_meta)
#Calculate yi and vi

#FE meta-analysis
(cannabismonth_meta <- rbind(ds1cannabismonth_meta,
ds2cannabismonth_meta))

##   m1i     sd1i n1i      m2i     sd2i n2i     yi     vi
## 1 NaN       NA   0 5.092308 2.227127  65     NA     NA
## 2   5 2.280351   6 4.722689 2.265902 119 0.2773 0.9098

(cannabismonth_vgdaysweek_meta <- rma(yi=yi, vi=vi, measure="MD",
method="FE", data=cannabismonth_meta))

##
## Fixed-Effects Model (k = 1)
##
## Test for Heterogeneity:
## Q(df = 0) = 0.0000, p-val = 1.0000
##
## Model Results:
##
## estimate      se    zval    pval    ci.lb   ci.ub
##   0.2773  0.9538  0.2907  0.7713  -1.5922  2.1468
##
## ---
## Signif. codes:  0 '***' 0.001 '**' 0.01 '*' 0.05 '.' 0.1 ' ' 1
```
